# Effectiveness of postsurgical rehabilitation following lumbar disc herniation surgery: A systematic review^☆^

**DOI:** 10.1016/j.bas.2024.102806

**Published:** 2024-04-16

**Authors:** Hainan Yu, Carol Cancelliere, Silvano Mior, Paulo Pereira, Margareta Nordin, Ginny Brunton, Jessica J. Wong, Heather M. Shearer, Gaelan Connell, Lauren Ead, Leslie Verville, Mana Rezai, Danny Myrtos, Dan Wang, Andrée-Anne Marchand, Andrew Romanelli, Darrin Germann, Daphne To, James J. Young, Danielle Southerst, Henry Candelaria, Sheilah Hogg-Johnson, Pierre Côté

**Affiliations:** a Faculty of Health Sciences, Ontario Tech University, Canada; b Institute for Disability and Rehabilitation Research, Faculty of Health Sciences, Ontario Tech University and Canadian Memorial Chiropractic College, Canada; c Undergraduate Education, Canadian Memorial Chiropractic College, Canada; d Research and Innovation, Canadian Memorial Chiropractic College, Canada; e Faculty of Medicine, University of Porto, Portugal; f Department of Neurosurgery - Centro Hospitalar Universitário S. João, Porto, Portugal; g Departments of Orthopedic Surgery and Environmental Medicine, Occupational and Industrial Orthopedic Center, NYU School of Medicine, New York University, New York, NY, USA; h Division of Graduate Studies, Canadian Memorial Chiropractic College, Canada; i Bloorview Research Institute, Holland Bloorview Kids Rehabilitation Hospital, Canada; j Department of Clinical Education and Diagnosis, Canadian Memorial Chiropractic College, Canada; k Department of Chiropractic, Université du Québec à Trois-Rivières, Canada; l Schroeder Arthritis Institute, Krembil Research Institute, University Health Network, Toronto, Canada; m Center for Muscle and Joint Health, Department of Sports Science and Clinical Biomechanics, University of Southern Denmark, Denmark; n Rapid Access Clinic for Low Back Pain, Trillium Health Partners, Mississauga, Ontario, Canada; o Epidemiology Division, Dalla Lana School of Public Health, University of Toronto, Canada; p Institute for Health Policy, Management and Evaluation, University of Toronto, Toronto, Ontario, Canada

**Keywords:** Enhanced postsurgical recovery, Rehabilitation, Disc herniation, Systematic review

## Abstract

**Introduction:**

The effectiveness of post-surgical rehabilitation following lumbar disc herniation (LDH) surgery is unclear.

**Research question:**

To investigate the effectiveness and safety of rehabilitation interventions initiated within three months post-surgery for adults treated surgically for LDH.

**Material and methods:**

This systematic review searched seven databases from inception to November 2023. Independent reviewers screened studies, assessed and extracted data, and rated the certainty of the evidence using the GRADE approach.

**Results:**

This systematic review retrieved 20,531 citations and included 25 randomized controlled trials. The high certainty evidence suggests that adding Pilates exercise to routine care and cognitive behavioral therapy may improve function immediately post-intervention (1 RCT), and that adding whole-body magnetic therapy to exercise, pharmacological and aquatic therapy may reduce low back pain intensity (1 RCT) immediately post-intervention. Compared to placebo, pregabalin did not reduce low back pain or leg pain intensity (1 RCT) (moderate to high certainty evidence). We found no differences between: 1) behavioral graded activity vs. physiotherapy (1 RCT); 2) exercise and education vs. neck massage or watchful waiting (1 RCT); 3) exercise, education, and in-hospital usual care vs. in-hospital usual care (1 RCT); 4) functional or staged exercise vs. usual post-surgical care including exercise (2 RCTs); and 5) supervised exercise with education vs. education (1 RCT). No studies assessed adverse events.

**Discussion and conclusion:**

Evidence on effective and safe post-surgical rehabilitation interventions is sparse. This review identified two interventions with potential short-term benefits (Pilates exercises, whole-body magnetic therapy) but safety is unclear, and one with an iatrogenic effect (pregabalin).

## Highlights

• The evidence on the rehabilitation of post-surgical lumbar disc herniation is sparse
• Pilates exercise may improve function in the short term
• Whole-body magnetic therapy may be associated with reducing post-surgical back pain
• Pregabalin may be iatrogenic as a post-surgical intervention for low back pain
• High-quality trials are urgently needed to inform post-surgical rehabilitation

## 1 Introduction

The annual prevalence of lumbar disc herniation (LDH) and radiculopathy is 2.2% in the general population (Konstantinou and Dunn, 2008). Although the prognosis of acute LDH and radiculopathy is favourable, 10% of patients with symptoms lasting more than six weeks consider lumbar surgery (Jordan et al., 2011). It is reported that 48% of all lumbar surgeries are for LDH with radiculopathy, making it the most common reason for lumbar surgery in the working-age population (Hu et al., 1997; Schroeder et al., 2016). In 2012, 176,330 lumbar discectomies were performed in the United States and in 2018, the incidence of lumbar discectomies was 58 per 100,000 person-years in Finland (Bernstein et al., 2017; Ponkilainen et al., 2021).

The World Health Organization (WHO) recommends post-surgical rehabilitation to achieve and maintain optimal functioning. (The World Health Organization) The WHO defines rehabilitation as, “a set of measures that assist individuals who experience, or are likely to experience, disability to achieve and maintain optimal functioning when interacting with their environments”. In this context, rehabilitation includes clinical and community-based interventions. To date, little is known about the most effective rehabilitation approaches to improve functioning following LDH surgery.

Previous systematic reviews assessing the effectiveness of clinical rehabilitation following LDH surgery have reported inconsistent findings (Oosterhuis et al., 2014; Ostelo et al., 2008; Rushton et al., 2011; Snowdon and Peiris, 2016). Three reviews reported moderate to very low certainty evidence that early active rehabilitation programs (including supervised/home exercise and education) improved pain and function compared to no treatment or sham for lumbar disc herniation/prolapse treated surgerically (Oosterhuis et al., 2014; Ostelo et al., 2008; Snowdon and Peiris, 2016). However, another review reported active rehabilitation (e.g., exercise, behavioural rehabilitation, or multimodal care) and control/sham interventions are associated with similar outcomes following lumbar discectomy (Rushton et al., 2011). These reviews need to be updated because of outdated searches, methodological limitations (e.g., meta-analysis of heterogeneous interventions, misclassification of rehabilitation interventions, mixed lumbar conditions/diagnoses), and inconsistent findings. In addition, previous reviews did not report on the safety of rehabilitation interventions after lumbar surgery. Therefore, the purpose of this systematic review is to evaluate the effectiveness and safety of post-surgical rehabilitation interventions delivered in any setting to improve function, pain, and other health outcomes in adults treated with LDH surgery.

## 2 Methods

### 2.1 Search strategy and selection criteria

This systematic review is registered on the International Prospective Register of Systematic Reviews (PROSPERO) (CRD42019134607) and its protocol is published in *BMJ Open* (Cancelliere et al., 2020). Modifications to the published protocol are reported in Appendices (Additional file 1). The reporting of this systematic review followed the statement on the Preferred Reporting Items for Systematic Reviews and Meta-Analyses (PRISMA) (Additional file 2, Appendices) (Page et al., 2021).

This review included studies that: 1) enrolled adults who underwent LDH surgery for leg pain with or without associated low back pain (LBP); 2) investigated rehabilitation interventions (Additional file 3, Appendices); and 3) reported patient-important outcomes (e.g., function, pain, adverse events) (Additional file 4, Appendices). The detailed eligibility criteria are described in Table 1.

**Table 1:** Eligibility criteria for selecting studies.

	Included	Excluded
**Population**	Adults (aged 18 years and older) who underwent LDH surgery for leg pain with or without associated LBP	- Adults with leg and/or LBP caused by conditions other than LDH (e.g., fracture, infection, tumour, osteoporosis, inflammatory arthritis, cauda equina syndrome, spinal stenosis, or spondylolisthesis) - Adults who had failed back surgery syndrome
**Intervention**	Rehabilitation interventions (pharmacological [e.g., NSAIDs, analgesics] and non-pharmacological) initiated within the first three months after surgery	- Interventions for procedural/wound pain alone (e.g., administered within the first week after surgery only) - Interventions solely conducted at the societal level (e.g., barrier removal initiatives)
**Comparison**	Placebo/sham, control (e.g., usual care, wait-list control, no intervention), or another intervention	
**Outcomes**	Patient-important outcomes as guided by the International Classification of Functioning, Disability and Health (ICF) framework (e.g., pain, function)^58^: - Primary outcome: function measures at any time following the completion of post-surgical rehabilitation - Secondary outcomes included pain intensity, adverse events related to the intervention, self-perceived recovery, quality of life, work outcomes, need for additional surgeries or healthcare, and symptoms of depression or anxiety	
**Study design**	RCTs (≥30 participants per arm at baseline), cohort or case-control studies (≥100 participants per group at baseline), or mixed methods studies published in peer-reviewed journals. The minimum sample size requirements were considered the minimum needed for the normal distribution for the sampling distribution of the mean.^12^	Pilot studies assessing feasibility, protocol studies, cross-sectional studies, case reports, case series, systematic reviews and other review papers, clinical practice guidelines, biomechanical studies, laboratory studies, cadaveric or animal studies, and conceptual papers
**Publication type**	Published in peer-reviewed journals without language restrictions	Letters, editorials, commentaries, unpublished manuscripts, dissertations, government reports, books, book chapters, conference proceedings, meeting abstracts, lectures and addresses, consensus development statements, and guideline statements

LDH: lumbar disc herniation; LBP: low back pain; NSAIDs: non-steroidal anti-inflammatory drugs; RCT: randomized controlled trial.

A health sciences librarian developed the search strategies (Additional file 5, Appendices) without language restrictions and searched MEDLINE (Ovid), Embase (Ovid), APA PsycInfo (Ovid), CINAHL (EBSCO*host*), the Index to Chiropractic Literature (Chiropractic Library Collaboration), the Cochrane Controlled Register of trials (Ovid), and the Rehabilitation & Sports Medicine Source (EBSCO*host*) from inception to November 14, 2023. The search strategies were reviewed by a second health sciences librarian using the Peer Review of Electronic Search Strategies (PRESS) Checklist (McGowan and Lefebvre, 2010; Sampson et al., 2009). The search terms included subject headings specific to each database (e.g., MeSH in MEDLINE) and free text words relevant to post-surgical rehabilitation and LDH treated by surgery. Reference lists of included articles were reviewed and the WHO International Clinical trials Registry Platform (ICTRP) (http://apps.who.int/trialsearch/) was searched.

Non-English articles (e.g., Czech, Danish) were translated into English by a professional translation company. One Chinese article was reviewed and extracted by two reviewers with Chinese as their first language. Pairs of reviewers independently screened titles/abstracts and full texts, extracted/reviewed data, and graded the certainty of evidence. Disagreements were resolved by consensus or a third reviewer. Authors were contacted to request missing or additional data for clarification when needed.

### 2.2 Data analysis

Pairs of reviewers independently assessed articles for risk of bias using the Scottish Intercollegiate Guidelines Network (SIGN) criteria for randomized clinical trials (RCTs) and cohort studies. SIGN criteria were used to evaluate the presence and impact of selection bias, information bias, and confounding on study results. This systematic review did not pre-define a quantitative score or a cutoff point to determine the methodological quality of studies. Rather, the SIGN criteria were used to assist reviewers in making an informed judgment about methodological quality of studies. Specifically, the SIGN criteria for RCTs focused on the following methodological aspects: (1) clarity of the research question, (2) randomization method, (3) allocation concealment, (4) blinding of treatment and outcome assessment, (5) baseline similarity between/among treatment groups, (6) similar co-intervention contamination, (7) validity and reliability of outcome measures, (8) drop-out rates, (9) intention-to-treat analysis, and (10) comparability of results across multiple study sites (where applicable). This systematic review categorized the scientific validity of each study as either low, moderate or high risk of bias (Cancelliere et al., 2020). Studies were considered to have a high risk of bias if reviewers considered that the methodological quality was markedly compromised due to biases and methodological flaws. This systematic review evaluated the clinical, statistical, and methodological homogeneity of studies where two or more studies examined the same intervention before conducting a meta-analysis (Cancelliere et al., 2020). Where a meta-analysis could not be performed, studies were narratively summarized according to the Synthesis Without Meta-analysis (SwiM) reporting guideline (Campbell et al., 2020).

We extracted data from articles using the Evidence for Policy and Practice Information and Coordinating (EPPI) Centre's EPPI-Reviewer website (ERWeb) (Thomas et al., 2020). Studies were synthesized according to: 1) outcomes and follow-up periods: short (>1 week to 3 months post-surgery), intermediate (>3 months to 1 year post-surgery), and long-term (>1 year post-surgery); 2) intervention type; and 3) comparison: sham/placebo, control or another intervention (McKenzie et al., 2019). For studies using multiple measures to assess the same outcome or multiple time points, this systematic review selected the most common outcome measure and time point used across the studies to maximize the synthesis of findings, or the longest follow-up reported in a given period. For example, if an outcome was measured at 6 weeks, and 6 and 9 months, this systematic review reported the findings at 6 weeks (since this is the only follow-up point in the short-term period) and 9 months (since this is the longest follow-up point in the intermediate time period). For studies that included total outcome measurement scores and sub-scores, this systematic review only assessed total scores. This systematic review excluded: 1) observational studies if an RCT examining the same post-surgical rehabilitation intervention was available; 2) studies reporting p-values only (i.e., no effect estimate) without data necessary to compute the effect estimate (e.g., calculating between-group mean differences in studies that report within-group mean change for each group); and 3) studies with incompletely reported outcomes or outcome measures (e.g., unclear score range, unspecified purpose of numerical rating scale) if authors could not be contacted.

To quantify the effectiveness of interventions, we extracted or computed effect estimates (e.g., mean differences, odds ratio or relative risk) and 95% CIs (confidence intervals). If computed, the 95% CI for the difference in mean change was based on the assumption that the pre- and post-intervention outcomes are highly correlated (r = 0.8) when the correlation and/or standard error of change were not reported (Abrams et al., 2005; Follmann et al., 1992). We used two criteria to determine whether an intervention was effective: 1) clinical importance for each outcome using clinical judgement and group consensus and 2) statistical significance (i.e., p < 0.05 or the 95% CI did not contain the null). Generally, an effect estimate of at least 10% of the range of the scale (for mean differences or median scores), or higher than 1.1 or lower than 0.9 for relative risk or odds ratio, was considered clinically important(Dworkin et al., 2008; Rubinstein et al., 2012). We described the effectiveness of interventions as either superior, no different, or inferior to placebo/sham, control or other intervention by considering statistical significance, clinical importance, and direction of the effect. An intervention was considered superior/inferior (depending on direction) to the comparison if the effect estimate was statistically significant and clinically important. An intervention was considered no different to the comparison if the effect estimate was: 1) not statistically nor clinically significant; 2) statistically, but not clinically significant; or 3) clinically, but not statistically significant. This systematic review assessed the safety of interventions by examining for adverse events reported in studies.

For the primary analysis, we analyzed studies with low/moderate risk of bias and adapted the GRADE approach to assess the certainty of evidence for each outcome as high, moderate, low, or very low by assessing five domains – risk of bias, directness, consistency, precision, and publication bias (Table 2). (Guyatt et al., 2008) Furthermore, we applied certainty of evidence ratings to single RCTs since meta-analyses could not be conducted. We assessed precision using between-group effect [point estimate (95% CI)]. This systematic review also assessed the potential impact of publication and reporting biases by searching and identifying relevant study protocols through the WHO International Clinical Trials Registry Platform (ICTRP) and comparing relevant protocols with the included studies. In addition, we used standardized statements for reporting effects: high, moderate, low, and very low certainty evidence (Table 3).

**Table 2:** Grading notes.

Risk of bias
Options are not serious, serious (rate certainty of evidence down one level, e.g., from high to moderate), and very serious (rate certainty of evidence down two levels, e.g., from high to low):
1. Not serious: study rated as ‘low risk of bias’ or ‘some concerns’ (e.g., unclear co-interventions, no detailed randomization method described but similar baseline characteristics between groups).
2. Serious: study rated as ‘high risk of bias’ with unbalanced baseline characteristics between groups, unclear co-interventions, high/unbalanced drop-out and/or unclear intention-to-treat analysis.
3. Very serious: study rated as ‘high risk of bias’ with unclear randomization sequence generation, inadequate allocation concealment and/or lack of blinding.
*Imprecision*
Options are not serious, serious (rate certainty of evidence down one level), and very serious (rate certainty of evidence down two levels). **Imprecision** assessed using between-group effect [point estimate (95% CI)].
1. Not serious: If the point estimate is not clinically important: the upper and lower boundaries of the CI do not cross a clinically important threshold; the CI may cross the null as long as neither boundary crosses a clinically important threshold. If the point estimate is clinically important: the CI does not cross the null and the boundaries do not cross a clinically important threshold.
2. Serious: If the point estimate is not clinically important: the CI may or may not cross the null but one of the boundaries crosses a clinically important threshold. If the point estimate is clinically important: the CI may cross the null but does not cross a clinically important threshold in the other direction.
3. Very serious: If the point estimate is or is not clinically important: the CI crosses the boundaries of both appreciable harm and benefit (i.e., very wide CI).
*Indirectness*
Options are not serious, serious (rate certainty of evidence down one level), and very serious (rate certainty of evidence down two levels). **Indirectness** assessed whether the patients, interventions, or outcomes are different from the research question under investigation.
*Inconsistency*
Options are not serious, serious (rate certainty of evidence down one level), and very serious (rate certainty of evidence down two levels). **Inconsistency** assessed effect estimate variance in direction or magnitude.
1. Not serious: effect estimates are consistent in direction and magnitude across studies.
2. Serious: effect estimates vary in magnitude across studies and the heterogeneity could not be explained.
3. Very serious: effect estimates vary in direction across studies and the heterogeneity could not be explained.
*Publication bias*
Publication bias assessed using funnel plot if possible, or based on available information from clinical trial registries.

**Table 3:** Certainty of evidence.

	Superior/inferior	No different
High/moderate certainty evidence	[Intervention] is superior/inferior to [comparison] for [outcome]	[Intervention] is no different to/than [comparison] for [outcome]
Low certainty evidence	[Intervention] may be superior/inferior to [comparison] for [outcome]	[Intervention] may be no different to/than [comparison] for [outcome]
Very low certainty evidence	We don't know if/It is uncertain whether [intervention] is superior/inferior to [comparison] for [outcome] because the certainty of this evidence is very low.	

Finally, we conducted sensitivity analysis by including studies with a high risk of bias in synthesis and comparing those results to the primary analysis. We prioritized the primary analysis in our review (Higgins et al., 2011).

### 2.3 Patient and public involvement

Patients and the public were not involved in the research conducted for this systematic review.

### 2.4 Role of the funding source

The funders of the study had no role in study design, data collection, data analysis, data interpretation, or writing of the report.

## 3 Results

### 3.1 Study selection

**Fig. 1:**
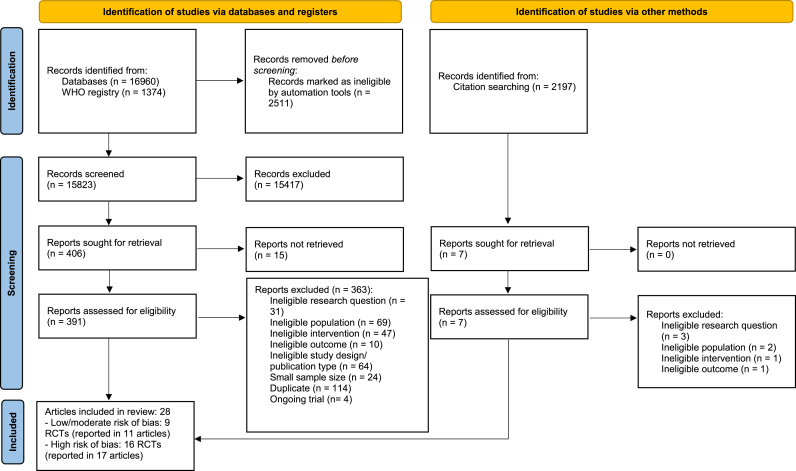
Identification and selection of articles.

Paired reviewers screened 20,531 citations including 398 full-text articles (Fig. 1; Additional file 6, Appendices). Of these, this systematic review included 25 RCTs (reported in 28 articles) that enrolled 2606 participants (Additional file 7, Appendices). Three hundred seventy articles were excluded due to: 1) ineligible research question (34 articles); 2) ineligible population (71 articles); 3) ineligible intervention (48 articles); 4) ineligible outcome (11 articles); 5) ineligible study designs (64 articles); 6) small sample size (24 articles); 7) duplicates (114 articles); and 7) ongoing trials (4 articles) (Additional file 6, Appendices).

### 3.2 Study characteristics

The mean age of participants across included studies ranged from 39 to 60 years, and 9.3%–59% were female. Participants received various surgeries including standard discectomy, micro-discectomy, discectomy with fusion, discectomy with foraminectomy/foraminotomy, discectomy with laminectomy, hemilaminectomy, laminectomy with fusion, lumbar intervertebral bone grafting and fusion, lumbar disc herniation nucleotomy, and lumbar transforaminal endoscopic surgery. Ten rehabilitation interventions were assessed: exercise (14 RCTs) (Aldemir and Gurkan, 2021; Abdi et al., 2023; Uysal et al., 2023; Zuo et al., 2021; Beneck et al., 2014; Choi et al., 2005; Häkkinen et al., 2005; He et al., 2021; Jentoft et al., 2020; Lu and Bai, 2020; Manniche et al., 1993; Paulsen et al., 2019, 2020; Skall et al., 1994; Zhang et al., 2018), education/self-management (2 RCTs) (Bono et al., 2017; Erdogan and Bulut, 2020), psychological intervention (1 RCT) (Ostelo et al., 2003a, 2003b), passive physical modalities (1 RCT) (Kulikov et al., 2018), multimodal care (education + exercise) (2 RCTs) (Ebenbichler et al., 2015; Erdogmus et al., 2007; Oosterhuis et al., 2017), pregabalin (1 RCT) (Zarei et al., 2016), acupuncture (1 RCT) (Zhao et al., 2008), systemic enzyme therapy (1 RCT) (Pekař and Steindler, 2010), Traditional Chinese Medicine (TCM) (1 RCT) (Fu and Ji, 2017), and Complementary and Alternative Therapy (CAM) (including usual care, supplementing qi, activating blood circulation and tonifying kidney therapy) (1 RCT) (Zheng et al., 2015). The duration of rehabilitation interventions varied: 1) during hospitalization (4 RCTs) (Jentoft et al., 2020; Bono et al., 2017; Erdogan and Bulut, 2020; Zheng et al., 2015); 2) 2 weeks (1 RCT) (Zarei et al., 2016); 3) six to 12 weeks (11 RCTs) (Aldemir and Gurkan, 2021; Abdi et al., 2023; Zuo et al., 2021; Beneck et al., 2014; Choi et al., 2005; He et al., 2021; Manniche et al., 1993; Paulsen et al., 2019, 2020; Skall et al., 1994; Ostelo et al., 2003a, 2003b; Ebenbichler et al., 2015; Erdogmus et al., 2007; Oosterhuis et al., 2017; Pekař and Steindler, 2010); 4) six to 12 months (2 RCTs) (Häkkinen et al., 2005; Lu and Bai, 2020); and 5) unspecified duration (4 RCTs) (Uysal et al., 2023; Zhang et al., 2018; Kulikov et al., 2018; Zhao et al., 2008; Fu and Ji, 2017). Main outcomes included LBP intensity (11 RCTs) (Aldemir and Gurkan, 2021; Abdi et al., 2023; Uysal et al., 2023; Jentoft et al., 2020; Lu and Bai, 2020; Paulsen et al., 2019, 2020; Bono et al., 2017; Ostelo et al., 2003a, 2003b; Kulikov et al., 2018; Ebenbichler et al., 2015; Erdogmus et al., 2007; Zarei et al., 2016), leg pain intensity (6 RCTs) (Aldemir and Gurkan, 2021; Jentoft et al., 2020; Paulsen et al., 2019, 2020; Bono et al., 2017; Ostelo et al., 2003a, 2003b; Zarei et al., 2016), pain intensity (unspecified location) (3 RCTs) (Pekař and Steindler, 2010; Fu and Ji, 2017; Zheng et al., 2015), function (16 RCTs) (Aldemir and Gurkan, 2021; Abdi et al., 2023; Uysal et al., 2023; Zuo et al., 2021; Choi et al., 2005; He et al., 2021; Jentoft et al., 2020; Lu and Bai, 2020; Paulsen et al., 2019, 2020; Bono et al., 2017; Erdogan and Bulut, 2020; Ostelo et al., 2003a, 2003b; Oosterhuis et al., 2017; Zhao et al., 2008; Pekař and Steindler, 2010; Zheng et al., 2015), and quality of life (8 RCTs) (Beneck et al., 2014; He et al., 2021; Lu and Bai, 2020; Paulsen et al., 2019, 2020; Zhang et al., 2018; Ostelo et al., 2003a, 2003b; Oosterhuis et al., 2017; Zheng et al., 2015).The RCTs were clinically heterogeneous, therefore, meta-analysis was not conducted (Deeks et al., 2019).

### 3.3 Risk of bias assessment

Nine RCTs reported in 11 articles (36%) had low/moderate risk of bias (Zuo et al., 2021; Beneck et al., 2014; He et al., 2021; Lu and Bai, 2020; Ostelo et al., 2003a, 2003b; Kulikov et al., 2018; Ebenbichler et al., 2015; Erdogmus et al., 2007; Oosterhuis et al., 2017; Zarei et al., 2016); with the remaining 16 RCTs reported in 17 articles considered to be at high risk of bias (Aldemir and Gurkan, 2021; Abdi et al., 2023; Uysal et al., 2023; Choi et al., 2005; Häkkinen et al., 2005; Jentoft et al., 2020; Manniche et al., 1993; Paulsen et al., 2019, 2020; Skall et al., 1994; Zhang et al., 2018; Bono et al., 2017; Erdogan and Bulut, 2020; Zhao et al., 2008; Pekař and Steindler, 2010; Fu and Ji, 2017; Zheng et al., 2015) (Additional file 8a, 8b, Appendices).

Studies minimized potential sources of bias related to: 1) generation of randomization sequence (60%) (Aldemir and Gurkan, 2021; Abdi et al., 2023; Zuo et al., 2021; Beneck et al., 2014; He et al., 2021; Jentoft et al., 2020; Lu and Bai, 2020; Manniche et al., 1993; Erdogan and Bulut, 2020; Ostelo et al., 2003a, 2003b; Ebenbichler et al., 2015; Erdogmus et al., 2007; Oosterhuis et al., 2017; Zarei et al., 2016; Fu and Ji, 2017; Zheng et al., 2015); 2) concealment of treatment allocation (24%) (Jentoft et al., 2020; Bono et al., 2017; Ostelo et al., 2003a, 2003b; Kulikov et al., 2018; Ebenbichler et al., 2015; Erdogmus et al., 2007; Oosterhuis et al., 2017); 3) blinding of patients (8%) (Lu and Bai, 2020; Zarei et al., 2016); 4) blinding of providers (4%) (Zarei et al., 2016); 5) blinding of outcome assessment (24%) (Beneck et al., 2014; Häkkinen et al., 2005; Manniche et al., 1993; Ostelo et al., 2003a, 2003b; Ebenbichler et al., 2015; Erdogmus et al., 2007; Zarei et al., 2016); 6) balance of baseline characteristics (48%) (Zuo et al., 2021; Beneck et al., 2014; Häkkinen et al., 2005; He et al., 2021; Lu and Bai, 2020; Bono et al., 2017; Ostelo et al., 2003a, 2003b; Kulikov et al., 2018; Ebenbichler et al., 2015; Erdogmus et al., 2007; Zarei et al., 2016; Zhao et al., 2008; Pekař and Steindler, 2010); and 7) validity and reliability of outcome measures (88%) (Abdi et al., 2023; Uysal et al., 2023; Zuo et al., 2021; Beneck et al., 2014; Choi et al., 2005; Häkkinen et al., 2005; Jentoft et al., 2020; Lu and Bai, 2020; Manniche et al., 1993; Paulsen et al., 2019, 2020; Skall et al., 1994; Bono et al., 2017; Erdogan and Bulut, 2020; Ostelo et al., 2003a, 2003b; Kulikov et al., 2018; Ebenbichler et al., 2015; Erdogmus et al., 2007; Oosterhuis et al., 2017; Zarei et al., 2016; Zhao et al., 2008; Pekař and Steindler, 2010; Fu and Ji, 2017; Zheng et al., 2015). Six RCTs (24%) had >20% missing outcome data(Beneck et al., 2014; Häkkinen et al., 2005; Paulsen et al., 2019, 2020; Bono et al., 2017; Ebenbichler et al., 2015; Erdogmus et al., 2007; Pekař and Steindler, 2010) and six RCTs (24%) did not report drop-out rates (Uysal et al., 2023; Zuo et al., 2021; Zarei et al., 2016; Zhao et al., 2008; Fu and Ji, 2017; Zheng et al., 2015). All relevant registered trials were published or ongoing except one RCT comparing exercise combined with education to education alone (data collection completed in April 2019, but author could not be reached). Therefore, publication or reporting biases is potentially minimal. (Therapeutic Exercises and Educational Sessions)

### 3.4 Certainty of evidence assessment

We applied the GRADE certainty of evidence ratings (i.e., high, moderate, low, very low) to single RCTs because meta-analyses could not be conducted. The method of grading the evidence is detailed in the footnotes of the evidence profile tables (Additional file 9, Appendices).

#### 3.4.1 Primary analysis: studies with low/moderate risk of bias

Nine low/moderate risk of bias RCTs were included in the primary analysis. They evaluated: 1) exercise(Zuo et al., 2021; Beneck et al., 2014; He et al., 2021; Lu and Bai, 2020); 2) multimodal care (Ebenbichler et al., 2015; Erdogmus et al., 2007; Oosterhuis et al., 2017); 3) passive physical modalities (Kulikov et al., 2018); 4) psychological intervention (Ostelo et al., 2003a, 2003b); and 5) oral pharmacological intervention (Zarei et al., 2016) (Additional file 9, Appendices).

##### 3.4.1.1 Function

Five RCTs assessed the effects of rehabilitation interventions on function: 1) multimodal care; 2) behavioural graded activity; and 3) exercise.

Supervised in-person Pilates exercise (30 min per session, 2 sessions per day over 6 weeks) provided additional benefits in improving function (MD 6.95 (5.58, 8.31) on Oswestry Disability Index [ODI, scale range 0–50]) when added to routine post-surgical care and cognitive behavioral therapy at the 6th week post-surgery in adults with fear of movement (37/68 on Tampa Scale of Kinesiophobia) and mixed surgery types (1 RCT, high certainty evidence) (Zuo et al., 2021). However, exercise provided remotely did not provide benefits when: 1) exercise provided remotely (an online communication platform to deliver texts, pictures and videos regarding functional exercises over 3 months after surgery) plus discharge education with two telephone follow-ups vs. discharge education with two telephone follow-ups alone in adults with first time LDH surgery (1 RCT, moderate certainty evidence) (He et al., 2021); and 2) remote staged exercise (graded exercise based on McKenzie's technology with remote online guidance initiated from the second week after surgery for the duration of 24 weeks) vs. routine post-surgical care focusing on lumbar muscle exercise without staging in adults with percutaneous transforaminal endoscopic discectomy (1 RCT, high certainty evidence) (Lu and Bai, 2020).

There is moderate to high certainty evidence of no differences in functional limitations reduction up to one year post-surgery for 1) multimodal care (i.e., education + individualized exercise initiated from the first week after discharge, 1–2 sessions per week, 30 min per session over 6–8 weeks) by physiotherapist plus usual post-surgical care during hospitalization vs. usual post-surgical care during hospitalization alone (1 RCT, high certainty evidence) (Oosterhuis et al., 2017); and 2) behavioural graded therapy (based on individual treatment goals and positive reinforcement to increase healthy behaviors and decrease pain behaviors, maximum 18 30-min sessions over 3 months) by physiotherapist vs. usual care by physiotherapists in adults with first time LDH surgery (1 RCT, moderate to high certainty evidence at different time points) (Ostelo et al., 2003a, 2003b).

##### 3.4.1.2 Pain

Five RCTs assessed the effectiveness of rehabilitation interventions on pain (Lu and Bai, 2020; Ostelo et al., 2003a, 2003b; Kulikov et al., 2018; Ebenbichler et al., 2015; Erdogmus et al., 2007; Zarei et al., 2016). In adults who had pain and discomfort in lumbar area within one month after lumbar discectomy, whole-body magnetic field therapy (10 sessions, initiated within the first month of the surgery) combined with exercise, pharmacological and aquatic therapy was slightly superior to exercise, pharmacological and aquatic therapy alone immediately post-intervention for reducing LBP intensity (MD -1.2 [−1.31 to −1.09]; scale range 0–10, 0 = no pain; 1 RCT, high certainty evidence) (Kulikov et al., 2018). Pregabalin was inferior to placebo (scale range 0–10, 0 = no pain; MD -1.58 [−2.90 to −0.26] for 1-day pregabalin (150 mg 12 and 24 h postoperatively); −1.49 [−2.75 to −0.23] for 14-day pregabalin (150 mg every 12 h postoperatively for 14 days)) in reducing LBP intensity up to 12 months following bilateral foraminotomy and interlaminar discectomy (1 RCT, moderate certainty evidence) (Zarei et al., 2016). However, pregabalin was no different to placebo in reducing leg pain up to 12 months post-surgery (1 RCT, moderate and high certainty evidence) (Zarei et al., 2016). Moreover, there is: 1) moderate certainty evidence that behavioral graded activity (based on individual treatment goals and positive reinforcement to increase healthy behaviors and decrease pain behaviors, maximum 18 30-min sessions over 3 months) by physiotherapist is no different than physiotherapy in reducing LBP or leg pain intensity in adults with first time LDH surgery (1 RCT) (Ostelo et al., 2003a, 2003b); 2) moderate to high certainty evidence that multimodal care (tailored instructions on education + exercise initiated from one week post-surgery, 20 30-min sessions over 12 weeks) is no different than neck massage or watchful waiting in reducing LBP intensity in adults with first time uncomplicated LDH surgery (1 RCT) (Ebenbichler et al., 2015; Erdogmus et al., 2007); and 3) high certainty evidence that remote staged exercise (graded exercise based on McKenzie's technology with remote online guidance initiated from the second week after surgery for the duration of 24 weeks) is no different than routine lumbar muscle exercise without staging in reducing LBP intensity in adults with percutaneous transforaminal endoscopic discectomy (1 RCT) (Lu and Bai, 2020).

##### 3.4.1.3 Quality of life

Five RCTs assessed the effectiveness of rehabilitation interventions on quality of life (Beneck et al., 2014; He et al., 2021; Lu and Bai, 2020; Ostelo et al., 2003a, 2003b; Oosterhuis et al., 2017). This systematic review found that up to one year post-surgery: 1) moderate to high certainty evidence that multimodal care (i.e., education + individualized exercise initiated from the first week after discharge, 1–2 sessions per week, 30 min per session over 6–8 weeks) provided by physiotherapists plus usual post-surgical care during hospitalization is no different than usual post-surgical care during hospitalization (1 RCT) (Oosterhuis et al., 2017); 2) high certainty evidence that exercise (initiated from 4 to 6 weeks after surgery, 3 sessions per week over 12 weeks) plus education provided by physiotherapists is no different than the same education by physiotherapists only in adults with single level microdiscectomy (1 RCT) (Beneck et al., 2014); 3) high certainty evidence that exercise (an online communication platform to deliver texts, pictures and videos regarding functional exercises over 3 months after surgery) provided remotely is no different than discharge education with two telephone follow-ups in adults with first time LDH surgery (1 RCT) (He et al., 2021); 4) moderate certainty evidence that remote staged exercise (graded exercise based on McKenzie's technology with remote online guidance initiated from the second week after surgery for the duration of 24 weeks) is no different than routine post-surgical care focusing on lumbar muscle exercise without staging in adults with percutaneous transforaminal endoscopic discectomy (1 RCT) (Lu and Bai, 2020); and 5) moderate to high certainty evidence that behavioural graded activity (based on individual treatment goals and positive reinforcement to increase healthy behaviors and decrease pain behaviors, maximum 18 30-min sessions over 3 months) by physiotherapist is no different than physiotherapy in adults with first time LDH surgery (1 RCT) (Ostelo et al., 2003a, 2003b).

##### 3.4.1.4 Self-perceived recovery

Two RCTs assessed the effectiveness of rehabilitation interventions on self-perceived recovery (Ostelo et al., 2003a, 2003b; Oosterhuis et al., 2017). This systematic review found low certainty evidence that up to one year post-surgery: 1) multimodal care (i.e., education, exercise initiated from the first week after discharge, 1–2 sessions per week, 30 min per session over 6–8 weeks) by physiotherapists plus usual post-surgical care during hospitalization may be no different than usual post-surgical care during hospitalization alone (1 RCT) (Oosterhuis et al., 2017); and 2) behavioral graded activity (based on individual treatment goals and positive reinforcement to increase healthy behaviors and decrease pain behaviors, maximum 18 30-min sessions over 3 months) by physiotherapist may be no different than physiotherapy in adults with first time LDH surgery (1 RCT) (Ostelo et al., 2003a, 2003b).

##### 3.4.1.5 Work outcomes, re-operation, kinesiophobia and pain catastrophizing, analgesic use, doctor visits

This systematic review found moderate to high certainty evidence of no difference in work outcomes, kinesiophobia and pain catastrophizing; and low certainty evidence of no difference in re-operation, analgesic use and doctor visits with behavioural graded activity (based on individual treatment goals and positive reinforcement to increase healthy behaviors and decrease pain behaviors, maximum 18 30-min sessions over 3 months) by physiotherapist compared to physiotherapy in adults with first time LDH surgery (1 RCT) (Ostelo et al., 2003a, 2003b).

#### 3.4.2 Adverse events

None of the nine RCTs assessed the safety of post-surgical rehabilitation interventions.

#### 3.4.3 Sensitivity analyses: studies with high risk of bias

This sensitivity analysis included 16 high risk of bias RCTs. These RCTs evaluated: 1) exercise (10 RCTs) (Aldemir and Gurkan, 2021; Abdi et al., 2023; Uysal et al., 2023; Choi et al., 2005; Häkkinen et al., 2005; Jentoft et al., 2020; Manniche et al., 1993; Paulsen et al., 2019, 2020; Skall et al., 1994; Zhang et al., 2018), 2) acupuncture (1 RCT) (Zhao et al., 2008), 3) education (2 RCTs) (Bono et al., 2017; Erdogan and Bulut, 2020), 4) systemic enzyme therapy (1 RCT) (Pekař and Steindler, 2010), 5), TCM (1 RCT) (Fu and Ji, 2017), and 6) CAM (1 RCT) (Zheng et al., 2015) (Additional file 9, Appendices). Results from the sensitivity analyses are similar to the primary analysis, but there are some differences (Table 4).

**Table 4:** Summary of evidence_Primary vs. sensitivity analysis Beneficial effect of interventions.

		Primary analysis				
		Supervised Pilates exercise	Whole-body magnetic field therapy			
**Sensitivity analysis**	Exercise, acupuncture, complementary and Alternative Therapy	**Function**				
	Exercise, traditional Chinese medicine, systemic enzyme		**Pain**			
	Traditional Chinese medicine			**Depression**		
	Traditional Chinese medicine				**Anxiety**	
	Systemic enzyme					**Recovery**

For exercise, there are 10 RCTs with high risk of bias. There is low certainty evidence that: 1) exercise by physiotherapist and standard information of post-surgical care may be superior to information alone for function and leg pain at one year (Jentoft et al., 2020); 2) standard post-surgical exercise may be superior to no intervention for function up to three months and back pain up to one year (Uysal et al., 2023); and 3) flexion exercise may be superior to conventional postoperative rehabilitation for function at the end of 14th week post-surgery (Abdi et al., 2023). Furthermore, there is low certainty evidence that supervised and graded exercise may be superior to home-based exercise for return-to-work at four months post-surgery. (Choi et al., 2005). There is conflicting evidence on the effect of walking exercise in improving function or pain when compared to no intervention (2 RCTs, low certainty evidence) (Aldemir and Gurkan, 2021; Uysal et al., 2023). Finally, there is very low certainty evidence that: 1) spinal stability exercise and usual care may be inferior to usual care alone for leg pain at one year (Paulsen et al., 2019, 2020); 2) early functional exercise may be superior to routine functional exercise for quality of life at one year (Zhang et al., 2018); 3) extension exercise may be no different to conventional postoperative rehabilitation (Abdi et al., 2023); 4) intense exercise may be no different to mild exercise for return-to-work (Manniche et al., 1993; Skall et al., 1994); and 5) strengthening and stretching may be no different to stretching alone for return-to-work (Häkkinen et al., 2005).

For non-exercise interventions, there are six RCTs with high risk of bias. These interventions were only assessed in high risk of bias RCTs (Additional file 9, Appendices). Accordingly, there is low certainty evidence that: 1) web-based information may be superior to a patient guidebook for anxiety up to three months post-surgery (Erdogan and Bulut, 2020); and 2) traditional Chinese medicine and routine nurse care may be superior to routine nurse care for pain, depression and anxiety up to two weeks post-intervention (Fu and Ji, 2017). Furthermore, this systematic review found that two-week post-surgical restriction may be no different to six-week post-surgical restriction for function (low certainty evidence), LBP (very low certainty evidence) and leg pain (very low to low certainty evidence) (Bono et al., 2017). Finally, there is very low certainty evidence that: 1) acupuncture and conventional rehabilitation may be superior to conventional rehabilitation for function up to one year post-surgery (Zhao et al., 2008); 2) systemic enzyme and routine post-surgical care may be superior to routine post-surgical care for pain and recovery up to three months and one year post-surgery, respectively (Pekař and Steindler, 2010); and 3) CAM and usual care may be superior to usual care for function up to one year post-surgery (Zheng et al., 2015).

#### 3.4.4 Adverse events

None of the 16 RCTs assessed the safety of post-surgical rehabilitation interventions.

## 4 Discussion

### 4.1 Summary of findings

Supervised Pilates exercise provided added benefits when combined with routine post-surgical care and cognitive behavioral therapy by improving function in adults with fear of movement who received surgery for LDH (Zuo et al., 2021). Furthermore, this systematic review identified one intervention with potentially short-term effects for people after undergoing rehabilitation after surgery for LDH. However, the reported benefits are small and temporary for pain reduction in the low back (not leg; and function was not assessed), and safety is unclear. Specifically, whole-body magnetic field therapy combined with exercise, pharmacological and aquatic therapy was associated with a small, short-term LBP reduction compared to exercise, pharmacological and aquatic therapy alone (Kulikov et al., 2018). We suggest that these results need to be replicated and safety needs to be assessed before consideration in clinical practice. This systematic review also found that pregabalin is inferior to placebo in reducing LBP in the short and intermediate terms suggesting that pregabalin may lead to iatrogenic effects (Zarei et al., 2016).

Moreover, this review suggests that several interventions have similar outcomes compared to their active control interventions in improving post-surgical outcomes. These interventions include: 1) behavioral graded activity vs. usual care by physiotherapists (Ostelo et al., 2003a, 2003b); 2) multimodal care (exercise and education) vs. sham or watchful waiting (Ebenbichler et al., 2015; Erdogmus et al., 2007); 3) multimodal care (exercise, education and usual post-surgical care during hospitalization) vs. usual post-surgical care during hospitalization (Oosterhuis et al., 2017); 4) remote functional exercise or remote staged exercise vs. usual post-surgical care including exercise (He et al., 2021; Lu and Bai, 2020); and 5) supervised exercise and education vs. education alone for quality of life (Beneck et al., 2014). None of the RCTs reported on adverse events.

### 4.2 Strengths and limitations

This systematic review has strengths. First, this review included comprehensive and peer-reviewed literature search strategies and examined all non-surgical rehabilitation interventions without language restrictions. Second, this review used a well-accepted WHO definition of rehabilitation, which allowed us to also capture what can be considered components of broader rehabilitation interventions (e.g., whole body magnetic therapy, pregabalin and enzyme therapy). These interventions, which may not be conceptualized as rehabilitation interventions on their own, per se, may be thought of as playing a role in the rehabilitation process (e.g., reduce pain intensity so people can participate in activity); thus, they were included in this review. Third, this review conducted a sensitivity analysis to assess the bias related to the inclusion of high risk of bias studies.

This review also has limitations. First, this review aimed to evaluate trials for patients who underwent LDH surgery for leg pain with or without associated LBP. However, 13 studies only specified participants with LDH without further information related to the presence of leg pain. Second, to compute 95% CIs in studies that did not report the correlation between pre- and post-intervention outcomes or the standard error of change, this review used a correlation coefficient of r = 0.8 that may over- or underestimate the true value (Abrams et al., 2005; Follmann et al., 1992). Third, this review was unable to assess potential publication bias using funnel plot due to heterogeneity of studies. However, this was mitigated by consulting trial registries and content experts for additional or unpublished studies. Last, the literature search strategies may have missed potentially relevant studies; however, a second health sciences librarian reviewed the search strategy, and reference lists of eligible studies and the WHO ICTRP were searched to mitigate this potential limitation.

### 4.3 Comparison with previous systematic reviews

There are five systematic reviews identified in evaluating rehabilitation interventions after lumbar surgery for LDH published between 2008 and 2023 (Oosterhuis et al., 2014; Ostelo et al., 2008; Rushton et al., 2011; Snowdon and Peiris, 2016; Manni et al., 2023). Relying on very low quality evidence, Ostelo et al. (2008) and Oosterhuis et al. (2014) reported that exercise programs beginning four to six weeks post-surgery result in a faster decrease in short-term pain and disability compared to no treatment. Furthermore, the newly published systematic review by Manni et al. (2023) concluded that: 1) supervised exercises are better than non-supervised exercises to reduce pain and disability; and 2) supervised exercises are better than advice in reducing pain and disability, both based on very low to low certainty evidence (i.e., heterogeneous and low quality RCTs) (Manni et al., 2023). Our systematic review augments their conclusions based on high certainty evidence that supervised exercise (i.e., Pilates exercise) improved function, and remote exercise was no different to education or usual post-surgical care. Regarding exercise types, the sensitivity analysis in our review supports findings of Ostelo et al. (2008), Oosterhuis et al. (2014) and Manni et al. (2023) that supervised exercise was superior to non-supervised exercise. It is important to note that these findings are related to high risk of bias studies and very low certainty evidence overall.

The results of our review differ from those found in meta-analyses by Snowdon et al. (2016)(Snowdon and Peiris, 2016) and Rushton et al. (2011) (Rushton et al., 2011). Snowdon et al. (2016) concluded that comprehensive physiotherapy (i.e., multimodal care, exercise) led to a moderate, statistically significant reduction in pain intensity compared with a control group (no treatment or exercise) (Snowdon and Peiris, 2016). The meta-analysis by Rushton et al. (2011) which pooled results from heterogeneous trials did not support the effectiveness of physiotherapy (e.g., exercise, multimodal care, psychological intervention) in the short and long term (Rushton et al., 2011).

Differences in the overall results between our review and the previous reviews may be due to several factors (Oosterhuis et al., 2014; Ostelo et al., 2008; Rushton et al., 2011; Snowdon and Peiris, 2016; Manni et al., 2023). First, we did not use broad categories of treatment such as physiotherapy because such a strategy prevents the assessment of the effectiveness and safety of specific interventions. Second, we considered statistical and clinical heterogeneity of RCTs to determine whether a meta-analysis was indicated. Third, we assessed methodological quality of eligible studies by judging the impact of potential sources of bias rather than applying a mechanical cut-off score as used in the Ostelo et al. (2008) and Oosterhuis et al. (2014) reviews. Fourth, our systematic review conducted a comprehensive literature search from inception to November 2023 without language limitations and used minimal sample size requirements (e.g., >30/arm in RCTs). Fifth, our review limited eligible rehabilitation interventions to those provided within three months post-surgery. Lastly, two studies(McGregor et al., 2011; Scrimshaw and Maher, 2001) included in Ostelo et al. (2008), Oosterhuis et al. (2014) and Manni et al. (2023) were excluded from this review because participants received fusion for lumbar stenosis.

### 4.4 Research implications

The findings in this review have important implications for research including the testing of interventions that were only evaluated in one RCT and improvement in the methodological quality of RCTs. Second, evidence is needed to understand the safety of post-surgical rehabilitation interventions. Third, when reporting trials, detailed participant information should be provided so that readers know for whom the findings may be applied. This includes the target symptoms (e.g., back and leg symptoms) before surgery, details of surgery types, time point of baseline evaluation (before surgery, after surgery or before rehabilitation intervention), and details of interventions and comparisons as outlined by the TIDieR (Template for Intervention Description and Replication) Checklist (Hoffmann et al., 2014). Fourth, RCTs should be designed so that the effects of interventions can be isolated (e.g., core strengthening exercise plus usual care vs. usual care alone). However, for most of the included RCTs in this review, interventions were compared to other active interventions such as physiotherapy care or usual care (which is a multimodal program of care, usually including some type of exercise component), without the ability to isolate intervention effects. As a result, while most of the interventions assessed showed no difference to active comparisons, it is possible that some of the specific interventions were beneficial, but the design of the studies precluded this determination.

### 4.5 Clinical implications

The findings from this systematic review advocate for individualized rehabilitation plans in managing patients after lumbar disc herniation surgery, acknowledging the varied effectiveness of interventions such as Pilates and magnetic therapy. The evidence suggests that while supervised Pilates exercises have shown benefits in improving function, the advantages of magnetic therapy, particularly when combined with other treatments, appear limited to small, short-term reductions in low back pain. This understanding challenges the one-size-fits-all approach to post-surgical rehabilitation, highlighting the need for tailored therapeutic strategies that consider the individual patient's responses and the specific nuances of each intervention.

Our review also evaluates the role of pregabalin, indicating its inferiority to placebo in managing low back pain post-surgery, which raises concerns about its potential iatrogenic effects. Furthermore, the review sheds light on the comparable outcomes of various interventions against their active controls, suggesting that while some treatments may not outperform standard care, they might still contribute valuably to personalized rehabilitation programs. This potentially underscores the necessity for ongoing research to confirm their safety and to determine their long-term effectiveness. Until such data are available, these interventions should be considered with caution, integrating them into patient care where appropriate but with a clear understanding of the existing evidence limitations.

This broader perspective underscores the importance of a flexible, patient-centered rehabilitation approach, integrating evidence-based practices with a deep understanding of individual patient needs, preferences, and the specific context of their recovery journey. Such an approach encourages the exploration of a spectrum of therapeutic options, including but not limited to, behavioral graded activities, multimodal care combining exercise and education, and various forms of exercise therapy, all tailored to enhance the patient's quality of life, functional recovery, and participation post-surgery.

Incorporating these insights, healthcare providers are urged to adopt a more adaptive, evidence-informed framework for post-surgical rehabilitation, ensuring that each patient receives a customized care plan that optimally addresses their specific rehabilitation goals, promotes their safety, and maximizes their recovery potential. This patient-centered model not only aligns with contemporary rehabilitation paradigms but also sets a foundation for future research to further refine and validate individualized rehabilitation strategies for patients recovering from lumbar disc herniation surgery.

1. Tailored Rehabilitation Approaches: Clinicians are encouraged to develop rehabilitation plans that are carefully tailored to each patient, reflecting the diverse outcomes observed in our review. While integrating intervention such as Pilates exercises, which have shown benefits in improving function, the inclusion of whole-body magnetic therapy should be approached with caution due to the limited evidence regarding its safety and longer-term effectiveness.
2. Comprehensive Patient Assessment: A thorough assessment should precede the rehabilitation planning, detailing the patient's physical, psychological and social status, the nature of their surgery, and their personal recovery aspirations. This comprehensive evaluation ensures the rehabilitation program is not only evidence-based but also finely tuned to the patient's unique circumstances.
3. Employing Patient-Centered Outcomes: The utilization of varied, meaningful outcome measures, such as the WHODAS 2.0 and the Patient-Specific Function Scale (PSFS), is important. These tools allow for a personalized assessment of the patient's functioning and progress, providing insights that are directly relevant to the patient's daily life and overall well-being.
4. Dynamic Rehabilitation Strategy: Embrace a dynamic approach to rehabilitation where the plan is regularly reviewed and adapted in response to the patient's feedback and progress. This flexibility ensures that the rehabilitation remains responsive to the patient's evolving needs and preferences throughout their recovery journey.
5. Shared Decision-Making: A collaborative decision-making process is vital, where patients are actively involved in selecting interventions that resonate with their values, goals, and preferences. This partnership ensures that the rehabilitation strategy is both patient-centered and aligned with the best available evidence.
6. Interdisciplinary Collaboration and Continuous Learning: An interdisciplinary team approach should be fostered, ensuring that various perspectives are considered in creating a comprehensive, holistic rehabilitation plan. Continuous education for both the patient and the healthcare team about the latest evidence and innovative rehabilitation strategies is important for ongoing improvement in care quality.

## 5 Conclusions

The current evidence base is insufficient to definitively support the effectiveness and safety of specific rehabilitation interventions post-surgery for lumbar disc herniation (LDH) with radiculopathy. There is a pressing need for high-quality research—studies that carry a low risk of bias and provide precise evidence—to guide clinical practice and policy-making in the rehabilitation of these patients.

To conclude, the optimal management of post-surgical rehabilitation for lumbar disc herniation should prioritize personalized care strategies, rooted in the patient's individual needs and informed by the latest evidence, including the benefits of specific interventions. By committing to these tailored care strategies and advocating for rigorous research to fill the existing evidence gaps, the healthcare community can advance rehabilitation practices that truly reflect the best interests and preferences of patients recovering from LDH surgery.

## Funding

This work was supported by 10.13039/501100011713EUROSPINE Task Force on Research and Canadian Chiropractic Research Foundation.

## Declaration of generative AI and AI-assisted technologies in the writing process

The authors did not use generative AI and AI-assisted technologies in the writing process.

## Declaration of competing interest

The authors declare the following financial interests/personal relationships which may be considered as potential competing interests.

Carol Cacelliere reports financial support was provided by 10.13039/501100011713EUROSPINE Task Force on Research. Carol Cancelliere reports financial support was provided by Canadian Chiropractic Research Foundation. Andree-Anne Marchand reports a relationship with Canadian Chiropractic Research Foundation that includes: funding grants. Andree-Anne Marchand reports a relationship with Fondation régionale pour la santé de Trois-Rivières that includes: funding grants. Dan Wang reports a relationship with Canadian Chiropractic Research Foundation that includes: funding grants. Dan Wang reports a relationship with Fondation régionale pour la santé de Trois-Rivières that includes: funding grants. Jessica Wong reports a relationship with Canadian Chiropractic Research Foundation that includes: funding grants. Jessica Wong reports a relationship with 10.13039/501100000024Canadian Institutes of Health Research (10.13039/501100000024CIHR) that includes: funding grants. James Young reports a relationship with 10.13039/501100000142Arthritis Society Canada (22–0000000058) that includes: funding grants. James Young reports a relationship with Diana Kerbel Fellowship in Arthritis Integrated Care that includes: funding grants. James Young reports a relationship with Danish Foundation for Chiropractic Research and Post-graduate Education that includes: funding grants. James Young reports a relationship with 10.13039/501100000024Canadian Institutes of Health Research Clinical Trials Fund that includes: funding grants. Margareta Nordin reports a relationship with Canadian Chiropractic Research Foundation that includes: funding grants. Margareta Nordin reports a relationship with Canadian Institute of 10.13039/100005622Health Research that includes: funding grants. Pierre Cote reports a relationship with Canada Research Chair Program that includes: funding grants. Pierre Cote reports a relationship with EUROSPINE that includes: speaking and lecture fees. Pierre Cote reports a relationship with Canadian Protective Chiropractic Association that includes: paid expert testimony. Sheilah Hogg-Johnson reports a relationship with Canadian Memorial Chiropractic College that includes: employment. Silvano Mior reports a relationship with Canadian Chiropractic Association that includes: funding grants. If there are other authors, they declare that they have no known competing financial interests or personal relationships that could have appeared to influence the work reported in this paper.
