# Supplementary material for: Effectiveness of postsurgical rehabilitation following lumbar disc herniation surgery: A systematic review
Source: Brain Spine. 2024 Apr 16;4:102806. doi: 10.1016/j.bas.2024.102806 (PMC11059472; doi:10.1016/j.bas.2024.102806)
Supplement: Multimedia component 1 [file mmc1.docx]

**APPENDICES**

**Additional file 1: Modifications of published protocol**

We modified our review protocol and updated our PROSPERO registration prior to the analyses. Specifically, we focused on lumbar disc herniation with leg pain treated by surgery (versus low back pain (LBP) with or without radiculopathy treated by surgery). We excluded studies that:

- assessed interventions used to control procedural/wound pain (e.g., administered and outcomes measured within the first week after surgery only);
- were observational studies when at least one randomized controlled trial (RCT) evaluating the same post-surgical rehabilitation intervention was available;
- reported p-values only (i.e., no data related to the effect size), and studies not providing data necessary to compute effect sizes; and
- provided incomplete details regarding the measurement of outcomes (e.g., unclear score range, unspecified purpose of numerical rating scale).

For studies using multiple measures to assess the same outcome, and at multiple time points, we selected the most common outcome measure and time point used across studies to maximize the synthesis of findings, or the longest follow-up reported in a given time period. For studies that included total outcome measurement scores and sub-scores, we prioritized and assessed total scores. When studies included more than two arms, we prioritized the comparisons beginning with sham/placebo, then control (e.g., usual care, no intervention, wait-list control), and finally another intervention. Finally, we used the Grading of Recommendations Assessment, Development and Evaluation (GRADE) approach to rate the quality of the evidence.^1^

**Additional file 2: PRISMA 2020 Checklist**

| **Section and Topic** | **Item #** | **Checklist item** | **Location where item is reported** |
| --- | --- | --- | --- |
| **TITLE** | | |  |
| Title | 1 | Identify the report as a systematic review. | p 1 |
| **ABSTRACT** | | |  |
| Abstract | 2 | See the PRISMA 2020 for Abstracts checklist. | p 5 |
| **INTRODUCTION** | | |  |
| Rationale | 3 | Describe the rationale for the review in the context of existing knowledge. | pp 7-8 |
| Objectives | 4 | Provide an explicit statement of the objective(s) or question(s) the review addresses. | p 8 |
| **METHODS** | | |  |
| Eligibility criteria | 5 | Specify the inclusion and exclusion criteria for the review and how studies were grouped for the syntheses. | p 8; table 1, Additional files 3 and 4 in Appendices |
| Information sources | 6 | Specify all databases, registers, websites, organisations, reference lists and other sources searched or consulted to identify studies. Specify the date when each source was last searched or consulted. | pp 8-9 |
| Search strategy | 7 | Present the full search strategies for all databases, registers and websites, including any filters and limits used. | Additional file 5 in Appendices |
| Selection process | 8 | Specify the methods used to decide whether a study met the inclusion criteria of the review, including how many reviewers screened each record and each report retrieved, whether they worked independently, and if applicable, details of automation tools used in the process. | p 9 |
| Data collection process | 9 | Specify the methods used to collect data from reports, including how many reviewers collected data from each report, whether they worked independently, any processes for obtaining or confirming data from study investigators, and if applicable, details of automation tools used in the process. | pp 10-11 |
| Data items | 10a | List and define all outcomes for which data were sought. Specify whether all results that were compatible with each outcome domain in each study were sought (e.g. for all measures, time points, analyses), and if not, the methods used to decide which results to collect. | pp 10-11 |
|  | 10b | List and define all other variables for which data were sought (e.g. participant and intervention characteristics, funding sources). Describe any assumptions made about any missing or unclear information. | pp 10-11 |
| Study risk of bias assessment | 11 | Specify the methods used to assess risk of bias in the included studies, including details of the tool(s) used, how many reviewers assessed each study and whether they worked independently, and if applicable, details of automation tools used in the process. | pp 9-10 |
| Effect measures | 12 | Specify for each outcome the effect measure(s) (e.g. risk ratio, mean difference) used in the synthesis or presentation of results. | pp 11-12 |
| Synthesis methods | 13a | Describe the processes used to decide which studies were eligible for each synthesis (e.g. tabulating the study intervention characteristics and comparing against the planned groups for each synthesis (item #5)). | pp 10-12 |
|  | 13b | Describe any methods required to prepare the data for presentation or synthesis, such as handling of missing summary statistics, or data conversions. | pp 11-12 |
|  | 13c | Describe any methods used to tabulate or visually display results of individual studies and syntheses. | pp 10-11 |
|  | 13d | Describe any methods used to synthesize results and provide a rationale for the choice(s). If meta-analysis was performed, describe the model(s), method(s) to identify the presence and extent of statistical heterogeneity, and software package(s) used. | pp 10-11 |
|  | 13e | Describe any methods used to explore possible causes of heterogeneity among study results (e.g. subgroup analysis, meta-regression). | pp 10-11, p14 |
|  | 13f | Describe any sensitivity analyses conducted to assess robustness of the synthesized results. | p12 |
| Reporting bias assessment | 14 | Describe any methods used to assess risk of bias due to missing results in a synthesis (arising from reporting biases). | pp 9-10 |
| Certainty assessment | 15 | Describe any methods used to assess certainty (or confidence) in the body of evidence for an outcome. | P 12, Table 2, Table 3 |
| **RESULTS** | | |  |
| Study selection | 16a | Describe the results of the search and selection process, from the number of records identified in the search to the number of studies included in the review, ideally using a flow diagram. | p 13; figure 1 |
|  | 16b | Cite studies that might appear to meet the inclusion criteria, but which were excluded, and explain why they were excluded. | Additional file 6 in Appendices |
| Study characteristics | 17 | Cite each included study and present its characteristics. | pp 13-14; Additional file 7, Appendices |
| Risk of bias in studies | 18 | Present assessments of risk of bias for each included study. | p14, Additional file 8a, 8b, Appendices |
| Results of individual studies | 19 | For all outcomes, present, for each study: (a) summary statistics for each group (where appropriate) and (b) an effect estimate and its precision (e.g. confidence/credible interval), ideally using structured tables or plots. | pp 15-19; Additional file 9, Appendices |
| Results of syntheses | 20a | For each synthesis, briefly summarise the characteristics and risk of bias among contributing studies. | pp 15-19; Additional file 9, Appendices |
|  | 20b | Present results of all statistical syntheses conducted. If meta-analysis was done, present for each the summary estimate and its precision (e.g. confidence/credible interval) and measures of statistical heterogeneity. If comparing groups, describe the direction of the effect. | pp 15-19; Additional file 9, Appendices |
|  | 20c | Present results of all investigations of possible causes of heterogeneity among study results. | pp 13-14 |
|  | 20d | Present results of all sensitivity analyses conducted to assess the robustness of the synthesized results. | pp 18-19 |
| Reporting biases | 21 | Present assessments of risk of bias due to missing results (arising from reporting biases) for each synthesis assessed. | NA |
| Certainty of evidence | 22 | Present assessments of certainty (or confidence) in the body of evidence for each outcome assessed. | pp 15-19 |
| **DISCUSSION** | | |  |
| Discussion | 23a | Provide a general interpretation of the results in the context of other evidence. | pp 19-20 |
|  | 23b | Discuss any limitations of the evidence included in the review. | pp 20-21 |
|  | 23c | Discuss any limitations of the review processes used. | pp 20-21 |
|  | 23d | Discuss implications of the results for practice, policy, and future research. | pp 22-23 |
| **OTHER INFORMATION** | | |  |
| Registration and protocol | 24a | Provide registration information for the review, including register name and registration number, or state that the review was not registered. | PROSPERO (CRD42019134607) |
|  | 24b | Indicate where the review protocol can be accessed, or state that a protocol was not prepared. | Cancelliere et al. (2020), *BMJ Open* |
|  | 24c | Describe and explain any amendments to information provided at registration or in the protocol. | Amendments to information provided at registration are described and explained below; Additional file 1 in Appendices |
|  |  | We modified our review protocol and updated our PROSPERO registration prior to the analyses. Specifically, we focused on lumbar disc herniation with leg pain treated by surgery (versus low back pain (LBP) with or without radiculopathy treated by surgery). We excluded studies that:  • assessed interventions used to control procedural/wound pain (e.g., administered and outcomes measured within the first week after surgery only);  • were observational studies when at least one randomized controlled trial (RCT) evaluating the same post-surgical rehabilitation intervention was available;  • reported p-values only (i.e., no data related to the effect size), and studies not providing data necessary to compute effect sizes; and  • provided incomplete details regarding the measurement of outcomes (e.g., unclear score range, unspecified purpose of numerical rating scale).  For studies using multiple measures to assess the same outcome, and at multiple time points, we selected the most common outcome measure and time point used across studies to maximize the synthesis of findings, or the longest follow-up reported in a given time period. For studies that included total outcome measurement scores and sub-scores, we prioritized and assessed total scores. When studies included more than two arms, we prioritized the comparisons beginning with sham/placebo, then control (e.g., usual care, no intervention, wait-list control), and finally another intervention. Finally, we used the Grading of Recommendations Assessment, Development and Evaluation (GRADE) approach to rate the quality of the evidence. | |
| Support | 25 | Describe sources of financial or non-financial support for the review, and the role of the funders or sponsors in the review. | Funding by EUROSPINE Task Force on Research, Canadian Chiropractic Research Foundation (no role in the review) |
| Competing interests | 26 | Declare any competing interests of review authors. | See declaration of interest |
| Availability of data, code and other materials | 27 | Report which of the following are publicly available and where they can be found: template data collection forms; data extracted from included studies; data used for all analyses; analytic code; any other materials used in the review. | Available from authors upon request |

**Additional file 3: Examples of selected interventions for rehabilitation**

| **Intervention** | **Definition** | **Examples** |
| --- | --- | --- |
| Patient education and self-management | Teaching patients skills that they can use to manage their health condition | - Learning disease-specific information - Learning general managing skills (e.g., problem-solving, finding and using community resources, working with healthcare team) - Learning strategies to increase confidence (i.e., self-efficacy) in ability to engage in behaviours that are needed to manage their condition on a daily basis - Adequate peer role models and support networks that facilitate the initiation and maintenance of desired behavioural changes |
| Exercise | A subcategory of physical activity that is planned, structured, repetitive, and purposeful; can be supervised (e.g., by a healthcare professional) or unsupervised | - Stretching - Strengthening - Range of motion exercises - Aerobic (e.g., swimming, cycling, walking, running) - Anaerobic (e.g., jumping, sprinting, weight lifting) |
| Manual therapies | - Manipulation: Techniques incorporating a high-velocity low-amplitude impulse or thrust applied at or near the end of a joint’s passive range of motion - Mobilization: Techniques incorporating a low-velocity and small or large amplitude oscillatory movement, within a joint’s passive range of motion - Traction: Manual or mechanically assisted application of an intermittent or continuous distractive force - Soft tissue therapy: A mechanical form of therapy where soft-tissue structures are pressed and kneaded, using physical contact with the hand or mechanical device | - Lumbar manipulation, mobilization, or traction - Massage - Muscle energy technique - Strain-counterstrain |
| Passive physical modalities | A form of cold, heat, or light application affecting the body at the skin level or ultrasonic or electromagnetic radiation affecting structures beneath the skin surface:   - Passive assistive devices: Device to encourage immobilization in anatomic positions or actively inhibit or prevent movement | - Heat application: heat pack, hydrotherapy - Cryotherapy: cold pack, vapocoolant spray - Low-level laser - Electrical muscle stimulation - Pulsed electromagnetic therapy |
| Acupuncture | Any body-needling, moxibustion, electric acupuncture, laser acupuncture, microsystem acupuncture, and acupressure | - Traditional needling - Dry needling - Burning of specific herbs - Electro-acupuncture - Photo-acupuncture |
| Pharmacological interventions | A substance used in treating disease or relieving pain | - Acetaminophen - Nonsteroidal anti-inflammatory drugs - Muscle relaxants - Antidepressants |
| Psychological interventions | Activities used to modify behaviour, emotional state, or feelings | - Cognitive behavioural therapy - Counselling - Social network and environment-based therapies - Psychoeducational interventions - Mindfulness meditation |
| Assistive technologies | Any item, piece of equipment or product system, used to increase, maintain, or improve the functional capabilities of people with disabilities | - Walking aids - Orthoses - Braces - Wheelchairs |

**Additional file 4: Definitions from the International Classification of Functioning, Disability and Health**

| **Term** | **Definition** |
| --- | --- |
| Body functions | Physiological functions of body systems (including psychological functions) |
| Body structures | Anatomical parts of the body such as organs, limbs and their components |
| Impairments | Problems in body function or structure such as a significant deviation or loss |
| Activity | Execution of a task or action by an individual |
| Participation | Involvement in a life situation |
| Activity limitations | Difficulties an individual may have in executing activities |
| Participation restrictions | Problems an individual may experience in involvement in life situations |
| Environmental factors | External contextual factors that make up the physical, social and attitudinal environment in which people live and conduct their lives |
| Personal factors | Internal contextual factors that influence how disability is experienced by the individual. |

**Additional file 5: Search strategies for electronic databases**

**CCGI_PostsurgicalRehab_2021_MEDLINE**

| 1 | exp Back Injuries/ |
| --- | --- |
| 2 | exp Back Pain/ |
| 3 | Intervertebral Disc Degeneration/ |
| 4 | Intervertebral Disc Displacement/ |
| 5 | Lumbar Vertebrae/in [Injuries] |
| 6 | Lumbosacral Region/in [Injuries] |
| 7 | Osteoarthritis, Spine/ |
| 8 | Piriformis Muscle Syndrome/ |
| 9 | Radiculopathy/ |
| 10 | Sciatica/ |
| 11 | Spinal Diseases/ |
| 12 | Spinal Stenosis/ |
| 13 | (back adj3 (ache* or injur* or pain*)).ab,ti. |
| 14 | (backache* adj3 (injur* or pain*)).ab,ti. |
| 15 | (back pain or back-pain).ab,ti. |
| 16 | (intervertebral adj3 (disease* or bulging or pain* or extru* or degenerat* or displac* or herniat* or prolaps* or sequest* or slipped* or protru* or avuls*)).ab,ti. |
| 17 | (lumbar disc* adj3 (extruded or degenerat* or herniat* or prolapse* or sequestered or slipped)).ab,ti. |
| 18 | (lumbar disk* adj3 (extruded or degenerat* or herniat* or prolapse* or sequestered or slipped)).ab,ti. |
| 19 | ((lumbarsacral* or lumbosacral) adj32 (pain* or facet* or nerve root* or osteoarthr* or radicul* or stenos* or spondylo* or zygapophys* or injur* or discomfort* or dysfunction* or sore* or herniat*)).ab,ti. |
| 20 | "low* back pain*".ab,ti. |
| 21 | (lumbar adj3 (pain or facet or nerve root* or osteoarthritis or radicul* or spinal stenosis or spondylo* or zygapophys*)).ab,ti. |
| 22 | "Piriformis syndrome*".ab,ti. |
| 23 | "radiculopath*".ab,ti. |
| 24 | ((sacral or sacroiliac) adj3 (pain* or facet* or nerve root* or osteoarthr* or radicul* or stenos* or spondylo* or zygapophys* or injur* or discomfort* or dysfunction* or sore* or herniat*)).ab,ti. |
| 25 | ((spine or spinal) adj4 (condition* or degenerat* or disable* or disabilit* or disease* or disorder* or osteoarthritis or pain or stenos?s)).ab,ti. |
| 26 | spondylosis.ab,ti. |
| 27 | or/1-26 [**back pain] |
| 28 | Arthrodesis/ |
| 29 | Back Pain/su [Surgery] |
| 30 | Decompression, Surgical/ |
| 31 | Diskectomy/ |
| 32 | Dislocations/su [Surgery] |
| 33 | Foraminotomy/ |
| 34 | Intervertebral Disc Degeneration/su [Surgery] |
| 35 | Intervertebral Disc Displacement/su [Surgery] |
| 36 | Intervertebral Disc/su [Surgery] |
| 37 | Laminectomy/ |
| 38 | Low Back Pain/su [Surgery] |
| 39 | Lumbar Fusion/ |
| 40 | Lumbar Vertebrae/su [Surgery] |
| 41 | Lumbosacral Region/su [Surgery] |
| 42 | Nerve Compression Syndromes/su [Surgery] |
| 43 | Radiculopathy/su [Surgery] |
| 44 | Sciatica/su [Surgery] |
| 45 | Spinal Diseases/su [Surgery] |
| 46 | Spinal Fusion/ |
| 47 | Spinal Stenosis/su [Surgery] |
| 48 | Spondylosis/su [Surgery] |
| 49 | Spondylolisthesis/su [Surgery] |
| 50 | Spine/su [Surgery] |
| 51 | Surgical Fixation Devices/ |
| 52 | Surgical Procedures, Minimally Invasive/ |
| 53 | arthrodesis.ab,ti. |
| 54 | "dis?ectom*".ab,ti. |
| 55 | "dis?otom*".ab,ti. |
| 56 | electroanalgesia.ab,ti. |
| 57 | (failed adj back).ab,ti. |
| 58 | "foraminotom*".ab,ti. |
| 59 | "foraminectom*".ab,ti. |
| 60 | (fusion adj2 decompress*).ab,ti. |
| 61 | ((disk or disc) adj3 (surgery or surgical)).ab,ti. |
| 62 | "laminectom*".ab,ti. |
| 63 | "laminoplast*".ab,ti. |
| 64 | "laminotom*".ab,ti. |
| 65 | (lumbar adj2 (decompression or endoscop* or fusion or surg*)).ab,ti. |
| 66 | (lumbosacral adj2 (decompression or endoscop* or fusion or surg*)).ab,ti. |
| 67 | "microdis?ectom*".ab,ti. |
| 68 | "microendoscop*".ab,ti. |
| 69 | (mini* adj3 invas* adj3 surgery).ab,ti. |
| 70 | "nucleotom*".ab,ti. |
| 71 | "postlaminectom*".ab,ti. |
| 72 | (radiculopath* adj3 surg*).ab,ti. |
| 73 | (root adj2 decompress*).ab,ti. |
| 74 | ((spine or spinal or back) adj2 (decompression or endoscop* or fusion or surg*)).ab,ti. |
| 75 | ((spondylosis or spondylolisthesis) adj2 surg*).ab,ti. |
| 76 | "surgical fixation device*".kf,tw. |
| 77 | traction.ab,ti. |
| 78 | or/28-77 [** spine surgery] |
| 79 | Acetaminophen/ |
| 80 | Acupressure/ |
| 81 | Acupuncture/ |
| 82 | exp Acupuncture Therapy/ |
| 83 | Analgesics/ |
| 84 | Antidepressive Agents/ |
| 85 | exp Anti-Inflammatory Agents, Non-Steroidal/ |
| 86 | "Bedding and Linens"/ |
| 87 | Behavior Therapy/ |
| 88 | exp Biofeedback, Psychology/ |
| 89 | Casts, Surgical/ |
| 90 | exp Cognitive Behavioral Therapy/ |
| 91 | Combined Modality Therapy/ |
| 92 | Community-Based Participatory Research/ |
| 93 | Community Health Services/ |
| 94 | Community Participation/ |
| 95 | Complementary Therapies/ |
| 96 | Cryotherapy/ |
| 97 | exp Diathermy/ |
| 98 | exp Electric Stimulation Therapy/ |
| 99 | Electroacupuncture/ |
| 100 | Ergonomics/ |
| 101 | exp Exercise/ |
| 102 | exp Exercise Movement Techniques/ |
| 103 | exp Exercise Therapy/ |
| 104 | Fluid Therapy/ |
| 105 | High-Energy Shock Waves/tu [Therapeutic Use] |
| 106 | Hospitals, Rehabilitation/ |
| 107 | Immobilization/ |
| 108 | Hot Temperature/tu [Therapeutic Use] |
| 109 | exp Hydrotherapy/ |
| 110 | Laser Therapy, Low-Level/ |
| 111 | Low-Level Light Therapy/ |
| 112 | Magnetic Field Therapy/ |
| 113 | Magnetics/tu [Therapeutic Use] |
| 114 | Massage/ |
| 115 | exp Medicine, Chinese Traditional/ |
| 116 | Muscle Relaxants, Central/ |
| 117 | exp Musculoskeletal Manipulations/ |
| 118 | exp Orthopedic Equipment/ |
| 119 | Outpatient Clinics, Hospital/ |
| 120 | Patient Education as Topic/ |
| 121 | Physical Therapy Modalities/ |
| 122 | Postoperative Period/ |
| 123 | exp Rehabilitation/ |
| 124 | Rehabilitation, Vocational/ |
| 125 | rehabilitation.fx. |
| 126 | Return to Work/ |
| 127 | Self Care/ |
| 128 | Self-Help Devices/ |
| 129 | Physical Fitness/ |
| 130 | Restraint, Physical/ |
| 131 | Transcutaneous Electric Nerve Stimulation/ |
| 132 | Vibration/tu [Therapeutic Use] |
| 133 | Wheelchairs/ |
| 134 | Work Capacity Evaluation/ |
| 135 | Work Engagement/ |
| 136 | acetaminophen.ab,ti. |
| 137 | acupressure.ab,ti. |
| 138 | "acupunctur*".ab,ti. |
| 139 | (advice or advise or advised).ab,ti. |
| 140 | alexander technique.ab,ti. |
| 141 | (anti-depressant* or antidepressant*).ab,ti. |
| 142 | "assistive device*".ab,ti. |
| 143 | "back belt*".ab,ti. |
| 144 | "back school*".ab,ti. |
| 145 | (back adj2 work).ab,ti. |
| 146 | (braces or brace or bracing).ab,ti. |
| 147 | canes.ab,ti. |
| 148 | chiropract*.ab,ti. |
| 149 | "cognitive behavioral therap*".ab,ti. |
| 150 | "cognitive behavioural therap*".ab,ti. |
| 151 | (cold adj3 (therap* or pack* or compress or massage or immersion or soak or treatment or therap*)).ab,ti. |
| 152 | Community-based rehabilitation.ab,ti. |
| 153 | Community based rehabilitation.ab,ti. |
| 154 | "core stabili*".ab,ti. |
| 155 | (corset or corsets).ab,ti. |
| 156 | crutches.ab,ti. |
| 157 | cryotherap*.ab,ti. |
| 158 | "deep tissue therap*".ab,ti. |
| 159 | diathermy.ab,ti. |
| 160 | (electric* adj3 (stimulation or EMS or heating pad*)).ab,ti. |
| 161 | electro-acupuncture.ab,ti. |
| 162 | (electrogalvanic stimulation or EGS).ab,ti. |
| 163 | (electromagnet* and (radiation or therap*)).ab,ti. |
| 164 | electromodalit*.ab,ti. |
| 165 | electrotherapy.ab,ti. |
| 166 | (exercise or exercises or exercising).ab,ti. |
| 167 | (flexion-distraction or flexion distraction).ab,ti. |
| 168 | fluidotherap*.ab,ti. |
| 169 | galvanic stimulation.ab,ti. |
| 170 | (H-Wave Device Stimulation or HWDS).ab,ti. |
| 171 | ((heat* or hot) adj3 (therap* or pack* or compress or massage or lamp or pad or bath or soak or tub or bottle or superficial or therapeutic)).ab,ti. |
| 172 | (high energy shock wave* or high-energy shock wave* or HESW).ab,ti. |
| 173 | hospital-based rehabilitation.ab,ti. |
| 174 | hospital based rehabilitation.ab,ti. |
| 175 | "hydrotherap*".ab,ti. |
| 176 | (ice adj3 (therap* or pack* or compress or massage or immersion or soak or treatment or therap*)).ab,ti. |
| 177 | "interferential current*".ab,ti. |
| 178 | infrared.ab,ti. |
| 179 | iontophoresis.ab,ti. |
| 180 | electroanalgesia.ab,ti. |
| 181 | ergonomic*.ab,ti. |
| 182 | kinesiotap*.ab,ti. |
| 183 | (laser* adj3 (phototherapy or irradiation or biostimulation or light or therap*)).ab,ti. |
| 184 | "low level laser*".ab,ti. |
| 185 | "lumbar support*".ab,ti. |
| 186 | (magnetic adj3 (necklace* or therap* or bracelet*)).ab,ti. |
| 187 | (manipulat* adj3 (therap* or treatment* or spinal or osteopath*)).ab,ti. |
| 188 | "manual therap*".ab,ti. |
| 189 | Microcurrent Electrical Neuromuscular Stimulation.ab,ti. |
| 190 | microwave*.ab,ti. |
| 191 | ((mobilisat* or mobilizat*) adj4 (osteopath* or orthopedic* or orthopaedic* or lumbar or spinal)).ab,ti. |
| 192 | "moist air bath*".ab,ti. |
| 193 | moxibustion.ab,ti. |
| 194 | ((multimodal* or multi-modal* or multi modal*) adj4 (treatment* or approach or care or therap* or procedure* or package* or manage*)).ab,ti. |
| 195 | muscle activation.ab,ti. |
| 196 | "muscle energy technique*".ab,ti. |
| 197 | (muscle adj relaxant*).ab,ti. |
| 198 | myofascial release.ab,ti. |
| 199 | (Neuromuscular Electrical Stimulation or NMES).ab,ti. |
| 200 | orthotic*.ab,ti. |
| 201 | "passive modalit*".ab,ti. |
| 202 | ((patient* or client*) adj3 (educat* or train*)).ab,ti. |
| 203 | "Percutaneous Electric* Nerve Stimulation".ab,ti. |
| 204 | (physical adj therap*).ab,ti. |
| 205 | physiotherap*.ab,ti. |
| 206 | photo-acupuncture.ab,ti. |
| 207 | pillow*.ab,ti. |
| 208 | pilates.ab,ti. |
| 209 | ((post-operative or postoperative) adj3 (care or manage* or therap*)).ab,ti. |
| 210 | (postur* adj3 (correct* or educat* or instruct* or train*)).ab,ti. |
| 211 | (pulsed adj3 (electromagnetic or magnetic or radio frequency or energy)).ab,ti. |
| 212 | radiant light.ab,ti. |
| 213 | (rehab or rehabilitat*).ab,ti. |
| 214 | (return* adj2 work).ab,ti. |
| 215 | RTW.ab,ti. |
| 216 | Russian stimulation.ab,ti. |
| 217 | "seat adj cushion*".ab,ti. |
| 218 | (self-manage* or self manage*).ab,ti. |
| 219 | (short wave* or short-wave*).ab,ti. |
| 220 | ((shockwave* or shock wave* or shock-wave*) adj3 (ultrasonic or therap* or radiation)).ab,ti. |
| 221 | "soft tissue therap*".ab,ti. |
| 222 | "spray and stretch".ab,ti. |
| 223 | strain-counterstrain.ab,ti. |
| 224 | strengthen*.ab,ti. |
| 225 | stretching.ab,ti. |
| 226 | (tape or taping).ab,ti. |
| 227 | thoracolumbosacral orthosis.ab,ti. |
| 228 | traction.ab,ti. |
| 229 | traditional Chinese medicine.ab,ti. |
| 230 | (transcutaneous electrical stimulation or TENS).ab,ti. |
| 231 | ultrasound.ab,ti. |
| 232 | vapocoolant spray.ab,ti. |
| 233 | "vibration therap*".ab,ti. |
| 234 | walkers.ab,ti. |
| 235 | "walking adj3 aid*".ab,ti. |
| 236 | "warm compress*".ab,ti. |
| 237 | whirlpool*.ab,ti. |
| 238 | yoga.ab,ti. |
| 239 | or/79-238 [**rehabilitation] |
| 240 | Case-Control Studies/ |
| 241 | Cohort Studies/ |
| 242 | Controlled Clinical Trials as Topic/ |
| 243 | Longitudinal Studies/ |
| 244 | Randomized Controlled Trials as Topic/ |
| 245 | ((case control or case-control) adj3 (stud* or design*)).ab,ti. |
| 246 | (cohort adj3 (stud* or design* or analysis)).ab,ti. |
| 247 | controlled clinical trial.pt. |
| 248 | ((followup or follow-up) adj3 (stud* or design* or analysis)).ab,ti. |
| 249 | (longitudinal* adj3 (stud* or design* or analysis)).ab,ti. |
| 250 | (prospective adj3 (stud* or design* or analysis)).ab,ti. |
| 251 | (random* and (control* or clinical or allocat*)).ab,ti. |
| 252 | randomized controlled trial.pt. |
| 253 | (retrospective adj3 (stud* or design*)).ab,ti. |
| 254 | Practice Guidelines as Topic/ |
| 255 | Guideline Adherence/ |
| 256 | practice guideline.pt. |
| 257 | guideline.pt. |
| 258 | (guideline* adj3 (clinical or consensus or practice or development or validate* or standard* or adhere*)).ab,ti. |
| 259 | Review Literature as Topic/ |
| 260 | Systematic Reviews as Topic/ |
| 261 | systematic review.pt. |
| 262 | (systematic adj3 (review* or overview*)).ab,ti. |
| 263 | or/240-262 [**study designs] |
| 264 | Attitude/ |
| 265 | Awareness/ |
| 266 | Diary as Topic/ |
| 267 | Focus Groups/ |
| 268 | Grounded Theory/ |
| 269 | Interviews as Topic/ |
| 270 | Mindfulness/ |
| 271 | Motivation/ |
| 272 | Narration/ |
| 273 | Observation/ |
| 274 | Patient Preference/ |
| 275 | Patient Satisfaction/ |
| 276 | Perception/ |
| 277 | Personal Narratives as Topic/ |
| 278 | Personal Satisfaction/ |
| 279 | Qualitative Research/ |
| 280 | Self Report/ |
| 281 | "attitude*".ab,ti. |
| 282 | (aware or awareness).ab,ti. |
| 283 | "belief*".ab,ti. |
| 284 | (diary or diaries).ab,ti. |
| 285 | "ethnograph*".ab,ti. |
| 286 | "expectation*".ab,ti. |
| 287 | "experience*".ab,ti. |
| 288 | feelings.ab,ti. |
| 289 | (focus adj2 group*).ab,ti. |
| 290 | (grounded adj theor*).ab,ti. |
| 291 | "interview*".ab,ti. |
| 292 | mindfulness.ab,ti. |
| 293 | (mixed adj method*).ab,ti. |
| 294 | "motivat*".ab,ti. |
| 295 | (narration* or narrative*).ab,ti. |
| 296 | (observe* or observation*).ab,ti. |
| 297 | "opinion*".ab,ti. |
| 298 | (patient* adj2 journey).ab,ti. |
| 299 | (perceiv* or perception*).ab,ti. |
| 300 | "perspective*".ab,ti. |
| 301 | "preference*".ab,ti. |
| 302 | qualitative.ab,ti. |
| 303 | satisfaction.ab,ti. |
| 304 | (self adj3 report*).ab,ti. |
| 305 | or/264-303 [**qualitative/experience] |
| 306 | 27 and 78 and 239 and 263 [**effectiveness] |
| 307 | 27 and 78 and 239 and 305 [**qualitative] |
| 308 | 306 or 307 |
| 309 | limit 308 to ed=20191001-20210801 |

**CCGI_PostsurgicalRehab_2021_Embase**

| 1 | backache/ |
| --- | --- |
| 2 | low back pain/ |
| 3 | intervertebral disc degeneration/ |
| 4 | intervertebral disk hernia/ |
| 5 | lumbar vertebra/ |
| 6 | lumbosacral region/ |
| 7 | piriformis syndrome/ |
| 8 | radiculopathy/ |
| 9 | sciatica/ |
| 10 | spine disease/ |
| 11 | vertebral canal stenosis/ |
| 12 | spondylosis/ |
| 13 | (back adj3 (ache* or injur* or pain*)).ab,ti. |
| 14 | (backache* adj3 (injur* or pain*)).ab,ti. |
| 15 | (back pain or back-pain).ab,ti. |
| 16 | (intervertebral adj3 (disease* or bulging or pain* or extru* or degenerat* or displac* or herniat* or prolaps* or sequest* or slipped* or protru* or avuls*)).ab,ti. |
| 17 | (lumbar disc* adj3 (extruded or degenerat* or herniat* or prolapse* or sequestered or slipped)).ab,ti. |
| 18 | (lumbar disk* adj3 (extruded or degenerat* or herniat* or prolapse* or sequestered or slipped)).ab,ti. |
| 19 | ((lumbarsacral* or lumbosacral) adj32 (pain* or facet* or nerve root* or osteoarthr* or radicul* or stenos* or spondylo* or zygapophys* or injur* or discomfort* or dysfunction* or sore* or herniat*)).ab,ti. |
| 20 | "low* back pain*".ab,ti. |
| 21 | (lumbar adj3 (pain or facet or nerve root* or osteoarthritis or radicul* or spinal stenosis or spondylo* or zygapophys*)).ab,ti. |
| 22 | "Piriformis syndrome*".ab,ti. |
| 23 | "radiculopath*".ab,ti. |
| 24 | ((sacral or sacroiliac) adj3 (pain* or facet* or nerve root* or osteoarthr* or radicul* or stenos* or spondylo* or zygapophys* or injur* or discomfort* or dysfunction* or sore* or herniat*)).ab,ti. |
| 25 | ((spine or spinal) adj4 (condition* or degenerat* or disable* or disabilit* or disease* or disorder* or osteoarthritis or pain or stenos?s)).ab,ti. |
| 26 | spondylosis.ab,ti. |
| 27 | or/1-26 [**back pain] |
| 28 | arthrodesis/ |
| 29 | decompression surgery/ |
| 30 | discectomy/ |
| 31 | dislocation/ |
| 32 | foraminotomy/ |
| 33 | exp spine surgery/ |
| 34 | orthopedic fixation device/ |
| 35 | Surgical Procedures, Minimally Invasive/ |
| 36 | arthrodesis.ab,ti. |
| 37 | "dis?ectom*".ab,ti. |
| 38 | "dis?otom*".ab,ti. |
| 39 | electroanalgesia.ab,ti. |
| 40 | (failed adj back).ab,ti. |
| 41 | "foraminotom*".ab,ti. |
| 42 | "foraminectom*".ab,ti. |
| 43 | (fusion adj2 decompress*).ab,ti. |
| 44 | ((disk or disc) adj3 (surgery or surgical)).ab,ti. |
| 45 | "laminectom*".ab,ti. |
| 46 | "laminoplast*".ab,ti. |
| 47 | "laminotom*".ab,ti. |
| 48 | (lumbar adj2 (decompression or endoscop* or fusion or surg*)).ab,ti. |
| 49 | (lumbosacral adj2 (decompression or endoscop* or fusion or surg*)).ab,ti. |
| 50 | "microdis?ectom*".ab,ti. |
| 51 | "microendoscop*".ab,ti. |
| 52 | (mini* adj3 invas* adj3 surgery).ab,ti. |
| 53 | "nucleotom*".ab,ti. |
| 54 | "postlaminectom*".ab,ti. |
| 55 | (radiculopath* adj3 surg*).ab,ti. |
| 56 | (root adj2 decompress*).ab,ti. |
| 57 | ((spine or spinal or back) adj2 (decompression or endoscop* or fusion or surg*)).ab,ti. |
| 58 | ((spondylosis or spondylolisthesis) adj2 surg*).ab,ti. |
| 59 | ["surgical fixation device*".kf,tw.] |
| 60 | traction.ab,ti. |
| 61 | or/28-60 [**spinal surgery] |
| 62 | paracetamol/ |
| 63 | acupressure/ |
| 64 | acupuncture/ |
| 65 | analgesic agent/ |
| 66 | antidepressant agent/ |
| 67 | nonsteroid antiinflammatory agent/ |
| 68 | behavior therapy/ |
| 69 | biofeedback/ |
| 70 | plaster cast/ |
| 71 | cognitive behavioral therapy/ |
| 72 | participatory research/ |
| 73 | community care/ |
| 74 | community participation/ |
| 75 | alternative medicine/ |
| 76 | cryotherapy/ |
| 77 | diathermy/ |
| 78 | electrostimulation therapy/ |
| 79 | electroacupuncture/ |
| 80 | ergonomics/ |
| 81 | exp exercise/ |
| 82 | exp kinesiotherapy/ |
| 83 | fluid therapy/ |
| 84 | shock wave/ |
| 85 | rehabilitation center/ |
| 86 | Immobilization/ |
| 87 | heat/ |
| 88 | exp Hydrotherapy/ |
| 89 | low level laser therapy/ |
| 90 | phototherapy/ |
| 91 | magnetotherapy/ |
| 92 | exp magnetism/ |
| 93 | Massage/ |
| 94 | Chinese medicine/ |
| 95 | muscle relaxant agent/ |
| 96 | manipulative medicine/ |
| 97 | exp orthosis/ |
| 98 | outpatient department/ |
| 99 | patient education/ |
| 100 | physiotherapy/ |
| 101 | postoperative period/ |
| 102 | rehabilitation/ |
| 103 | vocational rehabilitation/ |
| 104 | rehabilitation.fs. |
| 105 | return to work/ |
| 106 | self care/ |
| 107 | brace/ or splint/ |
| 108 | fitness/ |
| 109 | transcutaneous nerve stimulation/ |
| 110 | whole body vibration/ |
| 111 | wheelchair/ |
| 112 | work capacity/ |
| 113 | work engagement/ |
| 114 | acetaminophen.ab,ti. |
| 115 | acupressure.ab,ti. |
| 116 | "acupunctur*".ab,ti. |
| 117 | (advice or advise or advised).ab,ti. |
| 118 | alexander technique.ab,ti. |
| 119 | (anti-depressant* or antidepressant*).ab,ti. |
| 120 | "assistive device*".ab,ti. |
| 121 | "back belt*".ab,ti. |
| 122 | "back school*".ab,ti. |
| 123 | (back adj2 work).ab,ti. |
| 124 | (braces or brace or bracing).ab,ti. |
| 125 | canes.ab,ti. |
| 126 | chiropract*.ab,ti. |
| 127 | "cognitive behavioral therap*".ab,ti. |
| 128 | "cognitive behavioural therap*".ab,ti. |
| 129 | (cold adj3 (therap* or pack* or compress or massage or immersion or soak or treatment or therap*)).ab,ti. |
| 130 | Community-based rehabilitation.ab,ti. |
| 131 | Community based rehabilitation.ab,ti. |
| 132 | "core stabili*".ab,ti. |
| 133 | (corset or corsets).ab,ti. |
| 134 | crutches.ab,ti. |
| 135 | cryotherap*.ab,ti. |
| 136 | "deep tissue therap*".ab,ti. |
| 137 | diathermy.ab,ti. |
| 138 | (electric* adj3 (stimulation or EMS or heating pad*)).ab,ti. |
| 139 | electro-acupuncture.ab,ti. |
| 140 | (electrogalvanic stimulation or EGS).ab,ti. |
| 141 | (electromagnet* and (radiation or therap*)).ab,ti. |
| 142 | electromodalit*.ab,ti. |
| 143 | electrotherapy.ab,ti. |
| 144 | (exercise or exercises or exercising).ab,ti. |
| 145 | (flexion-distraction or flexion distraction).ab,ti. |
| 146 | fluidotherap*.ab,ti. |
| 147 | galvanic stimulation.ab,ti. |
| 148 | (H-Wave Device Stimulation or HWDS).ab,ti. |
| 149 | ((heat* or hot) adj3 (therap* or pack* or compress or massage or lamp or pad or bath or soak or tub or bottle or superficial or therapeutic)).ab,ti. |
| 150 | (high energy shock wave* or high-energy shock wave* or HESW).ab,ti. |
| 151 | hospital-based rehabilitation.ab,ti. |
| 152 | hospital based rehabilitation.ab,ti. |
| 153 | "hydrotherap*".ab,ti. |
| 154 | (ice adj3 (therap* or pack* or compress or massage or immersion or soak or treatment or therap*)).ab,ti. |
| 155 | "interferential current*".ab,ti. |
| 156 | infrared.ab,ti. |
| 157 | iontophoresis.ab,ti. |
| 158 | electroanalgesia.ab,ti. |
| 159 | ergonomic*.ab,ti. |
| 160 | kinesiotap*.ab,ti. |
| 161 | (laser* adj3 (phototherapy or irradiation or biostimulation or light or therap*)).ab,ti. |
| 162 | "low level laser*".ab,ti. |
| 163 | "lumbar support*".ab,ti. |
| 164 | (magnetic adj3 (necklace* or therap* or bracelet*)).ab,ti. |
| 165 | (manipulat* adj3 (therap* or treatment* or spinal or osteopath*)).ab,ti. |
| 166 | "manual therap*".ab,ti. |
| 167 | Microcurrent Electrical Neuromuscular Stimulation.ab,ti. |
| 168 | microwave*.ab,ti. |
| 169 | ((mobilisat* or mobilizat*) adj4 (osteopath* or orthopedic* or orthopaedic* or lumbar or spinal)).ab,ti. |
| 170 | "moist air bath*".ab,ti. |
| 171 | moxibustion.ab,ti. |
| 172 | ((multimodal* or multi-modal* or multi modal*) adj4 (treatment* or approach or care or therap* or procedure* or package* or manage*)).ab,ti. |
| 173 | muscle activation.ab,ti. |
| 174 | "muscle energy technique*".ab,ti. |
| 175 | (muscle adj relaxant*).ab,ti. |
| 176 | myofascial release.ab,ti. |
| 177 | (Neuromuscular Electrical Stimulation or NMES).ab,ti. |
| 178 | orthotic*.ab,ti. |
| 179 | "passive modalit*".ab,ti. |
| 180 | ((patient* or client*) adj3 (educat* or train*)).ab,ti. |
| 181 | "Percutaneous Electric* Nerve Stimulation".ab,ti. |
| 182 | (physical adj therap*).ab,ti. |
| 183 | physiotherap*.ab,ti. |
| 184 | photo-acupuncture.ab,ti. |
| 185 | pillow*.ab,ti. |
| 186 | pilates.ab,ti. |
| 187 | ((post-operative or postoperative) adj3 (care or manage* or therap*)).ab,ti. |
| 188 | (postur* adj3 (correct* or educat* or instruct* or train*)).ab,ti. |
| 189 | (pulsed adj3 (electromagnetic or magnetic or radio frequency or energy)).ab,ti. |
| 190 | radiant light.ab,ti. |
| 191 | (rehab or rehabilitat*).ab,ti. |
| 192 | (return* adj2 work).ab,ti. |
| 193 | RTW.ab,ti. |
| 194 | Russian stimulation.ab,ti. |
| 195 | "seat adj cushion*".ab,ti. |
| 196 | (self-manage* or self manage*).ab,ti. |
| 197 | (short wave* or short-wave*).ab,ti. |
| 198 | ((shockwave* or shock wave* or shock-wave*) adj3 (ultrasonic or therap* or radiation)).ab,ti. |
| 199 | "soft tissue therap*".ab,ti. |
| 200 | "spray and stretch".ab,ti. |
| 201 | strain-counterstrain.ab,ti. |
| 202 | strengthen*.ab,ti. |
| 203 | stretching.ab,ti. |
| 204 | (tape or taping).ab,ti. |
| 205 | thoracolumbosacral orthosis.ab,ti. |
| 206 | traction.ab,ti. |
| 207 | traditional Chinese medicine.ab,ti. |
| 208 | (transcutaneous electrical stimulation or TENS).ab,ti. |
| 209 | ultrasound.ab,ti. |
| 210 | vapocoolant spray.ab,ti. |
| 211 | "vibration therap*".ab,ti. |
| 212 | walkers.ab,ti. |
| 213 | "walking adj3 aid*".ab,ti. |
| 214 | "warm compress*".ab,ti. |
| 215 | whirlpool*.ab,ti. |
| 216 | yoga.ab,ti. |
| 217 | or/62-216 [**rehabilitation] |
| 218 | case control study/ |
| 219 | cohort analysis/ |
| 220 | "controlled clinical trial (topic)"/ |
| 221 | longitudinal study/ |
| 222 | "randomized controlled trial (topic)"/ |
| 223 | ((case control or case-control) adj3 (stud* or design*)).ab,ti. |
| 224 | (cohort adj3 (stud* or design* or analysis)).ab,ti. |
| 225 | ((followup or follow-up) adj3 (stud* or design* or analysis)).ab,ti. |
| 226 | (longitudinal* adj3 (stud* or design* or analysis)).ab,ti. |
| 227 | (prospective adj3 (stud* or design* or analysis)).ab,ti. |
| 228 | (random* and (control* or clinical or allocat*)).ab,ti. |
| 229 | (retrospective adj3 (stud* or design*)).ab,ti. |
| 230 | practice guideline/ |
| 231 | protocol compliance/ |
| 232 | (guideline* adj3 (clinical or consensus or practice or development or validate* or standard* or adhere*)).ab,ti. |
| 233 | "systematic review (topic)"/ |
| 234 | (systematic adj3 (review* or overview*)).ab,ti. |
| 235 | or/218-234 [**study designs] |
| 236 | attitude to health/ |
| 237 | patient attitude/ |
| 238 | Awareness/ |
| 239 | information processing/ |
| 240 | grounded theory/ |
| 241 | interview/ |
| 242 | Mindfulness/ |
| 243 | Motivation/ |
| 244 | verbal communication/ |
| 245 | Observation/ |
| 246 | participant observation/ |
| 247 | Patient Preference/ |
| 248 | Patient Satisfaction/ |
| 249 | Perception/ |
| 250 | satisfaction/ |
| 251 | Qualitative Research/ |
| 252 | Self Report/ |
| 253 | "attitude*".ab,ti. |
| 254 | (aware or awareness).ab,ti. |
| 255 | "belief*".ab,ti. |
| 256 | (diary or diaries).ab,ti. |
| 257 | "ethnograph*".ab,ti. |
| 258 | "expectation*".ab,ti. |
| 259 | "experience*".ab,ti. |
| 260 | feelings.ab,ti. |
| 261 | (focus adj2 group*).ab,ti. |
| 262 | (grounded adj theor*).ab,ti. |
| 263 | "interview*".ab,ti. |
| 264 | mindfulness.ab,ti. |
| 265 | (mixed adj method*).ab,ti. |
| 266 | "motivat*".ab,ti. |
| 267 | (narration* or narrative*).ab,ti. |
| 268 | (observe* or observation*).ab,ti. |
| 269 | "opinion*".ab,ti. |
| 270 | (patient* adj2 journey).ab,ti. |
| 271 | (perceiv* or perception*).ab,ti. |
| 272 | "perspective*".ab,ti. |
| 273 | "preference*".ab,ti. |
| 274 | qualitative.ab,ti. |
| 275 | satisfaction.ab,ti. |
| 276 | (self adj3 report*).ab,ti. |
| 277 | or/236-276 [**qualitative] |
| 278 | 27 and 61 and 217 and 235 |
| 279 | 27 and 61 and 217 and 277 |
| 280 | 278 or 279 |
| 281 | limit 280 to (editorial or letter) |
| 282 | 280 not 281 |
| 283 | limit 282 to dd=20191001-20210801 |

**CCGI_PostsurgicalRehab_2021_PsycINFO**

| 1 | exp Back Pain/ |
| --- | --- |
| 2 | Lumbar Spinal Cord/ |
| 3 | Spinal Cord Injuries/ |
| 4 | Spinal Column/ |
| 5 | (back adj3 (ache* or injur* or pain*)).ab,ti. |
| 6 | (backache* adj3 (injur* or pain*)).ab,ti. |
| 7 | (back pain or back-pain).ab,ti. |
| 8 | (intervertebral adj3 (disease* or bulging or pain* or extru* or degenerat* or displac* or herniat* or prolaps* or sequest* or slipped* or protru* or avuls*)).ab,ti. |
| 9 | (lumbar disc* adj3 (extruded or degenerat* or herniat* or prolapse* or sequestered or slipped)).ab,ti. |
| 10 | (lumbar disk* adj3 (extruded or degenerat* or herniat* or prolapse* or sequestered or slipped)).ab,ti. |
| 11 | ((lumbarsacral* or lumbosacral) adj32 (pain* or facet* or nerve root* or osteoarthr* or radicul* or stenos* or spondylo* or zygapophys* or injur* or discomfort* or dysfunction* or sore* or herniat*)).ab,ti. |
| 12 | "low* back pain*".ab,ti. |
| 13 | (lumbar adj3 (pain or facet or nerve root* or osteoarthritis or radicul* or spinal stenosis or spondylo* or zygapophys*)).ab,ti. |
| 14 | "Piriformis syndrome*".ab,ti. |
| 15 | "radiculopath*".ab,ti. |
| 16 | ((sacral or sacroiliac) adj3 (pain* or facet* or nerve root* or osteoarthr* or radicul* or stenos* or spondylo* or zygapophys* or injur* or discomfort* or dysfunction* or sore* or herniat*)).ab,ti. |
| 17 | ((spine or spinal) adj4 (condition* or degenerat* or disable* or disabilit* or disease* or disorder* or osteoarthritis or pain or stenos?s)).ab,ti. |
| 18 | spondylosis.ab,ti. |
| 19 | or/1-18 [**back pain] |
| 20 | arthrodesis.ab,ti. |
| 21 | "dis?ectom*".ab,ti. |
| 22 | "dis?otom*".ab,ti. |
| 23 | electroanalgesia.ab,ti. |
| 24 | (failed adj back).ab,ti. |
| 25 | "foraminotom*".ab,ti. |
| 26 | "foraminectom*".ab,ti. |
| 27 | (fusion adj2 decompress*).ab,ti. |
| 28 | ((disk or disc) adj3 (surgery or surgical)).ab,ti. |
| 29 | "laminectom*".ab,ti. |
| 30 | "laminoplast*".ab,ti. |
| 31 | "laminotom*".ab,ti. |
| 32 | (lumbar adj2 (decompression or endoscop* or fusion or surg*)).ab,ti. |
| 33 | (lumbosacral adj2 (decompression or endoscop* or fusion or surg*)).ab,ti. |
| 34 | "microdis?ectom*".ab,ti. |
| 35 | "microendoscop*".ab,ti. |
| 36 | (mini* adj3 invas* adj3 surgery).ab,ti. |
| 37 | "nucleotom*".ab,ti. |
| 38 | "postlaminectom*".ab,ti. |
| 39 | (radiculopath* adj3 surg*).ab,ti. |
| 40 | (root adj2 decompress*).ab,ti. |
| 41 | ((spine or spinal or back) adj2 (decompression or endoscop* or fusion or surg*)).ab,ti. |
| 42 | ((spondylosis or spondylolisthesis) adj2 surg*).ab,ti. |
| 43 | ["surgical fixation device*".kf,tw.] |
| 44 | traction.ab,ti. |
| 45 | or/20-44 [**back surgery] |
| 46 | exp Rehabilitation/ |
| 47 | exp Analgesic Drugs/ |
| 48 | Acupuncture/ |
| 49 | Antidepressant Drugs/ |
| 50 | Anti Inflammatory Drugs/ |
| 51 | exp Behavior Therapy/ |
| 52 | exp Biofeedback/ |
| 53 | exp Cognitive Behavior Therapy/ |
| 54 | Alternative Medicine/ |
| 55 | Electrical Stimulation/ |
| 56 | Human Factors Engineering/ |
| 57 | exp Exercise/ |
| 58 | Movement Therapy/ |
| 59 | Shock therapy/ |
| 60 | Heat/ |
| 61 | exp Hydrotherapy/ |
| 62 | Laser Irradiation/ |
| 63 | exp Magnetism/ |
| 64 | Massage/ |
| 65 | Client Education/ |
| 66 | Physical Therapy/ |
| 67 | exp Rehabilitation/ |
| 68 | Rehabilitation Centers/ |
| 69 | rehabilitation.cc. |
| 70 | Reemployment/ |
| 71 | Self-Care Skills/ |
| 72 | Self-Help Techniques/ |
| 73 | Physical Fitness/ |
| 74 | Physical Restraint/ |
| 75 | Vibration/ |
| 76 | Mobility Aids/ |
| 77 | Work-Life Balance/ |
| 78 | acetaminophen.ab,ti. |
| 79 | acupressure.ab,ti. |
| 80 | "acupunctur*".ab,ti. |
| 81 | (advice or advise or advised).ab,ti. |
| 82 | alexander technique.ab,ti. |
| 83 | (anti-depressant* or antidepressant*).ab,ti. |
| 84 | "assistive device*".ab,ti. |
| 85 | "back belt*".ab,ti. |
| 86 | "back school*".ab,ti. |
| 87 | (back adj2 work).ab,ti. |
| 88 | (braces or brace or bracing).ab,ti. |
| 89 | canes.ab,ti. |
| 90 | chiropract*.ab,ti. |
| 91 | "cognitive behavioral therap*".ab,ti. |
| 92 | "cognitive behavioural therap*".ab,ti. |
| 93 | (cold adj3 (therap* or pack* or compress or massage or immersion or soak or treatment or therap*)).ab,ti. |
| 94 | Community-based rehabilitation.ab,ti. |
| 95 | Community based rehabilitation.ab,ti. |
| 96 | "core stabili*".ab,ti. |
| 97 | (corset or corsets).ab,ti. |
| 98 | crutches.ab,ti. |
| 99 | cryotherap*.ab,ti. |
| 100 | "deep tissue therap*".ab,ti. |
| 101 | diathermy.ab,ti. |
| 102 | (electric* adj3 (stimulation or EMS or heating pad*)).ab,ti. |
| 103 | electro-acupuncture.ab,ti. |
| 104 | (electrogalvanic stimulation or EGS).ab,ti. |
| 105 | (electromagnet* and (radiation or therap*)).ab,ti. |
| 106 | electromodalit*.ab,ti. |
| 107 | electrotherapy.ab,ti. |
| 108 | (exercise or exercises or exercising).ab,ti. |
| 109 | (flexion-distraction or flexion distraction).ab,ti. |
| 110 | fluidotherap*.ab,ti. |
| 111 | galvanic stimulation.ab,ti. |
| 112 | (H-Wave Device Stimulation or HWDS).ab,ti. |
| 113 | ((heat* or hot) adj3 (therap* or pack* or compress or massage or lamp or pad or bath or soak or tub or bottle or superficial or therapeutic)).ab,ti. |
| 114 | (high energy shock wave* or high-energy shock wave* or HESW).ab,ti. |
| 115 | hospital-based rehabilitation.ab,ti. |
| 116 | hospital based rehabilitation.ab,ti. |
| 117 | "hydrotherap*".ab,ti. |
| 118 | (ice adj3 (therap* or pack* or compress or massage or immersion or soak or treatment or therap*)).ab,ti. |
| 119 | "interferential current*".ab,ti. |
| 120 | infrared.ab,ti. |
| 121 | iontophoresis.ab,ti. |
| 122 | electroanalgesia.ab,ti. |
| 123 | ergonomic*.ab,ti. |
| 124 | kinesiotap*.ab,ti. |
| 125 | (laser* adj3 (phototherapy or irradiation or biostimulation or light or therap*)).ab,ti. |
| 126 | "low level laser*".ab,ti. |
| 127 | "lumbar support*".ab,ti. |
| 128 | (magnetic adj3 (necklace* or therap* or bracelet*)).ab,ti. |
| 129 | (manipulat* adj3 (therap* or treatment* or spinal or osteopath*)).ab,ti. |
| 130 | "manual therap*".ab,ti. |
| 131 | Microcurrent Electrical Neuromuscular Stimulation.ab,ti. |
| 132 | microwave*.ab,ti. |
| 133 | ((mobilisat* or mobilizat*) adj4 (osteopath* or orthopedic* or orthopaedic* or lumbar or spinal)).ab,ti. |
| 134 | "moist air bath*".ab,ti. |
| 135 | moxibustion.ab,ti. |
| 136 | ((multimodal* or multi-modal* or multi modal*) adj4 (treatment* or approach or care or therap* or procedure* or package* or manage*)).ab,ti. |
| 137 | muscle activation.ab,ti. |
| 138 | "muscle energy technique*".ab,ti. |
| 139 | (muscle adj relaxant*).ab,ti. |
| 140 | myofascial release.ab,ti. |
| 141 | (Neuromuscular Electrical Stimulation or NMES).ab,ti. |
| 142 | orthotic*.ab,ti. |
| 143 | "passive modalit*".ab,ti. |
| 144 | ((patient* or client*) adj3 (educat* or train*)).ab,ti. |
| 145 | "Percutaneous Electric* Nerve Stimulation".ab,ti. |
| 146 | (physical adj therap*).ab,ti. |
| 147 | physiotherap*.ab,ti. |
| 148 | photo-acupuncture.ab,ti. |
| 149 | pillow*.ab,ti. |
| 150 | pilates.ab,ti. |
| 151 | ((post-operative or postoperative) adj3 (care or manage* or therap*)).ab,ti. |
| 152 | (postur* adj3 (correct* or educat* or instruct* or train*)).ab,ti. |
| 153 | (pulsed adj3 (electromagnetic or magnetic or radio frequency or energy)).ab,ti. |
| 154 | radiant light.ab,ti. |
| 155 | (rehab or rehabilitat*).ab,ti. |
| 156 | (return* adj2 work).ab,ti. |
| 157 | RTW.ab,ti. |
| 158 | Russian stimulation.ab,ti. |
| 159 | "seat adj cushion*".ab,ti. |
| 160 | (self-manage* or self manage*).ab,ti. |
| 161 | (short wave* or short-wave*).ab,ti. |
| 162 | ((shockwave* or shock wave* or shock-wave*) adj3 (ultrasonic or therap* or radiation)).ab,ti. |
| 163 | "soft tissue therap*".ab,ti. |
| 164 | "spray and stretch".ab,ti. |
| 165 | strain-counterstrain.ab,ti. |
| 166 | strengthen*.ab,ti. |
| 167 | stretching.ab,ti. |
| 168 | (tape or taping).ab,ti. |
| 169 | thoracolumbosacral orthosis.ab,ti. |
| 170 | traction.ab,ti. |
| 171 | traditional Chinese medicine.ab,ti. |
| 172 | (transcutaneous electrical stimulation or TENS).ab,ti. |
| 173 | ultrasound.ab,ti. |
| 174 | vapocoolant spray.ab,ti. |
| 175 | "vibration therap*".ab,ti. |
| 176 | walkers.ab,ti. |
| 177 | "walking adj3 aid*".ab,ti. |
| 178 | "warm compress*".ab,ti. |
| 179 | whirlpool*.ab,ti. |
| 180 | yoga.ab,ti. |
| 181 | or/46-180 [**rehabilitation] |
| 182 | Cohort Analysis/ |
| 183 | Clinical Trials/ |
| 184 | Longitudinal Studies/ |
| 185 | exp Randomized Controlled Trials/ |
| 186 | Treatment Guidelines/ |
| 187 | Best Practices/ |
| 188 | "Literature Review"/ |
| 189 | "Systematic Review"/ |
| 190 | ((case control or case-control) adj3 (stud* or design*)).ab,ti. |
| 191 | (cohort adj3 (stud* or design* or analysis)).ab,ti. |
| 192 | controlled clinical trial.pt. |
| 193 | ((followup or follow-up) adj3 (stud* or design* or analysis)).ab,ti. |
| 194 | (longitudinal* adj3 (stud* or design* or analysis)).ab,ti. |
| 195 | (prospective adj3 (stud* or design* or analysis)).ab,ti. |
| 196 | (random* and (control* or clinical or allocat*)).ab,ti. |
| 197 | (retrospective adj3 (stud* or design*)).ab,ti. |
| 198 | Practice Guidelines as Topic/ |
| 199 | Guideline Adherence/ |
| 200 | (guideline* adj3 (clinical or consensus or practice or development or validate* or standard* or adhere*)).ab,ti. |
| 201 | (systematic adj3 (review* or overview*)).ab,ti. |
| 202 | or/182-201 [**study designs] |
| 203 | exp Attitudes/ |
| 204 | Awareness/ |
| 205 | Journal Writing/ |
| 206 | Focus Group/ |
| 207 | Grounded Theory/ |
| 208 | Interviews/ |
| 209 | Mindfulness/ |
| 210 | Mindfulness-Based Interventions/ |
| 211 | Motivation/ |
| 212 | Narratives/ |
| 213 | exp Observation Methods/ |
| 214 | Preferences/ |
| 215 | Perception/ |
| 216 | Narratives/ |
| 217 | Satisfaction/ |
| 218 | Qualitative Methods/ |
| 219 | Self-Report/ |
| 220 | "attitude*".ab,ti. |
| 221 | (aware or awareness).ab,ti. |
| 222 | "belief*".ab,ti. |
| 223 | (diary or diaries).ab,ti. |
| 224 | "ethnograph*".ab,ti. |
| 225 | "expectation*".ab,ti. |
| 226 | "experience*".ab,ti. |
| 227 | feelings.ab,ti. |
| 228 | (focus adj2 group*).ab,ti. |
| 229 | (grounded adj theor*).ab,ti. |
| 230 | "interview*".ab,ti. |
| 231 | mindfulness.ab,ti. |
| 232 | (mixed adj method*).ab,ti. |
| 233 | "motivat*".ab,ti. |
| 234 | (narration* or narrative*).ab,ti. |
| 235 | (observe* or observation*).ab,ti. |
| 236 | "opinion*".ab,ti. |
| 237 | (patient* adj2 journey).ab,ti. |
| 238 | (perceiv* or perception*).ab,ti. |
| 239 | "perspective*".ab,ti. |
| 240 | "preference*".ab,ti. |
| 241 | qualitative.ab,ti. |
| 242 | satisfaction.ab,ti. |
| 243 | (self adj3 report*).ab,ti. |
| 244 | or/203-243 [** experience] |
| 245 | 19 and 45 and 181 and 202 |
| 246 | 19 and 45 and 181 and 244 |
| 247 | 245 or 246 |
| 248 | limit 247 to up=20191001-20210801 |

**CCGI_PostSurgicalRehab_2021_CINAHL**

| **#** | **Query** |
| --- | --- |
| S267 | S263 or S265 Limiters -Published Date: 20191001-20210831; Peer Reviewed; Exclude MEDLINE records Expanders - Apply equivalent subjects Search modes - Boolean/Phrase |
| S266 | S263 or S265 |
| S265 | S202 and S264 |
| S264 | S203 OR S204 OR S205 OR S206 OR S207 OR S208 OR S209 OR S210 OR S211 OR S212 OR S213 OR S214 OR S215 OR S216 OR S217 OR S218 OR S219 |
| S263 | S202 AND S261 |
| S262 | S202 AND S221 |
| S261 | S222 OR S223 OR S224 OR S225 OR S226 OR S227 OR S228 OR S229 OR S230 OR S231 OR S232 OR S233 OR S234 OR S235 OR S236 OR S237 OR S238 OR S239 OR S240 OR S241 OR S242 OR S243 OR S244 OR S245 OR S246 OR S247 OR S248 OR S249 OR S250 OR S251 OR S252 OR S253 OR S254 OR S255 OR S256 OR S257 OR S258 OR S259 OR S260 |
| S260 | self N3 report* |
| S259 | satisfaction |
| S258 | qualitative |
| S257 | preference* |
| S256 | perspective* |
| S255 | perceiv* or perception* |
| S254 | patient* N2 journey* |
| S253 | opinion* |
| S252 | observe or observation* |
| S251 | narration* or narrative* |
| S250 | motivat* |
| S249 | mindfulness |
| S248 | interview* |
| S247 | grounded theor* |
| S246 | focus N2 group* |
| S245 | feelings |
| S244 | experience* |
| S243 | expectation* |
| S242 | ethnograph* |
| S241 | diary or diaries |
| S240 | belief* |
| S239 | aware or awareness |
| S238 | attitude* |
| S237 | MH Self Report |
| S236 | MH Qualitative Studies |
| S235 | MH Personal Satisfaction |
| S234 | MH Narratives |
| S233 | MH Perception |
| S232 | MH Patient Satisfaction |
| S231 | MH Patient Preference |
| S230 | MH Narratives |
| S229 | MH Motivation |
| S228 | MH Mindfulness |
| S227 | MH Interviews |
| S226 | MH Grounded Theory |
| S225 | MH Focus Groups |
| S224 | MH Diaries |
| S223 | MH Cognition |
| S222 | MH Attitude |
| S221 | S203 OR S204 OR S205 OR S206 OR S207 OR S208 OR S209 OR S210 OR S211 OR S212 OR S213 OR S214 OR S215 OR S216 OR S217 OR S218 OR S219 OR S220 |
| S220 |  |
| S219 | guideline* N3 (clinical or consensus or practice or development or validate* or standard* or adhere*) |
| S218 | retrospective N3 (stud* or design*) |
| S217 | random* N3 (control* or clinical or allocat*) |
| S216 | prospective N3 (stud* or design* or analysis) |
| S215 | longitudinal* N3 (stud* or design* or analysis) |
| S214 | (followup or follow-up) N3 (stud* or design* or analysis) |
| S213 | cohort N3 (stud* or design* or analysis) |
| S212 | (case control or case-control) N3 (stud* or design*) |
| S211 | PT systematic review |
| S210 | MH Literature Review+ |
| S209 | MH Guideline Adherence |
| S208 | MH Practice Guidelines |
| S207 | PT Clinical Trial |
| S206 | MH Randomized Controlled Trials |
| S205 | MH Clinical Trials+ |
| S204 | MH Prospective Studies |
| S203 | MH Case Control Studies |
| S202 | S24 AND S55 AND S201 |
| S201 | S56 OR S57 OR S58 OR S59 OR S60 OR S61 OR S62 OR S63 OR S64 OR S65 OR S66 OR S67 OR S68 OR S69 OR S70 OR S71 OR S72 OR S73 OR S74 OR S75 OR S76 OR S77 OR S78 OR S79 OR S80 OR S81 OR S82 OR S83 OR S84 OR S85 OR S86 OR S87 OR S88 OR S89 OR S90 OR S91 OR S92 OR S93 OR S94 OR S95 OR S96 OR S97 OR S98 OR S99 OR S100 OR S101 OR S102 OR S103 OR S104 OR S105 OR S106 OR S107 OR S108 OR S109 OR S110 OR S111 OR S112 OR S113 OR S114 OR S115 OR S116 OR S117 OR S118 OR S119 OR S120 OR S121 OR S122 OR S123 OR S124 OR S125 OR S126 OR S127 OR S128 OR S129 OR S130 OR S131 OR S132 OR S133 OR S134 OR S135 OR S136 OR S137 OR S138 OR S139 OR S140 OR S141 OR S142 OR S143 OR S144 OR S145 OR S146 OR S147 OR S148 OR S149 OR S150 OR S151 OR S152 OR S153 OR S154 OR S155 OR S156 OR S157 OR S158 OR S159 OR S160 OR S161 OR S162 OR S163 OR S164 OR S165 OR S166 OR S167 OR S168 OR S169 OR S170 OR S171 OR S172 OR S173 OR S174 OR S175 OR S176 OR S177 OR S178 OR S179 OR S180 OR S181 OR S182 OR S183 OR S184 OR S185 OR S186 OR S187 OR S188 OR S189 OR S190 OR S191 OR S192 OR S193 OR S194 OR S195 OR S196 OR S197 OR S198 OR S199 OR S200 |
| S200 | yoga |
| S199 | whirlpool* |
| S198 | warm compress* |
| S197 | walking N3 aid* |
| S196 | walkers |
| S195 | vibration therap* |
| S194 | vapocoolant spray |
| S193 | ultrasound |
| S192 | transcutaneous electrical stimulation |
| S191 | traditional Chinese medicine |
| S190 | traction |
| S189 | thoracolumbosacral orthosis |
| S188 | tape or taping |
| S187 | stretching |
| S186 | strengthen* |
| S185 | strain counterstrain |
| S184 | spray N1 stretch |
| S183 | soft tissue therap* |
| S182 | (shockwave* or shock wave* or shock-wave*) N3 (ultrasonic or therap* or radiation) |
| S181 | short wave* or short-wave* |
| S180 | self-manage* or self manage* |
| S179 | seat cushion* |
| S178 | Russian stimulation |
| S177 | return* N2 work |
| S176 | rehab or rehabilitat* |
| S175 | radiant light |
| S174 | pulsed N3 (electromagnetic or magnetic or radio frequency or energy) |
| S173 | postur* N3 (correct* or educat* or instruct* or train*) |
| S172 | (post-operative or postoperative) N3 (care or manage* or therap* or period) |
| S171 | pilates |
| S170 | pillow* |
| S169 | photo-acupuncture |
| S168 | physiotherap* |
| S167 | physical therap* |
| S166 | Percutaneous Electric* Nerve Stimulation |
| S165 | (patient* or client*) N3 (educat* or train*) |
| S164 | passive modalit* |
| S163 | orthotic* |
| S162 | Neuromuscular Electrical Stimulation |
| S161 | myofascial release |
| S160 | muscle relaxant* |
| S159 | muscle energy technique* |
| S158 | muscle activation |
| S157 | (multimodal* or multi-modal* or multi modal*) N4 (treatment* or approach or care or therap* or procedure* or package* or manage*) |
| S156 | moxibustion |
| S155 | moist air bath* |
| S154 | (mobilisat* or mobilizat*) N4 (osteopath* or orthopedic* or orthopaedic* or lumbar or spinal) |
| S153 | microwave* |
| S152 | Microcurrent Electrical Neuromuscular Stimulation |
| S151 | manual therap* |
| S150 | manipulat* N3 (therap* or treatment* or spinal or osteopath*) |
| S149 | magnetic N3 (necklace* or therap* or bracelet*) |
| S148 | lumbar support* |
| S147 | low level laser* |
| S146 | laser* N3 (phototherapy or irradiation or biostimulation or light or therap*) |
| S145 | kinesiotap* |
| S144 | ergonomic* |
| S143 | electroanalgesia |
| S142 | iontophoresis |
| S141 | infrared |
| S140 | interferential current* |
| S139 | ice N3 (therap* or pack* or compress or massage or immersion or soak or treatment or therap*) |
| S138 | hydrotherap* |
| S137 | hospital-based rehabilitat* |
| S136 | high energy shock wave* or high-energy shock wave* or HESW |
| S135 | (heat* or hot) N3 (therap* or pack* or compress or massage or lamp or pad or bath or soak or tub or bottle or superficial or therapeutic) |
| S134 | H-Wave Device Stimulation or HWDS |
| S133 | galvanic stimulation |
| S132 | fluidotherap* |
| S131 | flexion-distraction or flexion distraction |
| S130 | exercise or exercises or exercising |
| S129 | electrotherapy |
| S128 | electromodalit* |
| S127 | electromagnet* and (radiation or therap*) |
| S126 | electrogalvanic stimulation |
| S125 | electro-acupuncture |
| S124 | electric* N3 (stimulation or EMS or heating pad*) |
| S123 | diathermy |
| S122 | deep tissue therap* |
| S121 | cryotherap* |
| S120 | crutches |
| S119 | corset or corsets |
| S118 | core stabili* |
| S117 | community based rehabilitation |
| S116 | cold N3 (therap* or pack* or compress or massage or immersion or soak or treatment or therap*) |
| S115 | cognitive behavioural therap* |
| S114 | cognitive behavioral therap* |
| S113 | chiropract* |
| S112 | canes |
| S111 | braces or brace or bracing |
| S110 | back N2 work |
| S109 | back school* |
| S108 | back belt* |
| S107 | assistive device* |
| S106 | anti-depressant* or antidepressant* |
| S105 | alexander technique |
| S104 | advice or advise or advised |
| S103 | acupunctur* |
| S102 | acupressure |
| S101 | acetaminophen |
| S100 | (MH "Work Capacity Evaluation") |
| S99 | (MH "Wheelchairs") |
| S98 | (MH "Vibration/TU") |
| S97 | (MH "Transcutaneous Electric Nerve Stimulation") |
| S96 | (MH "Restraint, Physical") |
| S95 | (MH "Physical Fitness") |
| S94 | (MH "Assistive Technology Devices") |
| S93 | (MH "Self Care") |
| S92 | (MH "Rehabilitation+") |
| S91 | (MH "Postoperative Period") |
| S90 | (MH "Physical Therapy") |
| S89 | (MH "Patient Education") |
| S88 | (MH "Outpatient Service") |
| S87 | (MH "Orthopedic Equipment and Supplies") |
| S86 | (MH "Manipulation, Orthopedic") OR (MH "Manipulation, Chiropractic") OR (MH "Manipulation, Osteopathic") OR (MH "Manual Therapy") |
| S85 | (MH "Muscle Relaxants, Central") |
| S84 | (MH "Medicine, Chinese Traditional") |
| S83 | (MH "Massage+") |
| S82 | (MH "Magnet Therapy") |
| S81 | (MH "Phototherapy") |
| S80 | (MH "Laser Therapy") |
| S79 | (MH "Hydrotherapy") |
| S78 | (MH "Heat-Cold Application") |
| S77 | (MH "Immobilization") |
| S76 | (MH "Rehabilitation Centers") |
| S75 | (MH "Fluid Therapy") |
| S74 | (MH "Exercise+") |
| S73 | (MH "Ergonomics") |
| S72 | (MH "Electroacupuncture") |
| S71 | (MH "Electric Stimulation") |
| S70 | (MH "Diathermy") |
| S69 | (MH "Cryotherapy") |
| S68 | (MH "Alternative Therapies") |
| S67 | (MH "Community Health Services") |
| S66 | (MH "Combined Modality Therapy") |
| S65 | (MH "Cognitive Therapy+") |
| S64 | (MH "Casts") |
| S63 | (MH "Biofeedback") |
| S62 | (MH "Behavior Therapy") |
| S61 | (MH "Bedding and Linens") |
| S60 | (MH "Antidepressive Agents") |
| S59 | (MH "Analgesics+") |
| S58 | (MH "Acupuncture") |
| S57 | (MH "Acupressure") |
| S56 | (MH "Analgesics, Nonnarcotic+") |
| S55 | S25 OR S26 OR S27 OR S28 OR S29 OR S30 OR S31 OR S32 OR S33 OR S34 OR S35 OR S36 OR S37 OR S38 OR S39 OR S40 OR S41 OR S42 OR S43 OR S44 OR S45 OR S46 OR S47 OR S48 OR S49 OR S50 OR S51 OR S52 OR S53 OR S54 |
| S54 | traction |
| S53 | surgical fixation device* |
| S52 | (spondylosis or spondylolisthesis) N2 surg*) |
| S51 | (spine or spinal or back) N2 (decompression or endoscop* or fusion or surg*) |
| S50 | root N2 decompress* |
| S49 | radiculopath* N3 surg* |
| S48 | postlaminectom* |
| S47 | nucleotom* |
| S46 | microendoscop* |
| S45 | microdis?ectom* |
| S44 | lumbosacral N2 (decompression or endoscop* or fusion or surg*) |
| S43 | lumbar N2 (decompression or endoscop* or fusion or surg*) |
| S42 | laminotom* |
| S41 | (disk or disc) N3 (surgery or surgical) |
| S40 | fusion N2 decompress* |
| S39 | foraminectom* |
| S38 | foraminotom* |
| S37 | electroanalgesia |
| S36 | dis?otom* |
| S35 | dis?ectom* |
| S34 | arthrodesis |
| S33 | (MH "Surgical Fixation Devices") |
| S32 | (MH "Spine+/SU") |
| S31 | (MH "Spinal Fusion") |
| S30 | (MH "Spinal Diseases+/SU") |
| S29 | (MH "Nerve Compression Syndromes/SU") |
| S28 | (MH "Laminectomy") |
| S27 | (MH "Diskectomy") |
| S26 | (MH "Decompression, Surgical") |
| S25 | (MH "Arthrodesis") |
| S24 | S1 OR S2 OR S3 OR S4 OR S5 OR S6 OR S7 OR S8 OR S9 OR S10 OR S11 OR S12 OR S13 OR S14 OR S15 OR S16 OR S17 OR S18 OR S19 OR S20 OR S21 OR S22 OR S23 |
| S23 | spondylosis |
| S22 | (spine or spinal) N4 (condition* or degenerat* or disable* or disabilit* or disease* or disorder* or osteoarthritis or pain or stenos?s) |
| S21 | (sacral or sacroiliac) N3 (pain* or facet* or nerve root* or osteoarthr* or radicul* or stenos* or spondylo* or zygapophys* or injur* or discomfort* or dysfunction* or sore* or herniat*) |
| S20 | radiculopath* |
| S19 | Piriformis syndrome* |
| S18 | lumbar N3 (pain or facet or nerve root* or osteoarthritis or radicul* or spinal stenosis or spondylo* or zygapophys*) |
| S17 | low* back pain* |
| S16 | (lumbarsacral* or lumbosacral) N3 (pain* or facet* or nerve root* or osteoarthr* or radicul* or stenos* or spondylo* or zygapophys* or injur* or discomfort* or dysfunction* or sore* or herniat*) |
| S15 | lumbar disk* N3 (extruded or degenerat* or herniat* or prolapse* or sequestered or slipped) |
| S14 | lumbar disc* N3 (extruded or degenerat* or herniat* or prolapse* or sequestered or slipped) |
| S13 | intervertebral N3 (disease* or bulging or pain* or extru* or degenerat* or displac* or herniat* or prolaps* or sequest* or slipped* or protru* or avuls*) |
| S12 | back pain or back-pain |
| S11 | backache* N3 (injur* or pain*) |
| S10 | back N3 (ache* or injur* or pain*) |
| S9 | (MH "Piriformis Syndrome") |
| S8 | (MH "Sciatica") |
| S7 | (MH "Radiculopathy") |
| S6 | (MH "Osteoarthritis, Spine") |
| S5 | (MH "Lumbar Vertebrae/IN") |
| S4 | (MH "Spinal Diseases+") |
| S3 | MH Intervertebral Disc Degeneration |
| S2 | (MH "Back Pain+") |
| S1 | (MH "Back Injuries") |

**CCGI_PostsurgicalRehab_2021_CochraneCentral**

| 1 | exp Back Injuries/ |
| --- | --- |
| 2 | exp Back Pain/ |
| 3 | Intervertebral Disc Degeneration/ |
| 4 | Intervertebral Disc Displacement/ |
| 5 | Osteoarthritis, Spine/ |
| 6 | Piriformis Muscle Syndrome/ |
| 7 | Radiculopathy/ |
| 8 | Sciatica/ |
| 9 | Spinal Diseases/ |
| 10 | Spinal Stenosis/ |
| 11 | (back adj3 (ache* or injur* or pain*)).ab,ti. |
| 12 | (backache* adj3 (injur* or pain*)).ab,ti. |
| 13 | (back pain* or back-pain*).ab,ti. |
| 14 | (intervertebral adj3 (disease* or bulging or pain* or extru* or degenerat* or displac* or herniat* or prolaps* or sequest* or slipped* or protru* or avuls*)).ab,ti. |
| 15 | (lumbar disc* adj3 (extruded or degenerat* or herniat* or prolapse* or sequestered or slipped)).ab,ti. |
| 16 | (lumbar disk* adj3 (extruded or degenerat* or herniat* or prolapse* or sequestered or slipped)).ab,ti. |
| 17 | ((lumbarsacral* or lumbosacral) adj32 (pain* or facet* or nerve root* or osteoarthr* or radicul* or stenos* or spondylo* or zygapophys* or injur* or discomfort* or dysfunction* or sore* or herniat*)).ab,ti. |
| 18 | "low* back pain*".ab,ti. |
| 19 | (lumbar adj3 (pain or facet or nerve root* or osteoarthritis or radicul* or spinal stenosis or spondylo* or zygapophys*)).ab,ti. |
| 20 | "Piriformis syndrome*".ab,ti. |
| 21 | "radiculopath*".ab,ti. |
| 22 | ((sacral or sacroiliac) adj3 (pain* or facet* or nerve root* or osteoarthr* or radicul* or stenos* or spondylo* or zygapophys* or injur* or discomfort* or dysfunction* or sore* or herniat*)).ab,ti. |
| 23 | ((spine or spinal) adj4 (condition* or degenerat* or disable* or disabilit* or disease* or disorder* or osteoarthritis or pain or stenos?s)).ab,ti. |
| 24 | spondylosis.ab,ti. |
| 25 | or/1-24 [**back pain] |
| 26 | Arthrodesis/ |
| 27 | Decompression, Surgical/ |
| 28 | Diskectomy/ |
| 29 | Foraminotomy/ |
| 30 | Intervertebral Disc Degeneration/su [Surgery] |
| 31 | Intervertebral Disc Displacement/su [Surgery] |
| 32 | Intervertebral Disc/su [Surgery] |
| 33 | Laminectomy/ |
| 34 | Low Back Pain/su [Surgery] |
| 35 | Lumbar Fusion/ |
| 36 | Nerve Compression Syndromes/su [Surgery] |
| 37 | Radiculopathy/su [Surgery] |
| 38 | Sciatica/su [Surgery] |
| 39 | Spinal Diseases/su [Surgery] |
| 40 | Spinal Fusion/ |
| 41 | Spinal Stenosis/su [Surgery] |
| 42 | Spondylosis/su [Surgery] |
| 43 | Spondylolisthesis/su [Surgery] |
| 44 | Spine/su [Surgery] |
| 45 | Surgical Fixation Devices/ |
| 46 | Surgical Procedures, Minimally Invasive/ |
| 47 | arthrodesis.ab,ti. |
| 48 | "dis?ectom*".ab,ti. |
| 49 | "dis?otom*".ab,ti. |
| 50 | electroanalgesia.ab,ti. |
| 51 | (failed adj back).ab,ti. |
| 52 | "foraminotom*".ab,ti. |
| 53 | "foraminectom*".ab,ti. |
| 54 | (fusion adj2 decompress*).ab,ti. |
| 55 | ((disk or disc) adj3 (surgery or surgical)).ab,ti. |
| 56 | "laminectom*".ab,ti. |
| 57 | "laminoplast*".ab,ti. |
| 58 | "laminotom*".ab,ti. |
| 59 | (lumbar adj2 (decompression or endoscop* or fusion or surg*)).ab,ti. |
| 60 | (lumbosacral adj2 (decompression or endoscop* or fusion or surg*)).ab,ti. |
| 61 | "microdis?ectom*".ab,ti. |
| 62 | "microendoscop*".ab,ti. |
| 63 | (mini* adj3 invas* adj3 surgery).ab,ti. |
| 64 | "nucleotom*".ab,ti. |
| 65 | "postlaminectom*".ab,ti. |
| 66 | (radiculopath* adj3 surg*).ab,ti. |
| 67 | (root adj2 decompress*).ab,ti. |
| 68 | ((spine or spinal or back) adj2 (decompression or endoscop* or fusion or surg*)).ab,ti. |
| 69 | ((spondylosis or spondylolisthesis) adj2 surg*).ab,ti. |
| 70 | "surgical fixation device*".ab,ti. |
| 71 | traction.ab,ti. |
| 72 | or/26-71 [** spine surgery] |
| 73 | Acetaminophen/ |
| 74 | Acupressure/ |
| 75 | Acupuncture/ |
| 76 | exp Acupuncture Therapy/ |
| 77 | Analgesics/ |
| 78 | Antidepressive Agents/ |
| 79 | exp Anti-Inflammatory Agents, Non-Steroidal/ |
| 80 | "Bedding and Linens"/ |
| 81 | Behavior Therapy/ |
| 82 | exp Biofeedback, Psychology/ |
| 83 | Casts, Surgical/ |
| 84 | exp Cognitive Therapy/ |
| 85 | Combined Modality Therapy/ |
| 86 | Community-Based Participatory Research/ |
| 87 | Community Health Services/ |
| 88 | Community Participation/ |
| 89 | Complementary Therapies/ |
| 90 | Cryotherapy/ |
| 91 | exp Diathermy/ |
| 92 | exp Electric Stimulation Therapy/ |
| 93 | Electroacupuncture/ |
| 94 | Ergonomics/ |
| 95 | exp Exercise/ |
| 96 | exp Exercise Movement Techniques/ |
| 97 | exp Exercise Therapy/ |
| 98 | Fluid Therapy/ |
| 99 | High-Energy Shock Waves/tu [Therapeutic Use] |
| 100 | exp Hydrotherapy/ |
| 101 | Laser Therapy, Low-Level/ |
| 102 | Low-Level Light Therapy/ |
| 103 | Magnetic Field Therapy/ |
| 104 | Massage/ |
| 105 | exp Medicine, Chinese Traditional/ |
| 106 | Muscle Relaxants, Central/ |
| 107 | exp Musculoskeletal Manipulations/ |
| 108 | exp Orthopedic Equipment/ |
| 109 | Outpatient Clinics, Hospital/ |
| 110 | Patient Education as Topic/ |
| 111 | Physical Therapy Modalities/ |
| 112 | exp Rehabilitation/ |
| 113 | Rehabilitation, Vocational/ |
| 114 | Return to Work/ |
| 115 | Self Care/ |
| 116 | Self-Help Devices/ |
| 117 | Physical Fitness/ |
| 118 | Restraint, Physical/ |
| 119 | Transcutaneous Electric Nerve Stimulation/ |
| 120 | Vibration/ |
| 121 | Wheelchairs/ |
| 122 | Work Capacity Evaluation/ |
| 123 | Work Engagement/ |
| 124 | acetaminophen.ab,ti. |
| 125 | acupressure.ab,ti. |
| 126 | "acupunctur*".ab,ti. |
| 127 | (advice or advise or advised).ab,ti. |
| 128 | alexander technique.ab,ti. |
| 129 | (anti-depressant* or antidepressant*).ab,ti. |
| 130 | "assistive device*".ab,ti. |
| 131 | "back belt*".ab,ti. |
| 132 | "back school*".ab,ti. |
| 133 | (back adj2 work).ab,ti. |
| 134 | (braces or brace or bracing).ab,ti. |
| 135 | canes.ab,ti. |
| 136 | chiropract*.ab,ti. |
| 137 | "cognitive behavioral therap*".ab,ti. |
| 138 | "cognitive behavioural therap*".ab,ti. |
| 139 | (cold adj3 (therap* or pack* or compress or massage or immersion or soak or treatment or therap*)).ab,ti. |
| 140 | Community-based rehabilitation.ab,ti. |
| 141 | Community based rehabilitation.ab,ti. |
| 142 | "core stabili*".ab,ti. |
| 143 | (corset or corsets).ab,ti. |
| 144 | crutches.ab,ti. |
| 145 | cryotherap*.ab,ti. |
| 146 | "deep tissue therap*".ab,ti. |
| 147 | diathermy.ab,ti. |
| 148 | (electric* adj3 (stimulation or EMS or heating pad*)).ab,ti. |
| 149 | electro-acupuncture.ab,ti. |
| 150 | (electrogalvanic stimulation or EGS).ab,ti. |
| 151 | (electromagnet* and (radiation or therap*)).ab,ti. |
| 152 | electromodalit*.ab,ti. |
| 153 | electrotherapy.ab,ti. |
| 154 | (exercise or exercises or exercising).ab,ti. |
| 155 | (flexion-distraction or flexion distraction).ab,ti. |
| 156 | fluidotherap*.ab,ti. |
| 157 | galvanic stimulation.ab,ti. |
| 158 | (H-Wave Device Stimulation or HWDS).ab,ti. |
| 159 | ((heat* or hot) adj3 (therap* or pack* or compress or massage or lamp or pad or bath or soak or tub or bottle or superficial or therapeutic)).ab,ti. |
| 160 | (high energy shock wave* or high-energy shock wave* or HESW).ab,ti. |
| 161 | hospital-based rehabilitat*.ab,ti. |
| 162 | hospital based rehabilitat*.ab,ti. |
| 163 | "hydrotherap*".ab,ti. |
| 164 | (ice adj3 (therap* or pack* or compress or massage or immersion or soak or treatment or therap*)).ab,ti. |
| 165 | "interferential current*".ab,ti. |
| 166 | infrared.ab,ti. |
| 167 | iontophoresis.ab,ti. |
| 168 | electroanalgesia.ab,ti. |
| 169 | ergonomic*.ab,ti. |
| 170 | kinesiotap*.ab,ti. |
| 171 | (laser* adj3 (phototherapy or irradiation or biostimulation or light or therap*)).ab,ti. |
| 172 | "low level laser*".ab,ti. |
| 173 | "lumbar support*".ab,ti. |
| 174 | (magnetic adj3 (necklace* or therap* or bracelet*)).ab,ti. |
| 175 | (manipulat* adj3 (therap* or treatment* or spinal or osteopath*)).ab,ti. |
| 176 | "manual therap*".ab,ti. |
| 177 | Microcurrent Electrical Neuromuscular Stimulation.ab,ti. |
| 178 | microwave*.ab,ti. |
| 179 | ((mobilisat* or mobilizat*) adj4 (osteopath* or orthopedic* or orthopaedic* or lumbar or spinal)).ab,ti. |
| 180 | "moist air bath*".ab,ti. |
| 181 | moxibustion.ab,ti. |
| 182 | ((multimodal* or multi-modal* or multi modal*) adj4 (treatment* or approach or care or therap* or procedure* or package* or manage*)).ab,ti. |
| 183 | muscle activation.ab,ti. |
| 184 | "muscle energy technique*".ab,ti. |
| 185 | myofascial release.ab,ti. |
| 186 | (Neuromuscular Electrical Stimulation or NMES).ab,ti. |
| 187 | orthotic*.ab,ti. |
| 188 | "passive modalit*".ab,ti. |
| 189 | ((patient* or client*) adj3 (educat* or train*)).ab,ti. |
| 190 | "Percutaneous Electric* Nerve Stimulation".ab,ti. |
| 191 | (physical adj therap*).ab,ti. |
| 192 | physiotherap*.ab,ti. |
| 193 | photo-acupuncture.ab,ti. |
| 194 | pillow*.ab,ti. |
| 195 | pilates.ab,ti. |
| 196 | ((post-operative or postoperative) adj3 (care or manage* or therap* or period)).ab,ti. |
| 197 | (postur* adj3 (correct* or educat* or instruct* or train*)).ab,ti. |
| 198 | (pulsed adj3 (electromagnetic or magnetic or radio frequency or energy)).ab,ti. |
| 199 | radiant light.ab,ti. |
| 200 | (rehab or rehabilitat*).ab,ti. |
| 201 | (return* adj2 work).ab,ti. |
| 202 | RTW.ab,ti. |
| 203 | Russian stimulation.ab,ti. |
| 204 | "seat adj cushion*".ab,ti. |
| 205 | (self-manage* or self manage*).ab,ti. |
| 206 | (short wave* or short-wave*).ab,ti. |
| 207 | ((shockwave* or shock wave* or shock-wave*) adj3 (ultrasonic or therap* or radiation)).ab,ti. |
| 208 | "soft tissue therap*".ab,ti. |
| 209 | "spray and stretch".ab,ti. |
| 210 | strain-counterstrain.ab,ti. |
| 211 | strengthen*.ab,ti. |
| 212 | stretching.ab,ti. |
| 213 | (tape or taping).ab,ti. |
| 214 | thoracolumbosacral orthosis.ab,ti. |
| 215 | traction.ab,ti. |
| 216 | traditional Chinese medicine.ab,ti. |
| 217 | (transcutaneous electrical stimulation or TENS).ab,ti. |
| 218 | ultrasound.ab,ti. |
| 219 | vapocoolant spray.ab,ti. |
| 220 | "vibration therap*".ab,ti. |
| 221 | walkers.ab,ti. |
| 222 | "walking adj3 aid*".ab,ti. |
| 223 | "warm compress*".ab,ti. |
| 224 | whirlpool*.ab,ti. |
| 225 | yoga.ab,ti. |
| 226 | or/73-225 [**rehabilitation] |
| 227 | Case-Control Studies/ |
| 228 | Cohort Studies/ |
| 229 | Controlled Clinical Trials as Topic/ |
| 230 | Longitudinal Studies/ |
| 231 | Randomized Controlled Trials as Topic/ |
| 232 | ((case control or case-control) adj3 (stud* or design*)).ab,ti. |
| 233 | (cohort adj3 (stud* or design* or analysis)).ab,ti. |
| 234 | controlled clinical trial.pt. |
| 235 | ((followup or follow-up) adj3 (stud* or design* or analysis)).ab,ti. |
| 236 | (longitudinal* adj3 (stud* or design* or analysis)).ab,ti. |
| 237 | (prospective adj3 (stud* or design* or analysis)).ab,ti. |
| 238 | (random* and (control* or clinical or allocat*)).ab,ti. |
| 239 | randomized controlled trial.pt. |
| 240 | (retrospective adj3 (stud* or design*)).ab,ti. |
| 241 | Practice Guidelines as Topic/ |
| 242 | Guideline Adherence/ |
| 243 | practice guideline.pt. |
| 244 | (guideline* adj3 (clinical or consensus or practice or development or validate* or standard* or adhere*)).ab,ti. |
| 245 | Review Literature as Topic/ |
| 246 | Systematic Reviews as Topic/ |
| 247 | systematic review.pt. |
| 248 | (systematic adj3 (review* or overview*)).ab,ti. |
| 249 | or/227-248 [**study designs] |
| 250 | Attitude/ |
| 251 | Awareness/ |
| 252 | Diary as Topic/ |
| 253 | Focus Groups/ |
| 254 | Grounded Theory/ |
| 255 | Interviews as Topic/ |
| 256 | Mindfulness/ |
| 257 | Motivation/ |
| 258 | Narration/ |
| 259 | Observation/ |
| 260 | Patient Preference/ |
| 261 | Patient Satisfaction/ |
| 262 | Perception/ |
| 263 | Personal Narratives as Topic/ |
| 264 | Personal Satisfaction/ |
| 265 | Qualitative Research/ |
| 266 | Self Report/ |
| 267 | "attitude*".ab,ti. |
| 268 | (aware or awareness).ab,ti. |
| 269 | "belief*".ab,ti. |
| 270 | (diary or diaries).ab,ti. |
| 271 | "ethnograph*".ab,ti. |
| 272 | "expectation*".ab,ti. |
| 273 | "experience*".ab,ti. |
| 274 | feelings.ab,ti. |
| 275 | (focus adj2 group*).ab,ti. |
| 276 | (grounded adj theor*).ab,ti. |
| 277 | "interview*".ab,ti. |
| 278 | mindfulness.ab,ti. |
| 279 | "motivat*".ab,ti. |
| 280 | (mixed adj method*).ab,ti. |
| 281 | (narration* or narrative*).ab,ti. |
| 282 | (observe* or observation*).ab,ti. |
| 283 | "opinion*".ab,ti. |
| 284 | (observe* or observation*).ab,ti. |
| 285 | "opinion*".ab,ti. |
| 286 | (patient* adj2 journey).ab,ti. |
| 287 | (perceiv* or perception*).ab,ti. |
| 288 | "perspective*".ab,ti. |
| 289 | "preference*".ab,ti. |
| 290 | qualitative.ab,ti. |
| 291 | satisfaction.ab,ti. |
| 292 | (self adj3 report*).ab,ti. |
| 293 | or/250-292 [**qualitative/experience] |
| 294 | 25 and 72 and 226 and 249 [**effectiveness] |
| 295 | 25 and 72 and 226 and 293 [**qualitative] |
| 296 | 294 or 295 |
| 297 | limit 296 to yr="2020 - 2021" |

**CCGI_PostsurgicalRehab_2021_ICL**

| [S1](https://www.chiroindex.org/?action=set&setId=4587008) | Subject:\"Back Pain / surgery\" OR Subject:\"Decompression, Surgical\" OR Subject:Diskectomy OR Subject:\"Dislocations / surgery\" OR Subject:\"Intervertebral Disc Degeneration / surgery\" OR Subject:\"Intervertebral Disc Displacement / surgery\" OR Subject:Laminectomy OR Subject:\"Low Back Pain / surgery\" OR Subject:\"Lumbar Vertebrae / surgery\" OR Subject:\"Radiculopathy / surgery\" OR Subject:\"Sciatica / surgery\" OR Subject:\"Spinal Fusion\" OR Subject:\"Spondylosis / surgery\" OR Subject:\"Spine / surgery\", Peer Review only |
| --- | --- |
| [S2](https://www.chiroindex.org/?action=set&setId=4587011) | All Fields:decompression OR All Fields:arthrodesis OR All Fields:foraminotomy OR All Fields:laminectomy OR All Fields:\"Nerve Compression Syndromes\" OR All Fields:\"spinal surgery\" OR All Fields:electroanalgesia OR All Fields:laminoplast* OR All Fields:laminotom* OR All Fields:microdiscectom* OR All Fields:microdiskectom* OR All Fields:nucleotom* OR All Fields:\"postlaminectom*, Peer Review only |
| [S3](https://www.chiroindex.org/?action=set&setId=4587012) | All Fields:spine OR All Fields:spinal OR All Fields:back, Peer Review only |
| [S4](https://www.chiroindex.org/?action=set&setId=4587013) | All Fields:decompression OR All Fields:endoscop* OR All Fields:fusion OR All Fields:surg*, Peer Review only |
| [S5](https://www.chiroindex.org/?action=set&setId=4587014) | All Fields:spine OR All Fields:spinal OR All Fields:back, Peer Review only AND All Fields:decompression OR All Fields:endoscop* OR All Fields:fusion OR All Fields:surg*, Peer Review only |
| [S6](https://www.chiroindex.org/?action=set&setId=4587020) | Subject:\"Back Pain / surgery\" OR Subject:\"Decompression, Surgical\" OR Subject:Diskectomy OR Subject:\"Dislocations / surgery\" OR Subject:\"Intervertebral Disc Degeneration / surgery\" OR Subject:\"Intervertebral Disc Displacement / surgery\" OR Subject:Laminectomy OR Subject:\"Low Back Pain / surgery\" OR Subject:\"Lumbar Vertebrae / surgery\" OR Subject:\"Radiculopathy / surgery\" OR Subject:\"Sciatica / surgery\" OR Subject:\"Spinal Fusion\" OR Subject:\"Spondylosis / surgery\" OR Subject:\"Spine / surgery\", Peer Review only OR All Fields:decompression OR All Fields:arthrodesis OR All Fields:foraminotomy OR All Fields:laminectomy OR All Fields:\"Nerve Compression Syndromes\" OR All Fields:\"spinal surgery\" OR All Fields:electroanalgesia OR All Fields:laminoplast* OR All Fields:laminotom* OR All Fields:microdiscectom* OR All Fields:microdiskectom* OR All Fields:nucleotom* OR All Fields:\"postlaminectom*, Peer Review only OR All Fields:spine OR All Fields:spinal OR All Fields:back, Peer Review only AND All Fields:decompression OR All Fields:endoscop* OR All Fields:fusion OR All Fields:surg*, Peer Review only |
| [S7](https://www.chiroindex.org/?action=set&setId=4587021) | Subject:\"Postoperative Care\" OR Subject:\"Postoperative Complications / diagnosis\" OR Subject:\"Rehabilitation\" OR Subject:\"Rehabilitation / methods\" OR Subject:\"Rehabilitation / standards\" OR Subject:\"Rehabilitation Centers\" OR Subject:\"Rehabilitation, Vocational\", Peer Review only |
| [S8](https://www.chiroindex.org/?action=set&setId=4587022) | All Fields:decompression OR All Fields:arthrodesis OR All Fields:foraminotomy OR All Fields:laminectomy OR All Fields:\"Nerve Compression Syndromes\" OR All Fields:\"spinal surgery\" OR All Fields:electroanalgesia OR All Fields:laminoplast* OR All Fields:laminotom* OR All Fields:microdiscectom* OR All Fields:microdiskectom* OR All Fields:nucleotom* OR All Fields:\"postlaminectom*, Peer Review only AND Subject:\"Postoperative Care\" OR Subject:\"Postoperative Complications / diagnosis\" OR Subject:\"Rehabilitation\" OR Subject:\"Rehabilitation / methods\" OR Subject:\"Rehabilitation / standards\" OR Subject:\"Rehabilitation Centers\" OR Subject:\"Rehabilitation, Vocational\", Peer Review only |
| [S9](https://www.chiroindex.org/?action=set&setId=4587024) | All Fields:spine OR All Fields:spinal OR All Fields:back, Peer Review only AND All Fields:decompression OR All Fields:endoscop* OR All Fields:fusion OR All Fields:surg*, Peer Review only AND Subject:\"Postoperative Care\" OR Subject:\"Postoperative Complications / diagnosis\" OR Subject:\"Rehabilitation\" OR Subject:\"Rehabilitation / methods\" OR Subject:\"Rehabilitation / standards\" OR Subject:\"Rehabilitation Centers\" OR Subject:\"Rehabilitation, Vocational\", Peer Review only |

**CCGI_PostSurgicalRehab_2021_RehabSportsMed**

| **#** | **Query** |
| --- | --- |
| S231 | S225 AND (S223 OR S228) Limiters - Publication Date: 20191001-20210831; Publication Type: Academic Journal Expanders - Apply equivalent subjects Search modes - Boolean/Phrase |
| S230 | S225 AND (S223 OR S228) |
| S229 | S225 AND (S223 OR S228) |
| S228 | S50 OR S51 OR S52 OR S53 OR S54 OR S55 OR S56 OR S57 OR S58 OR S59 OR S60 OR S61 OR S62 OR S63 OR S64 OR S65 OR S66 OR S67 OR S68 OR S69 OR S70 OR S71 OR S72 OR S73 OR S74 OR S75 OR S76 OR S77 OR S78 OR S79 OR S80 OR S81 OR S82 OR S83 OR S84 OR S85 OR S86 OR S87 OR S88 OR S89 OR S90 OR S91 OR S92 OR S93 OR S94 OR S95 OR S96 OR S97 OR S98 OR S99 OR S100 OR S101 OR S102 OR S103 OR S104 OR S105 OR S106 OR S107 OR S108 OR S109 OR S110 OR S111 OR S112 OR S113 OR S114 OR S115 OR S116 OR S117 OR S118 OR S119 OR S120 OR S121 OR S122 OR S123 OR S124 OR S125 OR S126 OR S127 OR S128 OR S129 OR S130 OR S131 OR S132 OR S133 OR S134 OR S135 OR S136 OR S137 OR S138 OR S139 OR S140 OR S141 OR S142 OR S143 OR S144 OR S145 OR S146 OR S147 OR S148 OR S149 OR S150 OR S151 OR S152 OR S153 OR S154 OR S155 OR S156 OR S157 OR S158 OR S159 OR S160 OR S161 OR S162 OR S163 OR S164 OR S165 OR S166 OR S167 OR S168 OR S169 OR S170 OR S171 OR S172 OR S173 OR S174 OR S175 OR S176 OR S177 OR S178 OR S179 OR S180 |
| S227 | S76 AND S225 |
| S226 | S223 AND S225 |
| S225 | S24 AND S221 AND S222 |
| S224 | S192 OR S193 OR S194 OR S195 OR S196 OR S197 OR S198 OR S199 OR S200 OR S201 OR S202 OR S203 OR S204 OR S205 OR S206 OR S207 OR S208 OR S209 OR S210 OR S211 OR S212 OR S213 OR S214 OR S215 OR S216 OR S217 OR S218 OR S219 OR S220 |
| S223 | S181 OR S182 OR S183 OR S184 OR S185 OR S186 OR S187 OR S188 OR S189 OR S190 OR S191 |
| S222 | S50 OR S51 OR S52 OR S53 OR S54 OR S55 OR S56 OR S57 OR S58 OR S59 OR S60 OR S61 OR S62 OR S63 OR S64 OR S65 OR S66 OR S67 OR S68 OR S69 OR S70 OR S71 OR S72 OR S73 OR S74 OR S75 OR S76 OR S77 OR S78 OR S79 OR S80 OR S81 OR S82 OR S83 OR S84 OR S85 OR S86 OR S87 OR S88 OR S89 OR S90 OR S91 OR S92 OR S93 OR S94 OR S95 OR S96 OR S97 OR S98 OR S99 OR S100 OR S101 OR S102 OR S103 OR S104 OR S105 OR S106 OR S107 OR S108 OR S109 OR S110 OR S111 OR S112 OR S113 OR S114 OR S115 OR S116 OR S117 OR S118 OR S119 OR S120 OR S121 OR S122 OR S123 OR S124 OR S125 OR S126 OR S127 OR S128 OR S129 OR S130 OR S131 OR S132 OR S133 OR S134 OR S135 OR S136 OR S137 OR S138 OR S139 OR S140 OR S141 OR S142 OR S143 OR S144 OR S145 OR S146 OR S147 OR S148 OR S149 OR S150 OR S151 OR S152 OR S153 OR S154 OR S155 OR S156 OR S157 OR S158 OR S159 OR S160 OR S161 OR S162 OR S163 OR S164 OR S165 OR S166 OR S167 OR S168 OR S169 OR S170 OR S171 OR S172 OR S173 OR S174 OR S175 OR S176 OR S177 OR S178 OR S179 OR S180 |
| S221 | S25 OR S26 OR S27 OR S28 OR S29 OR S30 OR S31 OR S32 OR S33 OR S34 OR S35 OR S36 OR S37 OR S38 OR S39 OR S40 OR S41 OR S42 OR S43 OR S44 OR S45 OR S46 OR S47 OR S48 OR S49 |
| S220 | self N3 report* |
| S219 | satisfaction |
| S218 | qualitative |
| S217 | preference* |
| S216 | perspective* |
| S215 | perceiv* or perception* |
| S214 | patient* N2 journey* |
| S213 | opinion* |
| S212 | observe or observation* |
| S211 | narration* or narrative* |
| S210 | motivat* |
| S209 | mindfulness |
| S208 | interview* |
| S207 | grounded theor* |
| S206 | focus N2 group* |
| S205 | feelings |
| S204 | experience* |
| S203 | expectation* |
| S202 | ethnograph* |
| S201 | diary or diaries |
| S200 | belief* |
| S199 | aware or awareness |
| S198 | attitude* |
| S197 | DE PERCEPTION |
| S196 | DE QUALITY of life |
| S195 | DE MOTIVATION (Psychology) |
| S194 | DE MIND-body walking |
| S193 | DE SITUATIONAL awareness |
| S192 | DE ATTITUDE (Psychology) |
| S191 | systematic N3 (review* or overview*) |
| S190 | guideline* N3 (clinical or consensus or practice or development or validate* or standard* or adhere*) |
| S189 | retrospective N3 (stud* or design*) |
| S188 | random* N3 (control* or clinical or allocat*) |
| S187 | prospective N3 (stud* or design* or analysis) |
| S186 | longitudinal* N3 (stud* or design* or analysis) |
| S185 | (followup or follow-up) N3 (stud* or design* or analysis) |
| S184 | cohort N3 (stud* or design* or analysis) |
| S183 | (case control or case-control) N3 (stud* or design*) |
| S182 | DE PHYSICIAN adherence |
| S181 | DE COHORT analysis |
| S180 | yoga |
| S179 | whirlpool* |
| S178 | warm compress* |
| S177 | walking N3 aid* |
| S176 | walkers |
| S175 | vibration therap* |
| S174 | vapocoolant spray |
| S173 | ultrasound |
| S172 | transcutaneous electrical stimulation |
| S171 | traditional Chinese medicine |
| S170 | traction |
| S169 | thoracolumbosacral orthosis |
| S168 | tape or taping |
| S167 | stretching |
| S166 | strengthen* |
| S165 | strain counterstrain |
| S164 | spray N1 stretch |
| S163 | soft tissue therap* |
| S162 | (shockwave* or shock wave* or shock-wave*) N3 (ultrasonic or therap* or radiation) |
| S161 | short wave* or short-wave* |
| S160 | self-manage* or self manage* |
| S159 | seat cushion* |
| S158 | Russian stimulation |
| S157 | return* N2 work |
| S156 | rehab or rehabilitat* |
| S155 | radiant light |
| S154 | pulsed N3 (electromagnetic or magnetic or radio frequency or energy) |
| S153 | postur* N3 (correct* or educat* or instruct* or train*) |
| S152 | (post-operative or postoperative) N3 (care or manage* or therap* or period) |
| S151 | pilates |
| S150 | pillow* |
| S149 | photo-acupuncture |
| S148 | physiotherap* |
| S147 | physical therap* |
| S146 | Percutaneous Electric* Nerve Stimulation |
| S145 | (patient* or client*) N3 (educat* or train*) |
| S144 | passive modalit* |
| S143 | orthotic* |
| S142 | Neuromuscular Electrical Stimulation |
| S141 | myofascial release |
| S140 | muscle relaxant* |
| S139 | muscle energy technique* |
| S138 | muscle activation |
| S137 | (multimodal* or multi-modal* or multi modal*) N4 (treatment* or approach or care or therap* or procedure* or package* or manage*) |
| S136 | moxibustion |
| S135 | moist air bath* |
| S134 | (mobilisat* or mobilizat*) N4 (osteopath* or orthopedic* or orthopaedic* or lumbar or spinal) |
| S133 | microwave* |
| S132 | Microcurrent Electrical Neuromuscular Stimulation |
| S131 | manual therap* |
| S130 | manipulat* N3 (therap* or treatment* or spinal or osteopath*) |
| S129 | magnetic N3 (necklace* or therap* or bracelet*) |
| S128 | lumbar support* |
| S127 | low level laser* |
| S126 | laser* N3 (phototherapy or irradiation or biostimulation or light or therap*) |
| S125 | kinesiotap* |
| S124 | ergonomic* |
| S123 | electroanalgesia |
| S122 | iontophoresis |
| S121 | infrared |
| S120 | interferential current* |
| S119 | ice N3 (therap* or pack* or compress or massage or immersion or soak or treatment or therap*) |
| S118 | hydrotherap* |
| S117 | hospital-based rehabilitat* |
| S116 | high energy shock wave* or high-energy shock wave* or HESW |
| S115 | (heat* or hot) N3 (therap* or pack* or compress or massage or lamp or pad or bath or soak or tub or bottle or superficial or therapeutic) |
| S114 | H-Wave Device Stimulation or HWDS |
| S113 | galvanic stimulation |
| S112 | fluidotherap* |
| S111 | flexion-distraction or flexion distraction |
| S110 | exercise or exercises or exercising |
| S109 | electrotherapy |
| S108 | electromodalit* |
| S107 | electromagnet* and (radiation or therap*) |
| S106 | electrogalvanic stimulation |
| S105 | electro-acupuncture |
| S104 | electric* N3 (stimulation or EMS or heating pad*) |
| S103 | diathermy |
| S102 | deep tissue therap* |
| S101 | cryotherap* |
| S100 | crutches |
| S99 | corset or corsets |
| S98 | core stabili* |
| S97 | community based rehabilitation |
| S96 | cold N3 (therap* or pack* or compress or massage or immersion or soak or treatment or therap*) |
| S95 | cognitive behavioural therap* |
| S94 | cognitive behavioral therap* |
| S93 | chiropract* |
| S92 | canes |
| S91 | braces or brace or bracing |
| S90 | back N2 work |
| S89 | back school* |
| S88 | back belt* |
| S87 | assistive device* |
| S86 | anti-depressant* or antidepressant* |
| S85 | alexander technique |
| S84 | advice or advise or advised |
| S83 | acupunctur* |
| S82 | acupressure |
| S81 | acetaminophen |
| S80 | DE WHEELCHAIRS |
| S79 | DE TRANSCUTANEOUS electrical nerve stimulation |
| S78 | DE PHYSICAL fitness |
| S77 | DE HEALTH self-care |
| S76 | DE REHABILITATION |
| S75 | DE POSTOPERATIVE care |
| S74 | DE PHYSICAL therapy practice |
| S73 | DE OUTPATIENT services in hospitals |
| S72 | DE ORTHOPEDIC apparatus |
| S71 | DE MANIPULATION therapy |
| S70 | DE MASSAGE OR DE MASSAGE therapy |
| S69 | DE MAGNETOTHERAPY |
| S68 | DE PHOTOTHERAPY |
| S67 | DE ALTERNATIVE medicine |
| S66 | DE HYDROTHERAPY |
| S65 | DE HEAT |
| S64 | DE THERAPEUTIC immobilization |
| S63 | DE FLUID therapy |
| S62 | DE EXERCISE OR DE EXERCISE therapy |
| S61 | DE ERGONOMICS |
| S60 | DE ELECTROACUPUNCTURE |
| S59 | DE ELECTROTHERAPEUTICS |
| S58 | DE DIATHERMY |
| S57 | DE COGNITIVE therapy |
| S56 | DE SURGICAL casts |
| S55 | DE BIOLOGICAL control systems |
| S54 | DE ANTIDEPRESSANTS |
| S53 | DE ANALGESICS |
| S52 | DE ACUPUNCTURE |
| S51 | DE ACUPRESSURE |
| S50 | DE ACETAMINOPHEN |
| S49 | traction |
| S48 | surgical fixation device* |
| S47 | (spondylosis or spondylolisthesis) N2 surg*) |
| S46 | (spine or spinal or back) N2 (decompression or endoscop* or fusion or surg*) |
| S45 | root N2 decompress* |
| S44 | radiculopath* N3 surg* |
| S43 | postlaminectom* |
| S42 | nucleotom* |
| S41 | microendoscop* |
| S40 | microdis?ectom* |
| S39 | lumbosacral N2 (decompression or endoscop* or fusion or surg*) |
| S38 | lumbar N2 (decompression or endoscop* or fusion or surg*) |
| S37 | laminotom* |
| S36 | (disk or disc) N3 (surgery or surgical) |
| S35 | fusion N2 decompress* |
| S34 | foraminectom* |
| S33 | foraminotom* |
| S32 | electroanalgesia |
| S31 | dis?otom* |
| S30 | dis?ectom* |
| S29 | arthrodesis |
| S28 | DE JOINT surgery |
| S27 | DE SPINAL fusion |
| S26 | DE SURGICAL procedures |
| S25 | DE ARTHRODESIS |
| S24 | S1 OR S2 OR S3 OR S4 OR S5 OR S6 OR S7 OR S8 OR S9 OR S10 OR S11 OR S12 OR S13 OR S14 OR S15 OR S16 OR S17 OR S18 OR S19 OR S20 OR S21 OR S22 OR S23 |
| S23 | spondylosis |
| S22 | (spine or spinal) N4 (condition* or degenerat* or disable* or disabilit* or disease* or disorder* or osteoarthritis or pain or stenos?s) |
| S21 | (sacral or sacroiliac) N3 (pain* or facet* or nerve root* or osteoarthr* or radicul* or stenos* or spondylo* or zygapophys* or injur* or discomfort* or dysfunction* or sore* or herniat*) |
| S20 | radiculopath* |
| S19 | Piriformis syndrome* |
| S18 | lumbar N3 (pain or facet or nerve root* or osteoarthritis or radicul* or spinal stenosis or spondylo* or zygapophys*) |
| S17 | low* back pain* |
| S16 | (lumbarsacral* or lumbosacral) N3 (pain* or facet* or nerve root* or osteoarthr* or radicul* or stenos* or spondylo* or zygapophys* or injur* or discomfort* or dysfunction* or sore* or herniat*) |
| S15 | lumbar disk* N3 (extruded or degenerat* or herniat* or prolapse* or sequestered or slipped) |
| S14 | lumbar disc* N3 (extruded or degenerat* or herniat* or prolapse* or sequestered or slipped) |
| S13 | intervertebral N3 (disease* or bulging or pain* or extru* or degenerat* or displac* or herniat* or prolaps* or sequest* or slipped* or protru* or avuls*) |
| S12 | back pain or back-pain |
| S11 | backache* N3 (injur* or pain*) |
| S10 | back N3 (ache* or injur* or pain*) |
| S9 | DE PIRIFORMIS syndrome |
| S8 | DE SCIATICA |
| S7 | DE RADICULOPATHY |
| S6 | DE INTERVERTEBRAL disk hernias |
| S5 | DE LUMBAR pain |
| S4 | DE SPINE diseases |
| S3 | DE INTERVERTEBRAL disk displacement |
| S2 | DE SPINAL stenosis |
| S1 | DE BACK injuries OR DE BACKACHE |

**Additional file 6: Studies identified from databases and excluded at full-text screening stage**

| **Record #** | **Author (year)** | **Title** | **Primary exclusion reason** |
| --- | --- | --- | --- |
| 3345 | Aalto et al. (2011)^2^ | Postoperative rehabilitation does not improve functional outcome in lumbar spinal stenosis: a prospective study with 2-year postoperative follow-up | Ineligible intervention |
| 3632 | Abbott et al. (2010)^3^ | Early rehabilitation targeting cognition, behavior, and motor function after lumbar fusion: a randomized controlled trial | Ineligible population |
| 11289 | Abbott et al. (2010)^4^ | Early rehabilitation targeting cognition, behavior, and motor function after lumbar fusion: a randomized controlled trial | Duplicate |
| 3393 | Abbott et al. (2011)^5^ | Leg pain and psychological variables predict outcome 2-3 years after lumbar fusion surgery | Ineligible research question |
| 10925 | Abbott et al. (2011)^6^ | The effectiveness of physiotherapeutic rehabilitation and issues of outcome prediction after lumbar fusion surgery | Ineligible study design/publication type |
| 10993 | Abbott et al. (2011)^7^ | Leg pain and psychological variables predict outcome 2-3 years after lumbar fusion surgery | Duplicate |
| 12858 | Abbott et al. (2011)^8^ | Patients' experience post-lumbar fusion regarding back problems, recovery and expectations in terms of the international classification of functioning, disability and health | Ineligible research question |
| 12859 | Abbott et al. (2011)^8^ | Patients' experience post-lumbar fusion regarding back problems, recovery and expectations in terms of the international classification of functioning, disability and health | Duplicate |
| 27 | Akyuz and Firidin (2022){Akyuz, 2022 #27} | Bilateral ultrasound-guided erector spinae plane block for postoperative persistent low back pain in lumbar disc surgery | Ineligible intervention |
| 5525 | Alaranta et al. (1986)^9^ | Rehabilitation after surgery for lumbar disc herniation: results of a randomized clinical trial | Ineligible population |
| 10177 | Alaranta et al. (1986)^10^ | Rehabilitation after surgery for lumbar disc herniation: Results of a randomized clinical trial | Duplicate |
| 10446 | Alaranta et al. (1986)^10^ | Rehabilitation after surgery for lumbar disc herniation: Results of a randomized clinical trial | Duplicate |
| 11160 | Alaranta et al. (1986)^11^ | Rehabilitation after surgery for lumbar disc herniation: results of a randomized clinical trial | Duplicate |
| 431 | Aldemir and Gurkan (2021)^12^ | The effect of pedometer-supported walking and telemonitoring after disc hernia surgery on pain and disability levels and quality of life | Duplicate |
| 1038 | Aldemir and Gurkan (2021)^12^ | The effect of pedometer-supported walking and telemonitoring after disc hernia surgery on pain and disability levels and quality of life | Duplicate |
| 1565 | Aldemir and Gurkan (2021)^12^ | The effect of pedometer-supported walking and telemonitoring after disc hernia surgery on pain and disability levels and quality of life | Duplicate |
| 10782 | Aldrete (2003)^13^ | Epidural injections of indomethacin for postlaminectomy syndrome: a preliminary report | Ineligible intervention |
| 1832 | Alimohammadi et al. (2015)^14^ | The effect of continuing care on patient's quality-of-life after disc surgery in neurosurgery and very important person wards | Ineligible population |
| 3764 | Andersen et al. (2009)^15^ | The effect of electrical stimulation on lumbar spinal fusion in older patients: a randomized, controlled, multi-center trial: part 2: fusion rates | Ineligible research question |
| 3765 | Andersen et al. (2009)^16^ | The effect of electrical stimulation on lumbar spinal fusion in older patients: a randomized, controlled, multi-center trial: part 1: functional outcome | Ineligible research question |
| 11266 | Andersen et al. (2009)^17^ | The effect of electrical stimulation on lumbar spinal fusion in older patients: a randomized, controlled, multi-center trial: part 2: fusion rates | Duplicate |
| 11269 | Andersen et al. (2009)^18^ | The effect of electrical stimulation on lumbar spinal fusion in older patients: a randomized, controlled, multi-center trial: part 1: functional outcome | Duplicate |
| 3613 | Andersen et al. (2010)^19^ | Fusion mass bone quality after uninstrumented spinal fusion in older patients | Ineligible research question |
| 2270 | Anderson et al. (2015)^20^ | Chronic Opioid Therapy After Lumbar Fusion Surgery for Degenerative Disc Disease in a Workers' Compensation Setting | Ineligible intervention |
| 350 | Anderson et al. (2018)^21^ | Multilevel Lumbar Fusion Is a Risk Factor for Lower Return to Work Rates Among Workers' Compensation Subjects With Degenerative Disc Disease | Cannot retrieve |
| 372 | Angelini et al. (2020)^22^ | Healthcare practitioners' experiences of postoperative pain management in lumbar spine surgery care-A qualitative study | Ineligible research question |
| 11916 | Archer et al. (2014)^23^ | Improving surgical spine outcomes through a targeted postoperative rehabilitation approach | Ineligible study design/publication type |
| 2139 | Archer et al. (2016)^24^ | Cognitive-Behavioral-Based Physical Therapy for Patients With Chronic Pain Undergoing Lumbar Spine Surgery: A Randomized Controlled Trial | Ineligible population |
| 7042 | Archer et al. (2016)^24^ | Cognitive-Behavioral-Based Physical Therapy for Patients with Chronic Pain Undergoing Lumbar Spine Surgery: A Randomized Controlled Trial | Duplicate |
| 11137 | Archer et al. (2016)^25^ | Cognitive-Behavioral-Based Physical Therapy for Patients With Chronic Pain Undergoing Lumbar Spine Surgery: a Randomized Controlled Trial | Duplicate |
| 11693 | Archer et al. (2016)^26^ | Cognitive-Behavioral-Based Physical Therapy for Patients with Chronic Pain Undergoing Lumbar Spine Surgery: a Randomized Controlled Trial | Duplicate |
| 10346 | Archer et al. (2017)^27^ | Cognitive-behavioral-based physical therapy for patients with chronic pain undergoing lumbar spine surgery: A randomized controlled trial: Erratum | Ineligible study design/publication type |
| 13009 | Aspegren and Burt (1994)^28^ | A study of postspinal surgery cases in chiropractic offices | Ineligible intervention |
| 24 | Atrarod et al. (2019)^29^ | Predictive factors for return to work after lumbar discectomy | Ineligible research question |
| 466 | Avila et al. (2023){Avila, 2023 #466} | Effectiveness of Cognitive Functional Therapy Versus Core Exercises and Manual Therapy in Patients with Chronic Low Back Pain after Spinal Surgery: Randomized Controlled Trial | Ineligile interevention |
| 93 | Akhshi et al. (2022){Bakhshi, 2022 #93} | Effect of Foot Reflexology and Gentle Stretching Exercises on Pain Intensity in Patients after Spine Surgery: A Randomized Controlled Trial | Ineligible population |
| 4099 | Bentsen et al. (2008)^30^ | The pain experience and future expectations of chronic low back pain patients following spinal fusion | Ineligible research question |
| 2001 | Benyamin et al. (2016)^31^ | MILD Is an Effective Treatment for Lumbar Spinal Stenosis with Neurogenic Claudication: MiDAS ENCORE Randomized Controlled Trial | Ineligible intervention |
| 3995 | Bible et al. (2009)^32^ | Postoperative bracing after spine surgery for degenerative conditions: a questionnaire study | Ineligible research question |
| 7789 | Bolesta (2013)^33^ | Meaningful improvement in perioperative pain control after lumbar surgery | Ineligible study design/publication type |
| 11210 | Bono et al. (2017)^34^ | The effect of short (2-weeks) versus long (6-weeks) post-operative restrictions following lumbar discectomy: a prospective randomized control trial | Duplicate |
| 8166 | Bouche et al. (2011)^35^ | Long term outcome of Lumbar Discectomy: Results from a biopsychosocial perspective | Ineligible research question |
| 5623 | Bourghli et al. (2019)^36^ | Opioids and analgesics use after adult spinal deformity surgery correlates with sagittal alignment and preoperative analgesic pattern | Ineligible population |
| 4878 | Brox et al. (2003)^37^ | Randomized clinical trial of lumbar instrumented fusion and cognitive intervention and exercises in patients with chronic low back pain and disc degeneration | Ineligible population |
| 10744 | Burke et al. (1994)^38^ | Return to work/work retention outcomes of a functional restoration program. A multi-center, prospective study with a comparison group | Ineligible population |
| 12148 | Byungho et al. (2016)^39^ | Early individualised manipulative rehabilitation following lumbar open laser microdiscectomy improves early post-operative functional disability: A randomized, controlled pilot study | Small sample size |
| 8581 | Caby et al. (2010)^40^ | Effects of a five-week intensive and multidisciplinary spine-specific functional restoration program in chronic low back pain patients with or without surgery. [French]; Effets d'un programme intensif et multidisciplinaire de restauration fonctionnelle du rachis de cinq semaines chez des sujets lombalgiques chroniques operes et non operes | Ineligible population |
| 5776 | Cai et al. (2019)^41^ | Effects of rehabilitation nursing on minimally invasive surgery and the application of long-term activity function in patients with intervertebral disc herniation | Ineligible intervention |
| 512 | Cerezci et al. (2023){Cerezci, 2023 #512} | Importance of Physiotherapy after Lumbar Microdiscectomy | Small sample size |
| 2384 | Chen et al. (2015)^42^ | Is rehabilitation intervention during hospitalization enough for functional improvements in patients undergoing lumbar decompression surgery? A prospective randomized controlled study | Duplicate |
| 7276 | Chen et al. (2015)^43^ | Is rehabilitation intervention during hospitalization enough for functional improvements in patients undergoing lumbar decompression surgery? A prospective randomized controlled study | Ineligible population |
| 11117 | Chen et al. (2015)^44^ | Is rehabilitation intervention during hospitalization enough for functional improvements in patients undergoing lumbar decompression surgery? A prospective randomized controlled study | Duplicate |
| 11760 | Chen et al. (2015)^45^ | Is rehabilitation intervention during hospitalization enough for functional improvements in patients undergoing lumbar decompression surgery? A prospective randomized controlled study | Duplicate |
| 981 | Cherkin et al. (1995)^46^ | Physician views about treating low back pain. The results of a national survey | Ineligible population |
| 10830 | Choi et al. (2005)^47^ | The effect of early isolated lumbar extension exercise program for patients with herniated disc undergoing lumbar discectomy | Duplicate |
| 10374 | Choi et al. (2013)^48^ | Combination of pregabalin and dexamethasone for postoperative pain and functional outcome in patients undergoing lumbar spinal surgery: A randomized placebo-controlled trial | Ineligible intervention |
| 10836 | Choi et al. (2013)^49^ | Combination of pregabalin and dexamethasone for postoperative pain and functional outcome in patients undergoing lumbar spinal surgery: a randomized placebo-controlled trial | Duplicate |
| 4849 | Christensen et al. (2003)^50^ | Importance of the back-cafe concept to rehabilitation after lumbar spinal fusion: a randomized clinical study with a 2-year follow-up | Ineligible intervention |
| 11468 | Christensen et al. (2003)^51^ | Importance of the back-cafe concept to rehabilitation after lumbar spinal fusion: a randomized clinical study with a 2-year follow-up | Duplicate |
| 3559 | Cienciala et al. (2010)^52^ | [Dynamic neutralization using the Dynesys system for treatment of degenerative disc disease of the lumbar spine] | Ineligible research question |
| 8369 | Cienciala et al. (2010)^53^ | Dynamic neutralization using the dynesys system for treatment of degenerative disc disease of the lumbar spine. [Czech]; Osetreni degenerativniho onemocneni bederni patere metodou dynamicke neutralizace systemem dynesys | Duplicate |
| 1769 | Claus et al. (2017)^54^ | An evidence-based information booklet helps reduce fear-avoidance beliefs after first-time discectomy for disc prolapse | Small sample size |
| 11184 | Claus et al. (2017)^55^ | An evidence-based information booklet helps reduce fear-avoidance beliefs after first-time discectomy for disc prolapse | Duplicate |
| 8565 | Corbin(2010)^56^ | Point of view: Clinical outcomes after posterolateral lumbar fusion in workers compensation patients: A case-control study | Ineligible study design/publication type |
| 285 | Coronado et al. (2020)^57^ | Psychosocial Mechanisms of Cognitive-Behavioral-Based Physical Therapy Outcomes After Spine Surgery: Preliminary Findings From Mediation Analyses | Ineligible research question |
| 1110 | Coronado et al. (2020)^58^ | Psychosocial Mechanisms of Cognitive-Behavioral-Based Physical Therapy Outcomes After Spine Surgery: preliminary Findings From Mediation Analyses | Duplicate |
| 12266 | Cox and Boswell (2000)^59^ | Integrating complementary health care in outpatient surgery for discectomy: the patient's perspective | Ineligible research question |
| 7636 | Dailey et al. (2014)^60^ | Guideline update for the performance of fusion procedures for degenerative disease of the lumbar spine. Part 14: Brace therapy as an adjunct to or substitute for lumbar fusion | Duplicate |
| 811 | Danielsen et al. (2000)^61^ | Early aggressive exercise for postoperative rehabilitation after discectomy | Small sample size |
| 10761 | Danielsen et al. (2000)^62^ | Early aggressive exercise for postoperative rehabilitation after discectomy | Duplicate |
| 596 | Debono et al. (2019)^63^ | Benefits of Enhanced Recovery After Surgery for fusion in degenerative spine surgery: impact on outcome, length of stay, and patient satisfaction | Ineligible intervention |
| 4502 | Donaldson et al. (2006)^64^ | Comparison of usual surgical advice versus a nonaggravating six-month gym-based exercise rehabilitation program post-lumbar discectomy: results at one-year follow-up | Ineligible population |
| 11512 | Donaldson et al. (2006)^65^ | Comparison of usual surgical advice versus a nonaggravating six-month gym-based exercise rehabilitation program post-lumbar discectomy: results at one-year follow-up | Duplicate |
| 10756 | Donceel et al. (1999)^66^ | Return to work after surgery for lumbar disc herniation. A rehabilitation-oriented approach in insurance medicine | Duplicate |
| 848 | Donceel et al. (1999)^67^ | Return to work after surgery for lumbar disc herniation. A rehabilitation-oriented approach in insurance medicine | Duplicate of relevant RCTs on the same intervention |
| 877 | Duculan et al. (2020)^68^ | 195. Fostering physical activity after complex lumbar spine surgery: long-term results of a randomized trial | Ineligible study design/publication type |
| 10880 | Ebata et al. (2017)^69^ | Role of Weekly Teriparatide Administration in Osseous Union Enhancement within Six Months After Posterior or Transforaminal Lumbar Interbody Fusion for Osteoporosis-Associated Lumbar Degenerative Disorders: a Multicenter, Prospective Randomized Study | Ineligible population |
| 11101 | Ebenbichler et al. (2015)^70^ | Twelve-year follow-up of a randomized controlled trial of comprehensive physiotherapy following disc herniation operation | Duplicate |
| 419 | Erdogan et al. (2020)^71^ | Effectiveness of Computer Assisted Training of Patients Undergoing Lumbar Disc Herniation Surgery | Duplicate |
| 11539 | Erdogmus et al. (2007)^72^ | Physiotherapy-based rehabilitation following disc herniation operation: results of a randomized clinical trial | Duplicate |
| 3133 | Feng et al. (2012)^73^ | [Analysis of therapeuttic effect of lower limb sensation disorder after lumbar disc herniation operation treated with plum-blossom needle along meridians] | Ineligible population |
| 11343 | Feng et al. (2012)^74^ | Analysis of therapeuttic effect of lower limb sensation disorder after lumbar disc herniation operation treated with plum-blossom needle along meridians | Duplicate |
| 595 | Ferrari et al. (2022){Ferrari, 2022 #595} | Thoughts and concerns of patients at hospital discharge after lumbar spine surgery. A qualitative study | Ineligible population |
| 2267 | Flanigan et al. (2015)^75^ | Psychological Factors Affecting Rehabilitation and Outcomes Following Elective Orthopaedic Surgery | Ineligible study design/publication type |
| 603 | Gallon (1989)^76^ | Perception of disability in chronic back pain patients: a long-term follow-up | Ineligible intervention |
| 10803 | Gerling et al. (2017)^77^ | Risk Factors for Reoperation in Patients Treated Surgically for Degenerative Spondylolisthesis: a Subanalysis of the 8-year Data From the SPORT Trial | Ineligible population |
| 63 | Gilmore et al. (2019)^78^ | Predictors of substantial improvement in physical function six months after lumbar surgery: is early post-operative walking important? A prospective cohort study | Ineligible outcome |
| 2172 | Green et al. (2016)^79^ | Physiotherapeutic Rehabilitation Following Lumbar Total Disc Replacement: A Retrospective Study | Ineligible population |
| 12419 | Green et al. (2016)^80^ | Physiotherapeutic Rehabilitation Following Lumbar Total Disc Replacement: A Retrospective Study | Duplicate |
| 627 | Greenwood et al. (2022){Greenwood, 2022 #627} | A qualitative evaluation of participants experiences of living with back pain, lumbar fusion surgery, and post-operative rehabilitation | Ineligible population |
| 2067 | Grieff et al. (2016)^81^ | Use of liposomal bupivacaine in the postoperative management of posterior spinal decompression | Small sample size |
| 5973 | Guo et al. (2019)^82^ | Rehabilitation nursing for patient rehabilitation after minimally invasive spine surgery | Ineligible outcome |
| 105 | Guo et al. (2022){Guo, 2022 #105} | Effects of a WeChat-based individualized post-discharge rehabilitation program on patients with lumbar fusion surgery | Ineligible population |
| 11484 | Hakkinen et al. (2005)^83^ | Effects of home strength training and stretching versus stretching alone after lumbar disk surgery: a randomized study with a 1-year follow-up | Duplicate |
| 7520 | Harper et al. (2014)^84^ | An open-label pilot study of pulsed electromagnetic feld therapy in the treatment of failed back surgery syndrome pain | Ineligible population |
| 3480 | He et al. (2010)^85^ | [Case-control study on comprehensive rehabilitation for postoperative pain after spinal surgery] | Cannot retrieve |
| 90 | He et al. (2021){He, 2021 #90} | Effect of continuous nursing based on wechat platform on postoperative rehabilitation of patients with lumbar disc herniation | Duplicate |
| 651 | Heard et al. (2023){Heard, 2023 #651} | The Impact of Physical Therapy After Lumbar Fusion Surgery | Ineligible population |
| 7300 | Hedlund (2015)^86^ | The long-term outcome of lumbar fusion in the Swedish lumbar spine study | Ineligible research question |
| 197 | Heo et al. (2021)^87^ | Multicentre randomised controlled clinical trial of electroacupuncture with usual care for patients with non-acute pain after back surgery | Ineligible intervention |
| 222 | Heo et al. (2021)^87^ | Multicentre randomised controlled clinical trial of electroacupuncture with usual care for patients with non-acute pain after back surgery | Duplicate |
| 8550 | Herkowitz (2010)^88^ | Degenerative lumbar spondylolisthesis: A surgeon's perspective of 30 years in practice | Ineligible study design/publication type |
| 1781 | Hoffman et al. (2018)^89^ | Minimally Invasive Decompression and Physiotherapy for Lumbar Spinal Stenosis in Geriatric Patients | Small sample size |
| 90 | Hou et al. (2019)^90^ | The Effectiveness and Safety of Utilizing Mobile Phone-Based Programs for Rehabilitation After Lumbar Spinal Surgery: Multicenter, Prospective Randomized Controlled Trial | Ineligible population |
| 622 | Hou et al. (2019)^91^ | The Effectiveness and Safety of Utilizing Mobile Phone-Based Programs for Rehabilitation After Lumbar Spinal Surgery: Multicenter, Prospective Randomized Controlled Trial | Duplicate |
| 10675 | Hou et al. (2019)^92^ | The Effectiveness and Safety of Utilizing Mobile Phone-Based Programs for Rehabilitation After Lumbar Spinal Surgery: multicenter, Prospective Randomized Controlled Trial | Duplicate |
| 1503 | Ilves et al. (2017)^93^ | Effectiveness of postoperative home-exercise compared with usual care on kinesiophobia and physical activity in spondylolisthesis: A randomized controlled trial | Ineligible population |
| 10891 | Ilves et al. (2017)^94^ | Effectiveness of postoperative home-exercise compared with usual care on kinesiophobia and physical activity in spondylolisthesis: a randomized controlled trial | Duplicate |
| 10968 | Ilves et al. (2017)^95^ | Postoperative back-specific and aerobic training does not improve trunk muscle strength and imbalance after lumbar spine fusion: a randomized controlled trial | Ineligible study design/publication type |
| 10984 | Ishida et al. (2010)^96^ | Early active rehabilitation after surgery for lumbar disc herniation: a prospective randomized control trial | Ineligible study design/publication type |
| 11934 | Ishida et al. (2010)^97^ | Early active rehabilitation after surgery for lumbar disc herniation: a prospective, randomized control trial | Duplicate |
| 5083 | Johnson (2001)^98^ | Electrical stimulation and lumbar spinal fusion | Ineligible study design/publication type |
| 1649 | Kaliya-Perumal et al. (2017)^99^ | Retrospective radiological outcome analysis following teriparatide use in elderly patients undergoing multilevel instrumented lumbar fusion surgery | Small sample size |
| 1696 | Kaptain et al. (2017)^100^ | Patient participation in postoperative pain assessment after spine surgery in a recovery unit | Small sample size |
| 8700 | Kara et al. (2009)^101^ | Physiotherapy results after nucleoplasty | Ineligible study design/publication type |
| 2351 | Katz (2015)^102^ | Surgery for lumbar spinal stenosis: informed patient preferences should weigh heavily | Ineligible population |
| 10900 | Khurana et al. (2014)^103^ | Postoperative pain and long-term functional outcome after administration of gabapentin and pregabalin in patients undergoing spinal surgery | Ineligible intervention |
| 7294 | Kim et al. (2015)^104^ | Rehabilitation with osteopathic manipulative treatment after lumbar disc surgery: A randomised, controlled pilot study | Ineligible study design/publication type |
| 11223 | Kim et al. (2017)^105^ | Comparative study of the efficacy of transdermal buprenorphine patches and prolonged-release tramadol tablets for postoperative pain control after spinal fusion surgery: a prospective, randomized controlled non-inferiority trial | Ineligible population |
| 11091 | Kim et al. (2018)^106^ | Sex-specific and Age-specific Analgesia for Early Postoperative Pain Management after Lumbar Decompressive Surgery: a Randomized Clinical Trial | Duplicate |
| 89 | Kim et al. (2019)^107^ | Sex-specific and Age-specific Analgesia for Early Postoperative Pain Management After Lumbar Decompressive Surgery: A Randomized Clinical Trial | Ineligible population |
| 4208 | Kitze and Angermeyer (2007)^108^ | [Pain related impairment and the ability to function in herniated disc patients during rehabilitation] | Ineligible research question |
| 11841 | Kjellby-Wendt et al. (2001)^109^ | Early active rehabilitation after surgical treatment of lumbar disc herniation. A prospective, randomized study of psychometric assessment and a 5 year evaluation | Ineligible study design/publication type |
| 10807 | Knape (1970)^110^ | Bezitramide, an orally active analgesic. An investigation on pain following operations for lumbar disc protrusion (preliminary report) | Ineligible study design/publication type |
| 10808 | Knape (1971)^111^ | Further experiences with bezitramide. Its analgesic action and side effects in patients operated upon for lumbar disc protrusion | Ineligible intervention |
| 740 | Koszela et al. (2022){Koszela, 2022 #740} | Age and Sex in Back Pain Intensity-Retrospective Study of Conservatory vs. Surgical Discopathy Treatment | Ineligible study design |
| 11114 | Kryuchkova (2015)^112^ | Efficacy of baclosan in combined therapy of muscular-tonic pain syndrome in the early postoperative period in patients after microdiscectomy | Small sample size |
| 11238 | Kulikov et al. (2018)^113^ | New approach for evaluating the effectiveness of whole-body magnetic field therapy in the rehabilitation of patients with lumbar discectomy | Duplicate |
| 11935 | Kullich et al. (2012)^114^ | Dynamic spinal traction in inpatient rehabilitation of low back pain: Sustained improvement of pain experience | Ineligible study design/publication type |
| 3292 | Lam and Groff (2011)^115^ | Reoperations after decompression for lumbar spinal stenosis | Ineligible study design/publication type |
| 751 | Lam et al. (2022){Lam, 2022 #751} | The Concerns and Experiences of Patients With Lumbar Spinal Stenosis Regarding Prehabilitation and Recovery After Spine Surgery: A Qualitative Study | Ineligible population |
| 11241 | Lavyne and Bilsky (1992)^116^ | Epidural steroids, postoperative morbidity, and recovery in patients undergoing microsurgical lumbar discectomy | Ineligible intervention |
| 1481 | Lee et al. (2017)^117^ | Efficacy and Safety of Transdermal Buprenorphine versus Oral Tramadol/Acetaminophen in Patients with Persistent Postoperative Pain after Spinal Surgery | Duplicate |
| 1504 | Lee et al. (2017)^118^ | The Efficacy of Vitamin C on Postoperative Outcomes after Posterior Lumbar Interbody Fusion: A Randomized, Placebo-Controlled Trial | Ineligible population |
| 6461 | Lee et al. (2017)^119^ | Efficacy and Safety of Transdermal Buprenorphine versus Oral Tramadol/Acetaminophen in Patients with Persistent Postoperative Pain after Spinal Surgery | Duplicate |
| 10344 | Lee et al. (2017)^117^ | Efficacy and safety of transdermal buprenorphine versus oral tramadol/acetaminophen in patients with persistent postoperative pain after spinal surgery | Duplicate |
| 11201 | Lee et al. (2017)^120^ | The Efficacy of Vitamin C on Postoperative Outcomes after Posterior Lumbar Interbody Fusion: a Randomized, Placebo-Controlled Trial | Duplicate |
| 11220 | Lee et al. (2017)^121^ | Efficacy and Safety of Transdermal Buprenorphine versus Oral Tramadol/Acetaminophen in Patients with Persistent Postoperative Pain after Spinal Surgery | Ineligible intervention |
| 10937 | Li et al. (2014)^122^ | Multimodal pain control protocol after lumbar spinal fusion | Cannot retrieve |
| 1971 | Lindgreen et al. (2016)^123^ | Interdisciplinary Cognitive-Behavioral Therapy as Part of Lumbar Spinal Fusion Surgery Rehabilitation: Experience of Patients With Chronic Low Back Pain | Ineligible population |
| 12268 | Lindgreen et al. (2016)^124^ | Interdisciplinary Cognitive-Behavioral Therapy as Part of Lumbar Spinal Fusion Surgery Rehabilitation | Duplicate |
| 51 | Liow et al. (2019)^125^ | Time Taken to Return to Work Does Not Influence Outcomes of Minimally Invasive Transforaminal Lumbar Interbody Fusion: A 5-Year Follow-Up Study | Ineligible research question |
| 1792 | Liu et al. (2017)^126^ | The effect of systematic lower-limb rehabilitation training in elderly patients undergoing lumbar fusion surgery: a retrospective study | Ineligible population |
| 2659 | Lobner et al. (2014)^127^ | Inpatient or outpatient rehabilitation after herniated disc surgery? - Setting-specific preferences, participation and outcome of rehabilitation | Ineligible outcome |
| 1510 | Lobner et al. (2017)^128^ | Choosing the right rehabilitation setting after herniated disc surgery: Motives, motivations and expectations from the patients' perspective | Ineligible outcome |
| 3994 | Lumawig et al. (2009)^129^ | Dose-dependent inhibition of diclofenac sodium on posterior lumbar interbody fusion rates | Small sample size |
| 7317 | Luo and Zhang (2015)^130^ | Core stabilization exercises, implant fixation and lumbar fusion for degenerative lumbar spondylolisthesis: Lumbar function evaluation. [Chinese] | Ineligible population |
| 1453 | Machado and Pinheiro (2018)^131^ | Early comprehensive physiotherapy after lumbar spine surgery (PEDro synthesis) | Ineligible study design/publication type |
| 7619 | Maffei (2014)^132^ | Massage interest in treatment of premature postoperative pain: Controlled randomized trial. [French]; Interet du massage dans le traitement de douleurs postoperatoires precoces: etude controlee randomisee | Small sample size |
| 5017 | Magnaes et al. (2002)^133^ | [Evaluation of methods in the treatment of lumbar disk prolapse with root avulsion] | Ineligible study design/publication type |
| 1870 | Magnussen (2011)^134^ | Surgery with disc prosthesis may produce better outcomes than multidisciplinary rehabilitation for patients with chronic low back pain | Ineligible study design/publication type |
| 82 | Malik et al. (2018)^135^ | Continued Inpatient Care After Elective 1- to 2-level Posterior Lumbar Fusions Increases 30-day Postdischarge Readmissions and Complications | Ineligible research question |
| 5923 | Malik et al. (2019)^136^ | Discharge to Inpatient Care Facility After Anterior Lumbar Interbody Fusion: Incidence, Predictors, and Postdischarge Outcomes | Ineligible research question |
| 7903 | Manchikanti et al. (2013)^137^ | Cost utility analysis of caudal epidural injections in the treatment of lumbar disc herniation, axial or discogenic low back pain, central spinal stenosis, and post lumbar surgery syndrome | Ineligible study design/publication type |
| 852 | Mancuso et al. (2019)^138^ | Improvement in multiple domains of functional status with increasing physical activity after lumbar surgery: Longitudinal analysis | Ineligible study design/publication type |
| 854 | Mancuso et al. (2019)^139^ | Fostering physical activity after complex lumbar spine surgery: A randomized trial | Ineligible study design/publication type |
| 10531 | Mancuso et al. (2019)^140^ | Fostering physical activity after complex lumbar spine surgery: a randomized trial | Duplicate |
| 67 | Mancuso et al. (2021)^141^ | Qualitative assessment of patients' perspectives and willingness to improve healthy lifestyle physical activity after lumbar surgery | Ineligible research question |
| 944 | Manniche et a. (1993)^142^ | Intensive dynamic back exercises with or without hyperextension in chronic back pain after surgery for lumbar disc protrusion. A clinical trial | Ineligible intervention |
| 10098 | Manniche et a. (1993)^143^ | Clinical trial of postoperative dynamic back exercises after first lumbar discectomy | Duplicate |
| 10734 | manniche et al. (1993)^144^ | Clinical trial of postoperative dynamic back exercises after first lumbar discectomy | Duplicate |
| 10736 | Manniche et al. (1993)^145^ | Intensive dynamic back exercises with or without hyperextension in chronic back pain after surgery for lumbar disc protrusion. A clinical trial | Duplicate |
| 10648 | Manniche et al. (1994)^146^ | Peroperative prednisolone fails to improve the clinical outcome following surgery for prolapsed lumbar intervertebral disc. A randomized controlled trial | Ineligible intervention |
| 1341 | Mannion et al. (2007)^147^ | A randomised controlled trial of post-operative rehabilitation after surgical decompression of the lumbar spine | Ineligible population |
| 11544 | Mannion et al. (2007)^148^ | A randomised controlled trial of post-operative rehabilitation after surgical decompression of the lumbar spine | Duplicate |
| 3548 | Mannion et al. (2010)^149^ | Five-year outcome of surgical decompression of the lumbar spine without fusion | Ineligible research question |
| 761 | Master et al. (2021)^150^ | How many steps per day during the early postoperative period is associated with patient-reported outcomes of disability, pain, and opioid use after lumbar spine surgery? | Ineligible population |
| 1284 | Master et al. (2021)^150^ | How many steps per day during the early postoperative period is associated with patient-reported outcomes of disability, pain, and opioid use after lumbar spine surgery? | Duplicate |
| 831 | Master et al. (2023){Master, 2023 #831} | Combining Wearable Technology and Telehealth Counseling for Rehabilitation after Lumbar Spine Surgery: Feasibility and Acceptability of a Physical Activity Intervention | Ineligible study design/publication type |
| 9715 | Mastronardi et al. (2002)^151^ | Efficacy of the morphine-Adcon-L compound in the management of postoperative pain after lumbar microdiscectomy | Ineligible intervention |
| 989 | Maybin et al. (2018)^152^ | The impact of postoperative physical therapy on patient-reported outcomes in patients undergoing spine surgery | Ineligible study design/publication type |
| 10791 | Mayer et al. (2004)^153^ | A randomized clinical trial of treatment for lumbar segmental rigidity | Ineligible population |
| 3685 | McGregor et al. (2010)^154^ | Function after spinal treatment, exercise and rehabilitation (FASTER): improving the functional outcome of spinal surgery | Ineligible study design/publication type |
| 3372 | McGregor et al. (2011)^155^ | ISSLS prize winner: Function After Spinal Treatment, Exercise, and Rehabilitation (FASTER): a factorial randomized trial to determine whether the functional outcome of spinal surgery can be improved | Duplicate |
| 6241 | McGregor et al. (2011)^156^ | Function after spinal treatment, exercise and rehabilitation (FASTER): A factorial randomised trial to determine whether the functional outcome of spinal surgery can be improved. [] | Ineligible population |
| 11054 | McGregor et al. (2011)^157^ | Function after spinal treatment, exercise and rehabilitation (FASTER): a factorial randomised trial to determine whether the functional outcome of spinal surgery can be improved | Ineligible study design/publication type |
| 11342 | McGregor et al. (2011)^158^ | ISSLS prize winner: function After Spinal Treatment, Exercise, and Rehabilitation (FASTER): a factorial randomized trial to determine whether the functional outcome of spinal surgery can be improved | Duplicate |
| 8477 | Mehren et al. (2010)^159^ | Minimal invasive anterior midline approach to L<inf>2</inf>-L<inf>5</inf>. [German]; Minimalinvasive ventrale mittellinienzugange L<inf>2</inf>-L <inf>5</inf> | Ineligible research question |
| 8671 | Meziat et al. (2009)^160^ | Long-term effects of a stabilization exercise therapy for chronic low back pain | Ineligible study design/publication type |
| 845 | Minetma et al. (2023){Minetama, 2023 #845} | Branched-chain amino acids plus vitamin D supplementation promote increased muscle strength following lumbar surgery for lumbar spinal stenosis: a randomized trial | Ineligible population |
| 6833 | Mirza (2016)^161^ | Surgery and physical therapy likely yield similar outcomes in spinal stenosis | Ineligible study design/publication type |
| 1630 | Mondanaro et al. (2017)^162^ | Music Therapy Increases Comfort and Reduces Pain in Patients Recovering From Spine Surgery | Ineligible population |
| 11173 | Mondanaro et al. (2017)^163^ | Music Therapy Increases Comfort and Reduces Pain in Patients Recovering From Spine Surgery | Duplicate |
| 1285 | Monticone et al. (2014)^164^ | Management of catastrophising and kinesiophobia improves rehabilitation after fusion for lumbar spondylolisthesis and stenosis. A randomised controlled trial | Ineligible population |
| 10838 | Monticone et al. (2014)^165^ | Management of catastrophising and kinesiophobia improves rehabilitation after fusion for lumbar spondylolisthesis and stenosis. A randomised controlled trial | Duplicate |
| 1707 | Monticone et al. (2017)^166^ | Responsiveness and minimal clinically important changes for the Tampa Scale of Kinesiophobia after lumbar fusion during cognitive behavioral rehabilitation | Ineligible outcome |
| 10413 | Morfeld et al. (2006)^167^ | Cognitive-behavioral therapy interventions in patients following initial surgical treatment for herniated disks and their return to work | Cannot retrieve |
| 1940 | Moussa and Khedr (2016)^168^ | Percutaneous radiofrequency facet capsule denervation as an alternative target in lumbar facet syndrome | Ineligible intervention |
| 5260 | Nair et al. (1995)^169^ | Long-term results of minimally invasive lumbar disc surgery | Ineligible intervention |
| 10921 | Neva et al. (2014)^170^ | Effect of lumbar spine fusion and postoperative exercise therapy on physical activity and kinesiophobia: a randomized controlled trial | Ineligible study design/publication type |
| 4842 | North American Spine Society Board of directors^171^ | Spine Patient Outcome Research Trial (SPORT): multi-center randomized clinical trial of surgical and non-surgical approaches to the treatment of low back pain | Ineligible study design/publication type |
| 1185 | Nykvist et al. (1995)^172^ | Severe sciatica: a 13-year follow-up of 342 patients | Ineligible outcome |
| 3126 | Oestergaard et al. (2012)^173^ | The effect of early initiation of rehabilitation after lumbar spinal fusion: a randomized clinical study | Duplicate |
| 3219 | Oestergaard et al. (2012)^174^ | The Canadian Occupational Performance Measure's semi-structured interview: its applicability to lumbar spinal fusion patients. A prospective randomized clinical study | Duplicate |
| 6237 | Oestergaard et al. (2012)^175^ | The effect of early initiation of rehabilitation after lumbar spinal fusion - A randomized clinical study. [] | Ineligible population |
| 8139 | Oestergaard et al. (2012)^176^ | The Canadian Occupational Performance Measure's semistructured interview: Its applicability to lumbar spinal fusion patients. A prospective randomized clinical study | Ineligible population |
| 11348 | Oestergaard et al. (2012)^177^ | The Canadian Occupational Performance Measure's semi-structured interview: its applicability to lumbar spinal fusion patients. A prospective randomized clinical study | Duplicate |
| 11398 | Oestergaard et al. (2012)^178^ | The effect of early initiation of rehabilitation after lumbar spinal fusion: a randomized clinical study | Duplicate |
| 2812 | Oestergaard et al. (2013)^179^ | Early versus late initiation of rehabilitation after lumbar spinal fusion: economic evaluation alongside a randomized controlled trial | Ineligible research question |
| 11408 | Oestergaard et al. (2013)^180^ | Early versus late initiation of rehabilitation after lumbar spinal fusion: economic evaluation alongside a randomized controlled trial | Duplicate |
| 10548 | Oestergaard et al. (2015)^181^ | Can a case manager reduce functional disability and absence from work for lumbar spinal fusion patients? A clinical randomized study with a two years follow-up | Ineligible study design/publication type |
| 392 | Oestergaard et al. (2020)^182^ | Does adding case management to standard rehabilitation affect functional ability, pain, or the rate of return to work after lumbar spinal fusion? A randomized controlled trial with two-year follow-up | Ineligible population |
| 1103 | Oestergaard et al. (2020)^183^ | Case manager-assisted rehabilitation for lumbar spinal fusion patients: an economic evaluation alongside a randomized controlled trial with two-year follow-up | Ineligible population |
| 1104 | Oestergaard et al. (2020)^184^ | Does adding case management to standard rehabilitation affect functional ability, pain, or the rate of return to work after lumbar spinal fusion? A randomized controlled trial with two-year follow-up | Duplicate |
| 1233 | Oestergaard et al. (2020)^185^ | Case manager–assisted rehabilitation for lumbar spinal fusion patients: an economic evaluation alongside a randomized controlled trial with two-year follow-up | Duplicate |
| 8476 | Ogon and Tuschel(2010)^186^ | Keel-based lumbar total disk replacement: Prodisc-L and Prodisc-O. [German]; Kielbasierte lumbale Bandscheibenprothesen: Prodisc-L und Prodisc-O | Ineligible intervention |
| 11025 | Oh et al. (2018)^187^ | Gender-and age-specific analgesia for early postoperative pain management after lumbar decompressive surgery: a randomized clinical trial | Ineligible study design/publication type |
| 3456 | Ohtori et al. (2011)^188^ | Surgical versus nonsurgical treatment of selected patients with discogenic low back pain: a small-sized randomized trial | Duplicate |
| 11319 | Ohtori et al. (2011)^189^ | Surgical versus nonsurgical treatment of selected patients with discogenic low back pain: a small-sized randomized trial | Ineligible population |
| 11357 | O'Neill et al. (1985)^190^ | Use of intrathecal morphine for postoperative pain relief following lumbar spine surgery | Small sample size |
| 10552 | Oosterhuis et al. (2017)^191^ | Early rehabilitation after lumbar disc surgery is not effective or cost-effective compared to no referral: a randomised trial and economic evaluation | Duplicate |
| 9581 | Ostelo et al. (2003)^192^ | Behavioral graded activity following first-time lumbar disc surgery: 1-Year results of a randomized clinical trial | Duplicate |
| 10785 | Ostelo et al. (2003)^193^ | Behavioral graded activity following first-time lumbar disc surgery: 1-year results of a randomized clinical trial | Duplicate |
| 11463 | Ostelo et al. (2003)^194^ | Effectiveness of behavioral graded activity after first-time lumbar disc surgery: short term results of a randomized controlled trial | Duplicate |
| 715 | Ostelo et al. (2004)^195^ | Economic evaluation of a behavioral-graded activity program compared to physical therapy for patients following lumbar disc surgery | Ineligible research question |
| 10790 | Ostelo et al. (2004)^196^ | Economic evaluation of a behavioral-graded activity program compared to physical therapy for patients following lumbar disc surgery | Duplicate |
| 2309 | Ozkara et al. (2015)^197^ | Chronic Opioid Therapy After Lumbar Fusion Surgery for Degenerative Disc Disease in a Workers' Compensation Setting | Small sample size |
| 4680 | Park et al. (2005)^198^ | The effects of ketorolac injected via patient controlled analgesia postoperatively on spinal fusion | Small sample size |
| 851 | Paulsen et al. (2019)^199^ | Recovery after surgery for lumbar disk herniation, a randomized clinical trial comparing the effect of supervised rehabilitation versus home exercises | Ineligible study design/publication type |
| 5701 | Paulsen et al. (2019)^200^ | Patient Reported Outcomes after Surgery for Lumbar Disk Herniation, a Randomized Controlled Trial Comparing the Effects of Referral to Municipal Physical Rehabilitation Versus no Referral | Duplicate |
| 313 | Paulsen et al. (2020)^201^ | Patient-reported Outcomes After Surgery for Lumbar Disc Herniation, a Randomized Controlled Trial Comparing the Effects of Referral to Municipal Physical Rehabilitation Versus No Referral | Duplicate |
| 421 | Paulsen et al. (2020)^202^ | Return to work after surgery for lumbar disc herniation, secondary analyses from a randomized controlled trial comparing supervised rehabilitation versus home exercises | Duplicate |
| 1085 | Paulsen et al. (2020)^203^ | Patient-reported Outcomes After Surgery for Lumbar Disc Herniation, a Randomized Controlled Trial Comparing the Effects of Referral to Municipal Physical Rehabilitation Versus No Referral | Duplicate |
| 1102 | Paulsen et al. (2020)^204^ | Return to work after surgery for lumbar disc herniation, secondary analyses from a randomized controlled trial comparing supervised rehabilitation versus home exercises | Duplicate |
| 11773 | Pekar and Steindler (2009)^205^ | Systemic oral enzyme therapy after lumbar disc herniation surgery | Duplicate |
| 2319 | Prokopovich and Parfenov (2021){Prokopovich, 2021 #2319} | Rehabilitation of patients after lumbar microdiscectomy | Small sample size |
| 11202 | Quinlan et al. (2017)^206^ | Effects of Localized Cold Therapy on Pain in Postoperative Spinal Fusion Patients: a Randomized Control Trial | Ineligible population |
| 12174 | Quinlan et al. (2017)^207^ | Effects of Localized Cold Therapy on Pain in Postoperative Spinal Fusion Patients: A Randomized Control Trial | Duplicate |
| 10718 | Radin and Bryan (1968)^208^ | Phenylbutazone for prolapsed discs? | Ineligible study design/publication type |
| 10075 | Rainov et al. (1994)^209^ | Transcutaneous electrical nerve stimulation (TENS) for acute postoperative pain after spinal surgery | Cannot retrieve |
| 10934 | Rainov et al. (1994)^210^ | Transcutaneous electrical nerve stimulation (TENS) for acute postoperative pain after spinal surgery | Duplicate |
| 4071 | Rasmussen et al. (2008)^211^ | Epidural steroid following discectomy for herniated lumbar disc reduces neurological impairment and enhances recovery: a randomized study with two-year follow-up | Ineligible intervention |
| 8806 | Rasmussen et al. (2008)^212^ | Epidural steroid following discectomy for herniated lumbar disc reduces neurological impairment and enhances recovery: A randomized study with two-year follow-up | Duplicate |
| 11822 | Reuben et al. (2006)^213^ | The effect of cyclooxygenase-2 inhibition on acute and chronic donor-site pain after spinal-fusion surgery.retraction in Neal JM. Reg Anesth Pain Med. 2009 Mar-Apr;34(2): 184; PMID: 19288610 | Ineligible study design/publication type |
| 10540 | Richter et al. (2001)^214^ | Results of applying ADCON-L gel after lumbar discectomy: the German ADCON-L study | Ineligible intervention |
| 558 | Roberts et al. (1984)^215^ | Health beliefs and rehabilitation after lumbar disc surgery | Ineligible research question |
| 2004 | Rolving et al. (2016)^216^ | Preoperative cognitive-behavioural intervention improves in-hospital mobilisation and analgesic use for lumbar spinal fusion patients | Ineligible intervention |
| 556 | Rush and Shore (1994)^217^ | Physician perceptions of the value of physical modalities in the treatment of musculoskeletal disease | Ineligible population |
| 1297 | Rushton et al. (2015)^218^ | Physiotherapy Post Lumbar Discectomy: Prospective Feasibility and Pilot Randomised Controlled Trial | Ineligible study design/publication type |
| 10842 | Rushton et al. (2015)^219^ | Physiotherapy Post Lumbar Discectomy: prospective Feasibility and Pilot Randomised Controlled Trial | Duplicate |
| 10931 | Rushton et al. (2015)^220^ | Quest physiotherapy post lumbar discectomy: pilot and feasibility study to inform the development of a randomised controlled trial | Ineligible study design/publication type |
| 1312 | Rushton et al. (2017)^221^ | Patient and physiotherapist perceptions of rehabilitation following primary lumbar discectomy: a qualitative focus group study embedded within an external pilot and feasibility trial | Ineligible population |
| 898 | Rushton et al. (2020)^222^ | The early patient journey following lumbar spinal fusion surgery: a qualitative study using semi structured interviews and weekly patient diaries | Ineligible study design/publication type |
| 2227 | Schiavolin et al (2015)^223^ | Change in quality of life, disability, and well-being after decompressive surgery: results from a longitudinal study | Small sample size |
| 2502 | Schroter et al. (2014)^224^ | [Structured rehabilitation after lumbar spine surgery : subacute treatment phase] | Small sample size |
| 11109 | Schroter et al. (2014)^225^ | Structured rehabilitation after lumbar spine surgery : subacute treatment phase | Duplicate |
| 750 | Scrimshaw and Maher (2001)^226^ | Randomized controlled trial of neural mobilization after spinal surgery | Ineligible population |
| 10774 | Scrimshaw and Maher (2001)^227^ | Randomized controlled trial of neural mobilization after spinal surgery | Duplicate |
| 11159 | Sekiguchi et al. (2015)^228^ | Loxoprofen sodium and celecoxib for postoperative pain in patients after spinal surgery: a randomized comparative study | Ineligible research question |
| 9134 | Selkowitz et al. (2006)^229^ | The immediate and long-term effects of exercise and patient education on physical, functional, and quality-of-life outcome measures after single-level lumbar microdiscectomy: A randomized controlled trial protocol | Ineligible study design/publication type |
| 4663 | Shabat et al. (2005)^230^ | Gender differences as an influence on patients' satisfaction rates in spinal surgery of elderly patients | Ineligible intervention |
| 4010 | Shatrova and Meskhi (2008)^231^ | [Mexidol in the post-surgery rehabilitation of patients with degenerative-dystrophic diseases of the spine] | Cannot retrieve |
| 7263 | Shimia et al. (2015)^232^ | Effect of body mass index on inpatient rehabilitation outcomes after single-level discectomy | Ineligible research question |
| 2320 | Shin et al. (2015)^233^ | Epidural Steroids After a Percutaneous Endoscopic Lumbar Discectomy | Ineligible intervention |
| 11105 | Shin et al. (2015)^234^ | Epidural Steroids After a Percutaneous Endoscopic Lumbar Discectomy | Duplicate |
| 8479 | Siepe et al. (2010)^235^ | Treatment of dynamic spinal canal stenosis with an interspinous spacer. [German]; Die Behandlung der dynamischen Spinalkanalstenose mit einem interspinosen Spacer | Ineligible intervention |
| 11610 | Skall et al. (1994)^236^ | Intensive back exercises 5 weeks after surgery of lumbar disk prolapse. A prospective, randomized multicenter trial with a historical control group | Duplicate |
| 3409 | Skidmore et al. (2011)^237^ | Cost-effectiveness of the X-STOP interspinous spacer for lumbar spinal stenosis | Ineligible research question |
| 2356 | Skolasky et al. (2015)^238^ | Health behavior change counseling in surgery for degenerative lumbar spinal stenosis. Part I: improvement in rehabilitation engagement and functional outcomes | Ineligible population |
| 10888 | Skolasky et al. (2015)^239^ | Health behavior change counseling in surgery for degenerative lumbar spinal stenosis. Part I: improvement in rehabilitation engagement and functional outcomes | Duplicate |
| 10889 | Skolasky et al. (2015)^240^ | Health behavior change counseling in surgery for degenerative lumbar spinal stenosis. Part II: patient activation mediates the effects of health behavior change counseling on rehabilitation engagement | Duplicate |
| 12229 | Skolasky et al. (2015)^241^ | Health Behavior Change Counseling in Surgery for Degenerative Lumbar Spinal Stenosis. Part I: Improvement in Rehabilitation Engagement and Functional Outcomes | Duplicate |
| 12230 | Skolasky et al. (2015)^242^ | Health Behavior Change Counseling in Surgery for Degenerative Lumbar Spinal Stenosis. Part II: Patient Activation Mediates the Effects of Health Behavior Change Counseling on Rehabilitation Engagement | Ineligible population |
| 305 | Skolasky et al. (2018)^243^ | Telephone-Based Intervention to Improve Rehabilitation Engagement After Spinal Stenosis Surgery: A Prospective Lagged Controlled Trial | Ineligible intervention |
| 11673 | Skolasky et al. (2018)^244^ | Telephone-Based Intervention to Improve Rehabilitation Engagement After Spinal Stenosis Surgery: a Prospective Lagged Controlled Trial | Duplicate |
| 12918 | Skolasky et al. (2018)^245^ | Telephone-Based Intervention to Improve Rehabilitation Engagement After Spinal Stenosis Surgery: A Prospective Lagged Controlled Trial | Duplicate |
| 4599 | Soegaard et al. (2006)^246^ | Lumbar spinal fusion patients' demands to the primary health sector: evaluation of three rehabilitation protocols. A prospective randomized study | Ineligible intervention |
| 11501 | Soegaard et al. (2006)^247^ | Lumbar spinal fusion patients' demands to the primary health sector: evaluation of three rehabilitation protocols. A prospective randomized study | Duplicate |
| 11082 | Sogaard et al. (2008)^248^ | Cost-effectiveness evaluation of an RCT in rehabilitation after lumbar spinal fusion: a low-cost, behavioural approach is cost-effective over individual exercise therapy | Ineligible research question |
| 11729 | Sogaard et al. (2008)^249^ | Cost-effectiveness evaluation of an RCT in rehabilitation after lumbar spinal fusion: a low-cost// behavioural approach is cost-effective over individual exercise therapy | Duplicate |
| 8555 | Sokunbi et al. (2010)^250^ | Experiences of individuals with chronic low back pain during and after their participation in a spinal stabilisation exercise programme - A pilot qualitative study | Ineligible population |
| 8164 | standaert (2011)^251^ | Core Stabilization for Low Back Pain and Performance | Ineligible study design/publication type |
| 3165 | Steib et al. (2012)^252^ | Predictors of facet joint syndrome after lumbar disc surgery | Ineligible research question |
| 6602 | Storheim et al. (2017)^253^ | Fat in the lumbar multifidus muscles - predictive value and change following disc prosthesis surgery and multidisciplinary rehabilitation in patients with chronic low back pain and degenerative disc: 2-year follow-up of a randomized trial | Ineligible population |
| 11021 | Strom et al. (2018)^254^ | Does a web-based spine platform featuring social interaction and animated information affect patient reported outcomes in patients undertaking lumbar spine fusion surgery? a randomized clinical trial | Ineligible study design/publication type |
| 11033 | Strom et al. (2018)^255^ | A web-based platform to accommodate symptoms of anxiety and depression by featuring social interaction and animated information in patients undergoing lumbar spine fusion: a randomized clinical trial | Duplicate |
| 541 | Strom et al. (2019)^256^ | Lumbar Spine Fusion Patients' Use of an Internet Support Group: Mixed Methods Study | Ineligible intervention |
| 659 | Strom et al. (2019)^257^ | A web-based platform to accommodate symptoms of anxiety and depression by featuring social interaction and animated information in patients undergoing lumbar spine fusion: a randomized clinical trial | Ineligible intervention |
| 10728 | Strom et al. (2019)^258^ | Lumbar Spine Fusion Patients' Use of an Internet Support Group: mixed Methods Study | Duplicate |
| 11930 | Svensson et al. (2013)^259^ | Patients' experience of health three years after structured physiotherapy or surgery for lumbar disc herniation | Ineligible population |
| 11259 | Tait et al. (2009)^260^ | Improved outcome after lumbar microdiscectomy in patients shown their excised disc fragments: a prospective, double blind, randomised, controlled trial | Ineligible population |
| 10922 | Tamburrrelli et al. (2014)^261^ | Efficacy of "NEURAC" post-operative rehabilitation method in patients who underwent microdiscectomy | Ineligible study design/publication type |
| 7285 | Techy and Benzel (2015)^262^ | Implementing an outpatient ambulatory discectomy protocol at a large academic center: A change for the better | Ineligible study design/publication type |
| 1028 | Terai et al. (2022){He, 2021 #90} | Postoperative Physical Therapy Program Focused on Low Back Pain Can Improve Treatment Satisfaction after Minimally Invasive Lumbar Decompression | Ineligible population |
| 2053 | Than et al. (2016)^263^ | How to predict return to work after lumbar discectomy: answers from the NeuroPoint-SD registry | Ineligible outcome |
| 5347 | Timm (1994)^264^ | A randomized-control study of active and passive treatments for chronic low back pain following L5 laminectomy | Ineligible intervention |
| 11612 | Timm (1994)^265^ | A randomized-control study of active and passive treatments for chronic low back pain following L5 laminectomy | Duplicate |
| 1044 | Uhrrand et al. (2023){Uhrbrand, 2023 #1044} | An individualised tapering protocol reduces opioid use 1 year after spine surgery: A randomised controlled trial of patients with preoperative opioid use | Ineligible population |
| 134 | Urban et al. (2018)^266^ | Pregabalin Did Not Improve Pain Management After Spinal Fusions | Ineligible intervention |
| 5140 | Vaccaro and Ball (2000)^267^ | Indications for instrumentation in degenerative lumbar spinal disorders | Ineligible study design/publication type |
| 12313 | Vaid et al. (2016)^268^ | Low-Dose Ketamine Infusions for Highly Opioid-Tolerant Adults Following Spinal Surgery: A Retrospective Before-and-after Study | Ineligible population |
| 3660 | Volchegorskii and Mester (2010)^269^ | [Effects of 3-hydroxypyridine and succinic acid derivatives on the dynamics of dorsalgia and affective disorders after surgical treatment of disc herniation] | Ineligible population |
| 11282 | Volchegorskii and Mester (2010)^270^ | Effects of 3-hydroxypyridine and succinic acid derivatives on the dynamics of dorsalgia and affective disorders after surgical treatment of disc herniation | Duplicate |
| 8569 | Volchegorskii et al. (2010)^271^ | Effects of 3-hydroxypyridine and succinic acid derivatives on the dynamics of dorsalgia and affective disorders after surgical treatment of disc herniation. [Russian] | Duplicate |
| 1069 | Wang et al. (2022){Wang, 2022 #1069} | Randomized controlled trial of overall functional exercise process in perioperative of percutaneous transforaminal endoscopic discectomy | Ineligible intervention |
| 1532 | Wang et al. (2022){Wang, 2022 #1069} | Randomized controlled trial of overall functional exercise process in perioperative of percutaneous transforaminal endoscopic discectomy | Duplicate |
| 11635 | Wang and Tronnier (2000)^272^ | Effect of acupuncture on pain management in patients before and after lumbar disc protrusion surgery--a randomized control study | Ineligible intervention |
| 10723 | Watters et al. (1989)^273^ | The use of dexamethasone in primary lumbar disc surgery. A prospective, randomized, double-blind study | Small sample size |
| 8424 | Weinstein et al. (2009)^274^ | Surgical compared with nonoperative treatment for lumbar degenerative spondylolisthesis: Four-year results in the Spine Patient Outcomes Research Trial (SPORT) randomized and observational cohorts | Ineligible research question |
| 5207 | White et al. (1998)^275^ | An evaluation of the effectiveness of patient-controlled analgesia after spinal surgery | Ineligible population |
| 4086 | Williamson et al. (2008)^276^ | What do patients feel they can do following lumbar microdiscectomy? A qualitative study | Ineligible population |
| 1086 | Willems et al. (2023) | Variability in recovery following microdiscectomy and postoperative physiotherapy for lumbar radiculopathy: A latent class trajectory analysis | Ineligible study design/publication type |
| 220 | Wirth et al. (2019)^277^ | An observational study on trajectories and outcomes of chronic low back pain patients referred from a spine surgery division for chiropractic treatment | Ineligible population |
| 88 | Witttayapairoj et al. (2023){Wittayapairoj, 2023 #88} | Effect of bilateral ultrasound-guided erector spinae plane block on postoperative pain after open lumbar spinal surgery: a double-blind, randomized controlled trial | Ineligible outcome |
| 11229 | Wu et al. (2017)^278^ | Percutaneous endoscopic lumbar discectomy combined with epidural injection for prolapsed lumbar disc herniation | Ineligible intervention |
| 339 | Wu and Ye (2021){Wu, 2021 #339} | The Effect of Lower-Limb Exercise on Pain Management of the Patients Undergoing Posterior Lumbar Fusion Surgery: A Retrospective Case-Control Study | Ineligible population |
| 1165 | Yaman et al. (2020)^279^ | Cold Therapy and the Effect on Pain and Physiological Parameters in Patients Recovering from Spine Surgery: a Randomized Prospective Study | Ineligible outcome |
| 721 | Yao et al. (2018)^280^ | Bracing Following Transforaminal Lumbar Interbody Fusion is not Necessary for Patients With Degenerative Lumbar Spine Disease: A Prospective, Randomized Trial | Ineligible population |
| 11015 | Yao et al. (2018)^281^ | Bracing Following Transforaminal Lumbar Interbody Fusion is not Necessary for Patients with Degenerative Lumbar Spine Disease | Duplicate |
| 4050 | Yee et al. (2008)^282^ | Use of a postoperative lumbar corset after lumbar spinal arthrodesis for degenerative conditions of the spine. A prospective randomized trial | Ineligible population |
| 11570 | Yee et al. (2008)^283^ | Use of a postoperative lumbar corset after lumbar spinal arthrodesis for degenerative conditions of the spine. A prospective randomized trial | Duplicate |
| 12953 | Yee et al. (2008)^284^ | Use of a Postoperative Lumbar Corset After Lumbar Spinal Arthrodesis for Degenerative Conditions of the Spine | Duplicate |
| 11333 | Yeh et al. (2011)^285^ | Pain reduction of acupoint electrical stimulation for patients with spinal surgery: a placebo-controlled study | Ineligible population |
| 3440 | Yeh et al. (2011)^286^ | Pain reduction of acupoint electrical stimulation for patients with spinal surgery: a placebo-controlled study | Duplicate |
| 11156 | Zarei et al. (2016)^287^ | Management of postoperative pain after Lumbar surgery-pregabalin for one day and 14 days-a randomized, triple-blinded, placebo-controlled study | Duplicate |
| 723 | Zhang et al. (2018)^288^ | Postoperative functional exercise for patients who underwent percutaneous transforaminal endoscopic discectomy for lumbar disc herniation | Duplicate |
| 11656 | Zhang et al. (2018)^289^ | Postoperative functional exercise for patients who underwent percutaneous transforaminal endoscopic discectomy for lumbar disc herniation | Duplicate |
| 5658 | Zhang et al. (2019)^290^ | Pestle needling at Yaoyangguan-Bazhen points for intractable lumbodynia after lumbar disc herniation surgery: A randomized controlled trial | Ineligible population |
| 1725 | Zhang et al. (2022){Zhang, 2022 #1725} | Clinical application of enhanced recovery after surgery in lumbar disk herniation patients undergoing dynamic stabilization and discectomy | Small sample size |
| 1364 | Zhang et al. (2021){Zhang, 2021 #1364} | Addition of Celebrex and Pregabalin to Ropivacaine for Posterior Spinal Surgery: A Randomized, Double-Blinded, Placebo-Controlled Trial | Ineligible outcome |
| 11595 | Zhao et al. (2008)^291^ | Clinical effects of acupuncture after surgical operation in patients with prolapse of the lumbar intervertebral disc | Duplicate |
| 1540 | Zheng et al. (2021){Zheng, 2021 #1540} | Effects of acupoint massage combined with relaxation therapy on patients with postoperative fatigue syndrome after lumbar surgery | Ineligible study design/publication type |
| 1169 | Zhong et al. (2023){Zhong, 2023 #1169} | [Effect of acupotomy on the fat infiltration degree of lumbar multifidus muscle in patients with lumbar disc herniation after percutaneous transforaminal endoscopic discectomy] | Ineligible intervention |
| 10899 | Zhu et al. (2016)^292^ | Therapeutic observation of qi-guiding needling plus electroacupuncture for intractable low back pain after lumbar disc herniation surgery and the change of infrared thermal image | Cannot retrieve |
| 3463 | Zieger et al. (2011)^293^ | The impact of psychiatric comorbidity on the return to work in patients undergoing herniated disc surgery | Ineligible intervention |
| 12937 | Zieger et al. (2011)^293^ | The Impact of Psychiatric Comorbidity on the Return to Work in Patients Undergoing Herniated Disc Surgery | Duplicate |
| 286 | Zoia et al. (2018)^294^ | Usefulness of corset adoption after single-level lumbar discectomy: a randomized controlled trial | Small sample size |
| 1060 | No name (2021)^295^ | Effect of Feedback Health Education on Postoperative Rehabilitation of Patients With Lumbar Disc Herniation: a Cluster Randomized Trials | Ineligible study design/publication type |
| 1158 | No name (2020)^296^ | Application of rehabilitation nursing in patients accepting minimally invasive spine surgery and its effects on pain and adverse emotions | Ineligible population |
| 10481 | No name (2018)^297^ | Dietary supplementation in patients following lumbar spine surgery: a randomized controlled trial | Ineligible study design/publication type |
| 10482 | No name (2017)^298^ | Multimodal Nutritional Management in Primary Lumbar Spinal surgery: a Randomized Controlled Trial | Ineligible intervention |
| 10486 | No name (2018)^299^ | Study of the effect of gentle stretching exercises and foot reflexology massage on the pain of patients after spinal surgery | Ineligible study design/publication type |
| 10516 | No name (2018)^300^ | The Use of Yoga for Lumbar Spine Post-Surgical Pain Management | Ineligible study design/publication type |
| 10520 | No name (2019)^301^ | A home video exercise program improves ADL/QOL and kinesiophobia in the patients with lumbar spine surgery A randomized controlled trial | Ineligible study design/publication type |
| 10522 | No name (2019)^302^ | The effect of pain management education on severity of pain, anxiety and disability after lumbar surgery in patients with chronic low back pain | Ineligible study design/publication type |
| 10526 | No name (2019)^303^ | The Effect of Ultrasound-guided Erector Spinae Plane Block (ESPB) on Perioperative Analgesia and Rehabilitation for Posterior Lumbar Interbody Fusion: a Prospective Randomized Controlled Study | Ineligible study design/publication type |
| 10560 | No name (2013)^304^ | Physiotherapy Following Disc Surgery: long Term Follow-up of a RCT | Ineligible study design/publication type |
| 10579 | No name (2007)^305^ | Assessment of Low Level Laser Therapy for Late Postoperative Pain After Lumbar Fusion Surgery | Ineligible study design/publication type |
| 10580 | No name (2007)^306^ | Preoperative Prevention and Early Rehabilitation for Patients Undergoing Elective Spine Surgery | Ineligible study design/publication type |
| 10590 | No name (2018)^307^ | Effect of a Case Manager to Assist the Rehabilitation for Lumbar Spinal Fusion Patients. A Randomised Controlled Trial | Ineligible study design/publication type |
| 10656 | No name (2017)^308^ | Transcranial Direct Current Stimulation in Reduction of Pain and Postoperative Opioids Consumption After Spine Surgery | Ineligible study design/publication type |
| 10698 | No name (2013)^309^ | A Randomized,controlled, multicentre Clinical Study to Evaluate the Efficacy and Safety of Smartphone in post-operative rehabilitation after lumbar spine surgeries | Ineligible study design/publication type |
| 10707 | No name (2016)^310^ | Lumbar microdiscectomy and post-operative activity restrictions | Ineligible study design/publication type |
| 1644 | No name (2021){, 2021 #1644} | Effect of Feedback Health Education on Postoperative Rehabilitation of Patients With Lumbar Disc Herniation: a Cluster Randomized Trials | Ineligible intervention |
| 1499 | No name (2022){, 2022 #1499} | The effects of auricular acupressure on low back pain, neuropathy and sleep in patients with lumbar spine surgery | Ineligible intervention |
| 1370 | No name (2023){, 2023 #1370} | The Necessity and Timing of Exercise After Lumbar Disc Herniation Surgery: a Randomized Controlled Trial | Duplicate |

**Additional file 7: Characteristics of studies**

| **First Author, Country** | **Year** | **Total patients randomized** | **Clinical condition** | **Surgery** | **Age (years)** | | **Female sex**  **No, (%)** | **Interventions** | |
| --- | --- | --- | --- | --- | --- | --- | --- | --- | --- |
|  |  |  |  |  | **Mean/ median** | **SD/ range** |  | **Intervention** | **Comparison** |
| Abdi et al. (2023)^311^  (Iran) | 2023 | 90 | Lumbar disc herniation | Single-level lumbar laminectomy and discectomy | I1: 44.20  I2: 44.95  C: 42.90 | SD not provided by group | I1: 13 (30.2%)  I2: 15 (34.9%)  C: 15 (34.1%) | **Type 1:** Williams group: exercises including pelvic tilt, single knee to chest, double knee to chest, partial sit-up, hamstring stretch, hip flexor stretch, and squat.  **Type 2:** McKenzie group: exercises including lying prone, prone on elbows, standing extension, lying flexion, sitting flexion, and standing flexion.  **Initiation postsurgery:** end of the 6^th^ week postsurgery  **Duration:** 8 weeks  **Frequency:** 45 minutes/session, 3x/week  **Setting:** home program | **Other intervention:** conventional postoperative rehabilitation including early walking and physiotherapy |
| Aldemir et al. (2021)^312^  (Turkey) | 2021 | 80 | Lumbar disc herniation | Standard lumbar discectomy using a microsurgical technique | I: 42.3  C: 44.88 | 9.92  9.25 | I: 16 (48.5%)  C: 18 (52.9%) | **Type:** Walking exercise with a minimum of 10-minute walking (4 days/week) and continuing with a minimum of 30-minute moderate intensity walking for a minimum of 5 days a week.  **Initiation postsurgery:** the 3rd post-operative week  **Duration:** 3 months  **Frequency:** 4-5x/week  **Setting:** community | No intervention |
| Beneck et al. (2014)^313^  (USA) | 2014 | 98 | Disc protrusion confirmed by MRI, predominant symptoms in the lower extremity, radicular pain distribution, restricted straight leg raise, and positive signs of adverse nerve-root tension (i.e., impaired mobility and/or pain and/or dysesthesia) | Single level microdiskectomy | I: 39.2  C: 41.4 | 10.2  9.9 | I: 22 (43%)  C: 23 (49%) | **Type:** exercise by PT. Back extensor strength and endurance training, trunk and lower extremity exercise training; two 1-hour back care one-on-one education sessions  **Initiation postsurgery:** 4-6 weeks  **Duration:** 12 weeks  **Frequency:** 3x/week  **Setting:** PT clinics (community) | **Other intervention:** Education by PT (same as intervention group) |
| Bono et al. (2017)^314^  (USA) | 2017 | 112 | Predominant radicular leg pain clinically correlated to a single-level, central or posterolateral lumbar disk herniation by advanced imaging study  Excluded: people with recurrent disk herniation, prior lumbar spine surgery, extraforaminal (far lateral) herniations, disk herniation with primarily back pain and mild/no radicular pain | Standard open surgical approach with the assistance of loupe or microscope magnification per surgeon preference | I: 42  C: 44.6 | 12.5  9.4 | I: 25 (47%)  C: 21 (38%) | **Type:** education/self-management by surgeon: short (2 week) postsurgical restriction avoiding forward bending at waist (excluding bending to sit), lifting more than 5–10lb, or twisting. No formal rehabilitation.  **Setting:** hospital | **Other intervention:** Long (6 week) post-surgical restriction by surgeon: avoiding forward bending at waist (excluding bending to sit), lifting more than 5–10lb, or twisting. No formal rehabilitation.  **Setting:** hospital |
| Choi et al. (2005)^315^  (Korea) | 2005 | 80 | Unilateral radiating leg pain with or without back pain, not responding to conservative treatment methods and having good radiologic correlation; first-time lumbar spine operation; single-level lumbar disc herniation | Lumbar microdiscectomy or percutaneous endoscopic discectomy | I: 51.0  C: 42.0 | 9.6  17.1 | I: 15 (43%)  C: 22 (55%) | **Type:** exercise: supervised and graded aerobic and limb-strengthening exercises, dynamic and isometric exercises for lumbar extensors. MedX system used to restrict hip and pelvic motion and isolate lumbar extensor muscles.  **Initiation postsurgery:** NR  **Duration:** 12 weeks  **Setting:** assume rehab clinic (community) | **Other intervention:** Home-based basic lumbar conditioning exercises (12 weeks)  **Setting:** hospital and home |
| Erdogan and Bulut (2020)^316^  (Turkey) | 2019 | 62 | Lumbar disc herniation | First-time lumbar disc herniation surgery (surgery type not specified) | I: 40  C: 40.6 | 10.9  11.4 | I: 14 (45.2%)  C: 13 (41.9%) | **Type:** education: web-based information (including answers to questions) about recovery period at home  **Initiation postsurgery:** 1 day  **Setting:** hospital | **Other intervention:** Patient-training guidebook: information about recovery period at home following discharge 1 day postsurgery. Questions were answered during telephone interviews.  **Setting:** hospital |
| Erdogmus et al. (2007)^317^  (Austria) | 2007 | 120 | Lumbar disc herniation with lumbar radicular syndrome < 6 months  Excluded: local complications in their operation area (such as discitis, local septic inflammation, etc.), postoperative muscle weakness in the legs (exceeding a force Grade 2/5), a sum score on the LBP rating scale exceeding 100 points, spinal stenosis, spondylolisthesis. | First-time uncomplicated disc surgery for lumbar disc herniation (standard laminectomy & discectomy/ micro-discectomy) | I: 39.8  Sham: 42.3  Wait and see:  41.8 | 10.5  9.8  10.4 | I: 19 (47.5%)  C: 19 (47.5%) | **Type:** Multimodal care (without psychological component) by PT. Tailored instructions: 1) early phase (education and exercise); 2) intermediate phase (exercises to achieve pain free spine mobility); 3) late phase (restoration of physiological movement patterns, patient reintegration)  **Initiation postsurgery:** 1 week  **Duration:** 12 weeks  **Frequency:** 20 30-min sessions  **Setting:** clinic | **Sham:** neck massage (20 30-min sessions over 12 weeks) by registered massage therapist  **Setting:** assume outpatient clinic  **Control:** watchful waiting |
| Ebenbichler et al. (2015)^318^  (Austria) | 2015 |  |  |  |  |  |  |  |  |
| Fu and Ji (2017)^319^  (China) | 2017 | 80 | Lumbar disc prolapse | Surgery type not specified | I: 58.4  C: 59.6 | 12.3  12.4 | I: 14 (35%)  C: 15 (38%) | **Type:** Traditional Chinese Medicine + routine care by nurses: 1) emotional care, Management of diet and lifestyle: frequent; 2) fumigation care: starting from the second post-surgery day, 1 time/day for 30minutes; 3) rehabilitation exercise: mild exercise 2-3 hours after the surgery, wearing waist protection for 1-2 weeks; then other moderate exercise: 2times/day, 30minutes/time; 4) acupressure: 1 time/day, 2-3 minutes/acupressure-point; and 5) routine nursing same as for the control group | Routine nursing by nurses: vital signs nursing, infection prevention, anti-inflammation, nourishing nerves, a light and easily digested diet (avoid spicy food), pain management, and discharge guidance. |
| Hakkinen et al. (2005)^320^  (Finland) | 2005 | 126 | Lumbar disk herniation:  Chronic unbearable back pain due to prolapsed disk, with or without muscle weakness, radiating down to lower extremity, not relieved by conservative treatment. Diagnosis based on preoperative clinical status, detected spinal nerve root compression in MRI or CT, confirmed during surgery. | First time lumbar disk surgery using  the open mini approach | I: 39  C: 39 | 7  8 | I: 29 (44.6%)  C: 26 (42.6%) | **Type:** home strength training and stretching exercise by PT: 1) strengthening exercises using body weight or adjustable dumbbells (2 sets of 8-12 repetitions for each exercise, twice weekly); 2) stretching exercises (3 repetitions for 30 seconds for each exercise, 3 times weekly); 3) stabilization exercises (5 -10 repetitions daily): isometric contractions of transversus abdominis muscle. Encouraged to do aerobic training 2-3x/week.  **Initiation postsurgery:** 2 months  **Duration:** 12 months (home program)  **Frequency:** 1 session to practice each exercise prior to home program  **Setting:** hospital and home | **Other intervention**: stretching alone stretching exercise (beginning 2 months postsurgery and home program for 12 months) by PT: 1) Stretching exercises (3 repetitions for 30 seconds for each exercise, 3 times weekly); 2) isometric contractions (5-10 repetitions daily). Each exercise was practiced under supervision for 1 session prior to home training. Encouraged to do aerobic training 2-3x/wk.  **Setting:** hospital and home |
| He et al. (2021)^321^  (China) | 2021 | 95 | Lumbar disc herniation | First time lumbar disc herniation surgery (surgery type not specified) | I: 46.01  C: 45.88 | 5.12  4.99 | I: 22 (46.8%)  C: 21 (43.7%) | **Type:** continuous nursing based on wechat platform to deliver texts, pictures and videos regarding functional exercises provided in the wechat group every day. The members of the group included doctors, nurses, patients and patients’ families to provide support and supervision + routine continuous nursing (same as comparison).  **Initiation postsurgery:** post-surgery  **Duration:** 3 months  **Frequency:** continuous care  **Setting:** community/home | **Type:** routine continuous nursing: pre-discharge education, including medication use, diet guidance, rehabilitation training methods, daily life precautions, management of lumbar and leg pain, the time for further consultation. Telephone follow-ups once every 2 weeks.  **Initiation postsurgery:** post-surgery  **Frequency:** once every 2 weeks  **Duration:** 3 months  **Setting:** community/home |
| Jentoft et al. (2020)^322^  (Norway) | 2020 | 80 | Lumbar disc prolapse | First time lumbar discectomy | I: 40.2  C: 39.4 | 10.2  10.3 | I: 12 (32.4%)  C: 14 (42.4%) | **Type:** exercise provided by physiotherapists: 1) before discharge (10-30 minutes/session, 2-6 sessions): 7 exercises, 8-10 repetitions, twice daily to regain mobility and strength, and improve coordination; 2) after discharge: continuing exercises and increasing the number of repetitions and sets gradually. Same information as control group.  **Initiation postsurgery:** first day after surgery  **Frequency:** 2-6 treatments lasting 10-30 minutes  **Setting:** hospital/community/home | **Type:** information (standard preoperative information and postoperative advice regarding pain, activity and recovery expectations)  **Initiation postsurgery:** first day after surgery  **Frequency:** as necessary  **Setting:** hospital |
| Kulikov et al. (2018)^323^  (Russia) | 2018 | 73 | Lumbar disc herniation treated by surgery within one month [17.1 (8.16) days]* with pain or discomfort in lumbar area | Lumbar discectomy | I: 39.9  C: 45.2 | 0.1  12.7 | I: 11 (29.7%)  C: 9 (25%) | **Type:** passive physical modality: whole-body magnetic field (WBMF) therapy + other intervention (same as comparison)  **Initiation postsurgery:** within 1 month  **Frequency:** 10 sessions  **Setting:** hospital | **Other intervention:** (exercise + pharmacological + aquatic therapy): nonsteroidal anti-inflammatory and antispastic drugs, glucocorticoids, nonnarcotic analgesics, therapeutic exercise (10 sessions), aquatic therapy (10 sessions)  **Setting:** hospital |
| Lu and Bai (2020)^324^    (China) | 2020 | 62 | Lumbar disc herniation with lower extremity symptoms | Percutaneous transforaminal endoscopic discectomy | I: 48.5  C: 50.2 | 16.5  14.0 | I: 13 (43.3%)  C: 14 (46.7%) | **Type:** remote staged rehabilitation exercise based on McKenzie’s technology: 2-6 weeks after operation as the first stage (wearing lumbar support device, extremity stretching and strengthening exercise, resuming non-physical demanding activities of daily living), 7-12 weeks as the second stage (resuming activities of daily activities, lumbar muscle strengthening exercise with support of lumbar support device), and 13-24 weeks as the third stage (removing lumbar support device, lumbar and core muscle strengthening exercise, resuming activities of daily living).  **Initiation postsurgery:** post-surgery  **Duration:** 24 weeks  **Frequency:** remote guidance  **Setting:** hospital/community/home | **Type:** routine post-surgical care focusing on lumbar muscle exercise without staging. |
| Manniche et al. (1993)^325^  (Denmark) | 1993 | 96 | Back pain with radiculopathy due to herniated lumbar intervertebral discs | First-time lumbar discectomy | Sample size not provided by group | Sample size not provided by group | 49 (51%) | **Type:** intensive exercise: training session: 5 heavy exercises, 50 repetitions regarding of pain during exercise in classes of 2-6 patients; 6 1-hour sessions over first 3 weeks; 6 30-minute sessions over 2nd 3 weeks.  **Initiation postsurgery**: 4-5 weeks  **Duration:** 6 weeks  **Frequency:** 14 hours in 12 sessions  **Setting:** class | **Type:** mild exercise: training session: 15 mild exercises, 10 repetitions each in classes of 2-6 patients; 6 30-minute sessions over first 3 weeks; 6 1-hour sessions over 2nd 3 weeks.  **Initiation postsurgery**: 4-5 weeks  **Duration:** 6 weeks  **Frequency:** 14 hours in 12 sessions  **Setting:** class |
| Oosterhuis et al. (2017)^326^  (Netherlands) | 2017 | 184 | Lumbosacral radicular syndrome/sciatica (lumbar disc herniation confirmed by MRI, signs of nerve root compression corresponding to herniation level)  Excluded: people with neurogenic claudication, spinal surgery in prior 12 months, previous lumbar disc surgery at the same level and on the same side | Lumbar disc surgery (surgery type not specified) | 47 | 12 | I: 54 (59%)  C: 44 (57%) | **Type:** multimodal care (without psychological component) by PT: individual in-person exercise (PT’s discretion), tailored advice on lifestyle and ADLs plus usual post-surgical care  **Initiation postsurgery:** 1^st^ week after discharge (1-2 days hospitalization)  **Duration:** 6-8 weeks  **Frequency:** 1-2x/week (30-min sessions)  **Setting:** clinic | **Type:** Usual post-surgical care during hospitalization: 1-2 sessions of education by physiotherapist or nurse and booklet at discharge providing advice (mainly ADLs); suggestions for exercises (muscle strengthening, core stability, mobilization)  **Setting:** hospital |
| Ostelo et al. (2003, 2003)^327, 328^  (Netherlands) | 2003 | 105 | Radicular leg pain with conclusive findings on MRI/radiography; complaints (e.g., residual leg or back pain) restricting ADL and/or work 6 weeks post-surgery  Excluded: stenosis | First-time lumbar disc surgery (one level only) (74% participants receiving lumbar discectomy, 23% participants receiving discectomy in combination with a partial foraminectomy) | I: 42.8  C: 43.7 | 8.8  8.8 | I: 26 (50%)  C: 19 (35.8%) | **Type:** psychological by PT: BGA: exercises based on individual treatment goals, progressed to setting quotas, systematically increased towards the pre-set goal, positive reinforcement to increase healthy behaviors and decrease pain behaviors.  **Initiation postsurgery:** NR  **Duration:** 3 months  **Frequency and dose:** max. 18 30-min sessions  **Setting:** NR | **Other intervention:** Usual care by PT: (max. 18 30-min sessions within 3 months): whole spectrum of techniques used by PTs. Not allowed: specific BGA components, acupuncture, osteopathic techniques, other “alternative” techniques  **Setting**: NR |
| Paulsen et al. (2019, 2020)^329, 330^  (Denmark) | 2019  2020 | 146 | Leg pain with/without back pain of various duration with MRI confirmed single-level symptomatic lumbar disc herniation  Exclusion: previous spine surgery | Open lumbar discectomy with or without the use of microscope and tubular-discectomy  depending on the surgeon’s preference | I: 42.9  C: 42.8 | 8.9  11.8 | I: 27 (37%)  C: 27 (37%) | **Type:** exercise (spinal stability) by PT + usual care  **Initiation postsurgery:** 4-6 weeks **Duration:** 8-10 weeks  **Frequency:** 1-2 x/week (team and individual training)  **Setting:** assume PT clinic (community) | **Usual care:** briefing session at discharge by physiotherapist (on the expected  postoperative course and instructed to perform standard exercises the first 4 weeks postoperatively; educational booklet; and outpatient clinic visit with a physiotherapist 1 month post-surgery) and advice to begin normal daily activities and work as soon as possible  **Setting:** assume hospital |
| Pekar and Steindler (2010)^331^  (Prague) | 2009 | 100 | Elective surgery of simple lumbar intervertebral disc herniation in the L4/5 or L5/S1 space  Excluded: history of surgery in same or different segment of spine | Lumbar disc herniation surgery (surgery type not specified) | 42 | 9.06 | I: 24 (48%)  C: 24 (48%) | **Type:** systemic enzyme therapy: systemic enzyme agent with active substances of two proteolytic enzymes bromelain 90 mg and trypsin 48 mg supplemented with flavonoid rutin 100 mg. In addition, participants received routine postoperative treatment including combinations of NSAID and opioid analgesics and completed rehabilitation  **Initiation postsurgery:** evening after surgery  **Duration:** 50 days  **Frequency and dose:** 5 tablets in the evening after surgery, then 5 tablets twice a day for 50 days  **Setting:** hospital | **Usual care:** routine postoperative treatment including combinations of NSAID and opioid analgesics and completed rehabilitation. |
| Skall et al. (1994)^332^  (Denmark) | 1994 | 159 | Lumbar disc prolapse  Excluded: people with spondylolisthesis, previous lumbar spine surgeries | First-time lumbar discectomy | I: 44  C: 45 | 10/90 percentile = 31/60  10/90 percentile = 33/64 | I: 49 (51%)  C: 31 (49%) | **Type 1:** exercise (intensive back exercise: 5 strength + 5 endurance exercises executed in dynamic tempo, repeated 50x in series of 10, without regard for eventual local back pain); education about back’s possible movements to get back to an active life  **Type 2:** exercise (mild back exercise: 15 mild, mobilizing exercises, repeated 10x, followed by 5 stretching exercises; stop training in case of pain); Swedish back school about body  **Initiation postsurgery:** 5 weeks  **Duration:** 14 hours during 6 weeks  **Setting:** hospital and assume rehab clinic (community) | **Usual care:** varied rehabilitation treatment  **Setting:** hospital and assume rehab clinic (community) |
| Uysal et al. (2023)^333^  (Turkey) | 2023 | 204 | Radicular pain that persisted despite 4-6 weeks of conservative care and MRI-confirmed disc herniation | Single-level unilateral lumbar microsurgery | I1: 46.3  I2: 48.5  I3: 48.3  I4: 48.7  C: 48.1 | I1: 11.3  I2: 11.2  I3: 10.7  I4: 10.8  C: 11 | I1: 20 (9.8%)  I2: 19 (9.3%)  I3: 19 (9.3%)  I4: 20  (9.8%)  C: 20 (9.8%) | **Type 1:** 2^nd^ week walking group: walk at a moderate or relaxed pace for 20 minutes 3x/week, beginning the second week postsurgery  **Type 2:** 1^st^ month walking group: walk at a moderate or relaxed pace for 20 minutes 3x/week, starting in the first month postsurgery  **Type 3:** 2^nd^ week waist exercise group: standard postoperative rehabilitation training (strengthening the lumbar core muscles for spinal stability) commencing 1^st^ week postsurgery  **Type 4:** 1^st^ month waist exercise group: standard postoperative rehabilitation (strengthening the lumbar core muscles for spinal stability) commencing 1^st^ month postsurgery.  **Duration:** NR  **Setting:** NR | **Non-exercise group:** instructed to perform usual daily activities without any additional exercise |
| Zarei et al. (2016)^334^  (Iran) | 2016 | 105 | LBP >12 weeks and radicular pain with evidence of nerve root compression of discs L1-L2 to L5-S1. | Standard surgery techniques, bilateral fora-menotomy and interlaminar discectomy | I1: 45  I2: 40  C: 44 | 12  10  9 | Pregabalin 14: 15 (42.9%)  Pregabalin 1: 18 (51.4%)  C: 14 (40%) | **Type 1:** pharmacological: one day oral Pregabalin (Pregabalin1): Pregabalin 300 mg 8 h preoperatively and 150 mg 12 and 24 hour postoperatively and placebo for the rest of 13 days.  **Type 2:** pharmacological: 14-day oral Pregabalin (Pregabalin14): Pregabalin 300 mg eight hours preoperatively and 150 mg every 12 hours postoperatively for 14 days | **Control:** placebo |
| Zhao et al. (2008)^335^  (China) | 2008 | 69 | Single segment lumbar intervertebral disc protrusion with chronic symptoms | Lumbar discectomy and fusion | I: 52.3  C: 52.5 | 7.6  8.9 | I: 16 (45.7%)  C: 14 (41.2%) | **Type:** acupuncture: Shenshu (BL 23), Dachangshu (BL 25), Baliao (BL 31-34), Zhibian (BL 54), Weizhong (BL 40), and Jiaji (EX-B2) to induce needling sensation. Electrical stimulation: Zhibian (BL 54) and Weizhong (BL 40) using G6805 electroacupuncture (continuous wave, 50 Hz, 30 min once daily). Cupping: every other day at Weizhong (BL 40) after electric stimulation. Conventional rehabilitation (same as comparison)  **Initiation postsurgery:** NR  **Frequency:** ≥6 treatment courses with 5-day interval (1 course = 15 days)  **Setting:** NR | **Control:** conventional rehabilitation: deaquation, nerve nutrition medication, 6 weeks of bed rest  Setting: NR |
| Zhang et al. (2018)^336^  (China) | 2018 | 92 | Disc protrusion with various severity of symptoms | Percutaneous transforaminal endoscopic discectomy | 57.4 | 6.1 | 44 (47.8%) (not reported by group) | **Type:** early functional exercises gradually added post-surgery (1 day, 1 week, 2 weeks, 5 weeks, and 10 weeks) | **Control:** routine functional exercises |
| Zheng et al. (2015)^337^  (China) | 2015 | 120 | Lumbar disc herniation | Fenestration of nucleus pulposus, hemilaminectomy decompression, total laminectomy decompression with fusion | I: 41.8  C: 42.7 | 14.8  12.7 | I: 24 (40%)  C: 18 (30%) | **Type:** usual care + supplementing qi, activating blood circulation and tonifying kidney therapy | **Control:** usual care including infection prevention, anti-inflammation, nourishing nerves, pain management, prevention of peptic ulcer and exercise |
| Zuo et al.  (2021)^338^  (China) | 2021 | 68 | Lumbar disc herniation with 37/68 on TSK  Excluded: other spinal conditions, chronic conditions with pain, previous spine surgery, participation in other studies, other surgeries performed with LDH surgery, severe pathologies | Lumbar discectomy, Lumbar intervertebral bone grafting and fusion, Lumbar disc herniation nucleectomy,  Lumbar transforaminal endoscopic surgery | I: 58.59  C: 59.65 | 15.09  14.79 | I: 18 (52.9%)  C: 17 (50.0%) | **Type:** Pilates exercises in addition to routine care and cognitive behavioral therapy  **Initiation postsurgery:** 1^st^ week postsurgery  **Duration:** 6 weeks  **Frequency:** 30 minutes/session, 2x/day  **Setting:** hospital and home | **Control:** routine care and cognitive behavioral therapy |

**ADL:** activities of daily living, **BGA:** behavioral graded activity, **C:** control group, **CBT:** cognitive behavioral therapy, **CBPT:** cognitive-behavioral-based physical therapy, **I:** intervention group, **LDH;** lumbar disc herniation, **MRI:** magnetic resonance imaging**, NR:** not reported, **PT:** physical therapist, **TSK:** Tampa Scale for Kinesiophobia

**Additional file 8a: Critical appraisal of low/moderate risk of bias studies**

| **Item** | **2.1 Question** | **2.2 Randomized** | **2.3 Concealment** | **2.4**  **Subject Blinding** | **2.5a Provider Blinding** | **2.5b Outcome Assessor Blinding** | **2.5c Biostatistician Blinding** | **2.6a Groups Similar** | **2.6b Baseline Differences Adjusted** | **2.7 Treatment Only Difference** | **2.8 Outcome Reliability** | **2.9 Outcome Validity** | **2.10a Dropouts** | **2.10b Missing Data** | **2.11 Intention to Treat** | **2.12 Site Comparability** |
| --- | --- | --- | --- | --- | --- | --- | --- | --- | --- | --- | --- | --- | --- | --- | --- | --- |
| Beneck et al. (2014)^313^ | -Yes | -Yes | -Cannot say | -Not possible | -Not possible | -Yes | -Yes | -Yes | -Yes | -Cannot say | -Yes | -Yes | I: *12%*  C: 32% | -Cannot say | -Cannot say | -Cannot say |
| Ebenbichler et al. (2015)^318^ | -Yes | -Yes | -Yes | -Not possible | -Not possible | -Yes | -Cannot say | -Yes | -Yes | -Cannot say | -Yes | -Yes | I: *28%*  Sham: *45%*  *Watchful waiting: 43%* | -Cannot say | -Yes | -Not applicable |
| Erdogmus et al. (2007)^317^ | -Yes | -Yes | -Yes | -Not possible | -Not possible | -Yes | -Cannot say | -Yes | -Yes | -Cannot say | -Yes | -Yes | I: *12.5%*  Sham: *20%*  Watchful waiting: *20%* | -Cannot say | -Yes | -Not applicable |
| He et al. (2021)^321^ | -Yes | -Yes | -No | -Not possible | -Not possible | -Cannot say | -Cannot say | -Yes | -No | -Cannot say | -Cannot say | -Cannot say | I: *0%*  C: *0%* | _Not applicable | -Cannot say | -Not applicable |
| Kulikov (2018)  (ID:45424904) | -Yes | -Cannot say | -Yes | -Not possible | -Not possible | -Cannot say | -Cannot say | -Yes | -Yes | -Cannot say | -Yes | -Yes | I: *0%*  C: *0%* | -Not applicable | -Yes | -Not applicable |
| Lu et al. (2020)^324^ | -Yes | -Yes | -No | -Yes | -Not possible | -Cannot say | -Cannot say | -Yes | -No | -Cannot say | -Yes | -Yes | I: *3.2%*  C: *3.2%* | -Cannot say | -Yes | -Not applicable |
| Oosterhuis et al. (2017)^326^ | -Yes | -Yes | -Yes | -Not possible | -Not possible | -Cannot say | -Cannot say | -No | -Yes | -Yes | -Yes | -Yes | I: *10.9%*  C: *7.8%* | -Cannot say | -Yes | -Cannot say |
| Ostelo et al. (2003)^328^ | -Yes | -Yes | -Yes | -Not possible | -Not possible | -Cannot say | -Cannot say | -Yes | -Yes | -Cannot say | -Yes | -Yes | I: *19.2%*  C: *3.8%* | -Yes | -Yes | -Not applicable |
| Ostelo et al. (2003)^327^ | -Yes | -Yes | -Yes | -Not possible | -Not possible | -Yes | -Cannot say | -Yes | -Yes | -Cannot say | -Yes | -Yes | I: *3.8%*  C: *1.8%* | -Yes | -Yes | -Not applicable |
| Zarei et al. (2016)^334^ | -Yes | -Yes | -Cannot say | -Yes | -Yes | -Yes | -Cannot say | -Yes | -Not applicable | -Cannot say | -Yes | -Yes | -Not reported | -Cannot say | -Cannot say | -Not applicable |
| Zuo et al.  (2021)^338^ | -Yes | -Yes | -No | -Not possible | -Not possible | -Not possible | -Cannot say | -Yes | -Not applicable | -Cannot say | -Yes | -Yes | -Not reported | -Cannot say | -Cannot say | -Not applicable |

**C:** control; **I:** intervention

**2.1 Question**: The study addresses an appropriate and clearly focused question; **2.2 Randomizaed**: The assignment of subjects to treatment groups is randomised; **2.3 Concealment**: An adequate concealment method is used; **2.4 Subject Blinding**: Subjects are kept ‘blind’ to treatment allocation; **2.5a Provider Blinding**: Treatment provider is kept ‘blind’ to treatment allocation; **2.5b Outcome Assessor Blinding**: Outcome assessor is kept ‘blind’ to treatment allocation; **2.5c Biostatistician Blinding**: Bio- statistician is kept ‘blind’ to treatment allocation; **2.6a Groups Similar**: The treatment and control groups are similar at the start of the trial; **2.6b Baseline Differences Adjusted**: Important baseline difference between groups was adjusted as covariates; **2.7 Treatment Only Difference**: The only difference between the groups is the treatment(s) under investigation; **2.8 Outcome Reliability**: The outcomes are measured in a reliable way; 2**.9 Outcome Validity**: The outcomes are measured in a valid way; **2.10a Dropouts**: What percentage of the individuals or clusters recruited into the study dropped out before the study was completed; **2.10b Missing Data**: Is the missing data was being dealt with; **2.11 Intention to Treat**: All subjects are analysed in the groups to which they were randomly allocated (intention to treat analysis); **2.12 Site Comparability**: Where the study is carried out at more than one site, results are comparable for all sites

*Drop-out reported at the last follow-up time point

**Additional file 8b: Critical appraisal of high risk of bias studies**

| **Item** | **2.1 Question** | **2.2 Randomized** | **2.3 Concealment** | **2.4**  **Subject Blinding** | **2.5a Provider Blinding** | **2.5b Outcome Assessor Blinding** | **2.5c Biostatistician Blinding** | **2.6a Groups Similar** | **2.6b Baseline Differences Adjusted** | **2.7 Treatment Only Difference** | **2.8 Outcome Reliability** | **2.9 Outcome Validity** | **2.10a Dropouts** | **2.10b Missing Data** | **2.11 Intention to Treat** | **2.12 Site Comparability** |
| --- | --- | --- | --- | --- | --- | --- | --- | --- | --- | --- | --- | --- | --- | --- | --- | --- |
| Abdi et al.,  (2023)^311^ | -Yes | -Yes | -No | -Not possible | -Not possible | -Not possible | -Cannot say | -Cannot say | -No | -Cannot say | -Yes | -Yes | I1: 6.7*%*  I2: 3.3%  C: *0%* | -No | -Cannot say | -Not applicable |
| Aldemir and Gurkan (2021)^312^ | -Yes | -Yes | -No | -Not possible | -Not possible | -Cannot say | -Cannot say | -No | -No | -Cannot say | -Cannot say | -Cannot say | I: *17.5%*  C: *15%* | -No | -Cannot say | -Not applicable |
| Bono et al. (2017)^314^ | -Yes | -Cannot say | -Yes | -Not possible | -Not possible | -Cannot say | -Cannot say | -Yes | -Not applicable | -Cannot say | -Yes | -Yes | I: *35.7%*  C: *41.1%* | -Cannot say | -Cannot say | -Cannot say |
| Choi et al. (2005)^315^ | -Yes | -Cannot say | -No | -Not possible | -Not possible | -Cannot say | -Cannot say | -Cannot say | -No | -Cannot say | -Yes | -Yes | -Not reported | -Cannot say | -Cannot say | -Not applicable |
| Erdogan and Bulut (2019)^316^ | -Yes | -Yes | -No | -Cannot say | -Cannot say | -Cannot say | -Cannot say | -No | -No | -No | -Yes | -Yes | I: *0%*  C: *0%* | -Not applicable | -Yes | -Not applicable |
| Hakkinen et al. (2005)^320^ | -Yes | -Cannot say | -No | -Cannot say | -Cannot say | -Yes | -Cannot say | -Yes | -Not applicable | -Yes | -Yes | -Yes | I: 29.2*%*  C: *23%* | -Cannot say | -Yes | -Not applicable |
| Jentoft et al. (2020)^322^ | -Yes | -Yes | -Yes | -Not possible | -Not possible | -Not possible | -Unclear | -No | -No | -Cannot say | -Yes | -Yes | I: *17.5%*  C: *7.5%* | -No | -Yes | -Not applicable |
| Manniche et al. (1993)^325^ | -Yes | -Yes | -Cannot say | -Not possible | -Not possible | -Yes | -Cannot say | -Cannot say | -Cannot say | -Cannot say | -Yes | -Yes | Overall: 15% | -Cannot say | -Cannot say | -Cannot say |
| Paulsen et al. (2019)^329^ | -Yes | -Cannot say | -No | -Cannot say | -Cannot say | -Cannot say | -Cannot say | -No | -No | -Cannot say | -Yes | -Yes | I: *22%*  C: *22%* | -Cannot say | -Yes | -Not applicable |
| Paulsen (2019)^330^ | -Yes | -Cannot say | -No | -Cannot say | -Cannot say | -Cannot say | -Cannot say | -No | -Yes | -Cannot say | -Yes | -Yes | I: *22%*  C: *22%* | -Cannot say | -Yes | -Not applicable |
| Pekar and Steindler (2009)^331^ | -Yes | -Cannot say | -No | -No | -No | -Cannot say | -No | -Yes | -Cannot say | -Cannot say | -Yes | -Yes | I: *8%*  C: *28%* | -Cannot say | -No | -Not applicable |
| Fu and Ji (2017)^319^ | -Yes | -Yes | -Cannot say | -Not possible | -Not possible | -Not possible | -Cannot say | -Cannot say | -Not applicable | -Cannot say | -Yes | -Yes | -Not reported | -Cannot say | -Cannot say | -Not applicable |
| Skall et al. (1994)^332^ | -Yes | -Cannot say | -No | -Not possible | -Not possible | -Not possible | -Cannot say | -No | -Cannot say | -Cannot say | -Yes | -Yes | I: *12.5%*  C: *17.1%* | -Cannot say | -No | -Cannot say |
| Uysal et al.  (2023)^333^ | -Yes | -Cannot say | -No | -Not possible | -Not possible | -Not possible | -Cannot say | -Cannot say | -No | -Cannot say | -Yes | -Yes | -Not reported | -Cannot say | -Cannot say | -Cannot say |
| Zhang et al. (2018)^336^ | -Yes | -Cannot say | -No | -Cannot say | -Cannot say | -Cannot say | -Cannot say | -Cannot say | -Cannot say | -Cannot say | -Cannot say | -Cannot say | I: *0%*  C: *0%* | -Not applicable | -Cannot say | -Not applicable |
| Zhao et al. (2008)^335^ | -Yes | -Cannot say | -No | -Not possible | -Not possible | -Cannot say | -Cannot say | -Yes | -Cannot say | -Cannot say | -Yes | -Yes | -Not reported | -Cannot say | -Cannot say | -Not applicable |
| Zheng et al. (2015)^337^ | -Yes | -Yes | -Cannot say | -Not possible | -Not possible | -Not possible | -Cannot say | -Cannot say | -Not applicable | -Cannot say | -Yes | -Yes | -Not reported | -Cannot say | -Cannot say | -Not applicable |

**C:** control; **I:** intervention

**2.1 Question**: The study addresses an appropriate and clearly focused question; **2.2 Randomizaed**: The assignment of subjects to treatment groups is randomised; **2.3 Concealment**: An adequate concealment method is used; **2.4 Subject Blinding**: Subjects are kept ‘blind’ to treatment allocation; **2.5a Provider Blinding**: Treatment provider is kept ‘blind’ to treatment allocation; **2.5b Outcome Assessor Blinding**: Outcome assessor is kept ‘blind’ to treatment allocation; **2.5c Biostatistician Blinding**: Bio- statistician is kept ‘blind’ to treatment allocation; **2.6a Groups Similar**: The treatment and control groups are similar at the start of the trial; **2.6b Baseline Differences Adjusted**: Important baseline difference between groups was adjusted as covariates; **2.7 Treatment Only Difference**: The only difference between the groups is the treatment(s) under investigation; **2.8 Outcome Reliability**: The outcomes are measured in a reliable way; 2**.9 Outcome Validity**: The outcomes are measured in a valid way; **2.10a Dropouts**: What percentage of the individuals or clusters recruited into the study dropped out before the study was completed; **2.10b Missing Data**: Is the missing data was being dealt with; **2.11 Intention to Treat**: All subjects are analysed in the groups to which they were randomly allocated (intention to treat analysis); **2.12 Site Comparability**: Where the study is carried out at more than one site, results are comparable for all sites

*Drop-out reported at the last follow-up time point

**Additional file 9: Evidence profiles of relevant studies**

**File 9.1: GRADE evidence profile of exercise vs. control (e.g., usual care, no intervention, wait-list control) for patients who were treated surgically for lumbar disc herniation from 2 randomized controlled trials**

| **Study** | **Outcome Measure** | **Risk of bias^a^** | **Imprecision^b^** | **Indirectness^c^** | **Inconsistency^d^** | **Publication bias detected?^e^** | **Certainty of evidence^f^** | **Intervention (no. of patients)** | **Comparison (no. of patients)** | **Between-group effect (mean difference (MD) or risk ratio (RR) (95% CI))** |
| --- | --- | --- | --- | --- | --- | --- | --- | --- | --- | --- |
| **Function (short-term follow up: >1 week to 3 months)** | | | | | | | | | | |
| He et al. (2021)^321^ | ODI, 0-50; lower is better;  3 months post-surgery  10% for between-group difference=5 | Not serious | Serious | Not serious | Not serious | No | Moderate | N=47 | N=48 | MD 3.33 (1.4, 5.3)* |
| Paulsen et al. (2019)^329^ | ODI, 0-100; lower is better  10% for between-group difference=10 | Very serious^g^ | Not serious | Not serious | Not serious | No | Low | N=73 | N=73 | MD 2.6 (-0.66, 5.86) |
| Uysal et al.  (2023)^333^  2^nd^ week walking | ODI, 0-100; lower is better  10% for between-group difference=10 | Very serious^h^ | Not serious | Not serious | Not serious | No | Low | N=40 | N=41 | MD 20.5 (18.09, 22.91)* |
| Uysal et al.  (2023)^333^  1^st^ month walking | ODI, 0-100; lower is better  10% for between-group difference=10 | Very serious^h^ | Serious | Not serious | Not serious | No | Very low | N=39 | N=41 | MD 10.7 (7.95, 13.45)* |
| Uysal et al.  (2023)^333^  2^nd^ week waist exercise | ODI, 0-100; lower is better  10% for between-group difference=10 | Very serious^h^ | Not serious | Not serious | Not serious | No | Low | N=42 | N=41 | MD 19.8 (17.40, 22.20)* |
| Uysal et al.  (2023)^333^  1^st^ month waist exercise | ODI, 0-100; lower is better  10% for between-group difference=10 | Very serious^h^ | Not serious | Not serious | Not serious | No | Low | N=42 | N=41 | MD 13.4 (11.02, 15.78)* |
| Zuo et al.  (2021)^338^ | ODI, 0-50  lower is better; 6^th^ week post-surgery  10% for between-group difference=5 | Not serious | Not serious | Not serious | Not serious | No | High | N=34 | N=34 | MD 6.95 (5.58, 8.31)* |
| **Function (intermediate term follow up: > 3 months to 1 year)** | | | | | | | | | | |
| Aldemir et al. (2021)^312^ | ODI, 0-100; lower is better;  3 months and 3 weeks post-surgery  10% for between-group difference=10 | Very serious^i^ | Not serious | Not serious | Not serious | No | Low | N=40 | N=40 | MD 0.7 (-1.0, 2.4)* |
| Lu and Bai (2020)^324^ | ODI, 0-100; lower is better;  24 weeks post-surgery  10% for between-group difference=10 | Not serious | Not serious | Not serious | Not serious | No | High | N=30 | N=30 | MD -1.06 (-3.3, 1.2)* |
| Paulsen et al. (2019)^329^ | ODI, 0-100; lower is better  10% for between-group difference=10 | Very serious^g^ | Not serious | Not serious | Not serious | No | Low | N=73 | N=73 | MD 0.8 (-2.38, 3.98) |
| Uysal et al.  (2023)^333^  2^nd^ week walking | ODI, 0-100; lower is better  10% for between-group difference=10 | Very serious^h^ | Not serious | Not serious | Not serious | No | Low | N=40 | N=41 | MD 12.9 (10.64, 15.16)* |
| Uysal et al.  (2023)^333^  1^st^ month walking | ODI, 0-100; lower is better  10% for between-group difference=10 | Very serious^h^ | Not serious | Not serious | Not serious | No | Low | N=39 | N=41 | MD 14.3 (11.70, 16.90)* |
| Uysal et al.  (2023)^333^  2^nd^ week waist exercise | ODI, 0-100; lower is better  10% for between-group difference=10 | Very serious^h^ | Serious | Not serious | Not serious | No | Very low | N=42 | N=41 | MD 9.5 (7.21, 11.79)* |
| Uysal et al.  (2023)^333^  1^st^ month waist exercise | ODI, 0-100; lower is better  10% for between-group difference=10 | Very serious^h^ | Not serious | Not serious | Not serious | No | Low | N=42 | N=41 | MD 4.9 (2.67, 7.13)* |
| **Function (long-term follow up: > 1 year)** | | | | | | | | | | |
| Paulsen et al. (2019)^329^ | ODI, 0-100; lower is better  10% for between-group difference=10 | Very serious^g^ | Not serious | Not serious | Not serious | No | Low | N=73 | N=73 | MD 3.1 (-0.08, 6.28) |
| **Low back pain (short-term follow up: >1 week to 3 months)** | | | | | | | | | | |
| Paulsen et al. (2019)^329^ | VAS, 100mm;  lower is better  10% for between-group difference=10 | Very serious^g^ | Not serious | Not serious | Not serious | No | Low | N=73 | N=73 | MD 1.2 (-4.48, 6.88) |
| Uysal et al.  (2023)^333^  2^nd^ week walking | VAS, 10cm;  lower is better  10% for between-group difference=1 | Very serious^h^ | Not serious | Not serious | Not serious | No | Low | N=40 | N=41 | MD 3.07 (2.81, 3.33)* |
| Uysal et al.  (2023)^333^  1^st^ month walking | VAS, 10cm;  lower is better  10% for between-group difference=1 | Very serious^h^ | Not serious | Not serious | Not serious | No | Low | N=39 | N=41 | MD 2.13 (1.87, 2.39)* |
| Uysal et al.  (2023)^333^  2^nd^ week waist exercise | VAS, 10cm;  lower is better  10% for between-group difference=1 | Very serious^h^ | Not serious | Not serious | Not serious | No | Low | N=42 | N=41 | MD 3.32 (3.07, 3.57)* |
| Uysal et al.  (2023)^333^  1^st^ month waist exercise | VAS, 10cm;  lower is better  10% for between-group difference=1 | Very serious^h^ | Not serious | Not serious | Not serious | No | Low | N=42 | N=41 | MD 2.82 (2.57, 3.07)* |
| **Low back pain (intermediate term follow up: > 3 months to 1 year)** | | | | | | | | | | |
| Lu and Bai (2020)^324^ | NRS, 0-10;  lower is better;  24 weeks post-surgery  10% for between-group difference=1 | Not serious | Not serious | Not serious | Not serious | No | High | N=30 | N=30 | MD -0.17 (-0.4, 0.1)* |
| Paulsen et al. (2019)^329^ | VAS, 100mm;  lower is better  10% for between-group difference=10 | Very serious^g^ | Serious | Not serious | Not serious | No | Very low | N=73 | N=73 | MD -8.21 (-13.94, -2.48) |
| Uysal et al.  (2023)^333^  2^nd^ week walking | VAS, 10cm;  lower is better  10% for between-group difference=1 | Very serious^h^ | Not serious | Not serious | Not serious | No | Low | N=40 | N=41 | MD 1.68 (1.44, 1.92)* |
| Uysal et al.  (2023)^333^  1^st^ month walking | VAS, 10cm;  lower is better  10% for between-group difference=1 | Very serious^h^ | Not serious | Not serious | Not serious | No | Low | N=39 | N=41 | MD 1.8 (1.55, 2.05)* |
| Uysal et al.  (2023)^333^  2^nd^ week waist exercise | VAS, 10cm;  lower is better  10% for between-group difference=1 | Very serious^h^ | Not serious | Not serious | Not serious | No | Low | N=42 | N=41 | MD 1.72 (1.48, 1.96)* |
| Uysal et al.  (2023)^333^  1^st^ month waist exercise | VAS, 10cm;  lower is better  10% for between-group difference=1 | Very serious^h^ | Not serious | Not serious | Not serious | No | Low | N=42 | N=41 | MD 1.41 (1.17, 1.65)* |
| **Low back pain (≥ 1 year)** | | | | | | | | | | |
| Paulsen et al. (2019)^329^ | VAS, 100mm;  lower is better  10% for between-group difference=10 | Very serious^g^ | Serious | Not serious | Not serious | No | Very low | N=73 | N=73 | MD -7.14 (-12.77, -1.51) |
| **Leg pain (short-term follow up: >1 week to 3 months)** | | | | | | | | | | |
| Paulsen et al. (2019)^329^ | VAS, 100mm;  lower is better  10% for between-group difference=10 | Very serious^g^ | Not serious | Not serious | Not serious | No | Low | N=73 | N=73 | MD -2.27 (-6.82, 2.28) |
| **Leg pain (intermediate term follow up: > 3 months to 1 year)** | | | | | | | | | | |
| Paulsen et al. (2019)^329^ | VAS, 100mm;  lower is better  10% for between-group difference=10 | Very serious^g^ | Serious | Not serious | Not serious | No | Very low | N=73 | N=73 | MD -10.38 (-14.92, -5.84) |
| **Leg pain (long-term follow up: > 1 year)** | | | | | | | | | | |
| Paulsen et al. (2019)^329^ | VAS, 100mm;  lower is better  10% for between-group difference=10 | Very serious^g^ | Serious | Not serious | Not serious | No | Very low | N=73 | N=73 | MD -4.73 (-10.06, 0.6) |
| **Low back and leg pain (intermediate term follow up: > 3 months to 1 year)** | | | | | | | | | | |
| Aldemir et al. (2021)^312^ | VAS (third section of McGill pain questionnaire), 10cm;  3 months and 3 weeks post-surgery  10% for between-group difference=1 | Very serious^i^ | Not serious | Not serious | Not serious | No | Low | N=40 | N=40 | MD 0.27 (-0.1, 0.7)* |
| **Quality of life (short-term follow up: >1 week to 3 months)** | | | | | | | | | | |
| He et al. (2021)^321^ | SF-36, 0-100; higher is better;  3 months post-surgery  10% for between-group difference=10 | Not serious | Not serious | Not serious | Not serious | No | High | N=47 | N=48 | MD: PCS: 3.46 (1.7, 5.3)*  MD: MCS: 3.87 (1.2, 6.6)* |
| Paulsen et al. (2019)^329^ | EQ-5D, 0-1;  higher is better  10% for between-group difference=0.1 | Very serious^g^ | Serious | Not serious | Not serious | No | Very low | N=73 | N=73 | MD 0.06 (0.00, 0.12) |
| **Quality of life (intermediate term follow up: > 3 months to 1 year)** | | | | | | | | | | |
| Lu and Bai (2020)^324^ | SF-36, unclear score range (>700); higher is better;  24 weeks post-surgery  10% for between-group difference>70 | Not serious | Serious^k^ | Not serious | Not serious | No | Moderate | N=30 | N=30 | MD 27.86 (9.3, 46.4)* |
| Paulsen et al. (2019)^329^ | EQ-5D, 0-1;  higher is better  10% for between-group difference=0.1 | Very serious^g^ | Not serious | Not serious | Not serious | No | Low | N=73 | N=73 | MD -0.01 (-0.07, 0.05) |
| **Quality of life (long-term follow up: > 1 year)** | | | | | | | | | | |
| Paulsen et al. (2019)^329^ | EQ-5D, 0-1;  higher is better  10% for between-group difference=0.1 | Very serious^g^ | Not serious | Not serious | Not serious | No | Low | N=73 | N=73 | MD -0.01 (-0.07, 0.05) |
| **Fear of movement (short-term follow up: >1 week to 3 months)** | | | | | | | | | | |
| Zuo et al.  (2021)^338^ | TSK, 17-68  lower is better; 6^th^ week post-surgery  10% for between-group difference=6.8 | Not serious | Not serious | Not serious | Not serious | No | High | N=34 | N=34 | MD 4.77 (3.41, 6.13)* |
| **Work outcomes** **(long-term follow up: > 1 year)** | | | | | | | | | | |
| Paulsen et al. (2020)^330^ | RTW at 1 year | Very serious^g^ | Very serious | Not serious | Not serious | No | Very low | N=73 | N=73 | RR: 0.93 (0.75, 1.15)  Participants in intervention group are 7% less likely to return to work |
| Skall et al. (1994) (mild exercise)^332^ | Shifted to easier job or left work force (yes/no) at 1 year since post-surgical intervention | Very serious^j^ | Very serious | Not serious | Not serious | No | Very low | N=49 | N=63 | RR 0.96 (0.53, 1.74)*  Participants in intervention group are 4% less likely to shift to easier job or leave work. |
| Skall et al. (1994) (intense exercise)^332^ | Shifted to easier job or left work force (yes/no) at 1 year since post-surgical intervention | Very serious^j^ | Serious | Not serious | Not serious | No | Very low | N=47 | N=63 | RR 0.46 (0.20, 1.04)*  Participants in intervention group are 54% less likely to shift to easier job or leave work. |

**CI:** confidence interval, **LBPRS:** Low Back Pain Rating Scale, **MD:** mean difference, **NRS:** Numeric Rating Scale, **ODI:** Oswestry Disability Index, **RMDQ:** Roland-Morris Disability Questionnaire, **VAS:** Visual Analogue Scale

^a^Risk of bias: Options are not serious, serious (rate certainty of evidence down one level), and very serious (rate certainty of evidence down two levels). 1. Not serious: study rated as ‘low risk of bias’ or ‘some concerns’ (e.g., unclear co-interventions, no detailed randomization method described but similar baseline characteristics between groups). 2. Serious: study rated as ‘high risk of bias’ with unbalanced baseline characteristics between groups, unclear co-interventions, high/unbalanced drop-out and/or unclear intention-to-treat analysis. 3. Very serious: study rated as ‘high risk of bias’ with unclear randomization sequence generation, inadequate allocation concealment and/or lack of blinding.

^b^Imprecision: Options are not serious, serious (rate certainty of evidence down one level), and very serious (rate certainty of evidence down two levels). Imprecision assessed using between-group effect [point estimate (95% CI)]. 1. Not serious: If the point estimate is not clinically important: the upper and lower boundaries of the CI do not cross a clinically important threshold; the CI may cross the null as long as neither boundary crosses a clinically important threshold. If the point estimate is clinically important: the CI does not cross the null and the boundaries do not cross a clinically important threshold. 2. Serious: If the point estimate is not clinically important: the CI may or may not cross the null but one of the boundaries crosses a clinically important threshold. If the point estimate is clinically important: the CI may cross the null but does not cross a clinically important threshold in the other direction. 3. Very serious: If the point estimate is or is not clinically important: the CI crosses the boundaries of both appreciable harm and benefit (i.e., very wide CI).

^c^Indirectness: Options are not serious, serious (rate certainty of evidence down one level), and very serious (rate certainty of evidence down two levels). Indirectness assessed whether the patients, interventions, or outcomes are different from the research question under investigation.

^d^Inconsistency: Options are not serious, serious (rate certainty of evidence down one level), and very serious (rate certainty of evidence down two levels). Inconsistency assessed effect estimate variance in direction or magnitude. 1. Not serious: effect estimates are consistent in direction and magnitude across studies. 2. Serious: effect estimates vary in magnitude across studies and the heterogeneity could not be explained. 3. Very serious: effect estimates vary in direction across studies and the heterogeneity could not be explained. We rate inconsistency as not serious when there is only one single RCT for an outcome; however, there are no other trials with which to compare findings.

^e^Publication bias assessed using funnel plot if possible, or based on available information from clinical trial registries.

^f^Certainty of evidence: High certainty: we are very confident that the true effect lies close to that of the estimate of the effect. Moderate certainty: we are moderately confident in the effect estimate; the true effect is likely to be close to the estimate of the effect, but there is a possibility that it is substantially different. Low certainty: our confidence in the effect estimate is limited: the true effect may be substantially different from the estimate of the effect. Very low certainty: we have very little confidence in the effect estimate: the true effect is likely to be substantially different from the estimate of effect.

^g^Unclear randomization sequence generation; inadequate allocation concealment; unclear blinding (patients, treatment providers, outcome assessors, data analyst); group differences at baseline; group differences not adjusted for in analysis; unclear co-interventions; unclear handling of missing data

^h^Unclear randomization sequence generation; inadequate allocation concealment; unclear blinding (patients, treatment providers, outcome assessors, data analyst); unclear group differences at baseline; unclear co-interventions; drop-out rates not reported by group; unclear intention-to-treat analysis; unclear comparability of results across sites

^i^No information of allocation concealment; group differences at baseline; group differences not adjusted for in analysis; unclear co-interventions; unclear intention-to-treat analysis

^j^Unclear randomization sequence generation; no information of allocation concealment; not possible to blind patients, treatment providers, outcome assessors; unclear blinding of data analyst; no information of group differences at baseline; unclear if group differences adjusted for in analysis; unclear co-interventions; unclear handling of missing data; unclear intention-to-treat analysis; unclear comparability of results across sites

^k^We are unsure about the scale range. Therefore, we downgraded one level for imprecision here.

^l^We cannot evaluate imprecision domain based on p value. Therefore, we did not GRADE Skall et al. (1994) for the outcomes of function and low back pain.

*Calculated by review authors based on raw data provided in RCT

^+^ 95% CI is not reported in the RCT and the review authors cannot calculate 95% CI due to lack of raw data

**File 9.2: GRADE evidence profile of exercise vs. another intervention for patients who were treated surgically for lumbar disc herniation from 5 randomized controlled trials**

| **Study** | **Outcome Measure** | **Risk of bias^a^** | **Imprecision^b^** | **Indirectness^c^** | **Inconsistency^d^** | **Publication bias detected?^e^** | **Certainty of evidence^f^** | **Intervention (no. of patients)** | **Comparison (no. of patients)** | **Between-group effect (mean difference (MD) or risk ratio (RR) (95% CI))** |
| --- | --- | --- | --- | --- | --- | --- | --- | --- | --- | --- |
| **Function (short-term follow up: >1 week to 3 months)** | | | | | | | | | | |
| Jentoft et al. (2020)^322^ | ODI, 0-100; lower is better;  6-8 weeks post-surgery  10% for between-group difference=10 | Serious^g^ | Serious | Not serious | Not serious | No | Low | N=33 | N=37 | MD 9.6 (4.4, 14.8)* |
| **Function (intermediate term follow up: > 3 months to 1 year)** | | | | | | | | | | |
| Abdi et al.  (2023)^311^  Flexion-based exercise | ODI, 0-50; lower is better; end of 14^th^ week post-surgery  10% for between-group difference=5 | Very seriou^h^ | Not serious | Not serious | Not serious | No | Low | N=30 | N=30 | MD 7.11 (5.75, 8.47)* |
| Abdi et al.  (2023)^311^  Extension-based exercise | ODI, 0-50; lower is better; end of 14^th^ week post-surgery  10% for between-group difference=5 | Very seriou^h^ | Serious | Not serious | Not serious | No | Very low | N=30 | N=30 | MD 4.42 (3.22, 5.62)* |
| Jentoft et al. (2020)^322^ | ODI, 0-100; lower is better; 12 months post-surgery  10% for between-group difference=10 | Serious^g^ | Serious | Not serious | Not serious | No | Low | N=33 | N=37 | MD 11.9 (5.9, 17.9)* |
| **Low back pain (short-term follow up: >1 week to 3 months)** | | | | | | | | | | |
| Jentoft et al. (2020)^322^ | NRS, 0-10;  lower is better;  6-8 weeks post-surgery  10% for between-group difference=1 | Serious^g^ | Not serious | Not serious | Not serious | No | Moderate | N=33 | N=37 | MD 0.1 (-0.7, 0.9)* |
| **Low back pain (intermediate term follow up: > 3 months to 1 year)** | | | | | | | | | | |
| Abdi et al.  (2023)^311^  Flexion-based exercise | VAS, 0-10; lower is better; end of 14^th^ week post-surgery  10% for between-group difference=1 | Very seriou^h^ | Serious | Not serious | Not serious | No | Very low | N=30 | N=30 | MD 0.85 (0.53, 1.17)* |
| Abdi et al.  (2023)^311^  Extension-based exercise | VAS, 0-10; lower is better; end of 14^th^ week post-surgery  10% for between-group difference=1 | Very seriou^h^ | Serious | Not serious | Not serious | No | Very low | N=30 | N=30 | MD 0.77 (0.45, 1.09)* |
| Jentoft et al. (2020)^322^ | NRS, 0-10;  lower is better;  12 months post-surgery  10% for between-group difference=1 | Serious^g^ | Serious | Not serious | Not serious | No | Low | N=33 | N=37 | MD 1 (0, 2.0)* |
| **Leg pain (short-term follow up: >1 week to 3 months)** | | | | | | | | | | |
| Jentoft et al. (2020)^322^ | NRS, 0-10;  lower is better;  12 months post-surgery  10% for between-group difference=1 | Serious^g^ | Serious | Not serious | Not serious | No | Low | N=33 | N=37 | MD 0.6 (-0.2, 1.4)* |
| **Leg pain (intermediate term follow up: > 3 months to 1 year)** | | | | | | | | | | |
| Jentoft et al. (2020)^322^ | NRS, 0-10;  lower is better;  12 months post-surgery  10% for between-group difference=1 | Serious^g^ | Serious | Not serious | Not serious | No | Low | N=33 | N=37 | MD 1.9 (0.8, 3.0)* |
| **Quality of life (intermediate term follow up: > 3 months to 1 year)** | | | | | | | | | | |
| Beneck et al. (2014)^313^ | SF-36, 0-100;  (Physical component scale)  higher is better  10% for between-group difference=10 | Not serious | Not serious | Not serious | Not serious | No | High | N=51 | N=47 | MD 4.26 (1.39; 7.13)* |
| Zhang et al. (2018)^336^ | SF-36, 0-100; higher is better  10% for between-group difference=10 | Very serious^i^ | Serious | Not serious | Not serious | No | Very low | N=46 | N=46 | MD 16.9 (9.41, 24.39)* |
| **Fear of movement (short-term follow up: >1 week to 3 months)** | | | | | | | | | | |
| Jentoft et al. (2020)^322^ | TSK-13, 13-52; lower is better; 6-8 weeks post-surgery  10% for between-group difference=5.2 | Serious^g^ | Not serious | Not serious | Not serious | No | Moderate | N=33 | N=37 | MD 1.6 (-1.0, 4.2)* |
| **Fear of movement (intermediate term follow up: >3 months to 1 year)** | | | | | | | | | | |
| Jentoft et al. (2020)^322^ | TSK-13, 13-52; lower is better; 6-12 months post-surgery  10% for between-group difference=5.2 | Serious^g^ | Serious | Not serious | Not serious | No | Low | N=33 | N=37 | MD 3.4 (0.5, 6.3)* |
| **Fear avoidance beliefs (short-term follow up: >1 week to 3 months)** | | | | | | | | | | |
| Jentoft et al. (2020)^322^ | FABQ, 0-24; lower is better; 6-8 weeks post-surgery  10% for between-group difference=2.4 | Serious^g^ | Serious | Not serious | Not serious | No | Low | N=33 | N=37 | MD 1 (-0.9, 2.9)* |
| **Fear avoidance beliefs (intermediate term follow up: >3 months to 1 year)** | | | | | | | | | | |
| Jentoft et al. (2020)^322^ | FABQ, 0-24; lower is better; 12 months post-surgery  10% for between-group difference=2.4 | Serious^g^ | Serious | Not serious | Not serious | No | Low | N=33 | N=37 | MD -1.2 (-3.2, 0.8)* |
| **Recovery** **(long-term follow up: > 1 year)** | | | | | | | | | | |
| Zhang et al. (2018)^336^ | Curative effect (excellent/good/poor) | Very serious^i^ | Serious | Not serious | Not serious | No | Very low | N=46 | N=46 | RR excellent/good vs. poor  1.10 (0.98, 1.22)  Participants in intervention group are 1.1 times more likely to perceive treatment effect. |
| **Work outcomes (intermediate term follow up: > 3 months to 1 year)** | | | | | | | | | | |
| Choi et al. (2020)^315^ | RTW Yes/no | Very serious^j^ | Not serious | Not serious | Not serious | No | Low | N=40 | N=40 | RR 3.43 (1.97, 5.96)*  Participants in intervention group are 3.43 times more likely to return to work. |
| **Work outcomes** **(long-term follow up: > 1 year)** | | | | | | | | | | |
| Hakkinen et al. (2005)^320^ | Sick leave Yes/no | Very serious^k^ | Very serious | Not serious | Not serious | No | Very low | N=65 | N=61 | RR 1.1 (0.5, 2.7)*  Participants in intervention group are 1.1 times more likely to sick leave. |
| Manniche et al. (1993) (mild vs. intense)^325^ | Return to original work  Yes/no | Very serious^l^ | Serious | Not serious | Not serious | No | Very low | N=42 | N=40 | RR 1.22 (0.97, 1.55)  Participants in intervention group are 1.2 times more likely to return to original work. |
| Skall et al. (1994) (mild vs. intense)^332^ | Shifted to easier job or left work force yes/no | Very serious^m^ | Serious | Not serious | Not serious | No | Very low | N=49 | N=47 | RR 2.11 (0.89, 5.03)*  Participants in intervention group are 2.11 times more likely to shift to easier job or leave work. |

**CI:** confidence interval, **LBPRS:** Low Back Pain Rating Scale, **MD:** mean difference, **NRS:** Numeric Rating Scale, **ODI:** Oswestry Disability Index, **SF-36:** Short-form 36, **VAS:** Visual Analogue Scale

^a^Risk of bias: Options are not serious, serious (rate certainty of evidence down one level), and very serious (rate certainty of evidence down two levels). 1. Not serious: study rated as ‘low risk of bias’ or ‘some concerns’ (e.g., unclear co-interventions, no detailed randomization method described but similar baseline characteristics between groups). 2. Serious: study rated as ‘high risk of bias’ with unbalanced baseline characteristics between groups, unclear co-interventions, high/unbalanced drop-out and/or unclear intention-to-treat analysis. 3. Very serious: study rated as ‘high risk of bias’ with unclear randomization sequence generation, inadequate allocation concealment and/or lack of blinding.

^b^Imprecision: Options are not serious, serious (rate certainty of evidence down one level), and very serious (rate certainty of evidence down two levels). Imprecision assessed using between-group effect [point estimate (95% CI)]. 1. Not serious: If the point estimate is not clinically important: the upper and lower boundaries of the CI do not cross a clinically important threshold; the CI may cross the null as long as neither boundary crosses a clinically important threshold. If the point estimate is clinically important: the CI does not cross the null and the boundaries do not cross a clinically important threshold. 2. Serious: If the point estimate is not clinically important: the CI may or may not cross the null but one of the boundaries crosses a clinically important threshold. If the point estimate is clinically important: the CI may cross the null but does not cross a clinically important threshold in the other direction. 3. Very serious: If the point estimate is or is not clinically important: the CI crosses the boundaries of both appreciable harm and benefit (i.e., very wide CI).

^c^Indirectness: Options are not serious, serious (rate certainty of evidence down one level), and very serious (rate certainty of evidence down two levels). Indirectness assessed whether the patients, interventions, or outcomes are different from the research question under investigation.

^d^Inconsistency: Options are not serious, serious (rate certainty of evidence down one level), and very serious (rate certainty of evidence down two levels). Inconsistency assessed effect estimate variance in direction or magnitude. 1. Not serious: effect estimates are consistent in direction and magnitude across studies. 2. Serious: effect estimates vary in magnitude across studies and the heterogeneity could not be explained. 3. Very serious: effect estimates vary in direction across studies and the heterogeneity could not be explained. We rate inconsistency as not serious when there is only one single RCT for an outcome; however, there are no other trials with which to compare findings.

^e^Publication bias assessed using funnel plot if possible, or based on available information from clinical trial registries.

^f^Certainty of evidence: studies began as ‘high’ quality. Studies with ‘yes’ in domain ‘a’ (risk of bias) were downgraded 2 levels. Studies were downgraded one level if ‘yes’ in domains b-e. High quality: we are very confident that the true effect lies close to that of the estimate of the effect. Moderate quality: we are moderately confident in the effect estimate; the true effect is likely to be close to the estimate of the effect, but there is a possibility that it is substantially different. Low quality: our confidence in the effect estimate is limited: the true effect may be substantially different from the estimate of the effect. Very low quality: we have very little confidence in the effect estimate: the true effect is likely to be substantially different from the estimate of effect.

^g^Unbalanced baseline and no adjustment in analysis, unclear co-intervention

^h^Unclear allocation concealment; not possible to blind patients or treatment providers; unclear blinding of data analyst; unclear group differences at baseline; unclear co-intervention; unclear intention-to-treat analysis

^i^Unclear randomization sequence generation; inadequate allocation concealment; unclear blinding (patients, treatment providers, outcome assessors, data analyst); unclear group differences at baseline; unclear adjustment of baseline group differences in analysis; unclear co-interventions; unclear outcome measures (procedure, reliability, validity); unclear intention to treat analysis

^j^Unclear randomization sequence generation; inadequate allocation concealment; not possible to blind patients or treatment providers; unclear blinding (outcome assessors, data analyst); unclear group differences at baseline; group differences not adjusted for in analysis; unclear co-interventions; follow-up not reported; unclear handling of missing data; unclear intention to treat analysis

^k^Unclear randomization sequence generation; inadequate allocation concealment; unclear blinding (patients, treatment providers, data analyst); unclear handling of missing data

^l^Unclear allocation concealment; not possible to blind patients or treatment providers; unclear blinding of data analyst; unclear group differences at baseline; unclear co-intervention; drop-out rates not reported by group; unclear intention-to-treat analysis; unclear comparability of results across sites

^m^Unclear randomization sequence generation; no information of allocation concealment; not possible to blind patients, treatment providers, outcome assessors; unclear blinding of data analyst; no information of group differences at baseline; unclear if group differences adjusted for in analysis; unclear co-interventions; unclear handling of missing data; unclear intention-to-treat analysis; unclear comparability of results across sites

^n^We cannot evaluate imprecision domain based on p value or mean difference. Therefore, we did not GRADE Skall et al. (1994) for the outcomes of function and low back pain, Choi et al. (2005) for function outcome, and Hakkinen et al. (2005) for function, low back pain and leg pain.

*Calculated by review authors based on raw data provided in RCT

^+^ 95% CI is not reported in the RCT and the review authors cannot calculate 95% CI due to lack of raw data

**File 9.3: GRADE evidence profile of acupuncture vs. control (e.g., usual care, no intervention, wait-list control) for patients who were treated surgically for lumbar disc herniation from 1 randomized controlled trial**

| **Study** | **Outcome Measure** | **Risk of bias^a^** | **Imprecision^b^** | **Indirectness^c^** | **Inconsistency^d^** | **Publication bias detected?^e^** | **Certainty of evidence^f^** | **Intervention (no. of patients)** | **Comparison (no. of patients)** | **Between-group effect (mean difference (MD) or risk ratio (RR) (95% CI))** |
| --- | --- | --- | --- | --- | --- | --- | --- | --- | --- | --- |
| **Function (short-term follow up: > 1 week – 3 months)** | | | | | | | | | | |
| Zhao et al. (2008)^335^ | Modified JOA, 0-15;  lower is better  10% for between-group difference=1.5 | Very serious^g^ | Serious | Not serious | Not serious | No | Very low | N=35 | N=34 | MD 1.6 (1.0, 2.3)* |
| **Function (intermediate follow up: > 3 months - 1 year)** | | | | | | | | | | |
| Zhao et al. (2008)^335^ | Modified JOA, 0-15;  lower is better  10% for between-group difference=1.5 | Very serious^g^ | Serious | Not serious | Not serious | No | Very low | N=35 | N=34 | MD 1.6 (0.5, 2.8)* |

**CI:** confidence interval, **JOA:** Japanese Orthopedic Association, **MD:** mean difference

^a^Risk of bias: Options are not serious, serious (rate certainty of evidence down one level), and very serious (rate certainty of evidence down two levels). 1. Not serious: study rated as ‘low risk of bias’ or ‘some concerns’ (e.g., unclear co-interventions, no detailed randomization method described but similar baseline characteristics between groups). 2. Serious: study rated as ‘high risk of bias’ with unbalanced baseline characteristics between groups, unclear co-interventions, high/unbalanced drop-out and/or unclear intention-to-treat analysis. 3. Very serious: study rated as ‘high risk of bias’ with unclear randomization sequence generation, inadequate allocation concealment and/or lack of blinding.

^b^Imprecision: Options are not serious, serious (rate certainty of evidence down one level), and very serious (rate certainty of evidence down two levels). Imprecision assessed using between-group effect [point estimate (95% CI)]. 1. Not serious: If the point estimate is not clinically important: the upper and lower boundaries of the CI do not cross a clinically important threshold; the CI may cross the null as long as neither boundary crosses a clinically important threshold. If the point estimate is clinically important: the CI does not cross the null and the boundaries do not cross a clinically important threshold. 2. Serious: If the point estimate is not clinically important: the CI may or may not cross the null but one of the boundaries crosses a clinically important threshold. If the point estimate is clinically important: the CI may cross the null but does not cross a clinically important threshold in the other direction. 3. Very serious: If the point estimate is or is not clinically important: the CI crosses the boundaries of both appreciable harm and benefit (i.e., very wide CI).

^c^Indirectness: Options are not serious, serious (rate certainty of evidence down one level), and very serious (rate certainty of evidence down two levels). Indirectness assessed whether the patients, interventions, or outcomes are different from the research question under investigation.

^d^Inconsistency: Options are not serious, serious (rate certainty of evidence down one level), and very serious (rate certainty of evidence down two levels). Inconsistency assessed effect estimate variance in direction or magnitude. 1. Not serious: effect estimates are consistent in direction and magnitude across studies. 2. Serious: effect estimates vary in magnitude across studies and the heterogeneity could not be explained. 3. Very serious: effect estimates vary in direction across studies and the heterogeneity could not be explained. We rate inconsistency as not serious when there is only one single RCT for an outcome; however, there are no other trials with which to compare findings.

^e^Publication bias assessed using funnel plot if possible, or based on available information from clinical trial registries.

^f^Certainty of evidence: Options are *high, moderate, low*, or *very low*. Studies began *high* and were downgraded one level if ‘serious’ or two levels if ‘very serious’ in domains b-e. *High:* we are very confident that the true effect lies close to that of the estimate of the effect. *Moderate:* we are moderately confident in the effect estimate; the true effect is likely to be close to the estimate of the effect, but there is a possibility that it is substantially different. *Low:* our confidence in the effect estimate is limited: the true effect may be substantially different from the estimate of the effect. *Very low:* we have very little confidence in the effect estimate: the true effect is likely to be substantially different from the estimate of effect.

^g^Unclear randomization sequence generation; inadequate allocation concealment; not possible to blind patients and treatment providers; unclear blinding (outcome assessors, data analyst); unclear co-interventions; follow-up not reported; unclear handling of missing data; unclear intention-to-treat analysis.

*Calculated by review authors based on raw data provided in RCT

^+^ 95% CI is not reported in the RCT and the review authors cannot calculate 95% CI due to lack of raw data

**File 9.4: GRADE evidence profile of psychological interventions vs. another intervention for patients who were treated surgically for lumbar disc herniation from 1 randomized controlled trial**

| **Study** | **Outcome Measure** | **Risk of bias^a^** | **Imprecision^b^** | **Indirectness^c^** | **Inconsistency^d^** | **Publication bias detected?^e^** | **Certainty of evidence^f^** | **Intervention (no. of patients)** | **Comparison (no. of patients)** | **Between-group effect (mean difference (MD) or risk ratio (RR) (95% CI))** |
| --- | --- | --- | --- | --- | --- | --- | --- | --- | --- | --- |
| **Function (intermediate term follow up: > 3 months to 1 year)** | | | | | | | | | | |
| Ostelo et al. (2003, 2003)^327, 328^ | RMDQ, 0-24; lower is better  10% for between-group difference=2.4 | Not serious | Serious | Not serious | Not serious | No | Moderate | N=52 | N=53 | MD -0.3 (-2.5, 2.0) |
| **Function (long-term follow up: > 1 year)** | | | | | | | | | | |
| Ostelo et al. (2003, 2003)^327, 328^ | RMDQ, 0-24; lower is better  10% for between-group difference=2.4 | Not serious | Not serious | Not serious | Not serious | No | High | N=52 | N=53 | MD 0.0 (-2.1, 2.1) |
| **Low back pain (intermediate term follow up: > 3 months to 1 year)** | | | | | | | | | | |
| Ostelo et al. (2003, 2003)^327, 328^ | VAS, 100mm;  lower is better  (pain location unspecified)  10% for between-group difference=10 | Not serious | Serious | Not serious | Not serious | No | Moderate | N=52 | N=53 | MD 7.2 (-4.9, 19.4) |
| **Low back pain (long-term follow up: > 1 year)** | | | | | | | | | | |
| Ostelo et al. (2003, 2003)^327, 328^ | VAS, 100mm;  lower is better  10% for between-group difference=10 | Not serious | Serious | Not serious | Not serious | No | Moderate | N=52 | N=53 | MD 4.8 (-7.9, 17.5) |
| **Leg pain (intermediate term follow up: > 3 months to 1 year)** | | | | | | | | | | |
| Ostelo et al. (2003, 2003)^327, 328^ | VAS, 100mm;  lower is better  10% for between-group difference=10 | Not serious | Serious | Not serious | Not serious | No | Moderate | N=52 | N=53 | MD 2.9 (-7.5, 13.3) |
| **Leg pain (long-term follow up: > 1 year)** | | | | | | | | | | |
| Ostelo et al. (2003, 2003)^327, 328^ | VAS, 100mm;  lower is better  10% for between-group difference=10 | Not serious | Serious | Not serious | Not serious | No | Moderate | N=52 | N=53 | MD 2.3 (-9.8, 14.4) |
| **Fear of movement (intermediate term follow up: > 3 months to 1 year)** | | | | | | | | | | |
| Ostelo et al. (2003, 2003)^327, 328^ | TSK, 17-68;  lower is better  10% for between-group difference=5.1 | Not serious | Not serious | Not serious | Not serious | No | High | N=52 | N=53 | MD -1.1 (-3.6, 1.4) |
| **Fear of movement (long-term follow up: > 1 year)** | | | | | | | | | | |
| Ostelo et al. (2003, 2003)^327, 328^ | TSK, 17-68;  lower is better  10% for between-group difference=5.1 | Not serious | Not serious | Not serious | Not serious | No | High | N=52 | N=53 | MD 0.0 (-2.5, 2.4) |
| **Pain Catastrophizing (intermediate term follow up: > 3 months to 1 year)** | | | | | | | | | | |
| Ostelo et al. (2003, 2003)^327, 328^ | PCS, 0-52;  higher is better  10% for between-group difference=5.2 | Not serious | Not serious | Not serious | Not serious | No | High | N=52 | N=53 | MD -0.3 (-3.6, 3.0) |
| **Pain Catastrophizing (long-term follow up: > 1 year)** | | | | | | | | | | |
| Ostelo et al. (2003, 2003)^327, 328^ | PCS, 0-52;  higher is better  10% for between-group difference=5.2 | Not serious | Serious | Not serious | Not serious | No | Moderate | N=52 | N=53 | MD -3.3 (-6.3, -0.2) |
| **Recovery (intermediate term follow up: > 3 months to 1 year)** | | | | | | | | | | |
| Ostelo et al. (2003, 2003)^327, 328^ | GPE, 7-point Likert scale (% improved) | Not serious | Very serious | Not serious | Not serious | No | Low | N=52 | N=53 | MD 3.0 (-16.3, 22.4) |
| **Recovery (long-term follow up: > 1 year)** | | | | | | | | | | |
| Ostelo et al. (2003, 2003)^327, 328^ | GPE, 7-point Likert scale (% improved) | Not serious | Very serious | Not serious | Not serious | No | Low | N=52 | N=53 | MD 2.0 (-19.5, 15.7) |
| **Quality of life (intermediate term follow up: > 3 months to 1 year)** | | | | | | | | | | |
| Ostelo et al. (2003, 2003)^327, 328^ | SF-36, 0-100;  higher is better  General health  Social functioning  10% for between-group difference=10 | Not serious  Not serious | Not serious  Serious | Not serious  Not serious | Not serious  Not serious | No  No | High  Moderate | N=52 | N=53 | MD -3.8 (-9.7, 2.0)  MD -4.3 (-14.2, 5.6) |
| **Quality of life (long-term follow up: > 1 year)** | | | | | | | | | | |
| Ostelo et al. (2003, 2003)^327, 328^ | SF-36, 0-100;  higher is better  General health  Social functioning  10% for between-group difference=10 | Not serious  Not Serious | Not serious  Serious | Not serious  Not serious | Not serious  Not serious | No  No | High  Moderate | N=52 | N=53 | MD -2.2 (-8.6, 4.2)  MD 4.3 (-14.5, 5.9) |
| **Analgesic use (long-term follow up: > 1 year)** | | | | | | | | | | |
| Ostelo et al. (2003, 2003)^327, 328^ | Yes/no | Not serious | Very serious | Not serious | Not serious | No | Low | N=52 | N=53 | RR 0.70 (0.36, 1.36)*  Participants in intervention group are 30% less likely to take analgesics. |
| **General practitioner visits (long-term follow up: > 1 year)** | | | | | | | | | | |
| Ostelo et al. (2003, 2003)^327, 328^ | Yes/no | Not serious | Very serious | Not serious | Not serious | No | Low | N=52 | N=53 | RR 0.91 (0.52, 1.58)*  Participants in intervention group are 9% less likely to visit general practitioners. |
| **Specialist visits (long-term follow up: > 1 year)** | | | | | | | | | | |
| Ostelo et al. (2003, 2003)^327, 328^ | Yes/no | Not serious | Very serious | Not serious | Not serious | No | Low | N=52 | N=53 | RR 1.17 (0.46, 2.98)*  Participants in intervention group are 1.17 times more likely to visit specialists |
| **Physiotherapist visits (long-term follow up: > 1 year)** | | | | | | | | | | |
| Ostelo et al. (2003, 2003)^327, 328^ | Yes/no | Not serious | Very serious | Not serious | Not serious | No | Low | N=52 | N=53 | RR 0.85 (0.28, 2.61)*  Participants in intervention group are 15% less likely to visit physiotherapists. |
| **Work outcomes (intermediate follow up: >3 months to 1 year)** | | | | | | | | | | |
| Ostelo et al. (2003, 2003)^327, 328^ | RTW Yes/no | Not serious | Serious | Not serious | Not serious | No | Moderate | N=52 | N=53 | p=0.10; RR 0.82 (0.66, 1.01)*  Participants in intervention group are 18% less likely to return to work |
| **Work outcomes (long-term follow up: > 1 year)** | | | | | | | | | | |
| Ostelo et al. (2003, 2003)^327, 328^ | RTW Yes/no | Not serious | Serious | Not serious | Not serious | No | Moderate | N=52 | N=53 | p=0.61; RR 0.82 (0.68, 1.00)*  Participants in intervention group are 18% less likely to return to work. |
| **Re-operation (intermediate follow up: >3 months to 1 year)** | | | | | | | | | | |
| Ostelo et al. (2003, 2003)^327, 328^ | Yes/no | Not serious | Very serious | Not serious | Not serious | No | Low | N=52 | N=53 | RR 1.04 (0.07, 16.18)*  Participants in intervention group are 1.04 times more likely to have re-operation |
| **Re-operation (long-term follow up: > 1 year)** | | | | | | | | | | |
| Ostelo et al. (2003, 2003)^327, 328^ | Yes/no | Not serious | Very serious | Not serious | Not serious | No | Low | N=52 | N=53 | RR 2.04 (0.39, 10.65)*  Participants in intervention group are 2.04 times more likely to have re-operation |

**CI:** confidence interval, **MD:** mean difference, **RMDQ:** Roland-Morris Disability Questionnaire, **VAS:** Visual Analogue Scale

^a^Risk of bias: Options are not serious, serious (rate certainty of evidence down one level), and very serious (rate certainty of evidence down two levels). 1. Not serious: study rated as ‘low risk of bias’ or ‘some concerns’ (e.g., unclear co-interventions, no detailed randomization method described but similar baseline characteristics between groups). 2. Serious: study rated as ‘high risk of bias’ with unbalanced baseline characteristics between groups, unclear co-interventions, high/unbalanced drop-out and/or unclear intention-to-treat analysis. 3. Very serious: study rated as ‘high risk of bias’ with unclear randomization sequence generation, inadequate allocation concealment and/or lack of blinding.

^b^Imprecision: Options are not serious, serious (rate certainty of evidence down one level), and very serious (rate certainty of evidence down two levels). Imprecision assessed using between-group effect [point estimate (95% CI)]. 1. Not serious: If the point estimate is not clinically important: the upper and lower boundaries of the CI do not cross a clinically important threshold; the CI may cross the null as long as neither boundary crosses a clinically important threshold. If the point estimate is clinically important: the CI does not cross the null and the boundaries do not cross a clinically important threshold. 2. Serious: If the point estimate is not clinically important: the CI may or may not cross the null but one of the boundaries crosses a clinically important threshold. If the point estimate is clinically important: the CI may cross the null but does not cross a clinically important threshold in the other direction. 3. Very serious: If the point estimate is or is not clinically important: the CI crosses the boundaries of both appreciable harm and benefit (i.e., very wide CI).

^c^Indirectness: Options are not serious, serious (rate certainty of evidence down one level), and very serious (rate certainty of evidence down two levels). Indirectness assessed whether the patients, interventions, or outcomes are different from the research question under investigation.

^d^Inconsistency: Options are not serious, serious (rate certainty of evidence down one level), and very serious (rate certainty of evidence down two levels). Inconsistency assessed effect estimate variance in direction or magnitude. 1. Not serious: effect estimates are consistent in direction and magnitude across studies. 2. Serious: effect estimates vary in magnitude across studies and the heterogeneity could not be explained. 3. Very serious: effect estimates vary in direction across studies and the heterogeneity could not be explained. We rate inconsistency as not serious when there is only one single RCT for an outcome; however, there are no other trials with which to compare findings.

^e^Publication bias assessed using funnel plot if possible, or based on available information from clinical trial registries.

^f^Certainty of evidence: Options are *high, moderate, low*, or *very low*. Studies began *high* and were downgraded one level if ‘serious’ or two levels if ‘very serious’ in domains b-e. *High:* we are very confident that the true effect lies close to that of the estimate of the effect. *Moderate:* we are moderately confident in the effect estimate; the true effect is likely to be close to the estimate of the effect, but there is a possibility that it is substantially different. *Low:* our confidence in the effect estimate is limited: the true effect may be substantially different from the estimate of the effect. *Very low:* we have very little confidence in the effect estimate: the true effect is likely to be substantially different from the estimate of effect.

*Calculated by review authors based on raw data provided in RCT

^+^ 95% CI is not reported in the RCT and the review authors cannot calculate 95% CI due to lack of raw data

**File 9.5: GRADE evidence profile of whole-body magnetic field therapy vs. another intervention for patients who were treated surgically for lumbar disc herniation from 1 randomized controlled trial**

| **Study** | **Outcome Measure** | **Risk of bias^a^** | **Imprecision^b^** | **Indirectness^c^** | **Inconsistency^d^** | **Publication bias^e^** | **Certainty of evidence^f^** | **Intervention (no. of patients, within- group effect [mean (95%CI)]^‡^)** | **Comparison (no. of patients)** | **Between-group effect (95% CI)** |
| --- | --- | --- | --- | --- | --- | --- | --- | --- | --- | --- |
| **Pain in lumbar area (short-term follow up: > 1 week – 3 months)** | | | | | | | | | | |
| Kulikov et al. (2018)^323^ | VAS, 10cm;  lower is better  10% for between-group difference=1 | Not serious | Not serious | Not serious | Not serious | No | High | N=37  3 (2.94, 3.06)* | N=36 | MD 1.2 (1.09, 1.31)* |

**CI:** confidence interval, **MD:** mean difference, **VAS:** Visual Analogue Scale

^a^Risk of bias: Options are not serious, serious (rate certainty of evidence down one level), and very serious (rate certainty of evidence down two levels). 1. Not serious: study rated as ‘low risk of bias’ or ‘some concerns’ (e.g., unclear co-interventions, no detailed randomization method described but similar baseline characteristics between groups). 2. Serious: study rated as ‘high risk of bias’ with unbalanced baseline characteristics between groups, unclear co-interventions, high/unbalanced drop-out and/or unclear intention-to-treat analysis. 3. Very serious: study rated as ‘high risk of bias’ with unclear randomization sequence generation, inadequate allocation concealment and/or lack of blinding.

^b^Imprecision: Options are not serious, serious (rate certainty of evidence down one level), and very serious (rate certainty of evidence down two levels). Imprecision assessed using between-group effect [point estimate (95% CI)]. 1. Not serious: If the point estimate is not clinically important: the upper and lower boundaries of the CI do not cross a clinically important threshold; the CI may cross the null as long as neither boundary crosses a clinically important threshold. If the point estimate is clinically important: the CI does not cross the null and the boundaries do not cross a clinically important threshold. 2. Serious: If the point estimate is not clinically important: the CI may or may not cross the null but one of the boundaries crosses a clinically important threshold. If the point estimate is clinically important: the CI may cross the null but does not cross a clinically important threshold in the other direction. 3. Very serious: If the point estimate is or is not clinically important: the CI crosses the boundaries of both appreciable harm and benefit (i.e., very wide CI).

^c^Indirectness: Options are not serious, serious (rate certainty of evidence down one level), and very serious (rate certainty of evidence down two levels). Indirectness assessed whether the patients, interventions, or outcomes are different from the research question under investigation.

^d^Inconsistency: Options are not serious, serious (rate certainty of evidence down one level), and very serious (rate certainty of evidence down two levels). Inconsistency assessed effect estimate variance in direction or magnitude. 1. Not serious: effect estimates are consistent in direction and magnitude across studies. 2. Serious: effect estimates vary in magnitude across studies and the heterogeneity could not be explained. 3. Very serious: effect estimates vary in direction across studies and the heterogeneity could not be explained. We rate inconsistency as not serious when there is only one single RCT for an outcome; however, there are no other trials with which to compare findings.

^e^Publication bias assessed using funnel plot if possible, or based on available information from clinical trial registries.

^f^Certainty of evidence: Options are *high, moderate, low*, or *very low*. Studies began *high* and were downgraded one level if ‘serious’ or two levels if ‘very serious’ in domains b-e. *High:* we are very confident that the true effect lies close to that of the estimate of the effect. *Moderate:* we are moderately confident in the effect estimate; the true effect is likely to be close to the estimate of the effect, but there is a possibility that it is substantially different. *Low:* our confidence in the effect estimate is limited: the true effect may be substantially different from the estimate of the effect. *Very low:* we have very little confidence in the effect estimate: the true effect is likely to be substantially different from the estimate of effect.

*Calculated by review authors based on raw data provided in RCT

^+^ 95% CI is not reported in the RCT and the review authors cannot calculate 95% CI due to lack of raw data

**File 9.6: GRADE evidence profile of education/self-management vs. another intervention for patients who were treated surgically for lumbar disc herniation from 2 randomized controlled trials**

| **Study** | **Outcome Measure** | **Risk of bias^a^** | **Imprecision^b^** | **Indirectness^c^** | **Inconsistency^d^** | **Publication bias^e^** | **Certainty of evidence^f^** | **Intervention (no. of patients)** | **Comparison (no. of patients)** | **Between-group effect (mean difference (MD) or risk ratio (RR) (95% CI))** |
| --- | --- | --- | --- | --- | --- | --- | --- | --- | --- | --- |
| **Function (short-term follow up: > 1 week - 3 months)** | | | | | | | | | | |
| Bono et al. (2017)^314^ | ODI, 0-100; lower is better  10% for between-group difference=10 | Very serious^g^ | Not serious | Not serious | Not serious | No | Low | N=53 | N=55 | MD 0.5 (-3.68, 4.68) |
| Erdogan and Bulut (2019)^316^ | ODI, 0-100; lower is better  10% for between-group difference=10 | Very serious^h^ | Serious | Not serious | Not serious | No | Very low | N=31 | N=31 | MD 8.69 (3.12, 14.26)* |
| **Function (intermediate follow up: >3 months - 1 year)** | | | | | | | | | | |
| Bono et al. (2017)^314^ | ODI, 0-100; lower is better  10% for between-group difference=10 | Very serious^g^ | Not serious | Not serious | Not serious | No | Low | N=53 | N=55 | MD -1.5 (-5.78, 2.78) |
| **Function (long-term follow up: > 1 year)** | | | | | | | | | | |
| Bono et al. (2017)^314^ | ODI, 0-100; lower is better  10% for between-group difference=10 | Very serious^g^ | Not serious | Not serious | Not serious | No | Low | N=53 | N=55 | MD -2.1 (-6.54, 2.34) |
| **Back pain (short-term follow up: > 1 week - 3 months)** | | | | | | | | | | |
| Bono et al. (2017)^314^ | VAS, 100mm;  lower is better  10% for between-group difference=10 | Very serious^g^ | Serious | Not serious | Not serious | No | Very low | N=53 | N=55 | MD -5 (-11.85, 1.85) |
| **Back pain (intermediate follow up: >3 months - 1 year)** | | | | | | | | | | |
| Bono et al. (2017)^314^ | VAS, 100mm;  lower is better  10% for between-group difference=10 | Very serious^g^ | Serious | Not serious | Not serious | No | Very low | N=53 | N=55 | MD -8.7 (-15.58, -1.82) |
| **Back pain (long-term follow up: > 1 year)** | | | | | | | | | | |
| Bono et al. (2017)^314^ | VAS, 100mm; lower is better  10% for between-group difference=10 | Very serious^g^ | Serious | Not serious | Not serious | No | Very low | N=53 | N=55 | MD -7.5 (-13.86, -1.14) |
| **Leg pain (short-term follow up: > 1 week - 3 months)** | | | | | | | | | | |
| Bono et al. (2017)^314^ | VAS, 100mm;  lower is better  10% for between-group difference=10 | Very serious^g^ | Serious | Not serious | Not serious | No | Very low | N=53 | N=55 | MD 4.7 (-0.63, 10.03) |
| **Leg pain (intermediate follow up: >3 months - 1 year)** | | | | | | | | | | |
| Bono et al. (2017)^314^ | VAS, 100mm;  lower is better  10% for between-group difference=10 | Very serious^g^ | Not serious | Not serious | Not serious | No | Low | N=53 | N=55 | MD -0.3 (-5.78, 5.18) |
| **Leg pain (long-term follow up: > 1 year)** | | | | | | | | | | |
| Bono et al. (2017)^314^ | VAS, 100mm;  lower is better  10% for between-group difference=10 | Very serious^g^ | Serious | Not serious | Not serious | No | Very low | N=53 | N=55 | MD -6.6 (-12.38, -0.82) |
| **Anxiety (short-term follow up: > 1 week - 3 months)** | | | | | | | | | | |
| Erdogan and Bulut (2019)^316^ | STAI, 20-80;  higher is better  10% for between-group difference=6 | Very serious^h^  Very serious^h^ | Not serious  Not serious | Not serious  Not serious | Not serious  Not serious | No  No | Low  Low | N=31 | N=31 | State Anxiety: MD 10.49 (6.59, 14.39) *  Trait Anxiety: MD 2.14 (-0.08, 4.36)* |

**CI:** confidence interval, **MD:** mean difference, **ODI:** Oswestry Disability Index, **STAI:** State-Trait Anxiety Inventory, **VAS:** Visual Analogue Scale

^a^Risk of bias: Options are not serious, serious (rate certainty of evidence down one level), and very serious (rate certainty of evidence down two levels). 1. Not serious: study rated as ‘low risk of bias’ or ‘some concerns’ (e.g., unclear co-interventions, no detailed randomization method described but similar baseline characteristics between groups). 2. Serious: study rated as ‘high risk of bias’ with unbalanced baseline characteristics between groups, unclear co-interventions, high/unbalanced drop-out and/or unclear intention-to-treat analysis. 3. Very serious: study rated as ‘high risk of bias’ with unclear randomization sequence generation, inadequate allocation concealment and/or lack of blinding.

^b^Imprecision: Options are not serious, serious (rate certainty of evidence down one level), and very serious (rate certainty of evidence down two levels). Imprecision assessed using between-group effect [point estimate (95% CI)]. 1. Not serious: If the point estimate is not clinically important: the upper and lower boundaries of the CI do not cross a clinically important threshold; the CI may cross the null as long as neither boundary crosses a clinically important threshold. If the point estimate is clinically important: the CI does not cross the null and the boundaries do not cross a clinically important threshold. 2. Serious: If the point estimate is not clinically important: the CI may or may not cross the null but one of the boundaries crosses a clinically important threshold. If the point estimate is clinically important: the CI may cross the null but does not cross a clinically important threshold in the other direction. 3. Very serious: If the point estimate is or is not clinically important: the CI crosses the boundaries of both appreciable harm and benefit (i.e., very wide CI).

^c^Indirectness: Options are not serious, serious (rate certainty of evidence down one level), and very serious (rate certainty of evidence down two levels). Indirectness assessed whether the patients, interventions, or outcomes are different from the research question under investigation.

^d^Inconsistency: Options are not serious, serious (rate certainty of evidence down one level), and very serious (rate certainty of evidence down two levels). Inconsistency assessed effect estimate variance in direction or magnitude. 1. Not serious: effect estimates are consistent in direction and magnitude across studies. 2. Serious: effect estimates vary in magnitude across studies and the heterogeneity could not be explained. 3. Very serious: effect estimates vary in direction across studies and the heterogeneity could not be explained. We rate inconsistency as not serious when there is only one single RCT for an outcome; however, there are no other trials with which to compare findings.

^e^Publication bias assessed using funnel plot if possible, or based on available information from clinical trial registries.

^f^Certainty of evidence: Options are *high, moderate, low*, or *very low*. Studies began *high* and were downgraded one level if ‘serious’ or two levels if ‘very serious’ in domains b-e. *High:* we are very confident that the true effect lies close to that of the estimate of the effect. *Moderate:* we are moderately confident in the effect estimate; the true effect is likely to be close to the estimate of the effect, but there is a possibility that it is substantially different. *Low:* our confidence in the effect estimate is limited: the true effect may be substantially different from the estimate of the effect. *Very low:* we have very little confidence in the effect estimate: the true effect is likely to be substantially different from the estimate of effect.

^g^ Unclear randomization sequence generation; not possible to blind patients and treatment providers; unclear blinding (outcome assessors, data analyst); unclear co-intervention; >35% loss to follow-up; unclear handling of missing data; unclear intention-to-treat analysis; unclear comparability of results across sites

^h^Unclear treatment allocation; not possible to blind patients and treatment providers; unclear blinding (outcome assessors, data analyst); group differences at baseline; group differences not adjusted for in analysis; unclear co-interventions.

*Calculated by review authors based on raw data provided in RCT

^+^ 95% CI is not reported in the RCT and the review authors cannot calculate 95% CI due to lack of raw data

**File 9.7: GRADE evidence profile of multimodal interventions (education + exercise without psychological component) vs. sham/placebo for patients who were treated surgically for lumbar disc herniation from 1 randomized controlled trial** (2 articles)

| **Study** | **Outcome Measure** | **Risk of bias^a^** | **Imprecision^b^** | **Indirectness^c^** | **Inconsistency^d^** | **Publication bias detected?^e^** | **Certainty of evidence^f^** | **Intervention (no. of patients)** | **Comparison (no. of patients)** | **Between-group effect (mean difference (MD) or risk ratio (RR) (95% CI))** |
| --- | --- | --- | --- | --- | --- | --- | --- | --- | --- | --- |
| **Low back pain (short term follow up: > 1 week to 3 months)** | | | | | | | | | | |
| Erdogmus et al. (2007)^317^ | LBPRS, 0-130;  lower is better  10% for between-group difference=13 | Not serious | Not serious | Not serious | Not serious | No | High | N=40 | N=40 | MD -0.59 (-8.22, 7.04) |
| **Low back pain (intermediate term follow up: > 3 months to 1 year)** | | | | | | | | | | |
| Erdogmus et al. (2007)^317^ | LBPRS, 0-130;  lower is better  10% for between-group difference=13 | Not serious | Not serious | Not serious | Not serious | No | High | N=40 | N=40 | MD 4.14 (-3.69, 11.98) |
| **Low back pain (long-term follow up: > 1 year)** | | | | | | | | | | |
| Erdogmus et al. (2007)^317^ | LBPRS, 0-130;  lower is better  10% for between-group difference=13 | Not serious | Not serious | Not serious | Not serious | No | High | N=40 | N=40 | MD -1.49 (-11.92, 8.94) |
| Ebenbichler et al. (2015)^318^ | LBPRS, 0-130;  lower is better  10% for between-group difference=13 | Not serious | Serious | Not serious | Not serious | No | Moderate | N=40 | N=40 | MD -2.7 (-13.2, 7.8)  Equal |
| **Back pain/sciatica/both (long-term follow up: > 1 year)** | | | | | | | | | | |
| Erdogmus et al. (2007)^317^ | Often/ permanent vs. never/sporadic/often always | Not serious | Very serious | Not serious | Not serious | No | Low | N=40 | N=40 | RR 0.9 (0.8, 1.2)*  Participants in intervention group are 10% less likely to have back pain/sciatica/both |

**CI:** confidence interval, **LBPRS:** Low Back Pain Rating Scale, **MD:** mean difference, **NRS:** Numeric Rating Scale, **ODI:** Oswestry Disability Index, **RMDQ:** Roland-Morris Disability Questionnaire, **VAS:** Visual Analogue Scale

^a^Risk of bias: Options are not serious, serious (rate certainty of evidence down one level), and very serious (rate certainty of evidence down two levels). 1. Not serious: study rated as ‘low risk of bias’ or ‘some concerns’ (e.g., unclear co-interventions, no detailed randomization method described but similar baseline characteristics between groups). 2. Serious: study rated as ‘high risk of bias’ with unbalanced baseline characteristics between groups, unclear co-interventions, high/unbalanced drop-out and/or unclear intention-to-treat analysis. 3. Very serious: study rated as ‘high risk of bias’ with unclear randomization sequence generation, inadequate allocation concealment and/or lack of blinding.

^b^Imprecision: Options are not serious, serious (rate certainty of evidence down one level), and very serious (rate certainty of evidence down two levels). Imprecision assessed using between-group effect [point estimate (95% CI)]. 1. Not serious: If the point estimate is not clinically important: the upper and lower boundaries of the CI do not cross a clinically important threshold; the CI may cross the null as long as neither boundary crosses a clinically important threshold. If the point estimate is clinically important: the CI does not cross the null and the boundaries do not cross a clinically important threshold. 2. Serious: If the point estimate is not clinically important: the CI may or may not cross the null but one of the boundaries crosses a clinically important threshold. If the point estimate is clinically important: the CI may cross the null but does not cross a clinically important threshold in the other direction. 3. Very serious: If the point estimate is or is not clinically important: the CI crosses the boundaries of both appreciable harm and benefit (i.e., very wide CI).

^c^Indirectness: Options are not serious, serious (rate certainty of evidence down one level), and very serious (rate certainty of evidence down two levels). Indirectness assessed whether the patients, interventions, or outcomes are different from the research question under investigation.

^d^Inconsistency: Options are not serious, serious (rate certainty of evidence down one level), and very serious (rate certainty of evidence down two levels). Inconsistency assessed effect estimate variance in direction or magnitude. 1. Not serious: effect estimates are consistent in direction and magnitude across studies. 2. Serious: effect estimates vary in magnitude across studies and the heterogeneity could not be explained. 3. Very serious: effect estimates vary in direction across studies and the heterogeneity could not be explained. We rate inconsistency as not serious when there is only one single RCT for an outcome; however, there are no other trials with which to compare findings.

^e^Publication bias assessed using funnel plot if possible, or based on available information from clinical trial registries.

^f^Certainty of evidence: studies began as ‘high’ quality. Studies with ‘yes’ in domain ‘a’ (risk of bias) were downgraded 2 levels. Studies were downgraded one level if ‘yes’ in domains b-e.High quality: we are very confident that the true effect lies close to that of the estimate of the effect. Moderate quality: we are moderately confident in the effect estimate; the true effect is likely to be close to the estimate of the effect, but there is a possibility that it is substantially different. Low quality: our confidence in the effect estimate is limited: the true effect may be substantially different from the estimate of the effect. Very low quality: we have very little confidence in the effect estimate: the true effect is likely to be substantially different from the estimate of effect.

^g^Same RCT as Erdogmus 2007

*Calculated by review authors based on raw data provided in RCT

^+^ 95% CI is not reported in the RCT and the review authors cannot calculate 95% CI due to lack of raw data

**File 9.8: GRADE evidence profile of multimodal interventions (without psychological component) vs. control (e.g., usual care, no intervention, wait-list control) for patients who were treated surgically for lumbar disc herniation from 2 randomized controlled trials (3 articles)**

| **Study** | **Outcome Measure** | **Risk of bias^a^** | **Imprecision^b^** | **Indirectness^c^** | **Inconsistency^d^** | **Publication bias detected?^e^** | **Certainty of evidence^f^** | **Intervention (no. of patients)** | **Comparison (no. of patients)** | **Between-group effect (mean difference (MD) or risk ratio (RR) (95% CI))** |
| --- | --- | --- | --- | --- | --- | --- | --- | --- | --- | --- |
| **Function (short term follow up: > 1 week to 3 months)** | | | | | | | | | | |
| Oosterhuis et al. (2017)^326^ | ODI, 0-100; lower is better  10% for between-group difference=10 | Not serious | Not serious | Not serious | Not serious | No | High | N=92 | N=77 | MD -3.7 (-6.89, -0.51)* |
| **Function (intermediate term follow up: > 3 months to 1 year)** | | | | | | | | | | |
| Oosterhuis et al. (2017)^326^ | ODI, 0-100; lower is better  10% for between-group difference=10 | Not serious | Not serious | Not serious | Not serious | No | High | N=92 | N=77 | MD 1.5 (-3.6, 6.7) |
| **Low back pain (short term follow up: > 1 week to 3 months)** | | | | | | | | | | |
| Erdogmus et al. (2007)^317^ | LBPRS, 0-130;  lower is better  10% for between-group difference=13 | Not serious | Not serious | Not serious | Not serious | No | High | N=40 | N=40 | MD 5.72 (-1.17, 12.61) |
| Oosterhuis et al. (2017)^326^ | NRS, 0-10;  lower is better  10% for between-group difference=1 | Not serious | Not serious | Not serious | Not serious | No | High | N=92 | N=77 | MD -0.1 (-0.59, 0.39)* |
| **Low back pain (intermediate term follow up: > 3 months to 1 year)** | | | | | | | | | | |
| Erdogmus et al. (2007)^317^ | LBPRS, 0-130;  lower is better  10% for between-group difference=13 | Not serious | Serious | Not serious | Not serious | No | Moderate | N=40 | N=40 | MD 11.24 (3.41, 19.07) |
| Oosterhuis et al. (2017)^326^ | NRS, 0-10;  lower is better  10% for between-group difference=1 | Not serious | Not serious | Not serious | Not serious | No | High | N=92 | N=77 | MD 0.3 (-0.3, 0.9) |
| **Low back pain (long-term follow up: > 1 year)** | | | | | | | | | | |
| Erdogmus et al. (2007)^317^ | LBPRS, 0-130; lower is better  10% for between-group difference=13 | Not serious | Serious | Not serious | Not serious | No | Moderate | N=40 | N=40 | MD 8.55 (-2.21, 19.3) |
| **Leg pain (short term follow up: > 1 week to 3 months)** | | | | | | | | | | |
| Oosterhuis et al. (2017)^326^ | NRS, 0-10;  lower is better  10% for between-group difference=1 | Not serious | Not serious | Not serious | Not serious | No | High | N=92 | N=77 | MD -0.1 (-0.59, 0.39)* |
| **Leg pain (intermediate term follow up: > 3 months to 1 year)** | | | | | | | | | | |
| Oosterhuis et al. (2017)^326^ | NRS, 0-10;  lower is better  10% for between-group difference=1 | Not serious | Not serious | Not serious | Not serious | No | High | N=92 | N=77 | MD 0.1 (-0.7, 0.8) |
| **Recovery (short term follow up: > 1 week to 3 months)** | | | | | | | | | | |
| Oosterhuis et al. (2017)^326^ | GPE, 7-point Likert scale | Not serious | Very serious | Not serious | Not serious | No | Low | N=92 | N=77 | OR 0.59 (0.29, 1.17)*  Participants in intervention group are 41% less likely to recover. |
| **Recovery (intermediate term follow up: > 3 months to 1 year)** | | | | | | | | | | |
| Oosterhuis et al. (2017)^326^ | GPE, 7-point Likert scale | Not serious | Very serious | Not serious | Not serious | No | Low | N=92 | N=77 | OR 1.0 (0.6, 1.7)*  Participants in both groups have an equal chance to recover. |
| **Quality of Life (short term follow up: > 1 week to 3 months)** | | | | | | | | | | |
| Oosterhuis et al. (2017)^326^ | SF-12, 0-100;  (Physical)  higher is better  10% for between-group difference=10 | Not serious | serious | Not serious | Not serious | No | Moderate | N=92 | N=77 | MD -3.9 (-10.41, 2.61)* |
| **Quality of Life (intermediate term follow up: > 3 months to 1 year)** | | | | | | | | | | |
| Oosterhuis et al. (2017)^326^ | SF-12, 0-100;  (Physical)  higher is better  10% for between-group difference=10 | Not serious | serious | Not serious | Not serious | No | Moderate | N=92 | N=77 | MD -3.5 (-11.3, 4.3) |
| **Quality of Life (short term follow up: > 1 week to 3 months)** | | | | | | | | | | |
| Oosterhuis et al. (2017)^326^ | SF-12, 0-100;  (Mental)  higher is better  10% for between-group difference=10 | Not serious | Not serious | Not serious | Not serious | No | High | N=92 | N=77 | MD -2.4 (-6.63, 1.83)* |
| **Quality of Life (intermediate term follow up: > 3 months to 1 year)** | | | | | | | | | | |
| Oosterhuis et al. (2017)^326^ | SF-12, 0-100;  (Mental)  higher is better  10% for between-group difference=10 | Not serious | Not serious | Not serious | Not serious | No | High | N=92 | N=77 | MD -4.1 (-9.4, 1.3)* |

**CI:** confidence interval, **GPE:** Global Perceived Effect; **LBPRS:** Low Back Pain Rating Scale, **MD:** mean difference, **NRS:** Numeric Rating Scale, **ODI:** Oswestry Disability Index, **RMDQ:** Roland-Morris Disability Questionnaire, **SF-12:** Short Form-12; **VAS:** Visual Analogue Scale

^a^Risk of bias: Options are not serious, serious (rate certainty of evidence down one level), and very serious (rate certainty of evidence down two levels). 1. Not serious: study rated as ‘low risk of bias’ or ‘some concerns’ (e.g., unclear co-interventions, no detailed randomization method described but similar baseline characteristics between groups). 2. Serious: study rated as ‘high risk of bias’ with unbalanced baseline characteristics between groups, unclear co-interventions, high/unbalanced drop-out and/or unclear intention-to-treat analysis. 3. Very serious: study rated as ‘high risk of bias’ with unclear randomization sequence generation, inadequate allocation concealment and/or lack of blinding.

^b^Imprecision: Options are not serious, serious (rate certainty of evidence down one level), and very serious (rate certainty of evidence down two levels). Imprecision assessed using between-group effect [point estimate (95% CI)]. 1. Not serious: If the point estimate is not clinically important: the upper and lower boundaries of the CI do not cross a clinically important threshold; the CI may cross the null as long as neither boundary crosses a clinically important threshold. If the point estimate is clinically important: the CI does not cross the null and the boundaries do not cross a clinically important threshold. 2. Serious: If the point estimate is not clinically important: the CI may or may not cross the null but one of the boundaries crosses a clinically important threshold. If the point estimate is clinically important: the CI may cross the null but does not cross a clinically important threshold in the other direction. 3. Very serious: If the point estimate is or is not clinically important: the CI crosses the boundaries of both appreciable harm and benefit (i.e., very wide CI).

^c^Indirectness: Options are not serious, serious (rate certainty of evidence down one level), and very serious (rate certainty of evidence down two levels). Indirectness assessed whether the patients, interventions, or outcomes are different from the research question under investigation.

^d^Inconsistency: Options are not serious, serious (rate certainty of evidence down one level), and very serious (rate certainty of evidence down two levels). Inconsistency assessed effect estimate variance in direction or magnitude. 1. Not serious: effect estimates are consistent in direction and magnitude across studies. 2. Serious: effect estimates vary in magnitude across studies and the heterogeneity could not be explained. 3. Very serious: effect estimates vary in direction across studies and the heterogeneity could not be explained. We rate inconsistency as not serious when there is only one single RCT for an outcome; however, there are no other trials with which to compare findings.

^e^Publication bias assessed using funnel plot if possible, or based on available information from clinical trial registries.

^f^Certainty of evidence: studies began as ‘high’ quality. Studies with ‘yes’ in domain ‘a’ (risk of bias) were downgraded 2 levels. Studies were downgraded one level if ‘yes’ in domains b-e.High quality: we are very confident that the true effect lies close to that of the estimate of the effect. Moderate quality: we are moderately confident in the effect estimate; the true effect is likely to be close to the estimate of the effect, but there is a possibility that it is substantially different. Low quality: our confidence in the effect estimate is limited: the true effect may be substantially different from the estimate of the effect. Very low quality: we have very little confidence in the effect estimate: the true effect is likely to be substantially different from the estimate of effect.

^g^Same RCT as Erdogmus 2007.

*Calculated by review authors based on raw data provided in RCT

^+^ 95% CI is not reported in the RCT and the review authors cannot calculate 95% CI due to lack of raw data

**File 9.9: GRADE evidence profile of systemic enzyme therapy vs. control (e.g., usual care, no intervention, wait-list control) for patients who were treated surgically for lumbar disc herniation from 1 randomized controlled trial**

| **Study** | **Outcome Measure** | **Risk of bias^a^** | **Imprecision^b^** | **Indirectness^c^** | **Inconsistency^d^** | **Publication bias detected?^e^** | **Certainty of evidence^f^** | **Intervention (no. of patients)** | **Comparison (no. of patients)** | **Between-group effect (mean difference (MD) or risk ratio (RR) (95% CI))** |
| --- | --- | --- | --- | --- | --- | --- | --- | --- | --- | --- |
| **Pain (location unspecified) (short term follow up: > 1 week to 3 months)** | | | | | | | | | | |
| Pekar and Steindler (2010)^331^ | VAS, 10cm;  lower is better  10% for between-group difference=1 | Very serious^g^ | Serious | Not serious | Not serious | No | Very low | N=50 | N=50 | MD 1.05 (0.14, 1.97)* |
| **Function (intermediate term follow up: > 3 months to 1 year)** | | | | | | | | | | |
| Pekar and Steindler (2010)^331^ | ODI, 0-100;  lower is better  10% for between-group difference=10 | Very serious^g^ | Serious | Not serious | Not serious | No | Very low | N=50 | N=50 | MD 9.6 (1.39, 17.81)* |
| **Recovery** **(intermediate term follow up: > 3 months to 1 year)** | | | | | | | | | | |
| Pekar and Steindler (2010)^331^ | GPE, 5-point Likert Scale | Very serious^g^ | Serious | Not serious | Not serious | No | Very low | N=50 | N=50 | RR (excellent to better versus same, worse) 1.17 (1.01, 1.37)*  Participants in intervention group are 1.17 times more likely to perceive recovery |
| **Work ability** **(intermediate term follow up: > 3 months to 1 year)** | | | | | | | | | | |
| Pekar and Steindler (2010)^331^ | Work ability, 4-point scale  after surgery | Very serious^g^ | Very serious | Not serious | Not serious | No | Very low | N=50 | N=50 | RR (same/lighter job versus partial/full disability pension) 1.07 (0.76, 1.49)*  Participants in intervention group are 1.07 times more likely to keep work ability. |

**CI:** confidence interval, **GPE:** Global Perceived Effect, **MD:** mean difference, **ODI:** Oswestry Disability Index, **RR:** relative risk, **VAS:** Visual Analogue Scale

^a^Risk of bias: Options are not serious, serious (rate certainty of evidence down one level), and very serious (rate certainty of evidence down two levels). 1. Not serious: study rated as ‘low risk of bias’ or ‘some concerns’ (e.g., unclear co-interventions, no detailed randomization method described but similar baseline characteristics between groups). 2. Serious: study rated as ‘high risk of bias’ with unbalanced baseline characteristics between groups, unclear co-interventions, high/unbalanced drop-out and/or unclear intention-to-treat analysis. 3. Very serious: study rated as ‘high risk of bias’ with unclear randomization sequence generation, inadequate allocation concealment and/or lack of blinding.

^b^Imprecision: Options are not serious, serious (rate certainty of evidence down one level), and very serious (rate certainty of evidence down two levels). Imprecision assessed using between-group effect [point estimate (95% CI)]. 1. Not serious: If the point estimate is not clinically important: the upper and lower boundaries of the CI do not cross a clinically important threshold; the CI may cross the null as long as neither boundary crosses a clinically important threshold. If the point estimate is clinically important: the CI does not cross the null and the boundaries do not cross a clinically important threshold. 2. Serious: If the point estimate is not clinically important: the CI may or may not cross the null but one of the boundaries crosses a clinically important threshold. If the point estimate is clinically important: the CI may cross the null but does not cross a clinically important threshold in the other direction. 3. Very serious: If the point estimate is or is not clinically important: the CI crosses the boundaries of both appreciable harm and benefit (i.e., very wide CI).

^c^Indirectness: Options are not serious, serious (rate certainty of evidence down one level), and very serious (rate certainty of evidence down two levels). Indirectness assessed whether the patients, interventions, or outcomes are different from the research question under investigation.

^d^Inconsistency: Options are not serious, serious (rate certainty of evidence down one level), and very serious (rate certainty of evidence down two levels). Inconsistency assessed effect estimate variance in direction or magnitude. 1. Not serious: effect estimates are consistent in direction and magnitude across studies. 2. Serious: effect estimates vary in magnitude across studies and the heterogeneity could not be explained. 3. Very serious: effect estimates vary in direction across studies and the heterogeneity could not be explained. We rate inconsistency as not serious when there is only one single RCT for an outcome; however, there are no other trials with which to compare findings.

^e^Publication bias assessed using funnel plot if possible, or based on available information from clinical trial registries.

^f^Certainty of evidence: studies began as ‘high’ quality. Studies with ‘yes’ in domain ‘a’ (risk of bias) were downgraded 2 levels. Studies were downgraded one level if ‘yes’ in domains b-e. High quality: we are very confident that the true effect lies close to that of the estimate of the effect. Moderate quality: we are moderately confident in the effect estimate; the true effect is likely to be close to the estimate of the effect, but there is a possibility that it is substantially different. Low quality: our confidence in the effect estimate is limited: the true effect may be substantially different from the estimate of the effect. Very low quality: we have very little confidence in the effect estimate: the true effect is likely to be substantially different from the estimate of effect.

gUnclear randomization; not possible to blind patients, treatment provider and outcome assessor; unclear blinding of data analyst; unclear co-interventions; unbalanced and high (in control) drop-out rates; unclear handling of missing data; unclear intention-to-treat analysis

*Calculated by review authors based on raw data provided in RCT

^+^ 95% CI is not reported in the RCT and the review authors cannot calculate 95% CI due to lack of raw data

**File 9.10: GRADE evidence profile of Traditional Chinese Medicine vs. control (e.g., usual care, no intervention, wait-list control) for patients who were treated surgically for lumbar disc herniation from 1 randomized controlled trial**

| **Study** | **Outcome Measure** | **Risk of bias^a^** | **Imprecision^b^** | **Indirectness^c^** | **Inconsistency^d^** | **Publication bias detected?^e^** | **Certainty of evidence^f^** | **Intervention (no. of patients)** | **Comparison (no. of patients)** | **Between-group effect (mean difference (MD) or risk ratio (RR) (95% CI))** |
| --- | --- | --- | --- | --- | --- | --- | --- | --- | --- | --- |
| **Pain** **(short-term follow up: >1 week to 3 months)** | | | | | | | | | | |
| Fu and Ji (2017)^319^ | VAS, 10cm;  lower is better  10% for between-group difference=1 | Very serious^g^ | Not serious | Not serious | Not serious | No | Low | N=40 | N=40 | MD 1.87 (1.03, 2.71) |
| **Depression** **(short-term follow up: >1 week to 3 months)** | | | | | | | | | | |
| Fu and Ji (2017)^319^ | SDS, 20-80;  lower is better  10% for between-group difference=6 | Very serious^g^ | Not serious | Not serious | Not serious | No | Low | N=40 | N=40 | MD 11.67 (9.73, 13.61) |
| **Anxiety** **(short-term follow up: >1 week to 3 months)** | | | | | | | | | | |
| Fu and Ji (2017)^319^ | SAS, 20-80;  lower is better  10% for between-group difference=6 | Very serious^g^ | Not serious | Not serious | Not serious | No | Low | N=40 | N=40 | MD 11.84 (9.79, 13.89) |

**CI:** confidence interval, **MD:** mean difference, **SAS:** Self-rating Anxiety Scale, **SDS:** Self-rating Depression Scale, **VAS:** Visual Analogue Scale

^a^Risk of bias: Options are not serious, serious (rate certainty of evidence down one level), and very serious (rate certainty of evidence down two levels). 1. Not serious: study rated as ‘low risk of bias’ or ‘some concerns’ (e.g., unclear co-interventions, no detailed randomization method described but similar baseline characteristics between groups). 2. Serious: study rated as ‘high risk of bias’ with unbalanced baseline characteristics between groups, unclear co-interventions, high/unbalanced drop-out and/or unclear intention-to-treat analysis. 3. Very serious: study rated as ‘high risk of bias’ with unclear randomization sequence generation, inadequate allocation concealment and/or lack of blinding.

^b^Imprecision: Options are not serious, serious (rate certainty of evidence down one level), and very serious (rate certainty of evidence down two levels). Imprecision assessed using between-group effect [point estimate (95% CI)]. 1. Not serious: If the point estimate is not clinically important: the upper and lower boundaries of the CI do not cross a clinically important threshold; the CI may cross the null as long as neither boundary crosses a clinically important threshold. If the point estimate is clinically important: the CI does not cross the null and the boundaries do not cross a clinically important threshold. 2. Serious: If the point estimate is not clinically important: the CI may or may not cross the null but one of the boundaries crosses a clinically important threshold. If the point estimate is clinically important: the CI may cross the null but does not cross a clinically important threshold in the other direction. 3. Very serious: If the point estimate is or is not clinically important: the CI crosses the boundaries of both appreciable harm and benefit (i.e., very wide CI).

^c^Indirectness: Options are not serious, serious (rate certainty of evidence down one level), and very serious (rate certainty of evidence down two levels). Indirectness assessed whether the patients, interventions, or outcomes are different from the research question under investigation.

^d^Inconsistency: Options are not serious, serious (rate certainty of evidence down one level), and very serious (rate certainty of evidence down two levels). Inconsistency assessed effect estimate variance in direction or magnitude. 1. Not serious: effect estimates are consistent in direction and magnitude across studies. 2. Serious: effect estimates vary in magnitude across studies and the heterogeneity could not be explained. 3. Very serious: effect estimates vary in direction across studies and the heterogeneity could not be explained. We rate inconsistency as not serious when there is only one single RCT for an outcome; however, there are no other trials with which to compare findings.

^e^Publication bias assessed using funnel plot if possible, or based on available information from clinical trial registries.

^f^Certainty of evidence: studies began as ‘high’ quality. Studies with ‘yes’ in domain ‘a’ (risk of bias) were downgraded 2 levels. Studies were downgraded one level if ‘yes’ in domains b-e.High quality: we are very confident that the true effect lies close to that of the estimate of the effect. Moderate quality: we are moderately confident in the effect estimate; the true effect is likely to be close to the estimate of the effect, but there is a possibility that it is substantially different. Low quality: our confidence in the effect estimate is limited: the true effect may be substantially different from the estimate of the effect. Very low quality: we have very little confidence in the effect estimate: the true effect is likely to be substantially different from the estimate of effect.

^g^Unclear allocation concealment; not possible to blind patients, treatment providers, outcome assessors; unclear blinding of data analyst; similar but limited variables reported at baseline; unclear co-interventions; drop-out not reported; unclear intention-to-treat analysis

*Calculated by review authors based on raw data provided in RCT

^+^ 95% CI is not reported in the RCT and the review authors cannot calculate 95% CI due to lack of raw data

**File 9.11: GRADE evidence profile of CAM vs. control (e.g., usual care, no intervention, wait-list control) for patients who were treated surgically for lumbar disc herniation from 1 randomized controlled trial**

| **Study** | **Outcome Measure** | **Risk of bias^a^** | **Imprecision^b^** | **Indirectness^c^** | **Inconsistency^d^** | **Publication bias detected?^e^** | **Certainty of evidence^f^** | **Intervention (no. of patients)** | **Comparison (no. of patients)** | **Between-group effect (mean difference (MD) or risk ratio (RR) (95% CI))** |
| --- | --- | --- | --- | --- | --- | --- | --- | --- | --- | --- |
| **Pain** **(short-term follow up: >1 week to 3 months)** | | | | | | | | | | |
| Zheng et al. (2015)^337^ | VAS, 100mm;  lower is better  10% for between-group difference=10 | Very serious^g^ | Not serious | Not serious | Not serious | No | Low | N=60 | N=60 | MD 0.25 (0.01, 0.49) |
| **Pain (intermediate term follow up: > 3 months to 1 year)** | | | | | | | | | | |
| Zheng et al. (2015)^337^ | VAS, 100mm;  lower is better  10% for between-group difference=10 | Very serious^g^ | Not serious | Not serious | Not serious | No | Low | N=60 | N=60 | MD -0.12 (-0.42, 0.18) |
| **Function** **(short-term follow up: >1 week to 3 months)** | | | | | | | | | | |
| Zheng et al. (2015)^337^ | JOA, 0-15;  lower is better  10% for between-group difference=1.5 | Very serious^g^ | serious | Not serious | Not serious | No | Very low | N=60 | N=60 | MD 2.01 ( 1.32, 2.70) |
| **Function** **(intermediate term follow up: > 3 months to 1 year)** | | | | | | | | | | |
| Zheng et al. (2015)^337^ | JOA, 0-15;  lower is better  10% for between-group difference=1.5 | Very serious^g^ | Serious | Not serious | Not serious | No | Very low | N=60 | N=60 | MD 2.19 (1.48, 2.90 ) |
| **Function** **(long-term follow up: > 1 year)** | | | | | | | | | | |
| Zheng et al. (2015)^337^ | JOA, 0-15;  lower is better  10% for between-group difference=1.5 | Very serious^g^ | Not serious | Not serious | Not serious | No | Low | N=60 | N=60 | MD 3.15 (2.43, 3.87) |
| **Quality of life** **(short-term follow up: >1 week to 3 months)** | | | | | | | | | | |
| Zheng et al. (2015)^337^ | WHOQOL-BREF, 0-100;  higher is better  10% for between-group difference=10 | Very serious^g^ | Not serious | Not serious | Not serious | No | Low | N=60 | N=60 | MD 2.71 (1.53, 3.89) |
| **Quality of life** **(intermediate term follow up: > 3 months to 1 year)** | | | | | | | | | | |
| Zheng et al. (2015)^337^ | WHOQOL-BREF, 0-100;  higher is better  10% for between-group difference=10 | Very serious^g^ | Not serious | Not serious | Not serious | No | Low | N=60 | N=60 | MD 0.05 (-1.11, 1.21) |
| **Quality of life** **(long-term follow up: > 1 year)** | | | | | | | | | | |
| Zheng et al. (2015)^337^ | WHOQOL-BREF, 0-100;  higher is better  10% for between-group difference=10 | Very serious^g^ | Not serious | Not serious | Not serious | No | Low | N=60 | N=60 | MD -0.53 (-1.70, 0.64) |

**CI:** confidence interval, **GPE:** Global Perceived Effect, **JOA:** Japanese Orthopaedic Association, **MD:** mean difference, **SAS:** Self-rating Anxiety Scale, **SDS:** Self-rating Depression Scale, **VAS:** Visual Analogue Scale, **WHOQOL-BREF:** World Health Organization Quality of Life short version

^a^Risk of bias: Options are not serious, serious (rate certainty of evidence down one level), and very serious (rate certainty of evidence down two levels). 1. Not serious: study rated as ‘low risk of bias’ or ‘some concerns’ (e.g., unclear co-interventions, no detailed randomization method described but similar baseline characteristics between groups). 2. Serious: study rated as ‘high risk of bias’ with unbalanced baseline characteristics between groups, unclear co-interventions, high/unbalanced drop-out and/or unclear intention-to-treat analysis. 3. Very serious: study rated as ‘high risk of bias’ with unclear randomization sequence generation, inadequate allocation concealment and/or lack of blinding.

^b^Imprecision: Options are not serious, serious (rate certainty of evidence down one level), and very serious (rate certainty of evidence down two levels). Imprecision assessed using between-group effect [point estimate (95% CI)]. 1. Not serious: If the point estimate is not clinically important: the upper and lower boundaries of the CI do not cross a clinically important threshold; the CI may cross the null as long as neither boundary crosses a clinically important threshold. If the point estimate is clinically important: the CI does not cross the null and the boundaries do not cross a clinically important threshold. 2. Serious: If the point estimate is not clinically important: the CI may or may not cross the null but one of the boundaries crosses a clinically important threshold. If the point estimate is clinically important: the CI may cross the null but does not cross a clinically important threshold in the other direction. 3. Very serious: If the point estimate is or is not clinically important: the CI crosses the boundaries of both appreciable harm and benefit (i.e., very wide CI).

^c^Indirectness: Options are not serious, serious (rate certainty of evidence down one level), and very serious (rate certainty of evidence down two levels). Indirectness assessed whether the patients, interventions, or outcomes are different from the research question under investigation.

^d^Inconsistency: Options are not serious, serious (rate certainty of evidence down one level), and very serious (rate certainty of evidence down two levels). Inconsistency assessed effect estimate variance in direction or magnitude. 1. Not serious: effect estimates are consistent in direction and magnitude across studies. 2. Serious: effect estimates vary in magnitude across studies and the heterogeneity could not be explained. 3. Very serious: effect estimates vary in direction across studies and the heterogeneity could not be explained. We rate inconsistency as not serious when there is only one single RCT for an outcome; however, there are no other trials with which to compare findings.

^e^Publication bias assessed using funnel plot if possible, or based on available information from clinical trial registries.

^f^Certainty of evidence: studies began as ‘high’ quality. Studies with ‘yes’ in domain ‘a’ (risk of bias) were downgraded 2 levels. Studies were downgraded one level if ‘yes’ in domains b-e.High quality: we are very confident that the true effect lies close to that of the estimate of the effect. Moderate quality: we are moderately confident in the effect estimate; the true effect is likely to be close to the estimate of the effect, but there is a possibility that it is substantially different. Low quality: our confidence in the effect estimate is limited: the true effect may be substantially different from the estimate of the effect. Very low quality: we have very little confidence in the effect estimate: the true effect is likely to be substantially different from the estimate of effect.

^g^Unclear allocation concealment; not possible to blind patients, treatment providers, outcome assessors; unclear blinding of data analyst; similar but limited variables reported baseline; unclear co-interventions; drop-out not reported; unclear handling of missing data; unclear intention-to-treat analysis

*Calculated by review authors based on raw data provided in RCT

^+^ 95% CI is not reported in the RCT and the review authors cannot calculate 95% CI due to lack of raw data

**File 9.12: GRADE evidence profile of pregabalin vs. placebo for patients who were treated surgically for lumbar disc herniation from 1 randomized controlled trial**

| **Study** | **Outcome Measure** | **Risk of bias^a^** | **Imprecision^b^** | **Indirectness^c^** | **Inconsistency^d^** | **Publication bias detected?^e^** | **Certainty of evidence^f^** | **Intervention (no. of patients)** | **Comparison (no. of patients)** | **Between-group effect (mean difference (MD) or risk ratio (RR) (95% CI))** |
| --- | --- | --- | --- | --- | --- | --- | --- | --- | --- | --- |
| **Low back pain** **(short-term follow up: >1 week to 3 months)** | | | | | | | | | | |
| Zarei et al. (2016)^334^  1-day pregabalin | NRS, 0-10;  lower is better  10% for between-group difference=1 | Not serious | Serious | Not serious | Not serious | No | Moderate | N=35 | N=35 | MD -1.77 (-3.05, -0.49) |
| Zarei et al. (2016)^334^  14-day pregabalin | NRS, 0-10;  lower is better  10% for between-group difference=1 | Not serious | Serious | Not serious | Not serious | No | Moderate | N=35 | N=35 | MD -1.49 (-2.79, -0.19) |
| **Low back pain** **(intermediate term follow up: > 3 months to 1 year)** | | | | | | | | | | |
| Zarei et al. (2016)^334^  1-day pregabalin | NRS, 0-10;  lower is better  10% for between-group difference=1 | Not serious | Serious | Not serious | Not serious | No | Moderate | N=35 | N=35 | MD -1.58 (-2.90, -0.26) |
| Zarei et al. (2016)^334^  14-day pregabalin | NRS, 0-10;  lower is better  10% for between-group difference=1 | Not serious | Serious | Not serious | Not serious | No | Moderate | N=35 | N=35 | MD -1.49 (-2.75, -0.23) |
| **Leg pain** **(short-term follow up: >1 week to 3 months)** | | | | | | | | | | |
| Zarei et al. (2016)^334^  1-day pregabalin | NRS, 0-10;  lower is better  10% for between-group difference=1 | Not serious | Not serious | Not serious | Not serious | No | High | N=35 | N=35 | MD 0.17 (-0.65, 0.99) |
| Zarei et al. (2016)^334^  14-day pregabalin | NRS, 0-10;  lower is better  10% for between-group difference=1 | Not serious | Serious | Not serious | Not serious | No | Moderate | N=35 | N=35 | MD -0.5 (-1.32, 0.32) |
| **Leg pain** **(long-term follow up: > 1 year)** | | | | | | | | | | |
| Zarei et al. (2016)^334^  1-day pregabalin | NRS, 0-10;  lower is better  10% for between-group difference=1 | Not serious | Serious | Not serious | Not serious | No | Moderate | N=35 | N=35 | MD 0.26 (-0.65, 1.17) |
| Zarei et al. (2016)^334^  14-day pregabalin | NRS, 0-10;  lower is better  10% for between-group difference=1 | Not serious | Serious | Not serious | Not serious | No | Moderate | N=35 | N=35 | MD -0.41 (-1.34, 0.52) |

**CI:** confidence interval, **MD:** mean difference, **NRS:** Numeric Rating Scale

^a^Risk of bias: Options are not serious, serious (rate certainty of evidence down one level), and very serious (rate certainty of evidence down two levels). 1. Not serious: study rated as ‘low risk of bias’ or ‘some concerns’ (e.g., unclear co-interventions, no detailed randomization method described but similar baseline characteristics between groups). 2. Serious: study rated as ‘high risk of bias’ with unbalanced baseline characteristics between groups, unclear co-interventions, high/unbalanced drop-out and/or unclear intention-to-treat analysis. 3. Very serious: study rated as ‘high risk of bias’ with unclear randomization sequence generation, inadequate allocation concealment and/or lack of blinding.

^b^Imprecision: Options are not serious, serious (rate certainty of evidence down one level), and very serious (rate certainty of evidence down two levels). Imprecision assessed using between-group effect [point estimate (95% CI)]. 1. Not serious: If the point estimate is not clinically important: the upper and lower boundaries of the CI do not cross a clinically important threshold; the CI may cross the null as long as neither boundary crosses a clinically important threshold. If the point estimate is clinically important: the CI does not cross the null and the boundaries do not cross a clinically important threshold. 2. Serious: If the point estimate is not clinically important: the CI may or may not cross the null but one of the boundaries crosses a clinically important threshold. If the point estimate is clinically important: the CI may cross the null but does not cross a clinically important threshold in the other direction. 3. Very serious: If the point estimate is or is not clinically important: the CI crosses the boundaries of both appreciable harm and benefit (i.e., very wide CI).

^c^Indirectness: Options are not serious, serious (rate certainty of evidence down one level), and very serious (rate certainty of evidence down two levels). Indirectness assessed whether the patients, interventions, or outcomes are different from the research question under investigation.

^d^Inconsistency: Options are not serious, serious (rate certainty of evidence down one level), and very serious (rate certainty of evidence down two levels). Inconsistency assessed effect estimate variance in direction or magnitude. 1. Not serious: effect estimates are consistent in direction and magnitude across studies. 2. Serious: effect estimates vary in magnitude across studies and the heterogeneity could not be explained. 3. Very serious: effect estimates vary in direction across studies and the heterogeneity could not be explained. We rate inconsistency as not serious when there is only one single RCT for an outcome; however, there are no other trials with which to compare findings.

^e^Publication bias assessed using funnel plot if possible, or based on available information from clinical trial registries.

^f^Certainty of evidence: studies began as ‘high’ quality. Studies with ‘yes’ in domain ‘a’ (risk of bias) were downgraded 2 levels. Studies were downgraded one level if ‘yes’ in domains b-e.High quality: we are very confident that the true effect lies close to that of the estimate of the effect. Moderate quality: we are moderately confident in the effect estimate; the true effect is likely to be close to the estimate of the effect, but there is a possibility that it is substantially different. Low quality: our confidence in the effect estimate is limited: the true effect may be substantially different from the estimate of the effect. Very low quality: we have very little confidence in the effect estimate: the true effect is likely to be substantially different from the estimate of effect.

*Calculated by review authors based on raw data provided in RCT

^+^ 95% CI is not reported in the RCT and the review authors cannot calculate 95% CI due to lack of raw data

**File 9.13: GRADE evidence profile of 14-day pregabalin vs. 1-day pregabalin for patients who were treated surgically for lumbar disc herniation from 1 randomized controlled trial**

| **Study** | **Outcome Measure** | **Risk of bias^a^** | **Imprecision^b^** | **Indirectness^c^** | **Inconsistency^d^** | **Publication bias detected?^e^** | **Certainty of evidence^f^** | **Intervention (no. of patients)** | **Comparison (no. of patients)** | **Between-group effect (mean difference (MD) or risk ratio (RR) (95% CI))** |
| --- | --- | --- | --- | --- | --- | --- | --- | --- | --- | --- |
| **Low back pain** **(short-term follow up: >1 week to 3 months)** | | | | | | | | | | |
| Zarei et al. (2016)^334^ | NRS, 0-10;  lower is better  10% for between-group difference=1 | Not serious | Serious | Not serious | Not serious | No | Moderate | N=35 | N=35 | MD 0.28 (-0.89, 1.45) |
| **Low back pain** **(long-term follow up: > 1 year)** | | | | | | | | | | |
| Zarei et al. (2016)^334^ | NRS, 0-10;  lower is better  10% for between-group difference=1 | Not serious | Very serious | Not serious | Not serious | No | Low | N=35 | N=35 | MD 0.09 (-1.11, 1.29) |
| **Leg pain** **(short-term follow up: >1 week to 3 months)** | | | | | | | | | | |
| Zarei et al. (2016)^334^ | NRS, 0-10;  lower is better  10% for between-group difference=1 | Not serious | Serious | Not serious | Not serious | No | Moderate | N=35 | N=35 | MD -0.67 (-1.43, 0.09) |
| **Leg pain** **(long-term follow up: > 1 year)** | | | | | | | | | | |
| Zarei et al. (2016)^334^ | NRS, 0-10;  lower is better  10% for between-group difference=1 | Not serious | Serious | Not serious | Not serious | No | Moderate | N=35 | N=35 | MD -0.67 (-1.57, 0.23) |

**CI:** confidence interval, **MD:** mean difference, **NRS:** Numeric Rating Scale

^a^Risk of bias: Options are not serious, serious (rate certainty of evidence down one level), and very serious (rate certainty of evidence down two levels). 1. Not serious: study rated as ‘low risk of bias’ or ‘some concerns’ (e.g., unclear co-interventions, no detailed randomization method described but similar baseline characteristics between groups). 2. Serious: study rated as ‘high risk of bias’ with unbalanced baseline characteristics between groups, unclear co-interventions, high/unbalanced drop-out and/or unclear intention-to-treat analysis. 3. Very serious: study rated as ‘high risk of bias’ with unclear randomization sequence generation, inadequate allocation concealment and/or lack of blinding.

^b^Imprecision: Options are not serious, serious (rate certainty of evidence down one level), and very serious (rate certainty of evidence down two levels). Imprecision assessed using between-group effect [point estimate (95% CI)]. 1. Not serious: If the point estimate is not clinically important: the upper and lower boundaries of the CI do not cross a clinically important threshold; the CI may cross the null as long as neither boundary crosses a clinically important threshold. If the point estimate is clinically important: the CI does not cross the null and the boundaries do not cross a clinically important threshold. 2. Serious: If the point estimate is not clinically important: the CI may or may not cross the null but one of the boundaries crosses a clinically important threshold. If the point estimate is clinically important: the CI may cross the null but does not cross a clinically important threshold in the other direction. 3. Very serious: If the point estimate is or is not clinically important: the CI crosses the boundaries of both appreciable harm and benefit (i.e., very wide CI).

^c^Indirectness: Options are not serious, serious (rate certainty of evidence down one level), and very serious (rate certainty of evidence down two levels). Indirectness assessed whether the patients, interventions, or outcomes are different from the research question under investigation.

^d^Inconsistency: Options are not serious, serious (rate certainty of evidence down one level), and very serious (rate certainty of evidence down two levels). Inconsistency assessed effect estimate variance in direction or magnitude. 1. Not serious: effect estimates are consistent in direction and magnitude across studies. 2. Serious: effect estimates vary in magnitude across studies and the heterogeneity could not be explained. 3. Very serious: effect estimates vary in direction across studies and the heterogeneity could not be explained. We rate inconsistency as not serious when there is only one single RCT for an outcome; however, there are no other trials with which to compare findings.

^e^Publication bias assessed using funnel plot if possible, or based on available information from clinical trial registries.

^f^Certainty of evidence: studies began as ‘high’ quality. Studies with ‘yes’ in domain ‘a’ (risk of bias) were downgraded 2 levels. Studies were downgraded one level if ‘yes’ in domains b-e.High quality: we are very confident that the true effect lies close to that of the estimate of the effect. Moderate quality: we are moderately confident in the effect estimate; the true effect is likely to be close to the estimate of the effect, but there is a possibility that it is substantially different. Low quality: our confidence in the effect estimate is limited: the true effect may be substantially different from the estimate of the effect. Very low quality: we have very little confidence in the effect estimate: the true effect is likely to be substantially different from the estimate of effect.

*Calculated by review authors based on raw data provided in RCT

^+^ 95% CI is not reported in the RCT and the review authors cannot calculate 95% CI due to lack of raw data

**References**

1. Guyatt GH, Oxman AD, Vist GE, Kunz R, Falck-Ytter Y, Alonso-Coello P, et al. GRADE: an emerging consensus on rating quality of evidence and strength of recommendations. Bmj. 2008;336(7650):924-6.

2. Aalto TJL, V.; Herno, A.; Alen, M.; Kroger, H.; Turunen, V.; Savolainen, S.; Saari, T.; Airaksinen, O. Postoperative rehabilitation does not improve functional outcome in lumbar spinal stenosis: a prospective study with 2-year postoperative follow-up. Eur Spine J. 2011;20(8):1331-40.

3. Abbott ADT-L, R.; Hedlund, R. Early rehabilitation targeting cognition, behavior, and motor function after lumbar fusion: a randomized controlled trial. Spine. 2010;35(8):848-57.

4. Abbott Ad T-LRHR. Early rehabilitation targeting cognition, behavior, and motor function after lumbar fusion: a randomized controlled trial 2010 [updated 2010//. 8:[848]. Available from: <http://ovidsp.ovid.com/ovidweb.cgi?T=JS&PAGE=reference&D=cctr&NEWS=N&AN=CN-00751001>.

5. Abbott ADT-L, R.; Hedlund, R. Leg pain and psychological variables predict outcome 2-3 years after lumbar fusion surgery. Eur Spine J. 2011;20(10):1626-34.

6. Abbott A T-LRHR. The effectiveness of physiotherapeutic rehabilitation and issues of outcome prediction after lumbar fusion surgery: Elsevier Ltd; 2011 [updated 2011//. eS20]. Available from: <http://ovidsp.ovid.com/ovidweb.cgi?T=JS&PAGE=reference&D=cctr&NEWS=N&AN=CN-01076346>.

7. Abbott Ad T-LRHR. Leg pain and psychological variables predict outcome 2-3 years after lumbar fusion surgery: Springer Verlag; 2011 [updated 2011//. 10:[1626]. Available from: <http://ovidsp.ovid.com/ovidweb.cgi?T=JS&PAGE=reference&D=cctr&NEWS=N&AN=CN-00898341>.

8. Abbott ADH, Rune; Tyni-lennÉÉ, Raija. Patients' experience post-lumbar fusion regarding back problems, recovery and expectations in terms of the international classification of functioning, disability and health. Disabil Rehabil. 2011;33(15/16):1399-408.

9. Alaranta HH, M.; Einola, S.; Kallio, V.; Knuts, L. R.; Torma, T. Rehabilitation after surgery for lumbar disc herniation: results of a randomized clinical trial. Int J Rehabil Res. 1986;9(3):247-57.

10. Alaranta HH, M.; Einola, S. Rehabilitation after surgery for lumbar disc herniation: Results of a randomized clinical trial. Int J Rehabil Res. 1986;9(3):247-57.

11. Alaranta H HMESKVKLRTT. Rehabilitation after surgery for lumbar disc herniation: results of a randomized clinical trial 1986 [updated 1986//. 3:[247]. Available from: <http://ovidsp.ovid.com/ovidweb.cgi?T=JS&PAGE=reference&D=cctr&NEWS=N&AN=CN-00045498>.

12. Aldemir K GA. The effect of pedometer-supported walking and telemonitoring after disc hernia surgery on pain and disability levels and quality of life. International journal of nursing practice. 2021;27(2):e12917.

13. Aldrete JA. Epidural injections of indomethacin for postlaminectomy syndrome: a preliminary report 2003 [updated 2003//. 2:[463]. Available from: <http://ovidsp.ovid.com/ovidweb.cgi?T=JS&PAGE=reference&D=cctr&NEWS=N&AN=CN-00412821>.

14. Alimohammadi NE, M.; Yousefi, H.; Tabesh, H. The effect of continuing care on patient's quality-of-life after disc surgery in neurosurgery and very important person wards. Journal of Education & Health Promotion. 2015;4:106.

15. Andersen TC, F. B.; Egund, N.; Ernst, C.; Fruensgaard, S.; Ostergaard, J.; Andersen, J. L.; Rasmussen, S.; Niedermann, B.; Hoy, K.; Helmig, P.; Holm, R.; Lindblad, B. E.; Hansen, E. S.; Bunger, C. The effect of electrical stimulation on lumbar spinal fusion in older patients: a randomized, controlled, multi-center trial: part 2: fusion rates. Spine. 2009;34(21):2248-53.

16. Andersen TC, F. B.; Ernst, C.; Fruensgaard, S.; Ostergaard, J.; Andersen, J. L.; Rasmussen, S.; Niedermann, B.; Hoy, K.; Helmig, P.; Holm, R.; Lindblad, B. E.; Hansen, E. S.; Egund, N.; Bunger, C. The effect of electrical stimulation on lumbar spinal fusion in older patients: a randomized, controlled, multi-center trial: part 1: functional outcome. Spine. 2009;34(21):2241-7.

17. Andersen T CFBENECFSOJAJLRSNBHKHPH. The effect of electrical stimulation on lumbar spinal fusion in older patients: a randomized, controlled, multi-center trial: part 2: fusion rates 2009 [updated 2009//. 21:[2248]. Available from: <http://ovidsp.ovid.com/ovidweb.cgi?T=JS&PAGE=reference&D=cctr&NEWS=N&AN=CN-00728648>.

18. Andersen T CFBECFSOJAJLRSNBHKHPHRL. The effect of electrical stimulation on lumbar spinal fusion in older patients: a randomized, controlled, multi-center trial: part 1: functional outcome 2009 [updated 2009//. 21:[2241]. Available from: <http://ovidsp.ovid.com/ovidweb.cgi?T=JS&PAGE=reference&D=cctr&NEWS=N&AN=CN-00731945>.

19. Andersen TC, F. B.; Langdahl, B. L.; Ernst, C.; Fruensgaard, S.; Ostergaard, J.; Andersen, J. L.; Rasmussen, S.; Niedermann, B.; Hoy, K.; Helmig, P.; Holm, R.; Lindblad, B. E.; Hansen, E. S.; Egund, N.; Bunger, C. Fusion mass bone quality after uninstrumented spinal fusion in older patients. Eur Spine J. 2010;19(12):2200-8.

20. Anderson JTH, A. R.; Percy, R.; Woods, S. T.; Ahn, U. M.; Ahn, N. U. Chronic Opioid Therapy After Lumbar Fusion Surgery for Degenerative Disc Disease in a Workers' Compensation Setting. Spine. 2015;40(22):1775-84.

21. Anderson JTT, E. Y.; Haas, A. R.; Percy, R.; Woods, S. T.; Ahn, U. M.; Ahn, N. U. Multilevel Lumbar Fusion Is a Risk Factor for Lower Return to Work Rates Among Workers' Compensation Subjects With Degenerative Disc Disease. J Surg Orthop Adv. 2018;27(3):209-18.

22. Angelini E, Baranto A, Brisby H, Wijk H. Healthcare practitioners' experiences of postoperative pain management in lumbar spine surgery care-A qualitative study. Journal of clinical nursing. 2020;29(9-10):1662-72.

23. Archer K VSKTPSAOSCJSMMJDCJWS. Improving surgical spine outcomes through a targeted postoperative rehabilitation approach: Elsevier Inc.; 2014 [updated 2014//. 11 SUPPL. 1:[S76]. Available from: <http://ovidsp.ovid.com/ovidweb.cgi?T=JS&PAGE=reference&D=cctr&NEWS=N&AN=CN-01023310>.

24. Archer KRD, C. J.; Vanston, S. W.; Koyama, T.; Phillips, S. E.; Mathis, S. L.; George, S. Z.; McGirt, M. J.; Spengler, D. M.; Aaronson, O. S.; Cheng, J. S.; Wegener, S. T. Cognitive-Behavioral-Based Physical Therapy for Patients With Chronic Pain Undergoing Lumbar Spine Surgery: A Randomized Controlled Trial. J Pain. 2016;17(1):76-89.

25. Archer Kr DCJVSWKTPSEMSLGSZMMJSD. Cognitive-Behavioral-Based Physical Therapy for Patients With Chronic Pain Undergoing Lumbar Spine Surgery: a Randomized Controlled Trial: Churchill Livingstone Inc.; 2016 [updated 2016//. 1:[76]. Available from: <http://ovidsp.ovid.com/ovidweb.cgi?T=JS&PAGE=reference&D=cctr&NEWS=N&AN=CN-01128378>.

26. Archer KRDCJVSWKTPSEGSZMMJSDMA. Cognitive-Behavioral-Based Physical Therapy for Patients with Chronic Pain Undergoing Lumbar Spine Surgery: a Randomized Controlled Trial 2016 [updated 2016//. 76]. Available from: <http://ovidsp.ovid.com/ovidweb.cgi?T=JS&PAGE=reference&D=cctr&NEWS=N&AN=CN-01380267>.

27. Archer KRD, Clinton J.; Vanston, Susan W.; Koyama, Tatsuki; Phillips, Sharon E.; Mathis, Shannon L.; George, Steven Z.; McGirt, Matthew J.; Spengler, Dan M.; Aaronson, Oran S.; Cheng, Joseph S.; Wegener, Stephen T. "Cognitive-behavioral-based physical therapy for patients with chronic pain undergoing lumbar spine surgery: A randomized controlled trial": Erratum. The Journal of Pain. 2017;18(4):477.

28. Aspegren DDB, A. L. A study of postspinal surgery cases in chiropractic offices. J Manipulative Physiol Ther. 1994;17(2):88-92.

29. Atarod MM, E.; Ghandehari, H.; Mehrdad, R.; Izadi, N. Predictive factors for return to work after lumbar discectomy. Int J Occup Saf Ergon. 2019:1-6.

30. Bentsen SBR, T.; Wahl, A. K.; Miaskowski, C. The pain experience and future expectations of chronic low back pain patients following spinal fusion. J Clin Nurs. 2008;17(7B):153-9.

31. Benyamin RMS, P. S.; Mi, D. A. S. Encore I. MILD Is an Effective Treatment for Lumbar Spinal Stenosis with Neurogenic Claudication: MiDAS ENCORE Randomized Controlled Trial. Pain physician. 2016;19(4):229-42.

32. Bible JEB, D.; Whang, P. G.; Simpson, A. K.; Rechtine, G. R.; Grauer, J. N. Postoperative bracing after spine surgery for degenerative conditions: a questionnaire study. Spine J. 2009;9(4):309-16.

33. Bolesta MJ. Meaningful improvement in perioperative pain control after lumbar surgery. Journal of Bone and Joint Surgery - Series A. 2013;95(5):e301-e2.

34. Bono Cm LDACTDSJHWKBHMBSAJ. The effect of short (2-weeks) versus long (6-weeks) post-operative restrictions following lumbar discectomy: a prospective randomized control trial 2017 [updated 2017//. 3:[905]. Available from: <http://ovidsp.ovid.com/ovidweb.cgi?T=JS&PAGE=reference&D=cctr&NEWS=N&AN=CN-01444130>.

35. Bouche KD, A. S.; Caemaert, J.; Vanderstraeten, G.; Danneels, L. Long term outcome of Lumbar Discectomy: Results from a biopsychosocial perspective. Acta Neurologica Belgica. 2011;111(4):287-95.

36. Bourghli AB, L.; Larrieu, D.; Kaissar, F.; Cawley, D.; Fujishiro, T.; Kieser, D.; Gille, O.; Vital, J. M.; Alanay, A.; Pellise, F.; Acaroglu, E.; Perez-Grueso, F. J.; Kleinstuck, F.; Obeid, I. Opioids and analgesics use after adult spinal deformity surgery correlates with sagittal alignment and preoperative analgesic pattern. European Spine Journal. 2019.

37. Brox JIS, R.; Friis, A.; Nygaard, O.; Indahl, A.; Keller, A.; Ingebrigtsen, T.; Eriksen, H. R.; Holm, I.; Koller, A. K.; Riise, R.; Reikeras, O. Randomized clinical trial of lumbar instrumented fusion and cognitive intervention and exercises in patients with chronic low back pain and disc degeneration. Spine. 2003;28(17):1913-21.

38. Burke Sa H-CCKAPS. Return to work/work retention outcomes of a functional restoration program. A multi-center, prospective study with a comparison group 1994 [updated 1994//. 17:[1880]. Available from: <http://ovidsp.ovid.com/ovidweb.cgi?T=JS&PAGE=reference&D=cctr&NEWS=N&AN=CN-00108119>.

39. Byungho JKJ, Ahn; Heecheol, Cho; Dongyun, Kim; Taeyeong, Kim; Bumchul, Yoon. Early individualised manipulative rehabilitation following lumbar open laser microdiscectomy improves early post-operative functional disability: A randomized, controlled pilot study. J Back Musculoskeletal Rehabil. 2016;29(1):23-9.

40. Caby IV, J.; Letombe, A.; Pelayo, P. Effects of a five-week intensive and multidisciplinary spine-specific functional restoration program in chronic low back pain patients with or without surgery. [French]; Effets d'un programme intensif et multidisciplinaire de restauration fonctionnelle du rachis de cinq semaines chez des sujets lombalgiques chroniques operes et non operes. Annals of Physical and Rehabilitation Medicine. 2010;53(10):621-31.

41. Cai JW, G.; Zhao, H. Effects of rehabilitation nursing on minimally invasive surgery and the application of long-term activity function in patients with intervertebral disc herniation. Int J Clin Exp Med. 2019;12(6):7418-25.

42. Chen CYC, C. W.; Lee, S. T.; Chen, Y. C.; Tang, S. F.; Cheng, C. H.; Lin, Y. H. Is rehabilitation intervention during hospitalization enough for functional improvements in patients undergoing lumbar decompression surgery? A prospective randomized controlled study. Clin Neurol Neurosurg. 2015;129 Suppl 1:S41-6.

43. Chen CYC, C. W.; Lee, S. T.; Chen, Y. C.; Tang, S. F. T.; Cheng, C. H.; Lin, Y. H. Is rehabilitation intervention during hospitalization enough for functional improvements in patients undergoing lumbar decompression surgery? A prospective randomized controlled study. Clinical Neurology and Neurosurgery. 2015;129(S1):S41-S6.

44. Chen Cy CCWLSTCYCTSFCCHLYH. Is rehabilitation intervention during hospitalization enough for functional improvements in patients undergoing lumbar decompression surgery? A prospective randomized controlled study 2015 [updated 2015//. S41]. Available from: <http://ovidsp.ovid.com/ovidweb.cgi?T=JS&PAGE=reference&D=cctr&NEWS=N&AN=CN-01110546>.

45. Chen C-Y CCWLSTCYCTSFTCCHLYH. Is rehabilitation intervention during hospitalization enough for functional improvements in patients undergoing lumbar decompression surgery? A prospective randomized controlled study: Elsevier; 2015 [updated 2015//. S1:[S41]. Available from: <http://ovidsp.ovid.com/ovidweb.cgi?T=JS&PAGE=reference&D=cctr&NEWS=N&AN=CN-01052767>.

46. Cherkin DCD, R. A.; Wheeler, K.; Ciol, M. A. Physician views about treating low back pain. The results of a national survey. Spine. 1995;20(1):1-9; discussion -10.

47. Choi G RPPKMJCDJCYSLSH. The effect of early isolated lumbar extension exercise program for patients with herniated disc undergoing lumbar discectomy 2005 [updated 2005//. 4:[764]. Available from: <http://ovidsp.ovid.com/ovidweb.cgi?T=JS&PAGE=reference&D=cctr&NEWS=N&AN=CN-00561347>.

48. Choi YSS, Jae-Kwang; Song, Jong Wook; Kim, Jong Chan; Yoo, Young Chul; Kwak, Young Lan. Combination of pregabalin and dexamethasone for postoperative pain and functional outcome in patients undergoing lumbar spinal surgery: A randomized placebo-controlled trial. The Clinical Journal of Pain. 2013;29(1):9-14.

49. Choi Ys SJKSJWKJCYYCKYL. Combination of pregabalin and dexamethasone for postoperative pain and functional outcome in patients undergoing lumbar spinal surgery: a randomized placebo-controlled trial 2013 [updated 2013//. 1:[9]. Available from: <http://ovidsp.ovid.com/ovidweb.cgi?T=JS&PAGE=reference&D=cctr&NEWS=N&AN=CN-00869851>.

50. Christensen FBL, I.; Bunger, C. E. Importance of the back-cafe concept to rehabilitation after lumbar spinal fusion: a randomized clinical study with a 2-year follow-up. Spine. 2003;28(23):2561-9.

51. Christensen Fb LIBCE. Importance of the back-cafe concept to rehabilitation after lumbar spinal fusion: a randomized clinical study with a 2-year follow-up 2003 [updated 2003//. 23:[2561]. Available from: <http://ovidsp.ovid.com/ovidweb.cgi?T=JS&PAGE=reference&D=cctr&NEWS=N&AN=CN-00488870>.

52. Cienciala JC, R.; Repko, M.; Krbec, M. [Dynamic neutralization using the Dynesys system for treatment of degenerative disc disease of the lumbar spine]. Acta Chir Orthop Traumatol Cech. 2010;77(3):203-8.

53. Cienciala JC, R.; Repko, M.; Krbec, M. Dynamic neutralization using the dynesys system for treatment of degenerative disc disease of the lumbar spine. [Czech]; Osetreni degenerativniho onemocneni bederni patere metodou dynamicke neutralizace systemem dynesys. Acta Chir Orthop Traumatol Cech. 2010;77(3):203-8.

54. Claus DC, E.; Chazal, J.; Irthum, B.; Mulliez, A.; Givron, P. An evidence-based information booklet helps reduce fear-avoidance beliefs after first-time discectomy for disc prolapse. Ann Phys Rehabil Med. 2017;60(2):68-73.

55. Claus D CECJIBMAGP. An evidence-based information booklet helps reduce fear-avoidance beliefs after first-time discectomy for disc prolapse: Elsevier Masson SAS (62 rue Camille Desmoulins, Issy les Moulineaux Cedex 92442, France); 2017 [updated 2017//. 2:[68]. Available from: <http://ovidsp.ovid.com/ovidweb.cgi?T=JS&PAGE=reference&D=cctr&NEWS=N&AN=CN-01373711>.

56. Corbin TP. Point of view: Clinical outcomes after posterolateral lumbar fusion in workers compensation patients: A case-control study. Spine. 2010;35(19):1820.

57. Coronado RA, Ehde DM, Pennings JS, Vanston SW, Koyama T, Phillips SE, et al. Psychosocial Mechanisms of Cognitive-Behavioral-Based Physical Therapy Outcomes After Spine Surgery: Preliminary Findings From Mediation Analyses. Physical therapy. 2020;100(10):1793-804.

58. Coronado Ra EDMPJSVSWKTPSEMSLMMJSD. Psychosocial Mechanisms of Cognitive-Behavioral-Based Physical Therapy Outcomes After Spine Surgery: preliminary Findings From Mediation Analyses. Physical therapy. 2020;100(10):1793.

59. Cox CLB, G. M. Integrating complementary health care in outpatient surgery for discectomy: the patient's perspective. Journal of Orthopaedic Nursing. 2000;4(4):179-84.

60. Dailey ATG, Z.; Choudhri, T. F.; Watters, Iii W. C.; Resnick, D. K.; Sharan, A.; Eck, J. C.; Mummaneni, P. V.; Wang, J. C.; Groff, M. W.; Dhall, S. S.; Kaiser, M. G. Guideline update for the performance of fusion procedures for degenerative disease of the lumbar spine. Part 14: Brace therapy as an adjunct to or substitute for lumbar fusion. Journal of Neurosurgery: Spine. 2014;21(1):91-101.

61. Danielsen JMJ, R.; Kibsgaard, S. K.; Hellevik, E. Early aggressive exercise for postoperative rehabilitation after discectomy. Spine. 2000;25(8):1015-20.

62. Danielsen Jm JRKSKHE. Early aggressive exercise for postoperative rehabilitation after discectomy 2000 [updated 2000//. 8:[1015]. Available from: <http://ovidsp.ovid.com/ovidweb.cgi?T=JS&PAGE=reference&D=cctr&NEWS=N&AN=CN-00277342>.

63. Debono B, Corniola MV, Pietton R, Sabatier P, Hamel O, Tessitore E. Benefits of Enhanced Recovery After Surgery for fusion in degenerative spine surgery: impact on outcome, length of stay, and patient satisfaction. Neurosurgical focus. 2019;46(4):E6.

64. Donaldson BLS, E. A.; Inglis, G.; Rivett, D.; Frampton, C. Comparison of usual surgical advice versus a nonaggravating six-month gym-based exercise rehabilitation program post-lumbar discectomy: results at one-year follow-up. Spine J. 2006;6(4):357-63.

65. Donaldson Bl SEAIGRDFC. Comparison of usual surgical advice versus a nonaggravating six-month gym-based exercise rehabilitation program post-lumbar discectomy: results at one-year follow-up 2006 [updated 2006//. 4:[357]. Available from: <http://ovidsp.ovid.com/ovidweb.cgi?T=JS&PAGE=reference&D=cctr&NEWS=N&AN=CN-00566362>.

66. Donceel P DBMLD. Return to work after surgery for lumbar disc herniation. A rehabilitation-oriented approach in insurance medicine 1999 [updated 1999//. 9:[872]. Available from: <http://ovidsp.ovid.com/ovidweb.cgi?T=JS&PAGE=reference&D=cctr&NEWS=N&AN=CN-00163176>.

67. Donceel PDB, M.; Lahaye, D. Return to work after surgery for lumbar disc herniation. A rehabilitation-oriented approach in insurance medicine. Spine. 1999;24(9):872-6.

68. Duculan R, Rigaud M, Cammisa FP, Sama AA, Hughes AP, Mancuso CA, et al. 195. Fostering physical activity after complex lumbar spine surgery: long-term results of a randomized trial. Spine Journal. 2020;20(9 Supplement):S96-S7.

69. Ebata S TJHTMKIYOTSYOTYZMYHH. Role of Weekly Teriparatide Administration in Osseous Union Enhancement within Six Months After Posterior or Transforaminal Lumbar Interbody Fusion for Osteoporosis-Associated Lumbar Degenerative Disorders: a Multicenter, Prospective Randomized Study 2017 [updated 2017//. 5:[365]. Available from: <http://ovidsp.ovid.com/ovidweb.cgi?T=JS&PAGE=reference&D=cctr&NEWS=N&AN=CN-01338896>.

70. Ebenbichler Gr ISPVSRWGNKCKRKL. Twelve-year follow-up of a randomized controlled trial of comprehensive physiotherapy following disc herniation operation 2015 [updated 2015//. 6:[548]. Available from: <http://ovidsp.ovid.com/ovidweb.cgi?T=JS&PAGE=reference&D=cctr&NEWS=N&AN=CN-01073307>.

71. Erdogan Z, Bulut H. Effectiveness of Computer Assisted Training of Patients Undergoing Lumbar Disc Herniation Surgery. Turkish neurosurgery. 2020;30(1):69-77.

72. Erdogmus Cb RKLSRMHNMSAPMOWUKEGR. Physiotherapy-based rehabilitation following disc herniation operation: results of a randomized clinical trial 2007 [updated 2007//. 19:[2041]. Available from: <http://ovidsp.ovid.com/ovidweb.cgi?T=JS&PAGE=reference&D=cctr&NEWS=N&AN=CN-00611805>.

73. Feng HZ, Y. F.; Ding, M. [Analysis of therapeuttic effect of lower limb sensation disorder after lumbar disc herniation operation treated with plum-blossom needle along meridians]. Zhongguo zhenjiu. 2012;32(2):129-32.

74. Feng H ZYFDM. Analysis of therapeuttic effect of lower limb sensation disorder after lumbar disc herniation operation treated with plum-blossom needle along meridians 2012 [updated 2012//. 2:[129]. Available from: <http://ovidsp.ovid.com/ovidweb.cgi?T=JS&PAGE=reference&D=cctr&NEWS=N&AN=CN-00814167>.

75. Flanigan DCE, J. S.; Glassman, A. H. Psychological Factors Affecting Rehabilitation and Outcomes Following Elective Orthopaedic Surgery. J Am Acad Orthop Surg. 2015;23(9):563-70.

76. Gallon RL. Perception of disability in chronic back pain patients: a long-term follow-up. Pain. 1989;37(1):67-75.

77. Gerling Mc LDPPGLVBKLAMTSLJDTTDZWS. Risk Factors for Reoperation in Patients Treated Surgically for Degenerative Spondylolisthesis: a Subanalysis of the 8-year Data From the SPORT Trial: Lippincott Williams and Wilkins (E-mail: kathiest.clai@apta.org); 2017 [updated 2017//. 20:[1559]. Available from: <http://ovidsp.ovid.com/ovidweb.cgi?T=JS&PAGE=reference&D=cctr&NEWS=N&AN=CN-01446027>.

78. Gilmore SJH, A. J.; Davidson, M.; McClelland, J. A. Predictors of substantial improvement in physical function six months after lumbar surgery: is early post-operative walking important? A prospective cohort study. BMC Musculoskelet Disord. 2019;20(1):418.

79. Green AG, P.; Scott-Young, M.; Abbott, A. Physiotherapeutic Rehabilitation Following Lumbar Total Disc Replacement: A Retrospective Study. Physiother Res Int. 2016;21(3):155-63.

80. Green AG, Philippa; Scott ‐ Young, Matthew; Abbott, Allan. Physiotherapeutic Rehabilitation Following Lumbar Total Disc Replacement: A Retrospective Study. Physiother Res Int. 2016;21(3):155-63.

81. Grieff ANG, G. M.; Jallo, J. Use of liposomal bupivacaine in the postoperative management of posterior spinal decompression. J Neurosurg Spine. 2016;25(1):88-93.

82. Guo XH, X.; Ding, S.; Chang, S. Rehabilitation nursing for patient rehabilitation after minimally invasive spine surgery. Int J Clin Exp Med. 2019;12(3):2450-5.

83. Hakkinen A YJKHTUKI. Effects of home strength training and stretching versus stretching alone after lumbar disk surgery: a randomized study with a 1-year follow-up 2005 [updated 2005//. 5:[865]. Available from: <http://ovidsp.ovid.com/ovidweb.cgi?T=JS&PAGE=reference&D=cctr&NEWS=N&AN=CN-00514449>.

84. Harper WLS, W. K.; Kubat, N. J.; Isenberg, R. A. An open-label pilot study of pulsed electromagnetic feld therapy in the treatment of failed back surgery syndrome pain. Int Med Case Rep J. 2014;8:13-22.

85. He JML, A. J.; Peng, Y. [Case-control study on comprehensive rehabilitation for postoperative pain after spinal surgery]. Zhongguo Gu Shang. 2010;23(12):929-31.

86. Hedlund R. The long-term outcome of lumbar fusion in the Swedish lumbar spine study. Journal fur Mineralstoffwechsel. 2015;22(4):135.

87. Heo I, Shin B-C, Cho J-H, Ha I-H, Hwang E-H, Lee J-H, et al. Multicentre randomised controlled clinical trial of electroacupuncture with usual care for patients with non-acute pain after back surgery. British journal of anaesthesia. 2021;126(3):692-9.

88. Herkowitz HN. Degenerative lumbar spondylolisthesis: A surgeon's perspective of 30 years in practice. Spine Journal. 2010;10(10):916-7.

89. Hoffman HB, S. S.; Li, C. H.; Haakana, P.; Lu, D. C. Minimally Invasive Decompression and Physiotherapy for Lumbar Spinal Stenosis in Geriatric Patients. Cureus. 2018;10(6):e2785.

90. Hou JY, R.; Yang, Y.; Tang, Y.; Deng, H.; Chen, Z.; Wu, Y.; Shen, H. The Effectiveness and Safety of Utilizing Mobile Phone-Based Programs for Rehabilitation After Lumbar Spinal Surgery: Multicenter, Prospective Randomized Controlled Trial. JMIR Mhealth Uhealth. 2019;7(2):e10201.

91. Hou J, Yang R, Yang Y, Tang Y, Deng H, Chen Z, et al. The Effectiveness and Safety of Utilizing Mobile Phone-Based Programs for Rehabilitation After Lumbar Spinal Surgery: Multicenter, Prospective Randomized Controlled Trial. JMIR mHealth and uHealth. 2019;7(2):e10201.

92. Hou J YRYYTYDHCZWYSH. The Effectiveness and Safety of Utilizing Mobile Phone-Based Programs for Rehabilitation After Lumbar Spinal Surgery: multicenter, Prospective Randomized Controlled Trial 2019 [updated 2019//. 2:[e10201]. Available from: <http://ovidsp.ovid.com/ovidweb.cgi?T=JS&PAGE=reference&D=cctr&NEWS=N&AN=CN-01707025>.

93. Ilves OH, A.; Dekker, J.; Wahlman, M.; Tarnanen, S.; Pekkanen, L.; Ylinen, J.; Kautiainen, H.; Neva, M. Effectiveness of postoperative home-exercise compared with usual care on kinesiophobia and physical activity in spondylolisthesis: A randomized controlled trial. J Rehabil Med. 2017;49(9):751-7.

94. Ilves O HADJWMTSPLYJKHNM. Effectiveness of postoperative home-exercise compared with usual care on kinesiophobia and physical activity in spondylolisthesis: a randomized controlled trial 2017 [updated 2017//. 9:[751]. Available from: <http://ovidsp.ovid.com/ovidweb.cgi?T=JS&PAGE=reference&D=cctr&NEWS=N&AN=CN-01572323>.

95. Ilves O NMHHKDJJSKKHA. Postoperative back-specific and aerobic training does not improve trunk muscle strength and imbalance after lumbar spine fusion: a randomized controlled trial: Springer Verlag; 2017 [updated 2017//. 2:[S254]. Available from: <http://ovidsp.ovid.com/ovidweb.cgi?T=JS&PAGE=reference&D=cctr&NEWS=N&AN=CN-01421800>.

96. Ishida K UYYHSS. Early active rehabilitation after surgery for lumbar disc herniation: a prospective randomized control trial: Lippincott Williams and Wilkins; 2010 [updated 2010//. Available from: <http://ovidsp.ovid.com/ovidweb.cgi?T=JS&PAGE=reference&D=cctr&NEWS=N&AN=CN-00792716>.

97. Ishida KUYAYHASS. Early active rehabilitation after surgery for lumbar disc herniation: a prospective, randomized control trial 2010 [updated 2010//. Suppl]. Available from: <http://ovidsp.ovid.com/ovidweb.cgi?T=JS&PAGE=reference&D=cctr&NEWS=N&AN=CN-00991421>.

98. Johnson RG. Electrical stimulation and lumbar spinal fusion. Orthopedics. 2001;24(2):87, 106.

99. Kaliya-Perumal AKL, M. L.; Luo, C. A.; Tsai, T. T.; Lai, P. L.; Chen, L. H.; Chen, W. J.; Niu, C. C. Retrospective radiological outcome analysis following teriparatide use in elderly patients undergoing multilevel instrumented lumbar fusion surgery. Medicine (Baltimore). 2017;96(5):e5996.

100. Kaptain KB, V.; Dreyer, P. Patient participation in postoperative pain assessment after spine surgery in a recovery unit. J Clin Nurs. 2017;26(19-20):2986-94.

101. Kara BY, Y.; Erbayraktar, S. Physiotherapy results after nucleoplasty. Neurosciences. 2009;14(4):389-90.

102. Katz JN. Surgery for lumbar spinal stenosis: informed patient preferences should weigh heavily. Ann Intern Med. 2015;162(7):518-9.

103. Khurana G JPSJPBKK. Postoperative pain and long-term functional outcome after administration of gabapentin and pregabalin in patients undergoing spinal surgery: Lippincott Williams and Wilkins; 2014 [updated 2014//. 6:[E363]. Available from: <http://ovidsp.ovid.com/ovidweb.cgi?T=JS&PAGE=reference&D=cctr&NEWS=N&AN=CN-01119345>.

104. Kim BJA, J.; Cho, H.; Kim, D.; Kim, T.; Yoon, B. Rehabilitation with osteopathic manipulative treatment after lumbar disc surgery: A randomised, controlled pilot study. International Journal of Osteopathic Medicine. 2015;18(3):181-8.

105. Kim Hj AHSNYCBSLCKYJS. Comparative study of the efficacy of transdermal buprenorphine patches and prolonged-release tramadol tablets for postoperative pain control after spinal fusion surgery: a prospective, randomized controlled non-inferiority trial 2017 [updated 2017//. 11:[2961]. Available from: <http://ovidsp.ovid.com/ovidweb.cgi?T=JS&PAGE=reference&D=cctr&NEWS=N&AN=CN-01600071>.

106. Kim S-I HKYAHSRKWLHWOIS. Sex-specific and Age-specific Analgesia for Early Postoperative Pain Management after Lumbar Decompressive Surgery: a Randomized Clinical Trial: Lippincott Williams and Wilkins; 2018 [updated 2018//. Available from: <http://ovidsp.ovid.com/ovidweb.cgi?T=JS&PAGE=reference&D=cctr&NEWS=N&AN=CN-01767076>.

107. Kim SIH, K. Y.; An, H. S.; Rhyu, K. W.; Lee, H. W.; Oh, I. S. Sex-specific and Age-specific Analgesia for Early Postoperative Pain Management After Lumbar Decompressive Surgery: A Randomized Clinical Trial. Clin Spine Surg. 2019;32(7):E311-E8.

108. Kitze KR, V.; Angermeyer, M. C. [Pain related impairment and the ability to function in herniated disc patients during rehabilitation]. Rehabilitation (Stuttg). 2007;46(6):333-9.

109. Kjellby-Wendt G SJCS. Early active rehabilitation after surgical treatment of lumbar disc herniation. A prospective, randomized study of psychometric assessment and a 5 year evaluation 2001 [updated 2001//. 7:[S58]. Available from: <http://ovidsp.ovid.com/ovidweb.cgi?T=JS&PAGE=reference&D=cctr&NEWS=N&AN=CN-00431140>.

110. Knape H. Bezitramide, an orally active analgesic. An investigation on pain following operations for lumbar disc protrusion (preliminary report) 1970 [updated 1970//. 4:[325]. Available from: <http://ovidsp.ovid.com/ovidweb.cgi?T=JS&PAGE=reference&D=cctr&NEWS=N&AN=CN-00004615>.

111. Knape H. Further experiences with bezitramide. Its analgesic action and side effects in patients operated upon for lumbar disc protrusion 1971 [updated 1971//. 1:[76]. Available from: <http://ovidsp.ovid.com/ovidweb.cgi?T=JS&PAGE=reference&D=cctr&NEWS=N&AN=CN-00005634>.

112. Kryuchkova SV. Efficacy of baclosan in combined therapy of muscular-tonic pain syndrome in the early postoperative period in patients after microdiscectomy 2015 [updated 2015//. 3:[41]. Available from: <http://ovidsp.ovid.com/ovidweb.cgi?T=JS&PAGE=reference&D=cctr&NEWS=N&AN=CN-01108993>.

113. Kulikov Ag VDDMAEZTNUKI. New approach for evaluating the effectiveness of whole-body magnetic field therapy in the rehabilitation of patients with lumbar discectomy: IOS Press (Nieuwe Hemweg 6B, Amsterdam 1013 BG, Netherlands); 2018 [updated 2018//. 6:[1065]. Available from: <http://ovidsp.ovid.com/ovidweb.cgi?T=JS&PAGE=reference&D=cctr&NEWS=N&AN=CN-01665958>.

114. Kullich W SBAMGCSHME. Dynamic spinal traction in inpatient rehabilitation of low back pain: Sustained improvement of pain experience 2012 [updated 2012//. Jahrestagung]. Available from: <http://ovidsp.ovid.com/ovidweb.cgi?T=JS&PAGE=reference&D=cctr&NEWS=N&AN=CN-00983516>.

115. Lam FCG, M. W. Reoperations after decompression for lumbar spinal stenosis. World Neurosurg. 2011;76(1-2):51-3.

116. Lavyne Mh BMH. Epidural steroids, postoperative morbidity, and recovery in patients undergoing microsurgical lumbar discectomy 1992 [updated 1992//. 1:[90]. Available from: <http://ovidsp.ovid.com/ovidweb.cgi?T=JS&PAGE=reference&D=cctr&NEWS=N&AN=CN-00084901>.

117. Lee JHK, J. H.; Kim, J. H.; Kim, H. S.; Min, W. K.; Park, Y. S.; Lee, K. Y.; Lee, J. H. Efficacy and Safety of Transdermal Buprenorphine versus Oral Tramadol/Acetaminophen in Patients with Persistent Postoperative Pain after Spinal Surgery. Pain Res Manag. 2017;2017:2071494.

118. Lee GWY, H. S.; Yeom, J. S.; Ahn, M. W. The Efficacy of Vitamin C on Postoperative Outcomes after Posterior Lumbar Interbody Fusion: A Randomized, Placebo-Controlled Trial. Clin. 2017;9(3):317-24.

119. Lee JHK, J. H.; Kim, H. S.; Min, W. K.; Park, Y. S.; Lee, K. Y.; Lee, J. H. Efficacy and Safety of Transdermal Buprenorphine versus Oral Tramadol/Acetaminophen in Patients with Persistent Postoperative Pain after Spinal Surgery. Pain Research and Management. 2017;2017 (no pagination)(2071494).

120. Lee Gw YHSYJSAMW. The Efficacy of Vitamin C on Postoperative Outcomes after Posterior Lumbar Interbody Fusion: a Randomized, Placebo-Controlled Trial: Korean Orthopaedic Association (E-mail: ortho@koa.or.kr); 2017 [updated 2017//. 3:[317]. Available from: <http://ovidsp.ovid.com/ovidweb.cgi?T=JS&PAGE=reference&D=cctr&NEWS=N&AN=CN-01425825>.

121. Lee Jh KJHKJHKHSMWKPYSLKYLJH. Efficacy and Safety of Transdermal Buprenorphine versus Oral Tramadol/Acetaminophen in Patients with Persistent Postoperative Pain after Spinal Surgery 2017 [updated 2017//. 2071494]. Available from: <http://ovidsp.ovid.com/ovidweb.cgi?T=JS&PAGE=reference&D=cctr&NEWS=N&AN=CN-01599690>.

122. Li H DWGZDYWH. Multimodal pain control protocol after lumbar spinal fusion: Journal of Clinical Rehabilitative Tissue Engineering Research; 2014 [updated 2014//. 53:[8656]. Available from: <http://ovidsp.ovid.com/ovidweb.cgi?T=JS&PAGE=reference&D=cctr&NEWS=N&AN=CN-01129399>.

123. Lindgreen PR, N.; Nielsen, C. V.; Lomborg, K. Interdisciplinary Cognitive-Behavioral Therapy as Part of Lumbar Spinal Fusion Surgery Rehabilitation: Experience of Patients With Chronic Low Back Pain. Orthop Nurs. 2016;35(4):238-47.

124. Lindgreen PR, Nanna; Vinther Nielsen, Claus; Lomborg, Kirsten. Interdisciplinary Cognitive-Behavioral Therapy as Part of Lumbar Spinal Fusion Surgery Rehabilitation. Orthop Nurs. 2016;35(4):238-47.

125. Liow MHLG, G. S.; Yeo, W.; Ling, Z. M.; Yue, W. M.; Guo, C. M.; Tan, S. B. Time Taken to Return to Work Does Not Influence Outcomes of Minimally Invasive Transforaminal Lumbar Interbody Fusion: A 5-Year Follow-Up Study. Spine. 2019;44(7):503-9.

126. Liu SKS, Y. L.; Ding, W. Y.; Yang, D. L.; Ma, L.; Yang, S. D. The effect of systematic lower-limb rehabilitation training in elderly patients undergoing lumbar fusion surgery: a retrospective study. Oncotarget. 2017;8(68):112720-6.

127. Lobner ML, M.; Konnopka, A.; Meisel, H. J.; Gunther, L.; Meixensberger, J.; Stengler, K.; Angermeyer, M. C.; Konig, H. H.; Riedel-Heller, S. G. Inpatient or outpatient rehabilitation after herniated disc surgery? - Setting-specific preferences, participation and outcome of rehabilitation. PLoS ONE. 2014;9(3):e89200.

128. Lobner MS, J.; Luppa, M.; Konnopka, A.; Meisel, H. J.; Gunther, L.; Meixensberger, J.; Stengler, K.; Angermeyer, M. C.; Konig, H. H.; Riedel-Heller, S. G. Choosing the right rehabilitation setting after herniated disc surgery: Motives, motivations and expectations from the patients' perspective. PLoS ONE. 2017;12(8):e0183698.

129. Lumawig JMY, A.; Watanabe, K. Dose-dependent inhibition of diclofenac sodium on posterior lumbar interbody fusion rates. Spine J. 2009;9(5):343-9.

130. Luo SFZ, D. J. Core stabilization exercises, implant fixation and lumbar fusion for degenerative lumbar spondylolisthesis: Lumbar function evaluation. [Chinese]. Chinese Journal of Tissue Engineering Research. 2015;19(31):5036-40.

131. Machado GCBPM. Early comprehensive physiotherapy after lumbar spine surgery (PEDro synthesis). BJSM online. 2018;52(2):96-7.

132. Maffei P. Massage interest in treatment of premature postoperative pain: Controlled randomized trial. [French]; Interet du massage dans le traitement de douleurs postoperatoires precoces: etude controlee randomisee. Kinesitherapie. 2014;14(145):16-25.

133. Magnaes BK, I.; Dullerud, R.; Grundnes, O.; Haagensen, O.; Indahl, A.; Ljunggren, A. E.; Laerum, E.; Nygaard, O.; Salvesen, R. [Evaluation of methods in the treatment of lumbar disk prolapse with root avulsion]. Tidsskr Nor Laegeforen. 2002;122(7):718-20.

134. Magnussen LH. Surgery with disc prosthesis may produce better outcomes than multidisciplinary rehabilitation for patients with chronic low back pain. J Physiother. 2011;57(4):257.

135. Malik ATJ, N.; Kim, J.; Yu, E.; Khan, S. N. Continued Inpatient Care After Elective 1- to 2-level Posterior Lumbar Fusions Increases 30-day Postdischarge Readmissions and Complications. Clin Spine Surg. 2018;31(9):E453-E9.

136. Malik ATK, J.; Yu, E.; Khan, S. N. Discharge to Inpatient Care Facility After Anterior Lumbar Interbody Fusion: Incidence, Predictors, and Postdischarge Outcomes. World Neurosurg. 2019;122:e584-e90.

137. Manchikanti LF, F. J. E.; Pampati, V.; Cash, K. A.; Benyamin, R. M.; Hirsch, J. A. Cost utility analysis of caudal epidural injections in the treatment of lumbar disc herniation, axial or discogenic low back pain, central spinal stenosis, and post lumbar surgery syndrome. Pain physician. 2013;16(3):E129-E43.

138. Mancuso CA, Rigaud MC, Duculan R, Cammisa FP, Sama AA, Hughes AP, et al. Improvement in multiple domains of functional status with increasing physical activity after lumbar surgery: Longitudinal analysis. European Spine Journal. 2019;28:2931-2.

139. Mancuso CA, Rigaud MC, Duculan R, Cammisa FP, Sama AA, Hughes AP, et al. Fostering physical activity after complex lumbar spine surgery: A randomized trial. European Spine Journal. 2019;28:2761.

140. Mancuso Ca RMDRCFPSAAHAPLDRHRCSHS. 51. Fostering physical activity after complex lumbar spine surgery: a randomized trial: Elsevier Inc.; 2019 [updated 2019//. 9:[S25]. Available from: <http://ovidsp.ovid.com/ovidweb.cgi?T=JS&PAGE=reference&D=cctr&NEWS=N&AN=CN-01978398>.

141. Mancuso CA, Rigaud MC, Wellington B, Duculan R, Cammisa FP, Sama AA, et al. Qualitative assessment of patients' perspectives and willingness to improve healthy lifestyle physical activity after lumbar surgery. European spine journal : official publication of the European Spine Society, the European Spinal Deformity Society, and the European Section of the Cervical Spine Research Society. 2021;30(1):200-7.

142. Manniche CA, K.; Lauritsen, B.; Vinterberg, H.; Karbo, H.; Abildstrup, S.; Fischer-Nielsen, K.; Krebs, R.; Ibsen, K. Intensive dynamic back exercises with or without hyperextension in chronic back pain after surgery for lumbar disc protrusion. A clinical trial. Spine. 1993;18(5):560-7.

143. Manniche CS, H. F.; Braendholt, L.; Christensen, B. H.; Christophersen, L.; Ellegaard, B.; Heilbuth, A.; Ingerslev, M.; Jorgensen, O. E.; Larsen, E.; Lorentzen, L.; Nielsen, C. J.; Nielsen, H.; Windelin, M. Clinical trial of postoperative dynamic back exercises after first lumbar discectomy. Spine. 1993;18(1):92-7.

144. Manniche C SHFBLCBHCLEBHAIMJOELE. Clinical trial of postoperative dynamic back exercises after first lumbar discectomy 1993 [updated 1993//. 1:[92]. Available from: <http://ovidsp.ovid.com/ovidweb.cgi?T=JS&PAGE=reference&D=cctr&NEWS=N&AN=CN-00090934>.

145. Manniche C AKLBVHKHASF-NKKRIK. Intensive dynamic back exercises with or without hyperextension in chronic back pain after surgery for lumbar disc protrusion. A clinical trial 1993 [updated 1993//. 5:[560]. Available from: <http://ovidsp.ovid.com/ovidweb.cgi?T=JS&PAGE=reference&D=cctr&NEWS=N&AN=CN-00092862>.

146. Manniche C LBVH. Peroperative prednisolone fails to improve the clinical outcome following surgery for prolapsed lumbar intervertebral disc. A randomized controlled trial 1994 [updated 1994//. 1:[30]. Available from: <http://ovidsp.ovid.com/ovidweb.cgi?T=JS&PAGE=reference&D=cctr&NEWS=N&AN=CN-00099175>.

147. Mannion AFD, R.; Dvorak, J.; Muntener, M.; Grob, D. A randomised controlled trial of post-operative rehabilitation after surgical decompression of the lumbar spine. Eur Spine J. 2007;16(8):1101-17.

148. Mannion Af DRDJMMGD. A randomised controlled trial of post-operative rehabilitation after surgical decompression of the lumbar spine 2007 [updated 2007//. 8:[1101]. Available from: <http://ovidsp.ovid.com/ovidweb.cgi?T=JS&PAGE=reference&D=cctr&NEWS=N&AN=CN-00618361>.

149. Mannion AFD, R.; Dvorak, J.; Grob, D. Five-year outcome of surgical decompression of the lumbar spine without fusion. Eur Spine J. 2010;19(11):1883-91.

150. Master H, Pennings JS, Bley J, Robinette PE, Haug CM, Coronado RA, et al. How many steps per day during the early postoperative period is associated with patient-reported outcomes of disability, pain, and opioid use after lumbar spine surgery? Archives of physical medicine and rehabilitation. 2021.

151. Mastronardi LP, M.; Puzzilli, F.; Tatta, C.; Sonntag, V. K. H.; Long, D. M.; Elias, W. J.; Burchiel, K. J.; Benzel, E. C. Efficacy of the morphine-Adcon-L compound in the management of postoperative pain after lumbar microdiscectomy. Neurosurgery. 2002;50(3):518-25.

152. Maybin S, Pennings J, Archer K. The impact of postoperative physical therapy on patient-reported outcomes in patients undergoing spine surgery. PM and R. 2018;10(9 Supplement):S41.

153. Mayer Tg GRJKJMDDJAC. A randomized clinical trial of treatment for lumbar segmental rigidity 2004 [updated 2004//. 20:[2199]. Available from: <http://ovidsp.ovid.com/ovidweb.cgi?T=JS&PAGE=reference&D=cctr&NEWS=N&AN=CN-00520795>.

154. McGregor AHD, C. J.; Morris, T. P.; Morris, S.; Jamrozik, K. Function after spinal treatment, exercise and rehabilitation (FASTER): improving the functional outcome of spinal surgery. BMC Musculoskelet Disord. 2010;11:17.

155. McGregor AHD, C. J.; Morris, T. P.; Morris, S.; Jamrozik, K. ISSLS prize winner: Function After Spinal Treatment, Exercise, and Rehabilitation (FASTER): a factorial randomized trial to determine whether the functional outcome of spinal surgery can be improved. Spine. 2011;36(21):1711-20.

156. McGregor AHD, C. J.; Morris, T. P.; Morris, S.; Jamrozik, K. Function after spinal treatment, exercise and rehabilitation (FASTER): A factorial randomised trial to determine whether the functional outcome of spinal surgery can be improved. []. Spine. 2011;03.

157. McGregor Ah DCJMTPMSJK. Function after spinal treatment, exercise and rehabilitation (FASTER): a factorial randomised trial to determine whether the functional outcome of spinal surgery can be improved 2011 [updated 2011//. Available from: <http://ovidsp.ovid.com/ovidweb.cgi?T=JS&PAGE=reference&D=cctr&NEWS=N&AN=CN-01729388>.

158. McGregor Ah DCJMTPMSJK. ISSLS prize winner: function After Spinal Treatment, Exercise, and Rehabilitation (FASTER): a factorial randomized trial to determine whether the functional outcome of spinal surgery can be improved 2011 [updated 2011//. 21:[1711]. Available from: <http://ovidsp.ovid.com/ovidweb.cgi?T=JS&PAGE=reference&D=cctr&NEWS=N&AN=CN-00814137>.

159. Mehren CK, A.; Siepe, C.; Grochulla, F.; Mayer, H. M. Minimal invasive anterior midline approach to L<inf>2</inf>-L<inf>5</inf>. [German]; Minimalinvasive ventrale mittellinienzugange L<inf>2</inf>-L <inf>5</inf>. Oper. 2010;22(5-6):573-81.

160. Meziat Filho NS, S.; Rocha, R. M. Long-term effects of a stabilization exercise therapy for chronic low back pain. Manual Ther. 2009;14(4):444-7.

161. Mirza SK. Surgery and physical therapy likely yield similar outcomes in spinal stenosis. Evidence-Based Medicine. 2016;21(1):31.

162. Mondanaro JFH, P.; Lonner, B.; Shepp, J.; Lichtensztein, M.; Loewy, J. V. Music Therapy Increases Comfort and Reduces Pain in Patients Recovering From Spine Surgery. Am J Orthop. 2017;46(1):E13-E22.

163. Mondanaro Jf HPLBSJLMLJV. Music Therapy Increases Comfort and Reduces Pain in Patients Recovering From Spine Surgery 2017 [updated 2017//. 1:[E13]. Available from: <http://ovidsp.ovid.com/ovidweb.cgi?T=JS&PAGE=reference&D=cctr&NEWS=N&AN=CN-01306751>.

164. Monticone MF, S.; Teli, M.; Rocca, B.; Foti, C.; Lovi, A.; Brayda Bruno, M. Management of catastrophising and kinesiophobia improves rehabilitation after fusion for lumbar spondylolisthesis and stenosis. A randomised controlled trial. Eur Spine J. 2014;23(1):87-95.

165. Monticone M FSTMRBFCLABBM. Management of catastrophising and kinesiophobia improves rehabilitation after fusion for lumbar spondylolisthesis and stenosis. A randomised controlled trial: Springer Verlag; 2014 [updated 2014//. 1:[87]. Available from: <http://ovidsp.ovid.com/ovidweb.cgi?T=JS&PAGE=reference&D=cctr&NEWS=N&AN=CN-00988593>.

166. Monticone MA, E.; Rocca, B.; Foti, C.; Ferrante, S. Responsiveness and minimal clinically important changes for the Tampa Scale of Kinesiophobia after lumbar fusion during cognitive behavioral rehabilitation. Eur J Phys Rehabil Med. 2017;53(3):351-8.

167. Morfeld MH, Kerstin; Moller, Jens Uwe; Fox, Mario; Hoder, Jurgen; Hintze, Ralf; Arlt, Andreas Christof; Wessinghage, Thomas; Koch, Uwe. Cognitive-behavioral therapy interventions in patients following initial surgical treatment for herniated disks and their return to work. Verhaltenstherapie & Verhaltensmedizin. 2006;27(3):338-62.

168. Moussa WMK, W. Percutaneous radiofrequency facet capsule denervation as an alternative target in lumbar facet syndrome. Clin Neurol Neurosurg. 2016;150:96-104.

169. Nair BN, A. B.; Elek, E. Long-term results of minimally invasive lumbar disc surgery. Acta Chir Hung. 1995;35(3-4):297-300.

170. Neva M IOEPLTDJMIHHA. Effect of lumbar spine fusion and postoperative exercise therapy on physical activity and kinesiophobia: a randomized controlled trial: Elsevier Inc.; 2014 [updated 2014//. 11:[S47]. Available from: <http://ovidsp.ovid.com/ovidweb.cgi?T=JS&PAGE=reference&D=cctr&NEWS=N&AN=CN-01023313>.

171. North American Spine Society Board of D. Spine Patient Outcome Research Trial (SPORT): multi-center randomized clinical trial of surgical and non-surgical approaches to the treatment of low back pain. Spine J. 2003;3(6):417-9.

172. Nykvist FH, M.; Alaranta, H.; Kaitsaari, M. Severe sciatica: a 13-year follow-up of 342 patients. Eur Spine J. 1995;4(6):335-8.

173. Oestergaard LGN, C. V.; Bunger, C. E.; Sogaard, R.; Fruensgaard, S.; Helmig, P.; Christensen, F. B. The effect of early initiation of rehabilitation after lumbar spinal fusion: a randomized clinical study. Spine. 2012;37(21):1803-9.

174. Oestergaard LGM, T.; Bunger, C. E.; Christensen, F. B. The Canadian Occupational Performance Measure's semi-structured interview: its applicability to lumbar spinal fusion patients. A prospective randomized clinical study. Eur Spine J. 2012;21(1):115-21.

175. Oestergaard LGN, C. V.; Bunger, C. E.; Sogaard, R.; Fruensgaard, S.; Helmig, P.; Christensen, F. B. The effect of early initiation of rehabilitation after lumbar spinal fusion - A randomized clinical study. []. Spine. 2012;04.

176. Oestergaard LGM, T.; Bunger, C. E.; Christensen, F. B. The Canadian Occupational Performance Measure's semistructured interview: Its applicability to lumbar spinal fusion patients. A prospective randomized clinical study. Eur Spine J. 2012;21(1):115-21.

177. Oestergaard Lg MTBCECFB. The Canadian Occupational Performance Measure's semi-structured interview: its applicability to lumbar spinal fusion patients. A prospective randomized clinical study 2012 [updated 2012//. 1:[115]. Available from: <http://ovidsp.ovid.com/ovidweb.cgi?T=JS&PAGE=reference&D=cctr&NEWS=N&AN=CN-00840732>.

178. Oestergaard Lg NCVBCESRFSHPCFB. The effect of early initiation of rehabilitation after lumbar spinal fusion: a randomized clinical study 2012 [updated 2012//. 21:[1803]. Available from: <http://ovidsp.ovid.com/ovidweb.cgi?T=JS&PAGE=reference&D=cctr&NEWS=N&AN=CN-00969532>.

179. Oestergaard LGC, F. B.; Nielsen, C. V.; Bunger, C. E.; Fruensgaard, S.; Sogaard, R. Early versus late initiation of rehabilitation after lumbar spinal fusion: economic evaluation alongside a randomized controlled trial. Spine. 2013;38(23):1979-85.

180. Oestergaard Lg CFBNCVBCEFSSR. Early versus late initiation of rehabilitation after lumbar spinal fusion: economic evaluation alongside a randomized controlled trial 2013 [updated 2013//. 23:[1979]. Available from: <http://ovidsp.ovid.com/ovidweb.cgi?T=JS&PAGE=reference&D=cctr&NEWS=N&AN=CN-00991756>.

181. Oestergaard Lg CFBBCEHRHPSRNCV. Can a case manager reduce functional disability and absence from work for lumbar spinal fusion patients? A clinical randomized study with a two years follow-up: Springer Verlag; 2015 [updated 2015//. 6 SUPPL. 1:[S771]. Available from: <http://ovidsp.ovid.com/ovidweb.cgi?T=JS&PAGE=reference&D=cctr&NEWS=N&AN=CN-01135931>.

182. Oestergaard LG, Christensen FB, Bunger CE, Sogaard R, Holm R, Helmig P, et al. Does adding case management to standard rehabilitation affect functional ability, pain, or the rate of return to work after lumbar spinal fusion? A randomized controlled trial with two-year follow-up. Clinical rehabilitation. 2020;34(3):357-68.

183. Oestergaard Lg CFBNCVBCEHRHPSR. Case manager-assisted rehabilitation for lumbar spinal fusion patients: an economic evaluation alongside a randomized controlled trial with two-year follow-up. Clinical rehabilitation. 2020;34(4):460.

184. Oestergaard Lg CFBBCESRHRHPNCV. Does adding case management to standard rehabilitation affect functional ability, pain, or the rate of return to work after lumbar spinal fusion? A randomized controlled trial with two-year follow-up. Clinical rehabilitation. 2020;34(3):357.

185. Oestergaard LG, Christensen FB, Nielsen CV, Bünger CE, Holm R, Helmig P, et al. Case manager–assisted rehabilitation for lumbar spinal fusion patients: an economic evaluation alongside a randomized controlled trial with two-year follow-up. Clinical Rehabilitation. 2020;34(4):460-70.

186. Ogon MT, A. Keel-based lumbar total disk replacement: Prodisc-L and Prodisc-O. [German]; Kielbasierte lumbale Bandscheibenprothesen: Prodisc-L und Prodisc-O. Oper. 2010;22(5-6):593-607.

187. Oh I-S HKYKYYKSI. Gender-and age-specific analgesia for early postoperative pain management after lumbar decompressive surgery: a randomized clinical trial: Springer Verlag; 2018 [updated 2018//. S646]. Available from: <http://ovidsp.ovid.com/ovidweb.cgi?T=JS&PAGE=reference&D=cctr&NEWS=N&AN=CN-01647385>.

188. Ohtori SK, T.; Yamashita, M.; Yamauchi, K.; Inoue, G.; Suzuki, M.; Orita, S.; Eguchi, Y.; Ochiai, N.; Kishida, S.; Takaso, M.; Kuniyoshi, K.; Aoki, Y.; Ishikawa, T.; Arai, G.; Miyagi, M.; Kamoda, H.; Suzuki, M.; Nakamura, J.; Toyone, T.; Takahashi, K. Surgical versus nonsurgical treatment of selected patients with discogenic low back pain: a small-sized randomized trial. Spine. 2011;36(5):347-54.

189. Ohtori S KTYMYKIGSMOSEYONKSTMKKAY. Surgical versus nonsurgical treatment of selected patients with discogenic low back pain: a small-sized randomized trial 2011 [updated 2011//. 5:[347]. Available from: <http://ovidsp.ovid.com/ovidweb.cgi?T=JS&PAGE=reference&D=cctr&NEWS=N&AN=CN-00784284>.

190. O'Neill P KCBSBAE. Use of intrathecal morphine for postoperative pain relief following lumbar spine surgery 1985 [updated 1985//. 3:[413]. Available from: <http://ovidsp.ovid.com/ovidweb.cgi?T=JS&PAGE=reference&D=cctr&NEWS=N&AN=CN-00039003>.

191. Oosterhuis T ORWvDJMPWCdBMRBJEV-LCLAM. Early rehabilitation after lumbar disc surgery is not effective or cost-effective compared to no referral: a randomised trial and economic evaluation 2017 [updated 2017//. 3:[144]. Available from: <http://ovidsp.ovid.com/ovidweb.cgi?T=JS&PAGE=reference&D=cctr&NEWS=N&AN=CN-01412580>.

192. Ostelo RWJGDV, H. C. W.; Vlaeyen, J. W. S.; Kerckhoffs, M. R.; Berfelo, W. M.; Wolters, P. M. J. C.; Van den Brandt, P. A. Behavioral graded activity following first-time lumbar disc surgery: 1-Year results of a randomized clinical trial. Spine. 2003;28(16):1757-65.

193. Ostelo Rw dVHCVJWKMRBWMWPMvdBPA. Behavioral graded activity following first-time lumbar disc surgery: 1-year results of a randomized clinical trial 2003 [updated 2003//. 16:[1757]. Available from: <http://ovidsp.ovid.com/ovidweb.cgi?T=JS&PAGE=reference&D=cctr&NEWS=N&AN=CN-00451770>.

194. Ostelo Rw dVHCBMWKMRVJWWPMvdBPA. Effectiveness of behavioral graded activity after first-time lumbar disc surgery: short term results of a randomized controlled trial 2003 [updated 2003//. 6:[637]. Available from: <http://ovidsp.ovid.com/ovidweb.cgi?T=JS&PAGE=reference&D=cctr&NEWS=N&AN=CN-00472496>.

195. Ostelo RWG, M. E.; de Vet, H. C.; van den Brandt, P. A. Economic evaluation of a behavioral-graded activity program compared to physical therapy for patients following lumbar disc surgery. Spine. 2004;29(6):615-22.

196. Ostelo Rw GMEdVHCvdBPA. Economic evaluation of a behavioral-graded activity program compared to physical therapy for patients following lumbar disc surgery 2004 [updated 2004//. 6:[615]. Available from: <http://ovidsp.ovid.com/ovidweb.cgi?T=JS&PAGE=reference&D=cctr&NEWS=N&AN=CN-00486820>.

197. Ozkara GOO, M.; Ozkara, E.; Armagan, O.; Arslantas, A.; Atasoy, M. A. Effectiveness of physical therapy and rehabilitation programs starting immediately after lumbar disc surgery. Turk. 2015;25(3):372-9.

198. Park SYM, S. H.; Park, M. S.; Oh, K. S.; Lee, H. M. The effects of ketorolac injected via patient controlled analgesia postoperatively on spinal fusion. Yonsei Med J. 2005;46(2):245-51.

199. Paulsen RT, Carreon LY, Andersen MO. Recovery after surgery for lumbar disk herniation, a randomized clinical trial comparing the effect of supervised rehabilitation versus home exercises. European Spine Journal. 2019;28:2770.

200. Paulsen RTC, L. Y.; Andersen, M. Patient Reported Outcomes after Surgery for Lumbar Disk Herniation, a Randomized Controlled Trial Comparing the Effects of Referral to Municipal Physical Rehabilitation Versus no Referral. Spine. 2019.

201. Paulsen RT, Carreon LY, Andersen MO. Patient-reported Outcomes After Surgery for Lumbar Disc Herniation, a Randomized Controlled Trial Comparing the Effects of Referral to Municipal Physical Rehabilitation Versus No Referral. Spine. 2020;45(1):3-9.

202. Paulsen RT, Rasmussen J, Carreon LY, Andersen MO. Return to work after surgery for lumbar disc herniation, secondary analyses from a randomized controlled trial comparing supervised rehabilitation versus home exercises. The spine journal : official journal of the North American Spine Society. 2020;20(1):41-7.

203. Paulsen Rt CLYAMO. Patient-reported Outcomes After Surgery for Lumbar Disc Herniation, a Randomized Controlled Trial Comparing the Effects of Referral to Municipal Physical Rehabilitation Versus No Referral. Spine. 2020;45(1):3.

204. Paulsen Rt RJCLYAMO. Return to work after surgery for lumbar disc herniation, secondary analyses from a randomized controlled trial comparing supervised rehabilitation versus home exercises. Spine journal. 2020;20(1):41.

205. Pekar L SJ. Systemic oral enzyme therapy after lumbar disc herniation surgery 2009 [updated 2009//. 4:[166]. Available from: <http://ovidsp.ovid.com/ovidweb.cgi?T=JS&PAGE=reference&D=cctr&NEWS=N&AN=CN-00784293>.

206. Quinlan P DJFKMPCLTDCDR. Effects of Localized Cold Therapy on Pain in Postoperative Spinal Fusion Patients: a Randomized Control Trial 2017 [updated 2017//. 5:[344]. Available from: <http://ovidsp.ovid.com/ovidweb.cgi?T=JS&PAGE=reference&D=cctr&NEWS=N&AN=CN-01426065>.

207. Quinlan PD, Jack; Fields, Kara; Madamba, Pia; Colman, Lisa; Tinca, Daniela; Drake, Regina Cannon. Effects of Localized Cold Therapy on Pain in Postoperative Spinal Fusion Patients: A Randomized Control Trial. Orthop Nurs. 2017;36(5):344-9.

208. Radin El BRS. Phenylbutazone for prolapsed discs? 1968 [updated 1968//. 7570:[736]. Available from: <http://ovidsp.ovid.com/ovidweb.cgi?T=JS&PAGE=reference&D=cctr&NEWS=N&AN=CN-00002351>.

209. Rainov NGH, V.; Albertz, C.; Burkert, W. Transcutaneous electrical nerve stimulation (TENS) for acute postoperative pain after spinal surgery. Eur J Pain. 1994;15(2-3):44-9.

210. Rainov Ng HVACBW. Transcutaneous electrical nerve stimulation (TENS) for acute postoperative pain after spinal surgery 1994 [updated 1994//. 2-3:[44]. Available from: <http://ovidsp.ovid.com/ovidweb.cgi?T=JS&PAGE=reference&D=cctr&NEWS=N&AN=CN-00180574>.

211. Rasmussen SK-M, D. S.; Lauridsen, L. R.; Jensen, S. E.; Mandoe, H.; Gerlif, C.; Kehlet, H. Epidural steroid following discectomy for herniated lumbar disc reduces neurological impairment and enhances recovery: a randomized study with two-year follow-up. Spine. 2008;33(19):2028-33.

212. Rasmussen SK-M, D. S.; Lauridsen, L. R.; Jensen, S. E. H.; Mandoe, H.; Gerlif, C.; Kehlet, H. Epidural steroid following discectomy for herniated lumbar disc reduces neurological impairment and enhances recovery: A randomized study with two-year follow-up. Spine. 2008;33(19):2028-33.

213. Reuben Ss EEFRKSRBBJLAJ. The effect of cyclooxygenase-2 inhibition on acute and chronic donor-site pain after spinal-fusion surgery.retraction in Neal JM. Reg Anesth Pain Med. 2009 Mar-Apr;34(2): 184; PMID: 19288610 2006 [updated 2006//. 1:[6]. Available from: <http://ovidsp.ovid.com/ovidweb.cgi?T=JS&PAGE=reference&D=cctr&NEWS=N&AN=CN-00691731>.

214. Richter Hp KETRBWGW. Results of applying ADCON-L gel after lumbar discectomy: the German ADCON-L study 2001 [updated 2001//. 2 Suppl:[179]. Available from: <http://ovidsp.ovid.com/ovidweb.cgi?T=JS&PAGE=reference&D=cctr&NEWS=N&AN=CN-00374448>.

215. Roberts NS, R.; Bennett, S.; Cape, J.; Norton, R.; Kilburn, P. Health beliefs and rehabilitation after lumbar disc surgery. J Psychosom Res. 1984;28(2):139-44.

216. Rolving NN, C. V.; Christensen, F. B.; Holm, R.; Bunger, C. E.; Oestergaard, L. G. Preoperative cognitive-behavioural intervention improves in-hospital mobilisation and analgesic use for lumbar spinal fusion patients. BMC Musculoskelet Disord. 2016;17:217.

217. Rush PJS, A. Physician perceptions of the value of physical modalities in the treatment of musculoskeletal disease. Br J Rheumatol. 1994;33(6):566-8.

218. Rushton AH, N. R.; Calvert, M.; Heap, A.; White, L.; Goodwin, P. C. Physiotherapy Post Lumbar Discectomy: Prospective Feasibility and Pilot Randomised Controlled Trial. PLoS ONE. 2015;10(11):e0142013.

219. Rushton A HNRCMHAWLGPC. Physiotherapy Post Lumbar Discectomy: prospective Feasibility and Pilot Randomised Controlled Trial: Public Library of Science; 2015 [updated 2015//. 11:[e0142013]. Available from: <http://ovidsp.ovid.com/ovidweb.cgi?T=JS&PAGE=reference&D=cctr&NEWS=N&AN=CN-01169007>.

220. Rushton A GPHAWLCMHN. Quest physiotherapy post lumbar discectomy: pilot and feasibility study to inform the development of a randomised controlled trial: Elsevier Ltd; 2015 [updated 2015//. eS1304]. Available from: <http://ovidsp.ovid.com/ovidweb.cgi?T=JS&PAGE=reference&D=cctr&NEWS=N&AN=CN-01126408>.

221. Rushton AH, N. R.; Heap, A.; White, L.; Calvert, M.; Goodwin, P. C. Patient and physiotherapist perceptions of rehabilitation following primary lumbar discectomy: a qualitative focus group study embedded within an external pilot and feasibility trial. BMJ Open. 2017;7(4):e015878.

222. Rushton A, Masson A, Jadhakhan F, Heneghan N, Staal JB, Verra M, et al. The early patient journey following lumbar spinal fusion surgery: a qualitative study using semi structured interviews and weekly patient diaries. Physiotherapy (United Kingdom). 2020;107(Supplement 1):e61-e2.

223. Schiavolin SB, M.; Visintini, S.; Schiariti, M.; Leonardi, M.; Ferroli, P. Change in quality of life, disability, and well-being after decompressive surgery: results from a longitudinal study. Int J Rehabil Res. 2015;38(4):357-63.

224. Schroter JL, M.; Hartmann, F.; Gercek, E. [Structured rehabilitation after lumbar spine surgery : subacute treatment phase]. Orthopade. 2014;43(12):1089-95.

225. Schroter J LMHFGE. Structured rehabilitation after lumbar spine surgery : subacute treatment phase 2014 [updated 2014//. 12:[1089]. Available from: <http://ovidsp.ovid.com/ovidweb.cgi?T=JS&PAGE=reference&D=cctr&NEWS=N&AN=CN-01086746>.

226. Scrimshaw SVM, C. G. Randomized controlled trial of neural mobilization after spinal surgery. Spine. 2001;26(24):2647-52.

227. Scrimshaw Sv MCG. Randomized controlled trial of neural mobilization after spinal surgery 2001 [updated 2001//. 24:[2647]. Available from: <http://ovidsp.ovid.com/ovidweb.cgi?T=JS&PAGE=reference&D=cctr&NEWS=N&AN=CN-00376212>.

228. Sekiguchi H IGNTITSWUKMMTNTM. Loxoprofen sodium and celecoxib for postoperative pain in patients after spinal surgery: a randomized comparative study 2015 [updated 2015//. 4:[617]. Available from: <http://ovidsp.ovid.com/ovidweb.cgi?T=JS&PAGE=reference&D=cctr&NEWS=N&AN=CN-01256005>.

229. Selkowitz DMK, K.; Poppert, E. M.; Flanagan, S. P.; Matthews, N. D.; Beneck, G. J.; Popovich Jr, J. M.; Lona, J. R.; Yamada, K. A.; Burke, W. S.; Ervin, C.; Powers, C. M. The immediate and long-term effects of exercise and patient education on physical, functional, and quality-of-life outcome measures after single-level lumbar microdiscectomy: A randomized controlled trial protocol. BMC Musculoskelet Disord. 2006;7 (no pagination)(70).

230. Shabat SF, Y.; Arinzon, Z.; Adunsky, A.; Catz, A.; Gepstein, R. Gender differences as an influence on patients' satisfaction rates in spinal surgery of elderly patients. Eur Spine J. 2005;14(10):1027-32.

231. Shatrova VPM, K. T. [Mexidol in the post-surgery rehabilitation of patients with degenerative-dystrophic diseases of the spine]. Zh Nevrol Psikhiatr Im S S Korsakova. 2008;108(12):68-70.

232. Shimia ME, B.; Akbari, M. G.; Habibzadeh, A.; Shahidi, B.; Ghabili, K. Effect of body mass index on inpatient rehabilitation outcomes after single-level discectomy. Neurosurgery Quarterly. 2015;25(2):260-3.

233. Shin SHH, B. W.; Keum, H. J.; Lee, S. J.; Park, S. J.; Lee, S. H. Epidural Steroids After a Percutaneous Endoscopic Lumbar Discectomy. Spine. 2015;40(15):E859-65.

234. Shin Sh HBWKHJLSJPSJLSH. Epidural Steroids After a Percutaneous Endoscopic Lumbar Discectomy: Lippincott Williams and Wilkins; 2015 [updated 2015//. 15:[E859]. Available from: <http://ovidsp.ovid.com/ovidweb.cgi?T=JS&PAGE=reference&D=cctr&NEWS=N&AN=CN-01081517>.

235. Siepe CJH, F.; Beisse, R.; Mayer, H. M.; Korge, A. Treatment of dynamic spinal canal stenosis with an interspinous spacer. [German]; Die Behandlung der dynamischen Spinalkanalstenose mit einem interspinosen Spacer. Oper. 2010;22(5-6):524-35.

236. Skall Fh MCNCJ. Intensive back exercises 5 weeks after surgery of lumbar disk prolapse. A prospective, randomized multicenter trial with a historical control group 1994 [updated 1994//. 5:[643]. Available from: <http://ovidsp.ovid.com/ovidweb.cgi?T=JS&PAGE=reference&D=cctr&NEWS=N&AN=CN-00101325>.

237. Skidmore GA, S. J.; Bergin, C.; Ross, D.; Butler, J.; Suthar, M.; Rittenberg, J. Cost-effectiveness of the X-STOP interspinous spacer for lumbar spinal stenosis. Spine. 2011;36(5):E345-56.

238. Skolasky RLM, A. M.; Li, D.; Riley, L. H., 3rd; Wegener, S. T. Health behavior change counseling in surgery for degenerative lumbar spinal stenosis. Part I: improvement in rehabilitation engagement and functional outcomes. Arch Phys Med Rehabil. 2015;96(7):1200-7.

239. Skolasky Rl MAMLDRLHWST. Health behavior change counseling in surgery for degenerative lumbar spinal stenosis. Part I: improvement in rehabilitation engagement and functional outcomes 2015 [updated 2015//. 7 // 1 R01HS 017990 (AHRQ) *Agency for Healthcare Research and Quality*:[1200]. Available from: <http://ovidsp.ovid.com/ovidweb.cgi?T=JS&PAGE=reference&D=cctr&NEWS=N&AN=CN-01471575>.

240. Skolasky Rl MAMLDRLHWST. Health behavior change counseling in surgery for degenerative lumbar spinal stenosis. Part II: patient activation mediates the effects of health behavior change counseling on rehabilitation engagement 2015 [updated 2015//. 7 // 1 R01HS 017990 (AHRQ) *Agency for Healthcare Research and Quality*:[1208]. Available from: <http://ovidsp.ovid.com/ovidweb.cgi?T=JS&PAGE=reference&D=cctr&NEWS=N&AN=CN-01473921>.

241. Skolasky RLM, Anica M.; Li, David; Iiiriley, Lee H.; Wegener, Stephen T. Health Behavior Change Counseling in Surgery for Degenerative Lumbar Spinal Stenosis. Part I: Improvement in Rehabilitation Engagement and Functional Outcomes. Arch Phys Med Rehabil. 2015;96(7):1200-7.

242. Skolasky RLM, Anica M.; Li, David; Iiiriley, Lee H.; Wegener, Stephen T. Health Behavior Change Counseling in Surgery for Degenerative Lumbar Spinal Stenosis. Part II: Patient Activation Mediates the Effects of Health Behavior Change Counseling on Rehabilitation Engagement. Arch Phys Med Rehabil. 2015;96(7):1208-14.

243. Skolasky RLM, A. M.; Wegener, S. T.; Riley, L. H., 3rd. Telephone-Based Intervention to Improve Rehabilitation Engagement After Spinal Stenosis Surgery: A Prospective Lagged Controlled Trial. J Bone Joint Surg Am. 2018;100(1):21-30.

244. Skolasky Rl MAMWSTRLH, 3rd. Telephone-Based Intervention to Improve Rehabilitation Engagement After Spinal Stenosis Surgery: a Prospective Lagged Controlled Trial 2018 [updated 2018//. 1:[21]. Available from: <http://ovidsp.ovid.com/ovidweb.cgi?T=JS&PAGE=reference&D=cctr&NEWS=N&AN=CN-01631787>.

245. Skolasky RLM, Anica M.; Wegener, Stephen T.; Riley Iii, Lee H.; Riley, Lee H., 3rd. Telephone-Based Intervention to Improve Rehabilitation Engagement After Spinal Stenosis Surgery: A Prospective Lagged Controlled Trial. Journal of Bone & Joint Surgery, American Volume. 2018;100(1):21-30.

246. Soegaard RC, F. B.; Lauerberg, I.; Bunger, C. E. Lumbar spinal fusion patients' demands to the primary health sector: evaluation of three rehabilitation protocols. A prospective randomized study. Eur Spine J. 2006;15(5):648-56.

247. Soegaard R CFBLILIBCE. Lumbar spinal fusion patients' demands to the primary health sector: evaluation of three rehabilitation protocols. A prospective randomized study 2006 [updated 2006//. 5:[648]. Available from: <http://ovidsp.ovid.com/ovidweb.cgi?T=JS&PAGE=reference&D=cctr&NEWS=N&AN=CN-00560759>.

248. Sogaard R BCELICFB. Cost-effectiveness evaluation of an RCT in rehabilitation after lumbar spinal fusion: a low-cost, behavioural approach is cost-effective over individual exercise therapy: Springer Verlag; 2008 [updated 2008//. 2:[262]. Available from: <http://ovidsp.ovid.com/ovidweb.cgi?T=JS&PAGE=reference&D=cctr&NEWS=N&AN=CN-01750768>.

249. Sogaard R BCELIC, F. B. Cost-effectiveness evaluation of an RCT in rehabilitation after lumbar spinal fusion: a low-cost// behavioural approach is cost-effective over individual exercise therapy 2008 [updated 2008//. 2:[262]. Available from: <http://ovidsp.ovid.com/ovidweb.cgi?T=JS&PAGE=reference&D=cctr&NEWS=N&AN=CN-00631821>.

250. Sokunbi OC, V.; Watt, P.; Moore, A. Experiences of individuals with chronic low back pain during and after their participation in a spinal stabilisation exercise programme - A pilot qualitative study. Manual Ther. 2010;15(2):179-84.

251. Standaert CJ. Core Stabilization for Low Back Pain and Performance. Sport-Orthopadie - Sport-Traumatologie. 2011;27(2):92-8.

252. Steib KP, M.; Brawanski, A.; Lange, M.; Schlaier, J.; Schebesch, K. M. Predictors of facet joint syndrome after lumbar disc surgery. J Clin Neurosci. 2012;19(3):418-22.

253. Storheim KB, L.; Hellum, C.; Gjertsen, O.; Neckelmann, G.; Espeland, A.; Keller, A. Fat in the lumbar multifidus muscles - predictive value and change following disc prosthesis surgery and multidisciplinary rehabilitation in patients with chronic low back pain and degenerative disc: 2-year follow-up of a randomized trial. BMC Musculoskelet Disord. 2017;18 (1) (no pagination)(145).

254. Strom J NCVJLBLM. Does a web-based spine platform featuring social interaction and animated information affect patient reported outcomes in patients undertaking lumbar spine fusion surgery? a randomized clinical trial: Springer Verlag; 2018 [updated 2018//. S596]. Available from: <http://ovidsp.ovid.com/ovidweb.cgi?T=JS&PAGE=reference&D=cctr&NEWS=N&AN=CN-01647361>.

255. Strom J NCVJLBANTLM. A web-based platform to accommodate symptoms of anxiety and depression by featuring social interaction and animated information in patients undergoing lumbar spine fusion: a randomized clinical trial: Elsevier Inc.; 2018 [updated 2018//. Available from: <http://ovidsp.ovid.com/ovidweb.cgi?T=JS&PAGE=reference&D=cctr&NEWS=N&AN=CN-01659502>.

256. Strom J, Hoybye MT, Laursen M, Jorgensen LB, Nielsen CV. Lumbar Spine Fusion Patients' Use of an Internet Support Group: Mixed Methods Study. Journal of medical Internet research. 2019;21(7):e9805.

257. Strom J, Nielsen CV, Jorgensen LB, Andersen NT, Laursen M. A web-based platform to accommodate symptoms of anxiety and depression by featuring social interaction and animated information in patients undergoing lumbar spine fusion: a randomized clinical trial. The spine journal : official journal of the North American Spine Society. 2019;19(5):827-39.

258. Strom J HMTLMJLBNCV. Lumbar Spine Fusion Patients' Use of an Internet Support Group: mixed Methods Study: NLM (Medline); 2019 [updated 2019//. 7:[e9805]. Available from: <http://ovidsp.ovid.com/ovidweb.cgi?T=JS&PAGE=reference&D=cctr&NEWS=N&AN=CN-01962206>.

259. ckvensson Gunilla WGKTRDE. Patients' experience of health three years after structured physiotherapy or surgery for lumbar disc herniation 2013 [updated 2013//. 3:[293]. Available from: <http://ovidsp.ovid.com/ovidweb.cgi?T=JS&PAGE=reference&D=cctr&NEWS=N&AN=CN-00987780>.

260. Tait Mj LJNMPCPVBBAPMC. Improved outcome after lumbar microdiscectomy in patients shown their excised disc fragments: a prospective, double blind, randomised, controlled trial 2009 [updated 2009//. 9:[1044]. Available from: <http://ovidsp.ovid.com/ovidweb.cgi?T=JS&PAGE=reference&D=cctr&NEWS=N&AN=CN-00721085>.

261. Tamburrelli Fc SSSGRDGEPL. Efficacy of "NEURAC" post-operative rehabilitation method in patients who underwent microdiscectomy: Springer-Verlag Italia s.r.l.; 2014 [updated 2014//. S85]. Available from: <http://ovidsp.ovid.com/ovidweb.cgi?T=JS&PAGE=reference&D=cctr&NEWS=N&AN=CN-01023519>.

262. Techy FB, E. C. Implementing an outpatient ambulatory discectomy protocol at a large academic center: A change for the better. World Neurosurg. 2015;83(3):341-2.

263. Than KDC, J. N.; Resnick, D. K.; Shaffrey, C. I.; Ghogawala, Z.; Mummaneni, P. V. How to predict return to work after lumbar discectomy: answers from the NeuroPoint-SD registry. J Neurosurg Spine. 2016;25(2):181-6.

264. Timm KE. A randomized-control study of active and passive treatments for chronic low back pain following L5 laminectomy. J Orthop Sports Phys Ther. 1994;20(6):276-86.

265. Timm KE. A randomized-control study of active and passive treatments for chronic low back pain following L5 laminectomy 1994 [updated 1994//. 6:[276]. Available from: <http://ovidsp.ovid.com/ovidweb.cgi?T=JS&PAGE=reference&D=cctr&NEWS=N&AN=CN-00110226>.

266. Urban MKL, K. M.; Reid, S. C.; Goon, A. K.; Rotundo, V.; Cammisa, F. P., Jr.; Girardi, F. P. Pregabalin Did Not Improve Pain Management After Spinal Fusions. Hss J. 2018;14(1):41-6.

267. Vaccaro ARB, S. T. Indications for instrumentation in degenerative lumbar spinal disorders. Orthopedics. 2000;23(3):260-71; quiz 72-3.

268. Vaid PG, Theresa; Shinkaruk, Kelly; King-Shier, Kathryn. Low-Dose Ketamine Infusions for Highly Opioid-Tolerant Adults Following Spinal Surgery: A Retrospective Before-and-after Study. Pain Manag Nurs. 2016;17(2):150-8.

269. Volchegorskii IAM, K. M. [Effects of 3-hydroxypyridine and succinic acid derivatives on the dynamics of dorsalgia and affective disorders after surgical treatment of disc herniation]. Eksp Klin Farmakol. 2010;73(1):33-9.

270. Volchegorskii Ia MKM. Effects of 3-hydroxypyridine and succinic acid derivatives on the dynamics of dorsalgia and affective disorders after surgical treatment of disc herniation 2010 [updated 2010//. 1:[33]. Available from: <http://ovidsp.ovid.com/ovidweb.cgi?T=JS&PAGE=reference&D=cctr&NEWS=N&AN=CN-00735066>.

271. Volchegorskii IAM, K. M. Effects of 3-hydroxypyridine and succinic acid derivatives on the dynamics of dorsalgia and affective disorders after surgical treatment of disc herniation. [Russian]. Eksperimental'naya i Klinicheskaya Farmakologiya. 2010;73(1):33-9.

272. Wang Rr TV. Effect of acupuncture on pain management in patients before and after lumbar disc protrusion surgery--a randomized control study 2000 [updated 2000//. 1:[25]. Available from: <http://ovidsp.ovid.com/ovidweb.cgi?T=JS&PAGE=reference&D=cctr&NEWS=N&AN=CN-00296603>.

273. Watters Wc TAPGM. The use of dexamethasone in primary lumbar disc surgery. A prospective, randomized, double-blind study 1989 [updated 1989//. 4:[440]. Available from: <http://ovidsp.ovid.com/ovidweb.cgi?T=JS&PAGE=reference&D=cctr&NEWS=N&AN=CN-00059881>.

274. Weinstein JNL, J. D.; Tosteson, T. D.; Zhao, W.; Blood, E. A.; Tosteson, A. N. A.; Birkmeyer, N.; Herkowitz, H.; Longley, M.; Lenke, L.; Emery, S.; Hu, S. S. Surgical compared with nonoperative treatment for lumbar degenerative spondylolisthesis: Four-year results in the Spine Patient Outcomes Research Trial (SPORT) randomized and observational cohorts. Journal of Bone and Joint Surgery - Series A. 2009;91(6):1295-304.

275. White CLP, R. P.; Chan, M. H. An evaluation of the effectiveness of patient-controlled analgesia after spinal surgery. J Neurosci Nurs. 1998;30(4):225-32.

276. Williamson JB, C.; Coutts, F. What do patients feel they can do following lumbar microdiscectomy? A qualitative study. Disabil Rehabil. 2008;30(18):1367-73.

277. Wirth BR, F.; Peterson, C.; Humphreys, B. K.; Farshad, M.; Becker, S.; Schweinhardt, P. An observational study on trajectories and outcomes of chronic low back pain patients referred from a spine surgery division for chiropractic treatment. Chiropr Man Therap. 2019;27:6.

278. Wu Hh TTPQJWYZZK. Percutaneous endoscopic lumbar discectomy combined with epidural injection for prolapsed lumbar disc herniation 2017 [updated 2017//. 2:[110]. Available from: <http://ovidsp.ovid.com/ovidweb.cgi?T=JS&PAGE=reference&D=cctr&NEWS=N&AN=CN-01614721>.

279. Yaman Aktas Y DHDR. Cold Therapy and the Effect on Pain and Physiological Parameters in Patients Recovering from Spine Surgery: a Randomized Prospective Study. Complementary medicine research. 2020.

280. Yao Y-C, Lin H-H, Chang M-C. Bracing Following Transforaminal Lumbar Interbody Fusion is not Necessary for Patients With Degenerative Lumbar Spine Disease: A Prospective, Randomized Trial. Clinical spine surgery. 2018;31(9):E441-E5.

281. Yao Y-C LHHCMC. Bracing Following Transforaminal Lumbar Interbody Fusion is not Necessary for Patients with Degenerative Lumbar Spine Disease: Lippincott Williams and Wilkins; 2018 [updated 2018//. 9:[E441]. Available from: <http://ovidsp.ovid.com/ovidweb.cgi?T=JS&PAGE=reference&D=cctr&NEWS=N&AN=CN-01623728>.

282. Yee AJY, J. U.; Marsolais, E. B.; Carlson, G.; Poe-Kochert, C.; Bohlman, H. H.; Emery, S. E. Use of a postoperative lumbar corset after lumbar spinal arthrodesis for degenerative conditions of the spine. A prospective randomized trial. J Bone Joint Surg Am. 2008;90(10):2062-8.

283. Yee Aj YJUMEBCGP-KCBHHESE. Use of a postoperative lumbar corset after lumbar spinal arthrodesis for degenerative conditions of the spine. A prospective randomized trial 2008 [updated 2008//. 10:[2062]. Available from: <http://ovidsp.ovid.com/ovidweb.cgi?T=JS&PAGE=reference&D=cctr&NEWS=N&AN=CN-00651310>.

284. Yee AJY, J. U.; Marsolais, E. B.; Carison, G.; Poe-Kochert, C.; Bohlman, H. H.; Emery, S. E. Use of a Postoperative Lumbar Corset After Lumbar Spinal Arthrodesis for Degenerative Conditions of the Spine. Journal of Bone & Joint Surgery, American Volume. 2008;90(10):2062-8.

285. Yeh Ml CYCCKMCHH. Pain reduction of acupoint electrical stimulation for patients with spinal surgery: a placebo-controlled study 2011 [updated 2011//. 6:[703]. Available from: <http://ovidsp.ovid.com/ovidweb.cgi?T=JS&PAGE=reference&D=cctr&NEWS=N&AN=CN-00802285>.

286. Yeh MLC, Y. C.; Chen, K. M.; Chen, H. H. Pain reduction of acupoint electrical stimulation for patients with spinal surgery: a placebo-controlled study. Int J Nurs Stud. 2011;48(6):703-9.

287. Zarei M NAMPS-YSSHMMFM. Management of postoperative pain after Lumbar surgery-pregabalin for one day and 14 days-a randomized, triple-blinded, placebo-controlled study: Elsevier B.V.; 2016 [updated 2016//. 37]. Available from: <http://ovidsp.ovid.com/ovidweb.cgi?T=JS&PAGE=reference&D=cctr&NEWS=N&AN=CN-01247688>.

288. Zhang R, Zhang SJ, Wang XJ. Postoperative functional exercise for patients who underwent percutaneous transforaminal endoscopic discectomy for lumbar disc herniation. European review for medical and pharmacological sciences. 2018;22(1 Suppl):15-22.

289. Zhang R ZSJWXJ. Postoperative functional exercise for patients who underwent percutaneous transforaminal endoscopic discectomy for lumbar disc herniation 2018 [updated 2018//. 1 Suppl:[15]. Available from: <http://ovidsp.ovid.com/ovidweb.cgi?T=JS&PAGE=reference&D=cctr&NEWS=N&AN=CN-01665959>.

290. Zhang JW, Y.; Li, S.; Sun, Y. Pestle needling at Yaoyangguan-Bazhen points for intractable lumbodynia after lumbar disc herniation surgery: A randomized controlled trial. World Journal of Acupuncture - Moxibustion. 2019;29(3):194-9.

291. Zhao Bx WKZZJXWCSHXHS-qMQH. Clinical effects of acupuncture after surgical operation in patients with prolapse of the lumbar intervertebral disc 2008 [updated 2008//. 4:[250]. Available from: <http://ovidsp.ovid.com/ovidweb.cgi?T=JS&PAGE=reference&D=cctr&NEWS=N&AN=CN-00688241>.

292. Zhu Y D ZJFWYC. Therapeutic observation of qi-guiding needling plus electroacupuncture for intractable low back pain after lumbar disc herniation surgery and the change of infrared thermal image 2016 [updated 2016//. 3:[311]. Available from: <http://ovidsp.ovid.com/ovidweb.cgi?T=JS&PAGE=reference&D=cctr&NEWS=N&AN=CN-01929288>.

293. Zieger ML, M.; Meisel, H. J.; Gunther, L.; Winkler, D.; Toussaint, R.; Stengler, K.; Angermeyer, M. C.; Konig, H. H.; Riedel-Heller, S. G. The impact of psychiatric comorbidity on the return to work in patients undergoing herniated disc surgery. J Occup Rehabil. 2011;21(1):54-65.

294. Zoia CB, D.; Alicino, C.; Chimenti, M.; Pugliese, R.; Gaetani, P. Usefulness of corset adoption after single-level lumbar discectomy: a randomized controlled trial. J Neurosurg Spine. 2018;28(5):481-5.

295. Nct. Effect of Feedback Health Education on Postoperative Rehabilitation of Patients With Lumbar Disc Herniation. Effect of Feedback Health Education on Postoperative Rehabilitation of Patients With Lumbar Disc Herniation: a Cluster Randomized Trials. 2021.

296. Application of rehabilitation nursing in patients accepting minimally invasive spine surgery and its effects on pain and adverse emotions. Application of rehabilitation nursing in patients accepting minimally invasive spine surgery and its effects on pain and adverse emotions. 2020;13(7):5160.

297. Jprn U. Dietary supplementation in patients following lumbar spine surgery: a randomized controlled trial 2018 [updated 2018//. Available from: <http://ovidsp.ovid.com/ovidweb.cgi?T=JS&PAGE=reference&D=cctr&NEWS=N&AN=CN-01901115>.

298. Chi CI. Multimodal Nutritional Management in Primary Lumbar Spinal surgery: a Randomized Controlled Trial 2017 [updated 2017//. Available from: <http://ovidsp.ovid.com/ovidweb.cgi?T=JS&PAGE=reference&D=cctr&NEWS=N&AN=CN-01901750>.

299. Irct20180404039194N. Study of the effect of gentle stretching exercises and foot reflexology massage on the pain of patients after spinal surgery 2018 [updated 2018//. Available from: <http://ovidsp.ovid.com/ovidweb.cgi?T=JS&PAGE=reference&D=cctr&NEWS=N&AN=CN-01905285>.

300. Jprn U. The Use of Yoga for Lumbar Spine Post-Surgical Pain Management 2018 [updated 2018//. Available from: <http://ovidsp.ovid.com/ovidweb.cgi?T=JS&PAGE=reference&D=cctr&NEWS=N&AN=CN-01950951>.

301. Jprn U. A home video exercise program improves ADL/QOL and kinesiophobia in the patients with lumbar spine surgery A randomized controlled trial 2019 [updated 2019//. Available from: <http://ovidsp.ovid.com/ovidweb.cgi?T=JS&PAGE=reference&D=cctr&NEWS=N&AN=CN-01970358>.

302. Irct20180310039021N. The effect of pain management education on severity of pain, anxiety and disability after lumbar surgery in patients with chronic low back pain 2019 [updated 2019//. Available from: <http://ovidsp.ovid.com/ovidweb.cgi?T=JS&PAGE=reference&D=cctr&NEWS=N&AN=CN-01971658>.

303. ChiCtr. The Effect of Ultrasound-guided Erector Spinae Plane Block (ESPB) on Perioperative Analgesia and Rehabilitation for Posterior Lumbar Interbody Fusion: a Prospective Randomized Controlled Study 2019 [updated 2019//. Available from: <http://ovidsp.ovid.com/ovidweb.cgi?T=JS&PAGE=reference&D=cctr&NEWS=N&AN=CN-01973214>.

304. Nct. Physiotherapy Following Disc Surgery: long Term Follow-up of a RCT 2013 [updated 2013//. Available from: <http://ovidsp.ovid.com/ovidweb.cgi?T=JS&PAGE=reference&D=cctr&NEWS=N&AN=CN-01489542>.

305. Nct. Assessment of Low Level Laser Therapy for Late Postoperative Pain After Lumbar Fusion Surgery 2007 [updated 2007//. Available from: <http://ovidsp.ovid.com/ovidweb.cgi?T=JS&PAGE=reference&D=cctr&NEWS=N&AN=CN-01513989>.

306. Nct. Preoperative Prevention and Early Rehabilitation for Patients Undergoing Elective Spine Surgery 2007 [updated 2007//. Available from: <http://ovidsp.ovid.com/ovidweb.cgi?T=JS&PAGE=reference&D=cctr&NEWS=N&AN=CN-01514919>.

307. Nct. Effect of a Case Manager to Assist the Rehabilitation for Lumbar Spinal Fusion Patients. A Randomised Controlled Trial 2018 [updated 2018//. Available from: <http://ovidsp.ovid.com/ovidweb.cgi?T=JS&PAGE=reference&D=cctr&NEWS=N&AN=CN-01522974>.

308. Nct. Transcranial Direct Current Stimulation in Reduction of Pain and Postoperative Opioids Consumption After Spine Surgery 2017 [updated 2017//. Available from: <http://ovidsp.ovid.com/ovidweb.cgi?T=JS&PAGE=reference&D=cctr&NEWS=N&AN=CN-01592713>.

309. Chi CT. A Randomized,controlled, multicentre Clinical Study to Evaluate the Efficacy and Safety of Smartphone in post-operative rehabilitation after lumbar spine surgeries 2013 [updated 2013//. Available from: <http://ovidsp.ovid.com/ovidweb.cgi?T=JS&PAGE=reference&D=cctr&NEWS=N&AN=CN-01821862>.

310. Actrn. Lumbar microdiscectomy and post-operative activity restrictions 2016 [updated 2016//. Available from: <http://ovidsp.ovid.com/ovidweb.cgi?T=JS&PAGE=reference&D=cctr&NEWS=N&AN=CN-01842723>.

311. Abdi A, Bagheri SR, Shekarbeigi Z, Usefvand S, Alimohammadi E. The effect of repeated flexion-based exercises versus extension-based exercises on the clinical outcomes of patients with lumbar disk herniation surgery: a randomized clinical trial. Neurol Res. 2023;45(1):28-40.

312. Aldemir K, Gurkan A. The effect of pedometer-supported walking and telemonitoring after disc hernia surgery on pain and disability levels and quality of life. International journal of nursing practice. 2021;27(2):e12917.

313. Beneck GJ, Popovich JM, Jr., Selkowitz DM, Azen S, Kulig K. Intensive, progressive exercise improves quality of life following lumbar microdiskectomy: a randomized controlled trial. Clin Rehabil. 2014;28(9):892-901.

314. Bono CM, Leonard DA, Cha TD, Schwab JH, Wood KB, Harris MB, et al. The effect of short (2-weeks) versus long (6-weeks) post-operative restrictions following lumbar discectomy: a prospective randomized control trial. Eur Spine J. 2017;26(3):905-12.

315. Choi G, Raiturker PP, Kim MJ, Chung DJ, Chae YS, Lee SH. The effect of early isolated lumbar extension exercise program for patients with herniated disc undergoing lumbar discectomy. Neurosurgery. 2005;57(4):764-72; discussion -72.

316. Erdogan Z, Bulut H. Effectiveness of Computer Assisted Training of Patients Undergoing Lumbar Disc Herniation Surgery. Turk Neurosurg. 2020;30(1):69-77.

317. Erdogmus CB, Resch KL, Sabitzer R, Müller H, Nuhr M, Schöggl A, et al. Physiotherapy-based rehabilitation following disc herniation operation: results of a randomized clinical trial. Spine (Phila Pa 1976). 2007;32(19):2041-9.

318. Ebenbichler GR, Inschlag S, Pflüger V, Stemberger R, Wiesinger G, Novak K, et al. Twelve-year follow-up of a randomized controlled trial of comprehensive physiotherapy following disc herniation operation. Clin Rehabil. 2015;29(6):548-60.

319. Fu S, Ji X. Effect of traditional Chinese medicine-based nursing intervention on gastrointestinal function, psychological mood, and pain in patients after surgery for lumbar disc herniation. World Chinese Journal of Digestology. 2017;25(25):2296.

320. Häkkinen A, Ylinen J, Kautiainen H, Tarvainen U, Kiviranta I. Effects of home strength training and stretching versus stretching alone after lumbar disk surgery: a randomized study with a 1-year follow-up. Arch Phys Med Rehabil. 2005;86(5):865-70.

321. He Q, Zhao J, Fan M, Wang F. Effect of continuous nursing based on wechat platform on postoperative rehabilitation of patients with lumbar disc herniation. Japan journal of nursing science : JJNS. 2021;18(2):e12382.

322. Jentoft ES, Kvale A, Assmus J, Moen VP. Effect of information and exercise programmes after lumbar disc surgery: A randomized controlled trial. Physiotherapy research international : the journal for researchers and clinicians in physical therapy. 2020;25(4):e1864.

323. Kulikov AG, Voronina DD, Morozov AE, Zajceva TN, Ustinova KI. New approach for evaluating the effectiveness of whole-body magnetic field therapy in the rehabilitation of patients with lumbar discectomy. J Back Musculoskelet Rehabil. 2018;31(6):1065-73.

324. Lu Z, Bai J. A prospective study on the application of staged lumbar motion chain rehabilitation based on mckenzie's technique after lumbar percutaneous transforaminal endoscopic discectomy. A prospective study on the application of staged lumbar motion chain rehabilitation based on mckenzie's technique after lumbar percutaneous transforaminal endoscopic discectomy. 2020;25(9):1398.

325. Manniche C, Skall HF, Braendholt L, Christensen BH, Christophersen L, Ellegaard B, et al. Clinical trial of postoperative dynamic back exercises after first lumbar discectomy. Spine (Phila Pa 1976). 1993;18(1):92-7.

326. Oosterhuis T, Ostelo RW, van Dongen JM, Peul WC, de Boer MR, Bosmans JE, et al. Early rehabilitation after lumbar disc surgery is not effective or cost-effective compared to no referral: a randomised trial and economic evaluation. J Physiother. 2017;63(3):144-53.

327. Ostelo RW, de Vet HC, Berfelo MW, Kerckhoffs MR, Vlaeyen JW, Wolters PM, et al. Effectiveness of behavioral graded activity after first-time lumbar disc surgery: short term results of a randomized controlled trial. Eur Spine J. 2003;12(6):637-44.

328. Ostelo RW, de Vet HC, Vlaeyen JW, Kerckhoffs MR, Berfelo WM, Wolters PM, et al. Behavioral graded activity following first-time lumbar disc surgery: 1-year results of a randomized clinical trial. Spine (Phila Pa 1976). 2003;28(16):1757-65.

329. Paulsen RT, Carreon LY, Andersen M. Patient-reported Outcomes After Surgery for Lumbar Disc Herniation, a Randomized Controlled Trial Comparing the Effects of Referral to Municipal Physical Rehabilitation Versus No Referral. Spine (Phila Pa 1976). 2019;45(1):3-9.

330. Paulsen RT, Rasmussen J, Carreon LY, Andersen M. Return to work after surgery for lumbar disc herniation, secondary analyses from a randomized controlled trial comparing supervised rehabilitation versus home exercises. Spine J. 2020;20(1):41-7.

331. Pekař L, Steindler J. Systémová enzymoterapie po operacích výhřezu bederní meziobratlové ploténky. Klinická farmakologie a farmacie. 2010;23(4):166-70.

332. Skall FH, Manniche C, Nielsen CJ. [Intensive back exercises 5 weeks after surgery of lumbar disk prolapse. A prospective, randomized multicenter trial with a historical control group]. Ugeskr Laeger. 1994;156(5):643-6.

333. Uysal E, Cine HS, Cetin E. The necessity and timing of exercise after lumbar disc herniation surgery. Eur Rev Med Pharmacol Sci. 2023;27(20):9521-9.

334. Zarei M, Najafi A, Mansouri P, Sadeghi-Yazdankhah S, Saberi H, Moradi M, et al. Management of postoperative pain after Lumbar surgery-pregabalin for one day and 14 days-a randomized, triple-blinded, placebo-controlled study. Clin Neurol Neurosurg. 2016;151:37-42.

335. Zhao BX, Wang KZ, Zhao JX, Wang CS, Huang XH, Shu-qiang M, et al. Clinical effects of acupuncture after surgical operation in patients with prolapse of the lumbar intervertebral disc. J Tradit Chin Med. 2008;28(4):250-4.

336. Zhang R, Zhang SJ, Wang XJ. Postoperative functional exercise for patients who underwent percutaneous transforaminal endoscopic discectomy for lumbar disc herniation. Eur Rev Med Pharmacol Sci. 2018;22(1 Suppl):15-22.

337. Zheng SW, Shaikh Atik B, Wang J, Jiang H, Li M, Chen GF. [Effect of supplementing qi, blood-activating and kidney-nourishing therapy on postoperative recovery in patients with lumber disc herniation]. Nan Fang Yi Ke Da Xue Xue Bao. 2015;35(1):137-40.

338. Zuo Q, Cao Y, He S, Min J. Application of cognitive behavior therapy combined with Pilates exercise in postoperative patients with lumbar intervertebral disc herniation. Chinese Nursing Research. 2021;35(16):2852-7.
